# Crystal Engineering of Chelating Hybrid Ultramicroporous Materials via Pillar Modulation for Energy‐Efficient Acetylene Separation

**DOI:** 10.1002/smll.74171

**Published:** 2026-06-12

**Authors:** Asif Raza, Julia Korenko, Sousa Javan Nikkhah, Sayan Maiti, Debobroto Sensharma, Lilia Croitor, Kui Tan, Matthias Vandichel, Michael J. Zaworotko, Soumya Mukherjee

**Affiliations:** ^1^ Bernal Institute and Research Ireland Centre for Pharmaceuticals (SSPC) Department of Chemical Sciences University of Limerick Limerick Ireland; ^2^ Department of Chemistry Kathleen Lonsdale Institute Maynooth University Maynooth Kildare Ireland; ^3^ Department of Chemistry University of North Texas Denton Texas USA; ^4^ Department of Chemistry and Biochemistry The University of Texas at El Paso El Paso Texas USA

**Keywords:** acetylene selectivity, crystal engineering, gas separation, hybrid ultramicroporous materials, physisorbents, porous material

## Abstract

Acetylene, C_2_H_2,_ a commodity chemical, is a building block for producing plastics, synthetic rubbers, and pharmaceuticals. Among emerging classes of recyclable adsorbents, hybrid ultramicroporous materials (HUMs) demonstrate C_2_H_2_‐selective physisorption, yet their C_2_H_2_ over CO_2_ (C_2_H_2_/CO_2_) separation performances are often constrained by trade‐offs between uptake capacity, selectivity, ease in regeneration, and stability under humid conditions. Expanding upon the prototypal chelating ligand *N* ^1^,*N* ^2^‐bis(pyridine‐4‐ylmethyl)ethane‐1,2‐diamine (enmepy)‐derived HUM family [Zn(enmepy)(MF_6_)]_n_, **MFSIX‐enmepy‐Zn**, through systematic pillar modulation, three previously unreported HUMs are isolated as single crystals. [Zn(enmepy)(SnF_6_)]_n_, **SNIFSIX‐enmepy‐Zn**, offers limited stability, whereas [Zn(enmepy)(TiF_6_)]_n_, **TIFSIX‐enmepy‐Zn**, and [Zn(enmepy)(NbOF_5_)]_n_, **NbOFFIVE‐enmepy‐Zn** are more robust. Gas sorption isotherms, sorption kinetics, dynamic column breakthrough experiments, and molecular modelling identify **NbOFFIVE‐enmepy‐Zn** as offering a combination of high C_2_H_2_/CO_2_ selectivity (>5) with low regeneration energy requirements (≈31 kJ mol^−^^1^) and superior hydrolytic stability (>7 days, 75% relative humidity). Further, **NbOFFIVE‐enmepy‐Zn** delivers effective C_2_H_2_/CO_2_ separation (a separation factor > 5) under both dry and humid conditions, underscoring the importance of fine‐tuning inorganic pillars in balancing adsorption performance and material robustness.

## Graphical abstract

A new chelating hybrid ultramicroporous material platform is developed using anion modulation to fine‐tune pore chemistry and binding environments, enabling selective C_2_H_2_ capture over CO_2_. One of them, **NbOFFIVE‐enmepy‐Zn**, exhibits multiple binding interactions for C_2_H_2_ vs. a single CO_2_ adsorption mode, enabling selective C_2_H_2_ capture with easy regeneration, thus highlighting how targeted anion substitution could profoundly impact separation performance.

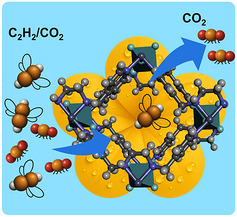

## 1 Introduction

Acetylene (C_2_H_2_) is an industrial feedstock used in the production of value‐added chemicals, such as vinyl chloride, acrylonitrile, and vinyl acetate [1, 2]. It is typically produced through incomplete combustion of methane or thermal cracking of hydrocarbons, processes that inevitably afford carbon dioxide (CO_2_) as a significant byproduct. The presence of CO_2_ is known to disrupt downstream reactions, requiring separation of high‐purity C_2_H_2_ (>99.5%) from C_2_H_2_/CO_2_ mixtures [3, 4]. However, this is intrinsically challenging because C_2_H_2_ and CO_2_ possess nearly identical molecular sizes (kinetic diameters ≈ 3.3 Å), shapes, and boiling points (189.2 K for C_2_H_2_ and 194.7 K for CO_2_) (Scheme S1). Conventional purification techniques, including cryogenic distillation and solvent extraction, are both energy and capital‐intensive. To mitigate these drawbacks, high‐performing C2 physisorbents that are recyclable under low‐energy regeneration conditions have lately emerged as energy‐efficient alternatives [3, 5].

Among physisorbents, metal–organic materials (MOMs) [6], including porous coordination polymers [7, 8], metal–organic frameworks (MOFs) [9], and hybrid ultramicroporous materials (HUMs) stand out as particularly attractive for designing C_2_H_2_‐selective sorbents [3, 10, 11]. Their modular compositions lend themselves well to crystal engineering strategies that can be developed from first principles [3]. HUMs are typically sustained by two‐dimensional (2D) square lattice (**sql**) coordination networks pillared by inorganic anions (e.g., SiF_6_^2−^: hexafluorosilicate, and NbOF_5_^2−^: oxopentafluoroniobate), which give rise to three‐dimensional (3D) primitive cubic (**pcu**) topology networks with predictable pore chemistry and size [12, 13]. Thanks to a high density of tight sorbate binding sites, prototypical HUMs have established benchmark C_2_H_2_/CO_2_ and C_2_H_2_/C_2_H_4_ separation performances. For instance, [Cu(4,4′‐dipyridylacetylene)_2_SiF_6_]_n_, **SIFSIX‐2‐Cu‐i** has optimal pore size (5.2 Å × 5.2 Å, thanks to interpenetration) and pore chemistry (electrostatic interactions) [12], and related next‐generation HUMs afford high‐purity CO_2_ and ethylene (C_2_H_4_) streams during dynamic separation experiments [3, 14, 15].

More broadly, thanks to pillar modulation, isostructural HUMs can be fine‐tuned to impart optimal sorbate–sorbent interactions, which can afford thermodynamic *sweet spots*, offering binding sites that can balance high adsorption selectivity with low regeneration energy penalty, exemplified by trace CO_2_ capture records held by [Ni(pyrazine)_2_SiF_6_]_n_, **SIFSIX‐3‐Ni**, and [Ni(pyrazine)_2_TiF_6_]_n_, **TIFSIX‐3‐Ni** [3, 16, 17]. Despite these advances in properties, translating HUMs to higher technological readiness levels (TRLs) has been hindered by limited hydrolytic stability. For example, stemming from their hydrolytically labile metal‐fluorine bonds, prototypal **pcu**‐topology HUMs, such as [Cu(4,4′‐bipyridine)_2_SiF_6_]_n_, **SIFSIX‐1‐Cu**, and **SIFSIX‐3‐Ni**, undergo phase conversions from porous 3D **pcu** to non‐porous 2D **sql** networks upon exposure to humidity [18, 19]. Starting from these hydrolytically unstable blueprints, enhancing hydrolytic stability is therefore essential to enable the utility of HUMs in light hydrocarbon separations, including but not limited to C_2_H_2_‐selective HUMs. An effective countermeasure involves anion modulation to enhance the moisture stability of HUMs. This approach has resulted in HUMs such as [Ni(pyrazine)_2_AlF_5_]_n_, **ALFFIVE‐1‐Ni**, and [Ni(pyrazine)_2_FeF_5_]_n,_ **FeFFIVE‐1‐Ni**, that exhibit resistance to moisture, in contrast to the isostructural **SIFSIX‐3‐Ni** [20].

In parallel, although uncommon, HUMs can exhibit enhanced moisture stability through judicious selection of their organic linker ligands [19, 21, 22]. HUMs leveraging this crystal engineering approach generally exploit ditopic linker ligands (e.g., pyrazine and 4,4′‐bipyridine), enabling such HUMs to exhibit **pcu** topology with nearly cylindrical one‐dimensional (1D) channels. By contrast, the use of polytopic ligands has more recently led to alternative topologies (e.g., **fsc, ith‐d, znv, wly**), introducing new channel architectures and structure–sorption relationships [21, 23, 24, 25, 26, 27, 28]. However, utilization of polytopic chelating ligands remains underexplored (Table S1 and Figures S1–S5): 3.74% of the anion‐pillared coordination networks in the Cambridge Structural Database [29]. Chelating ligands offer coordination modes distinct from prototypal ditopic linkers, thus leading to unusual HUM topologies, e.g., **dia** [30]; They can also enable enhanced heterogeneity from the chelating atoms (e.g., O, N) [31], potentially impacting the sorbate binding sites. Recently, our group introduced a pair of chelating ethylenediamine derivative linker ligands‐sustained HUMs as sorbents, [Zn(enmepy)(SiF_6_)]_n_ (**SIFSIX‐24‐Zn**, i.e., **SIFSIX‐enmepy‐Zn**) and [Zn(enmepy)(SO_4_)]_n_ (**SOFOUR‐2‐Z**n, i.e., **SOFOUR‐enmepy‐Zn**) [31, 32], a compositionally modular Generation‐1 platform [31]. Building on this platform of chelating‐ligand‐derived HUMs (hereinafter regarded as *chelating HUMs*), we now expand the library to a Generation‐2 platform by anion modulation to systematically fine‐tune pore size, pore chemistry, and moisture stability. By substituting the SiF_6_^2−^ anions in **SIFSIX‐ enmepy‐Zn** with TiF_6_^2−^, SnF_6_^2−^, and NbOF_5_^2−^, we obtained three new members of this chelating HUM family: [Zn(enmepy)(TiF_6_)]_n_ (**TIFSIX‐enmepy‐Zn**), [Zn(enmepy)(SnF_6_)]_n_ (**SNIFSIX‐enmepy‐Zn**), and [Zn(enmepy)(NbOF_5_)]_n_ (**NbOFFIVE‐enmepy‐Zn**). As reported herein, we have evaluated these HUMs for C_2_H_2_/CO_2_ separation studies under both dry and humid conditions, alongside static and dynamic conditions.

## 2 Results and Discussion

### 2.1 Synthesis and Characterization

**FIGURE 1:**
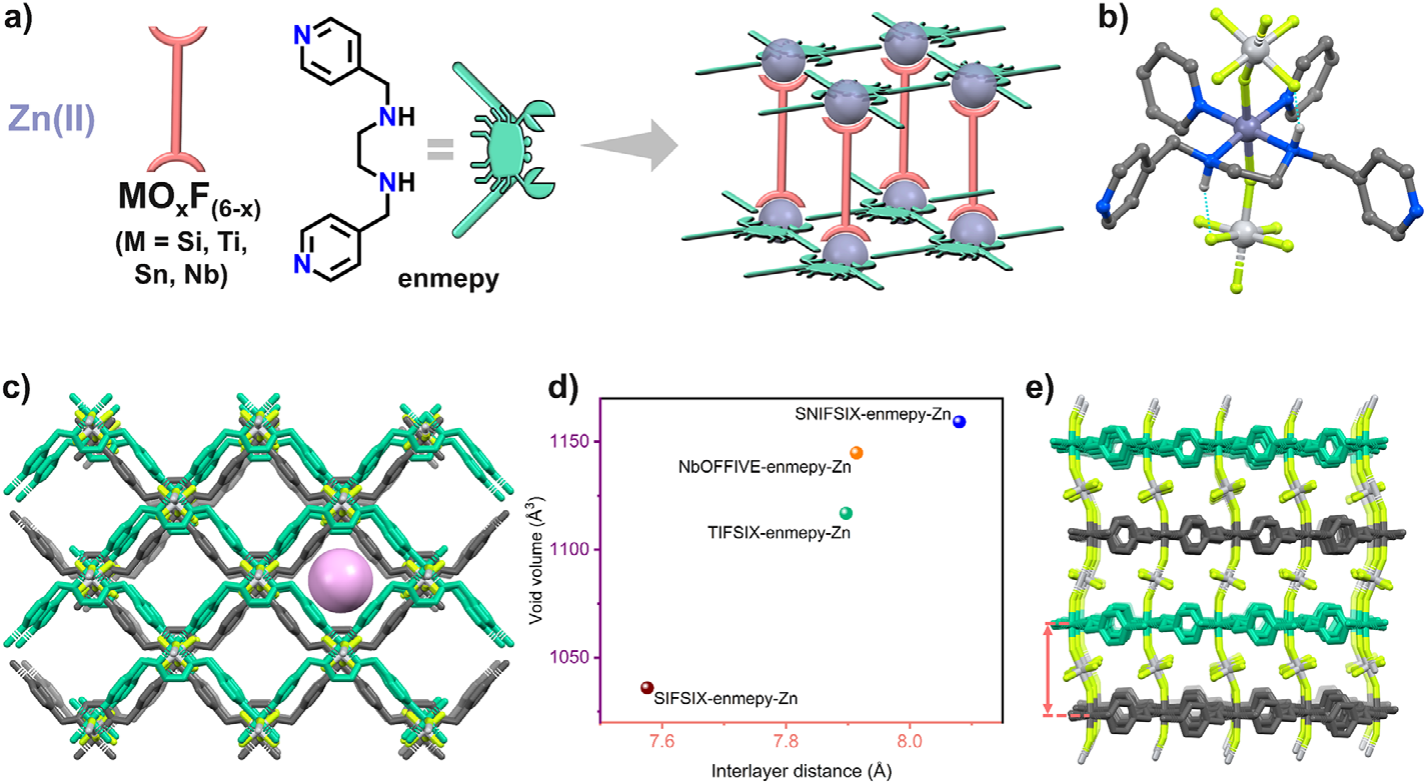
Structural analysis of the enmepy‐based chelating HUMs reported herein: (a) chelating enmepy ligands coordinate Zn(II) to generate 2D **sql** layers (marked in green). (b) Coordination environment around Zn(II) with TiF_6_^2−^(**TIFSIX‐enmepy‐Zn** is an exemplar): H‐bonding interactions between amine protons and fluoride anions are shown in green dotted lines. (c) Crystal packing of **TIFSIX‐enmepy‐Zn** viewed along the *c*‐axis, illustrating alternating **sql** layers (green, top; gray, and bottom) that define a uniform 1D pore channel (lilac sphere). (d) Void volume vs. interlayer Zn–Zn distance in this chelating HUM family, highlighting the impact of anion substitution (SiF_6_^2−^, TiF_6_^2−^, NbOF_5_^2−^, and SnF_6_^2−^) on pore volume. (e) Crystal packing of **TIFSIX‐enmepy‐Zn** viewed along the *a*‐axis, showing alternating **sql** layers (green, top; gray, bottom) and zig‐zag arrangement of anions.

Single crystals of **NbOFFIVE‐enmepy‐Zn**, **SNIFSIX‐enmepy‐Zn,** and **TIFSIX‐enmepy‐Zn** were obtained by layering a methanolic solution of **enmepy** onto aqueous solutions of the corresponding zinc salts ([ZnXO_y_F_(6‐y)_·6H_2_O]; X = Ti, Sn, Nb) with a 1:1 (*v*/*v*) buffer layer of methanol/water in between, yielding colourless crystals after one week (see Supporting Information, for detailed synthesis methods). Single‐crystal X‐ray diffraction (SCXRD) studies reveal that the resulting HUMs crystallized as **pcu** nets in the monoclinic space group *C*2/*c* (Table S2). Figure 1a reveals that the enmepy linker coordinates to Zn(II) octahedral nodes through both pyridyl and secondary amine nitrogen atoms: ethylenediamine moieties chelate to metal centres; pyridyl nitrogen atoms connect adjacent Zn(II) nodes to generate **sql** layers (Figure S6). These layers self‐assemble through π‐stacking in an alternating fashion and are pillared by octahedral inorganic pillars to afford a three‐dimensional framework (Figure 1b) that features 1D channels with pore‐limiting diameters as follows: 3.99 Å (**TIFSIX‐enmepy‐Zn**) < 4.03 Å (**SNIFSIX‐enmepy‐Zn**) < 4.10 Å (**NbOFFIVE‐enmepy‐Zn**) < 4.34 Å (**SIFSIX‐enmepy‐Zn**), calculated using CCDC Mercury pore analyser with helium probe (Figure 1c). The alternating 2D layers elicit an offset that leads to tilted anionic pillaring, while the previously reported tetrahedral pillar analogues, e.g., stemming from SO_4_^2−^ such as **SOFOUR‐enmepy‐Zn**, exhibit non‐alternating layers (Figure 1e; Figure S7). In the chelating HUMs we report herein, the offset layers exhibit N─H···F hydrogen bonds between amine NH groups and fluoride moieties (Figure 1b). H‐bonding distances are 3.0 Å (**TIFSIX‐enmepy‐Zn**) < 3.04 Å (**NbOFFIVE‐enmepy‐Zn**) < 3.1 Å (**SNIFSIX‐enmepy‐Zn**) < 3.23 Å (**SIFSIX‐enmepy‐Zn**).

These intramolecular H‐bonding interactions facilitate the alternating arrangement of anions between adjacent layers (Table S4; Figure 1e). Differences in the pillaring angles (Table S3; Figure S8) and X─F bond distances (X = Si, Ti, Nb, Sn) lead to variations in the interlayer Zn─Zn distances, which in turn increase as follows: SiF_6_^2−^ (7.5 Å) < TiF_6_^2−^ (7.8 Å)< NbOF_5_^2−^ (7.9 Å) < SnF_6_^2−^ (8.0 Å) (Figure S9). These subtle sub‐angstrom changes impact the accessible porosity as reflected in their void fractions and void volumes per unit cell (CCDC Mercury contact surface [33], using a probe radius of 1.2 Å): void fractions are 37.9% (**SIFSIX‐24‐Zn**) < 41.6% (**TIFSIX‐enmepy‐Zn**) < 42.4% (**NbOFFIVE‐enmepy‐Zn**) < 43% (**SNIFSIX‐enmepy‐Zn**); void volumes follow the same increasing order of 1035 Å^3^ (**SIFSIX‐24‐Zn**) < 1116 Å^3^ (**TIFSIX‐enmepy‐Zn**) < 1144 Å^3^ (**NbOFFIVE‐enmepy‐Zn**) < 1159 Å^3^ (**SNIFSIX‐enmepy‐Zn**) (Figure 1d). These results further exemplify how subtle structural tweaks can impact porosity in HUMs, aligned with our earlier findings on organic linker ligands‐driven crystal engineering design of C2‐selective HUMs [15].

Bulk samples of **NbOFFIVE‐enmepy‐Zn**, **SNIFSIX‐enmepy‐Zn**, and **TIFSIX‐enmepy‐Zn** were prepared by mixing a methanolic solution of enmepy with aqueous solutions of zinc salts [ZnXO_y_F_(6‐y)_·6H_2_O; X = Ti, Sn, Nb] at room temperature to obtain block‐shaped microcrystalline powders (Figures S10 and S11) (see Supporting Information for more details on synthesis) with powder X‐ray diffraction (PXRD) patterns matching those calculated from SCXRD data, consistent with phase purity (Figures S12–S15). Thermogravimetric analysis (TGA) of **TIFSIX‐enmepy‐Zn**, **NbOFFIVE‐enmepy‐Zn**, **SNIFSIX‐enmepy‐Zn** indicated loss of solvent molecules at 90°C, 60°C, and 90°C respectively (Figures S16–S18). **SNIFSIX‐enmepy‐Zn** was found to be unstable upon guest removal, whereas variable temperature powder X‐ray diffraction (VT‐PXRD) revealed that **TIFSIX‐enmepy‐Zn** and **NbOFFIVE‐enmepy‐Zn** remained crystalline to 300°C when heated under N_2_ atmosphere (Figures S19 and S20). These diffractograms (Figures S19 and S20) are consistent with the TGA degradation temperatures (Figures S16 and S18), and highlight the thermal stability of these two HUMs.

### 2.2 Pure Gas and Vapour Adsorption Studies

**FIGURE 2:**
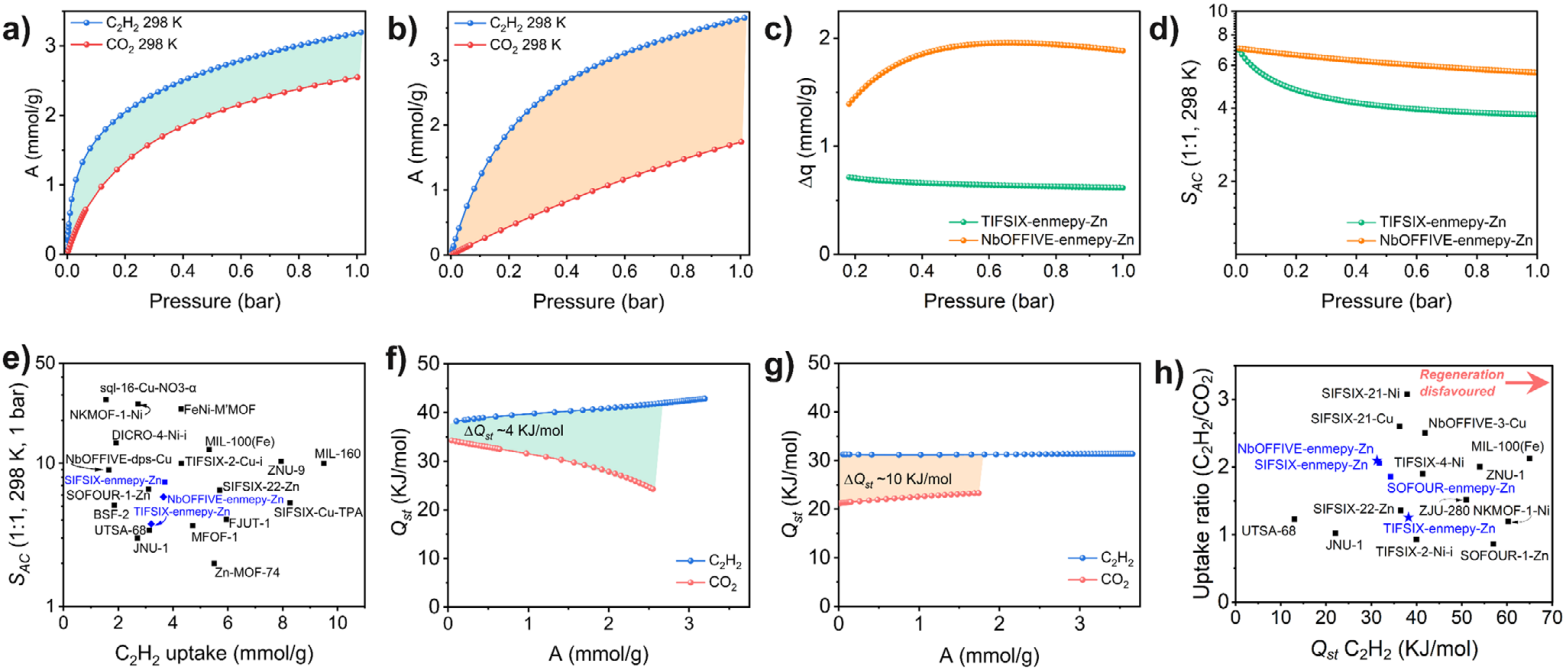
Gas sorption properties of **TIFSIX‐enmepy‐Zn** and **NbOFFIVE‐enmepy‐Zn**: (a, b) 298 K C_2_H_2_ (blue) and CO_2_ (red) pure gas adsorption isotherms for **TIFSIX‐enmepy‐Zn** and **NbOFFIVE‐enmepy‐Zn**, respectively; (c) comparison of uptake capacity difference (Δ*q* = C_2_H_2_ uptake−CO_2_ uptake) for C_2_H_2_ vs. CO_2_; (d) IAST selectivity for C_2_H_2_ over CO_2_ (*S*_AC_) at 298 K and 1 : 1 (*v*/*v*) mixture; (e) comparative analysis of the leading C_2_H_2_‐selective sorbents with respect to *S*_AC_ vs. C_2_H_2_ uptake (cf. Table S7); (f,g) *Q*_st_(C_2_H_2_) and *Q*_st_(CO_2_) profiles for **TIFSIX‐enmepy‐Zn** and **NbOFFIVE‐enmepy‐Zn** respectively; (h) Comparison of *Q*_st_(C_2_H_2_) at low loading and uptake ratios at 1 bar for the best‐performing C_2_H_2_/CO_2_ separating sorbents.

Following activation at 80°C under dynamic vacuum, pure gas sorption isotherms were recorded to evaluate adsorption properties. Permanent microporosity was confirmed through collecting isotherms at 195 K, **TIFSIX‐enmepy‐Zn** and **NbOFFIVE‐enmepy‐Zn** were found to adsorb 5.01 and 8.13 mmol g^−1^ of CO_2_ at 1 bar, respectively, with typical isotherm profiles (Figures S21 and S22). The Brunauer–Emmett–Teller (BET) surface areas were determined to be 415 and 675 m^2^ g^−1^, respectively (Figures S23 and S24). The Horvath–Kawazoe pore size distributions from 195 K CO_2_ isotherms were found to be centred around 5.0 Å, classifying them as ultramicroporous (Figures S25 and S26), consistent with their crystallographically determined pore limiting diameters (Figure 1c). At 298 K, CO_2_ uptakes at 1 bar were 2.54 mmol g^−^^1^ for **TIFSIX‐enmepy‐Zn** and 1.74 mmol g^−^^1^ for **NbOFFIVE‐enmepy‐Zn**, whereas C_2_H_2_ uptakes reached 3.19 and 3.65 mmol g^−^^1^, respectively (Figure 2a,b).

**FIGURE 3:**
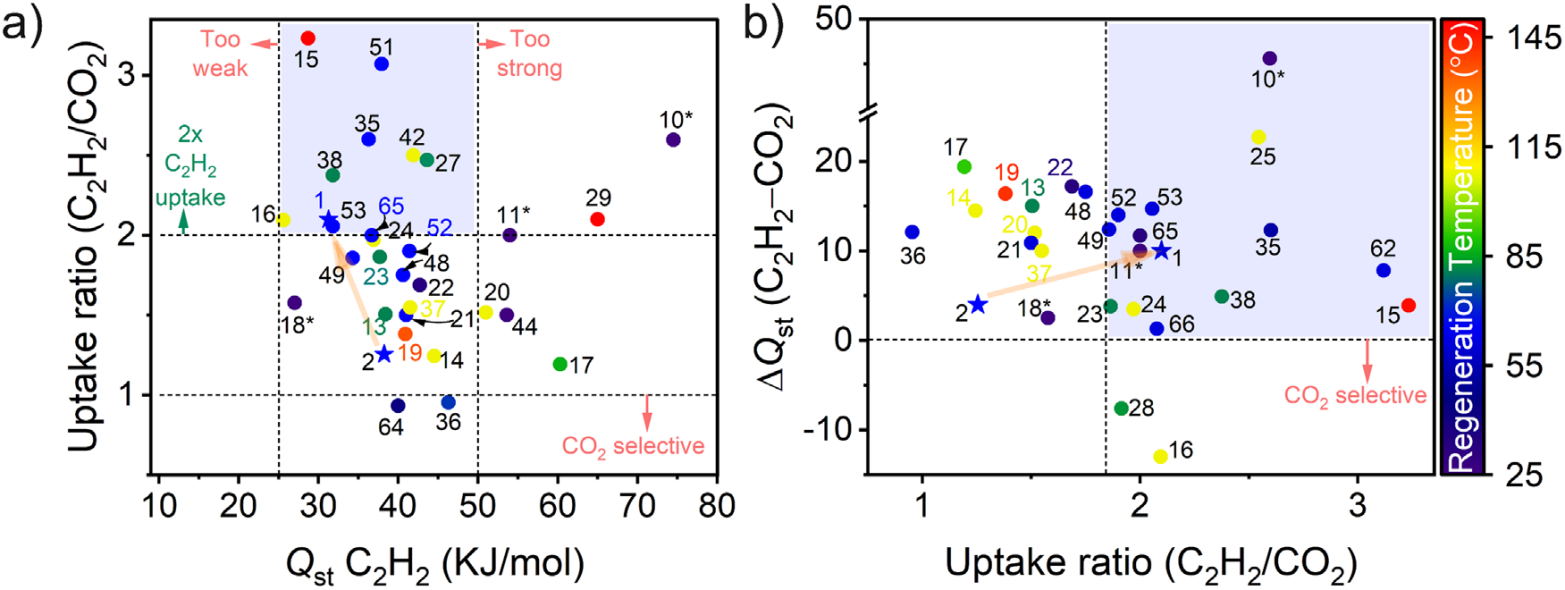
Key properties' landscape for C_2_H_2_/CO_2_ separation in **NbOFFIVE‐enmepy‐Zn (1**★) compared to the lead sorbents that exhibit C_2_H_2_/CO_2_ separation. Each data point is labelled with a serial number corresponding to the materials listed in Table S7: (a) C_2_H_2_/CO_2_ uptake ratio at 1 bar plotted against *Q*_st_(C_2_H_2_); the shaded region highlights the zone featuring optimal binding energies (25–50 kJ mol^−^^1^). (b) Δ*Q_st_* plotted against C_2_H_2_/CO_2_ uptake ratio; the shaded upper‐right zone highlights two features together: favoured adsorption (uptake capacity) and positive Δ*Q_st_*, corresponding to selective C_2_H_2_ binding (thermodynamics). Data points are colour‐coded by regeneration temperature (* = regeneration under vacuum, rest regenerated under inert gas flow).

These data indicate that **NbOFFIVE‐enmepy‐Zn** combines higher C_2_H_2_ uptake with lower CO_2_ affinity, yielding a high C_2_H_2_–CO_2_ uptake capacity‐difference (Δq) of 1.9 mmol g^−^^1^ (at 1 bar) (Figure 2c). This difference is comparable with several leading C_2_H_2_ sorbents, including **FeNi‐M′MOF** (1.6 mmol g^−1^) [34], **GEFSIX‐4‐Zn** (1.0 mmol g^−1^) [23], **BSF‐4** (0.78 mmol  g^−1^) [35], **NKMOF‐1‐Ni** (0.44 mmol g^−1^) [36]. High‐uptake sorbents such as **ZNU‐9** and **SIFSIX‐Cu‐TPA** achieve higher C_2_H_2_ uptakes of about 8 mmol g^−1^, at 298 K and 1 bar; and register C_2_H_2_–CO_2_ uptake differences of 3.6 and 3.5 mmol g^−1^, respectively. However, these sorbents also adsorb large amounts of CO_2_ (4.3 and 4.8 mmol g^−1^ for **ZNU‐9** and **SIFSIX‐Cu‐TPA**, respectively), which impacts their overall C_2_H_2_ selectivity [25, 28]. The Ideal adsorbed solution theory (IAST) selectivity for C_2_H_2_/CO_2_ (1:1 (*v*/*v*), 298 K, 1 bar) for **TIFSIX‐enmepy‐Zn** and **NbOFFIVE‐enmepy‐Zn** were 3.7 and 5.8, respectively, suggesting that selectivity can be improved through pillar substitution (Figure 2d; Figure S27 and Tables S5 and S6). This value is comparable to several C_2_H_2_‐selective sorbents, including **SIFSIX‐Cu‐TPA** (5.3) [25], **ZNU‐9** (3.7) [28], **TIFSIX‐6‐Zn** (5.3) [23], higher than **Zn‐MOF‐74** (2.0), **JNU‐1** (3.0), and **UTSA‐68** (3.4) (Figure 2e; Table S7). Thermodynamic insights from isosteric enthalpies of adsorption (*Q_st_*) were obtained from a virial fit of the single‐component adsorption isotherms recorded at 298, 283, and 273 K (Figures S28–S35), further highlighting these trends. For C_2_H_2_, *Q_st_* values at low loading (*Q*_st_(C_2_H_2_)) were determined to be 38.2 kJ mol^−^^1^ (**TIFSIX‐enmepy‐Zn**) and 31.2 kJ mol^−^^1^ (**NbOFFIVE‐enmepy‐Zn**). For CO_2_, the corresponding values were found to be 34.2 and 21.2 kJ mol^−^^1^, respectively. *Q_st_* (C_2_H_2_) in **NbOFFIVE‐enmepy‐Zn** is lower than in previously reported analogues, **SIFSIX‐enmepy‐Zn** (31.8 kJ mol^−^^1^), and **SOFOUR‐enmepy‐Zn** (34.3 kJ mol^−^^1^) [31]. When benchmarked against other C_2_H_2_‐selective adsorbents, e.g. **SIFSIX‐21‐Cu** (36.3 k  mol^−^^1^) [37], **TIFSIX‐4‐Cu** (40.6 kJ mol^−^^1^) [37], **SOFOUR‐1‐Zn** (57.0 kJ mol^−^^1^) [38], **UTSA‐300a** (57.6 kJ mol^−^^1^) [39], and **NbOFFIVE‐3‐Cu** (41.9 kJ mol^−^^1^) [37], **NbOFFIVE‐enmepy‐Zn** nevertheless maintains a significant difference between the *Q_st_* values of C_2_H_2_ and CO_2_ (Δ*Q_st_* = *Q_st_* (C_2_H_2_) – *Q_st_* (CO_2_) ≈ 10 kJ mol^−^^1^; Figure 2e). Conversely, **TIFSIX‐enmepy‐Zn** interacts with both gases almost equally (Δ*Q_st_* ≈ 4 kJ mol^−^^1^; Figure 2d). As shown in Figure 2h, the combination of a high C_2_H_2_/CO_2_ uptake ratio (uptake of C_2_H_2_ at 1 bar/uptake of CO_2_ at 1 bar) and moderate binding energy indicates that **NbOFFIVE‐enmepy‐Zn** can achieve good separation performance with a reduced energy penalty for regeneration. The energetic *sweet spot* of C_2_H_2_ binding in HUMs is typically reflected in *Q_st_* (C_2_H_2_) between 25 and 50 kJ mol^−^^1^, which can be described as neither too strong nor too weak (Table S7) [40]. In this vein, Figure 3a,b highlights how anion modulation positions **NbOFFIVE‐enmepy‐Zn** (1★) within the zone of optimal performance (marked in transparent blue), outperforming **TIFSIX‐enmepy‐Zn** (2★) and several other leading C_2_H_2_/CO_2_ separating sorbents. Collectively, these results identify **NbOFFIVE‐enmepy‐Zn** as a well‐balanced C_2_H_2_/CO_2_ separating sorbent that balances the trifecta− uptake capacity, selectivity, and regenerability.

To study adsorption kinetics and regenerability under dynamic conditions, we conducted gravimetric kinetic adsorption‐desorption cycling experiments (at 303 K, ≈1 bar) by flowing analyte gases into a TGA balance preloaded with a sorbent. **TIFSIX‐enmepy‐Zn** reached its maximum C_2_H_2_ adsorption capacity in 115 min; however, desorption at 60°C remained incomplete even after 90 min (Figure S36). Full regeneration required a higher temperature: at 120°C, enabling complete desorption within 60 min. The maximum adsorption capacity was fully retained over multiple cycles (Figure S37). In contrast, CO_2_ adsorption in **TIFSIX‐enmepy‐Zn** was moderate and overall slower, quick to adsorb in the beginning but reaching saturation only after prolonged exposure, and showed a gradual decline in capacity upon cycling (Figure S38). Simply put, whereas C_2_H_2_ offers complete regenerability, CO_2_ regenerability is somewhat limited, despite identical temperature swing‐based desorption at 120°C.

Instead, **NbOFFIVE‐enmepy‐Zn** exhibited a distinct behaviour, not only regenerating fully to both sorbates, but under reduced temperature swing conditions of 60°C. As presented in Figures S39 and S40, C_2_H_2_ adsorption was relatively slow but could be fully regenerated at 60°C over multiple cycles, whereas CO_2_ uptake was minimal with fast saturation. To ensure that this minimal CO_2_ uptake was not an artefact of particle size, we tested two independently prepared batches of **NbOFFIVE‐enmepy‐Zn**. Both batches reproduced identical CO_2_ adsorption at 303 K and ≈1 bar (Figure S41), confirming this as an intrinsic property of the sorbent.

We also evaluated moisture stability. **TIFSIX‐enmepy‐Zn** exhibits a Type‐I H_2_O vapour adsorption isotherm, reaching ≈30 weight% (wt%) at 90% relative humidity (R.H.), but shows incomplete desorption and pronounced hysteresis, indicating that framework–water interactions hinder complete regeneration (Figure S42). Regeneration could not be achieved through humidity swing alone (Figure S43), and heating at 60°C under temperature‐swing conditions resulted in complete desorption only after prolonged exposure (≈581 min), highlighting the energy‐intensive nature of water removal in this material (Figure S44). By contrast, **NbOFFIVE‐enmepy‐Zn** exhibits a stepped adsorption isotherm and complete desorption with minimal hysteresis (Figure S45), reflecting weaker water‐framework interactions in **NbOFFIVE‐enmepy‐Zn** vs. that in **TIFSIX‐enmepy‐Zn**. Aligned with this absence of hysteresis, the humidity swing itself could fully regenerate **NbOFFIVE‐enmepy‐Zn** at ambient temperature within 19 min (Figure S46). This stark disparity (581 min at 60°C for **TIFSIX‐enmepy‐Zn** vs. only 19 min at 27°C for **NbOFFIVE‐enmepy‐Zn**) underscores how pillar substitution can govern water sorption properties. The collective comparison between C_2_H_2_, CO_2,_ and H_2_O kinetics in **NbOFFIVE‐enmepy‐Zn** is presented in Figure S47. The superior humidity tolerance of **NbOFFIVE‐enmepy‐Zn** was found to be consistent with accelerated stability tests conducted by incubating activated samples at 35°C and 75% R.H for 7 days (Figures S48 and S49). Furthermore, chemical stability tests of **NbOFFIVE‐enmepy‐Zn** in solvents of varying polarity showed retention of the characteristic PXRD reflections, indicating stability of **NbOFFIVE‐enmepy‐Zn** (Figure S50). Notably, an ambient‐aged sample of **NbOFFIVE‐enmepy‐Zn** stored under laboratory conditions for one year also retained C_2_H_2_ uptake capacity over multiple adsorption–desorption cycles, demonstrating excellent long‐term stability (Figure S51).

### 2.3 Dynamic Column Breakthrough Experiments

**FIGURE 4:**
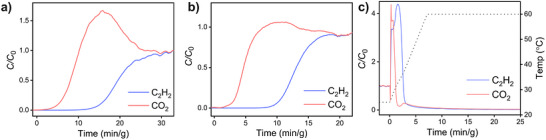
Binary C_2_H_2_/CO_2_ dynamic column breakthrough experiments on a **NbOFFIVE‐enmepy‐Zn** fixed‐bed: mass spectrometry signals showing the separation of C_2_H_2_ and CO_2_ from equimolar (a) dry and (b) wet mixtures; (c) temperature‐programmed desorption traces for C_2_H_2_ and CO_2_ after equilibration in a dry equimolar mixture of C_2_H_2_ and CO_2_.

The C_2_H_2_/CO_2_ separation properties of **NbOFFIVE‐enmepy‐Zn** and **TIFSIX‐enmepy‐Zn** were evaluated by dynamic column breakthrough (DCB) experiments. Passing a dry equimolar mixture (1:1 (*v*/*v*)) of C_2_H_2_ and CO_2_ through a column packed with **NbOFFIVE‐enmepy‐Zn** at a combined flow rate of 1.28 cm^3^ min^−1^ (298 K; flowrate of 0.64 cm^3^ min^−1^ for each gas; 400 mg sample mass) resulted in a clear separation of the two gases, with CO_2_ beginning to elute at ca. 2 min g^−1^ and C_2_H_2_ beginning to elute with a more gradual slope at ca. 12 min g^−1^. Equilibrium mixed‐gas uptakes were calculated (from this data) at 12.96 cm^3^ g^−1^ for C_2_H_2_ and 1.31 cm^3^ g^−1^ for CO_2_, yielding a separation factor of 9.7 (Figure 4a). Under identical dry flow conditions, a packed bed of **TIFSIX‐enmepy‐Zn** exhibited lesser separation performance with elution times of ca. 5 and ca. 2 min g^−1^, and mixed‐gas uptakes of 5.07and 3.94 cm^3^ g^−1^ for C_2_H_2_ and CO_2_, respectively, yielding a separation factor of 1.3 (Figure S52). Under wet conditions (60% R.H.) and identical gas flow rates, **NbOFFIVE‐enmepy‐Zn** was found to partially retain its separation performance, with elution times of ca. 2 and ca. 8 min g^−1^ for CO_2_ and C_2_H_2_, respectively. These correspond to uptakes of 8.77 and 1.83 cm^3^ g^−1^ of C_2_H_2_ and CO_2_, respectively. The separation factor under humid conditions was determined to be 4.8, a reduction of ca. 50% vs. the separation performance under dry conditions (Figure 4b). After completing the adsorption branches during the dry and wet DCB measurements for **NbOFFIVE‐enmepy‐Zn**, temperature‐programmed desorption (TPD) studies were performed by switching the inlet gas mixture to a helium flow of 20 cm^3^ g^−1^ and applying a controlled heating ramp of 5 K min^−1^ from 25°C up to 60°C. Desorption was monitored until negligible further release of the sorbate gases was observed. A final temperature of 60°C proved adequate for regenerating **NbOFFIVE‐enmepy‐Zn** after both dry and wet DCB experiments. Further, the TPD plots (Figure 4c; Figure S53) confirm that regenerating **NbOFFIVE‐enmepy‐Zn** under both conditions was facile and could be accomplished in less than 20 min g^−1^. Acetylene was recovered at technical‐grade purity (> 90%) from 1.28 to 2.32 min g^−1^ and > 80% from 1.19 to 2.58 min g^−1^, with a maximum purity of 96.5% (Figure 4). TPD recorded after adsorption of a wet gas mixture pointed at a slight reduction in maximum C_2_H_2_ purity (93.6%, Figure S53).

### 2.4 In Situ Infrared Spectroscopy

**FIGURE 5:**
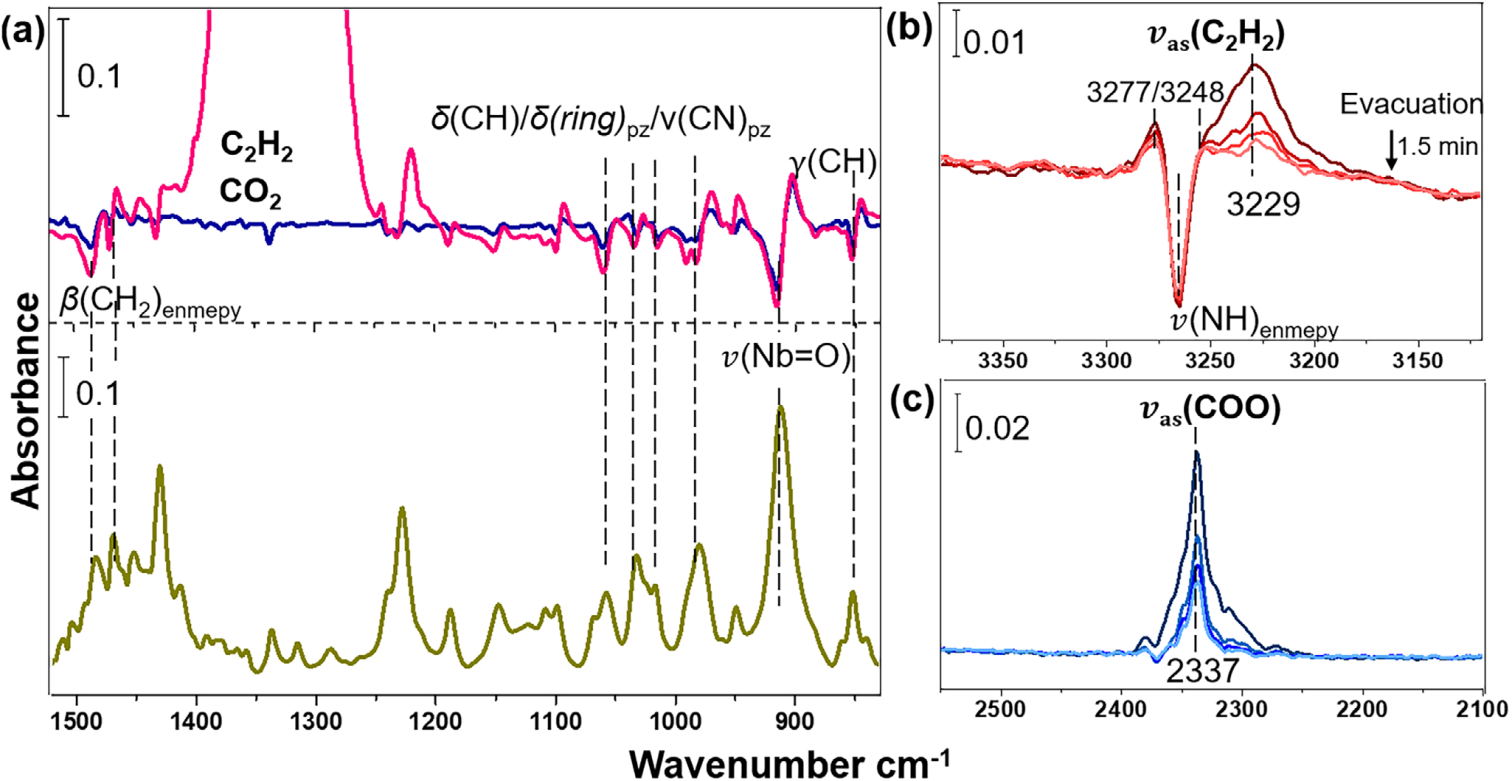
(a) In situ IR difference spectra for CO_2_ (blue) and C_2_H_2_ (pink)‐loaded **NbOFFIVE‐enmepy‐Zn**; each spectrum is referenced to the spectrum for activated **NbOFFIVE‐enmepy‐Zn** (bottom brown line). Each gas was loaded at ≈1 bar and 24°C. (b,c): the decay of ʋ_as_(C_2_H_2_) and ʋ_as_(CO_2_) bands during evacuation for ≈1.5 min. (ʋ, stretching; β, bending, δ, in plane deformation; γ, out of plane deformation; as, asymmetric; pz, pyridine).

To understand the interactions of C_2_H_2_ and CO_2_ with **NbOFFIVE‐enmepy‐Zn**, we further conducted *in‐situ* infrared (IR) spectroscopy measurements of loading C_2_H_2_ and CO_2_ into the activated structure. The difference spectra in Figure 5a show evident perturbations of **NbOFFIVE‐enmepy‐Zn** IR bands in the region 1500–850 cm^−1^ arising from the loading of C_2_H_2_ and CO_2_. The observed C_2_H_2_‐selective response is particularly associated with NbOF_5_^2−^ and enmepy (Figure S54), as typified by the derivative feature. The most strongly perturbed band is ʋ(Nb═O) at 912 cm^−1^ [41], which suggests C_2_H_2_ and CO_2_ interacting with the NbOF_5_ pillar. The pyridine bands, such as coupled δ(CH)/ ʋ(CN)/δ(ring), and localized *γ*(CH) modes [15, 42], are also appreciably affected.

As identified by our former works [43, 44, 45], these bands are sensitive toward the occupation of guest sorbate molecules in their proximity. Thus, we infer that C_2_H_2_ and CO_2_ molecules also interact with the pyridine moiety of enmepy. In addition, the β(CH_2_) band of the organic linker is also perturbed. The greater perturbations caused by C_2_H_2_ inclusion in these bands indicate stronger interactions of C_2_H_2_ with **NbOFFIVE‐enmepy‐Zn**. To probe the adsorbed C_2_H_2_ and CO_2_ molecules, we pumped out the gas phase that dominates the spectral signals (Figure S55). Both C_2_H_2_ and CO_2_ are characterized by their asymmetric stretching (ʋ_as_) absorption (Figure 5b,c). For C_2_H_2_, the spectral line of the ʋ_as_ band is complicated by the perturbed ʋ(N‐H) band occurring in the same region. As a result, only the band located at 3229 cm^−1^ can be clearly identified. This band is shifted further by 58 cm^−1^ with respect to C_2_H_2_ gas phase value (at 3287 cm^−1^) [15], indicating the presence of hydrogen bonding [15, 37, 46]. Careful examination of ʋ_as_(C_2_H_2_) spectra reveals that a higher wavenumber absorption band closer to the gas phase value is also present, indicated by the gain features at 3277 and 3248 cm^−1^. The observations on ʋ_as_(C_2_H_2_) spectra point to the presence of multiple C_2_H_2_ species and contrast with the adsorbed CO_2_. The latter displays a single peak at 2337 cm^−1^, thus indicating a single type of adsorbed CO_2_ within **NbOFFIVE‐enmepy‐Zn**.

### 2.5 Computational Studies

**FIGURE 6:**
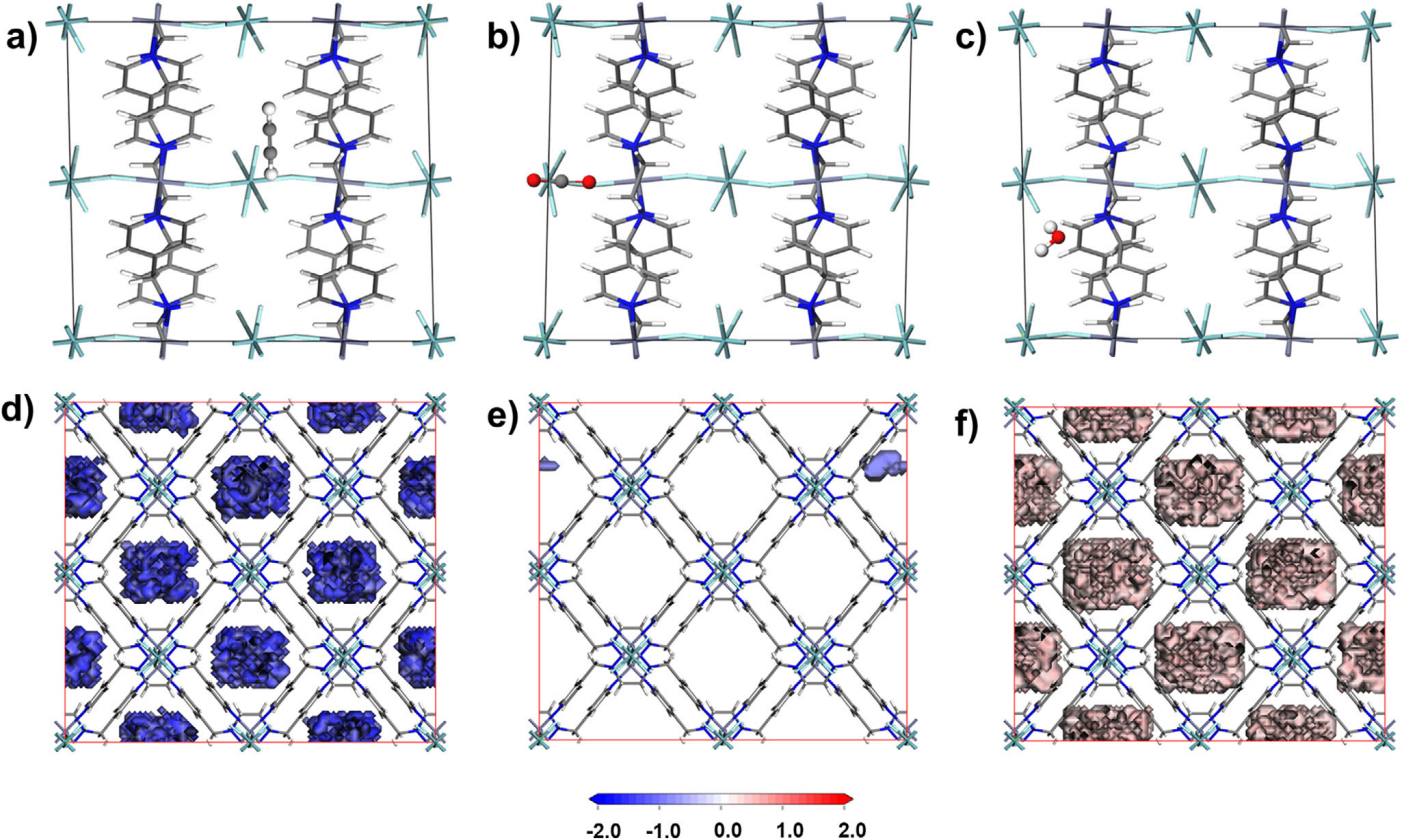
Sorbate binding sites: (a–c) show **NbOFFIVE‐enmepy‐Zn** loaded with C_2_H_2_, CO_2_, and H_2_O, respectively, using the lowest‐energy loaded structures obtained from CMC calculations. (d–f) illustrate combined visualisations of C_2_H_2_, CO_2_, and H_2_O energy and density distributions. The isosurfaces represent constant density (isovalue = 0.0003 #/Å^3^) and are coloured by potential energy. The colour map values indicate the adsorbate potential energy in kcal/mol.

Computational studies were undertaken to rationalise the C_2_H_2_/CO_2_ separation properties (Figure 6; Figures S56–S60, Tables S9 and S10) of **NbOFFIVE‐enmepy‐Zn** and **TIFSIX‐enmepy‐Zn**. Figure 6a–c shows the lowest‐energy single‐adsorbate configurations (one molecule per supercell) of C_2_H_2_, CO_2_, and H_2_O in **NbOFFIVE‐enmepy‐Zn**, as located by the Canonical Monte Carlo calculations, making the primary adsorption sites explicit. C_2_H_2_ and CO_2_ bind adjacent to the NbOF_5_^2−^ pillar (Figure 6a,b), whereas C_2_H_2_ further makes short contact interactions with the aromatic rings of the enmepy ligand via C─H─π interactions, consistent with our in situ IR signatures (Figure 5a). Grand Canonical Monte Carlo (GCMC) simulations corroborated the experimental isotherms (Figure S58), confirming that C_2_H_2_ has a higher uptake capacity than CO_2_ in **NbOFFIVE‐enmepy‐Zn**, consistent with the former's stronger binding at well‐defined sites in the framework (Figure 6a,b). Full computational details are provided in the Molecular Modelling section of the Supporting Information.

To understand the effect of water and the relative binding strengths of C_2_H_2_ and CO_2_, we analysed combined energy–density maps (isosurfaces at constant density, isovalue = 0.0003 #/Å^3^, color‐coded by potential energy in kcal mol^−1^; Figure 6d–f). In these maps, blue denotes lower (more negative) potential energy and red denotes higher (less negative) values. C_2_H_2_ exhibits a darker blue isosurface than CO_2_, which indicates lower potential energies, stronger binding, and consequently higher uptake. Periodic density functional theory calculations were performed to quantify these interactions, yielding binding energies of approximately −39.5 and −35.9 kJ mol^−^^1^ for C_2_H_2_ and CO_2_ respectively in **NbOFFIVE‐enmepy‐Zn**. While the computed values in stable binding pockets are higher in magnitude than the experimental *Q_st_* values (−31.2 kJ mol^−1^ (C_2_H_2_); −21.2 kJ mol^−^^1^ (CO_2_)), the relative trend is consistent. By contrast, **TIFSIX‐enmepy‐Zn**, with its symmetric and fluorine‐rich TiF_6_^2−^ pillar, strengthens CO_2_–framework interactions, resulting in higher CO_2_ uptake and comparatively lower C_2_H_2_/CO_2_ selectivity, consistent with the computed energy–density maps and binding energies (Figure S57e and Figure 6e). H_2_O isosurface is predominantly red in **NbOFFIVE‐enmepy‐Zn** (Figure 6f), consistent with weak, non‐specific interactions with the framework (Table S9). This energy–density landscape is well‐reflected in the mean square displacement (MSD) analysis, which reveals a pronounced loading‐dependent diffusion mechanism for C_2_H_2_. At low pressures, C_2_H_2_ molecules are preferentially localised at high‐affinity adsorption sites, resulting in restricted mobility and low MSD values. As pressure increases and these sites become progressively occupied, additional C_2_H_2_ molecules populate less strongly binding regions of the pore, leading to a marked increase in MSD and enhanced molecular mobility (Figure S59a). In contrast, CO_2_ exhibits a smooth and monotonic MSD profile across the pressure range studied, indicating weaker host–guest interactions (Figure S59b). H_2_O displays the highest MSD values and remains highly mobile even at low pressures (Figure S59c), indicative of weak, non‐specific interactions with the framework. These computational trends closely mirror the experimental kinetics, wherein H_2_O exhibits the fastest adsorption dynamics, CO_2_ exhibits limited uptake, and C_2_H_2_ displays a distinct loading‐dependent uptake behaviour (Figure S47), underscoring the consistency between experimental and computational findings. Complementary short‐contact analysis of the optimized guest‐loaded structures further confirms that H_2_O does not form close contacts with the framework, consistent with its weak and non‐specific interactions, and easier regeneration (Figures S46 and S60). Altogether, the energy–density portraits rationalise several aspects collectively: the adsorption enthalpies, the IAST‐derived adsorption selectivities (Figure 2d,g), and the observed kinetics (Figure S47), while elucidating why competing water molecules have a minimal adverse impact on C_2_H_2_/CO_2_ separation (Figure 4a,b) under the conditions studied. Collectively, we observe how minimal preference of CO_2_ (both in experiments and modelling) and weak interactions with H_2_O guide the lead sorbent herein, **NbOFFIVE‐enmepy‐Zn**, to achieve energy‐efficient C_2_H_2_/CO_2_ separation.

## 3 Conclusion

Expanding along the prototypal chelating HUMs platform through systematic pillar substitution enabled us to fine‐tune their properties, especially regeneration energy and stability. Whereas SnF_6_^2–^ produced an unstable framework, **SNIFSIX‐enmepy‐Zn**, incorporation of TiF_6_^2–^ and NbOF_5_^2–^ yielded more robust analogues. Among these, **NbOFFIVE‐enmepy‐Zn** stands out by optimally combining higher C_2_H_2_ uptake with lower CO_2_ affinity than other chelating ligand‐derived HUMs, including **TIFSIX‐enmepy‐Zn** as introduced herein. Gravimetric and volumetric adsorption‐desorption studies revealed that **NbOFFIVE‐enmepy‐Zn** afforded high selectivity combined with facile regeneration and high moisture tolerance. DCB experiments confirmed that **NbOFFIVE‐enmepy‐Zn** offers dynamic C_2_H_2_/CO_2_ separation performance of an order akin to the benchmark C_2_H_2_‐selective sorbents while offering rapid regeneration [47]. TPD experiments demonstrate technical‐grade C_2_H_2_ delivery (>90% purity from 1.28 to 2.32 mmol g^−^^1^, >80% from 1.19 to 2.58 mmol g^−^^1^) even from 1:1 (*v*/*v*) C_2_H_2_/CO_2_ mixtures, with performance largely retained under humid conditions. *In‐situ* IR spectroscopy reveals multiple adsorbed C_2_H_2_ species, implying the presence of more than one host–guest interaction for C_2_H_2_, whereas CO_2_ exhibits a single interaction mode, consistent with the preferential adsorption behaviour of **NbOFFIVE‐enmepy‐Zn**. Complementary computational studies provide insight into the binding sites of both gases, supporting affinity for C_2_H_2_ over CO_2_ and explaining the effect of water. Despite its strong kinetic preference, water interacts only weakly with **NbOFFIVE‐enmepy‐Zn**, therefore, affecting its C_2_H_2_ over CO_2_ affinity minimally, even more so when seen in comparison to **TIFSIX‐enmepy‐Zn**.

In summary, anion‐modulated chelating HUMs were found to overcome the trade‐offs often encountered in C_2_H_2_/CO_2_ separation by delivering high uptake capacity difference (Δq, C_2_H_2_ vs. CO_2_) (>1.5 mmol g^−1^), strong selectivity (*S*_AC_ > 5), easy regenerability (25 ≤ *Q*_st_(C_2_H_2_) ≤ 50; in kJ mol^−1^), and prolonged stability (>7 days) under humid conditions. **NbOFFIVE‐enmepy‐Zn** exemplifies this balance, combining higher C_2_H_2_ affinity with low regeneration energy penalty. Beyond this, the results also highlight chelating polytopic ligands as an underexplored platform to control the resulting HUM structures, leaning into their separation properties thereof.

## Funding

Research Ireland Pathway grant 21/PATH‐S/9454; SSPC (the Research Ireland Centre for Pharmaceuticals) grant 12/RC/2275_P2; European Research Council grant ADG 885695; Research Ireland grant 21/US/3760; Research Ireland grant 23/FFP‐A/12221; U. S. NSF grant CBET‐ 2530186.

## Conflicts of Interest

The authors declare no conflicts of interest.

## Supporting information




**Supporting File 1**: smll74171‐sup‐0001‐SuppMat.pdf.

**Supporting File 2**: smll74171‐sup‐0002‐Data.zip.

## Data Availability

The data that support the findings of this study are available from the corresponding author upon reasonable request.
