# Supplementary material for: Crystal Engineering of Chelating Hybrid Ultramicroporous Materials via Pillar Modulation for Energy‐Efficient Acetylene Separation
Source: Small. 2026 Jun 12;22(43):e74171. doi: 10.1002/smll.74171 (PMC13432632; doi:10.1002/smll.74171)
Supplement: Supplementary file 1 — Supporting File 1: smll74171‐sup‐0001‐SuppMat.pdf. [file SMLL-22-e74171-s001.pdf]

Supporting Information to

Crystal Engineering of Chelating Hybrid Ultramicroporous Materials via Pillar Modulation for Energy-efficient Acetylene Separation

Asif Raza,[a] Julia Korenko,[a] Sousa Javan Nikkhah,[a,b] Sayan Maiti, [c] Debobroto Sensharma,[a] Lilia Croitor, [a] Kui Tan, [d] Matthias Vandichel,[a] Michael J. Zaworotko,*[a] and Soumya Mukherjee*[a]

[a] Bernal Institute and Research Ireland Centre for Pharmaceuticals (SSPC), Department of Chemical Sciences, University of Limerick, Limerick, V94 T9PX Ireland. E-mails: Michael.Zaworotko@ul.ie; Soumya.Mukherjee@ul.ie

[b] Department of Chemistry, Kathleen Lonsdale Institute, Maynooth University, Maynooth W23 F2H6, Kildare, Ireland.

[c] Department of Chemistry, University of North Texas, Denton, Texas 76203, United States.

[d] Department of Chemistry and Biochemistry, The University of Texas at El Paso, El Paso, Texas 79968, United States.

Correspondence E-mails: Michael.Zaworotko@ul.ie; Soumya.Mukherjee@ul.ie

Contents

Methods 3

Materials and general experimental procedures 3

Synthesis of $ZnMF_6 \cdot 6H_2O$ (M = Si, Ti, Sn) and $ZnNbOF_5 \cdot 6H_2O$ salts 3

Synthesis of *N*[1],*N*[2]-bis(pyridine-4-ylmethyl)ethane-1,2-diamine (enmepy) 3

Synthesis of single crystals of HUMs 3

Synthesis of bulk powder of HUMs 3

CSD survey 4

Single Crystal Structural Information 48

Scanning Electron Microscopy (SEM) 54

Particle size analysis 55

Powder X-ray diffraction (PXRD) 57

Thermogravimetric analysis (TGA) 60

Variable-Temperature Powder X-ray Diffraction (VT PXRD) 62

Single-component gas sorption experiments 65

Adsorption selectivity calculations. 70

Adsorption energy calculations. 72

Gravimetric kinetic adsorption of gases 77

Water vapour sorption 80

Stability tests 86

Dynamic column breakthrough experiments 88

Comparison of gas sorption and separation performance 90

***In-situ* Infrared (IR) spectroscopy** 94

Molecular Modelling 95

References 105

Methods

Materials and general experimental procedures

All chemicals were used as received. Isonicotinaldehyde (99%) was procured from Fluorochem; Sodium borohydride ($NaBH_4$) from TCI; and ethylenediamine (>98%) from Sigma-Aldrich. HPLC-grade solvents: methanol (MeOH), dichloromethane (DCM), and diethyl ether (Et_2O) were purchased from Sigma-Aldrich. Research-grade gases—CO_2 (99.999%), N_2 (99.9992%), He (99.996%), and C_2H_2 (99.9995%)—were obtained from BOC Gases Ireland and were used without further purification.

Synthesis of $ZnMF_6·6H_2O$ (M = Si, Ti, Sn) and $ZnNbOF_5·6H_2O$ salts

$ZnMF_6·6H_2O$ ((M = Si, Ti, Sn) was synthesized as per the previously reported procedure.[1–2] And $ZnNbOF_5.6H_2O$ was synthesized as per the reported procedure.[3]

Synthesis of N^1,N^2-bis(pyridine-4-ylmethyl)ethane-1,2-diamine (enmepy)

The ligand enmepy was prepared following a modified literature procedure.[4] Ethylenediamine (1.0 equivalent (eq.), 0.53 mL) in MeOH (30 mL) was added dropwise via a dropping funnel with equilibration arm to a solution of isonicotinaldehyde (2.0 eq., 1.41 mL) in MeOH (30 mL) in a round-bottom flask. The mixture was refluxed under N_2 for 3 h, cooled to room temperature, and $NaBH_4$ (10 eq.) was added in small portions. The reaction mixture was stirred overnight. Deionised water (30 mL) was then added, and stirring was continued for 24 h. MeOH was removed under reduced pressure, and the residue was extracted with DCM (4 × 25 mL), affording a yellow oil. This was dissolved in DCM (3 mL) and diethyl ether (30 mL), and the flask was scratched in an ice bath until a fine white precipitate formed. The solid was collected by filtration and dried under vacuum at room temperature overnight. The product was characterised by ^1H NMR, in good agreement with literature data.

Synthesis of single crystals of HUMs

In an NMR tube, 400 µL of a MeOH solution of enmepy (2.91 mg, 0.03 mmol mL^{-1}) was carefully layered over 400 µL of an aqueous solution of salt ($ZnSiF_6·6H_2O$ (3.79 mg), $ZnTiF_6·6H_2O$ (4.02 mg), $ZnSnF_6·6H_2O$ (4.87 mg), and $ZnNbOF_5·6H_2O$ (4.53 mg)). A 200 µL MeOH/H_2O (1:1 v/v) mixture was introduced as a buffer layer. Crystals suitable for single-crystal X-ray diffraction (SCXRD) were obtained after 2–3 weeks.

Synthesis of bulk powder of HUMs

0.4 mmol of salts ($ZnSiF_6·6H_2O$ (126.2 mg), $ZnTiF_6·6H_2O$ (134.1 mg), $ZnSnF_6·6H_2O$ (162.4 mg), and $ZnNbOF_5·6H_2O$ (150.9 mg)) was dissolved in deionised water, and a solution of enmepy (96 mg, 0.4 mmol) in MeOH (5 mL) was added separately to each salt solution, yielding white precipitate. The

mixture was stirred overnight at room temperature, then filtered through a 0.4 μm membrane, washed with MeOH, and air-dried.

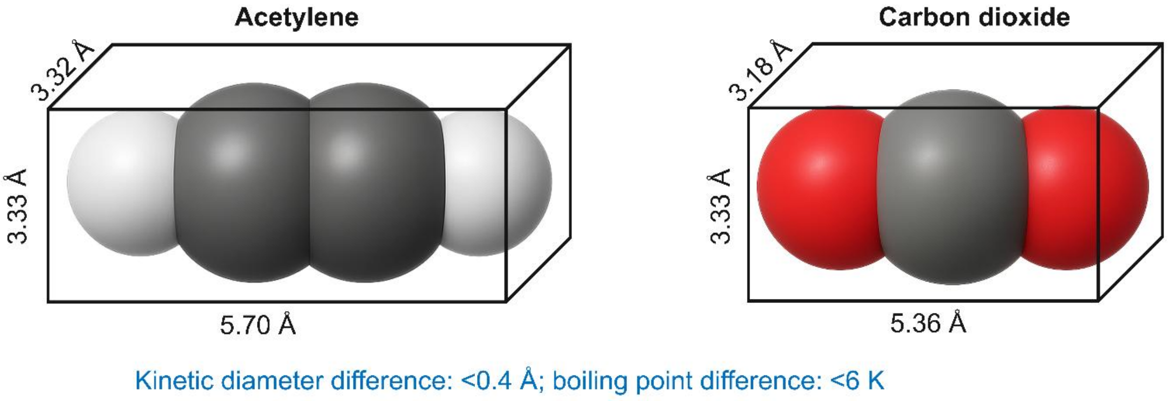

Scheme S1: Comparison of the molecular structures and physical properties of C_2H_2 and CO_2.

CSD survey

The Cambridge Structural database (CSD version 6.0, August 2025) was searched for hybrid coordination networks (HCNs), *i.e.*, structures containing anion pillars (fluorinated anions or tetraoxo anions) and *N*-donor organic linker ligands (Figures S1 and S2). The search afforded 1163, 598, and 1492 hits respectively, whereas manual inspection resulted in a total of 455 hits. Each of the latter subset of structures was found pillared by anions of interest, with 83 duplicates (mentioned in parenthesis in the Refcode column). The results are grouped by *N*-donor linker ligands (IUPAC names), columns report inorganic anion, metal centre, and denticity of the ligand.

a)

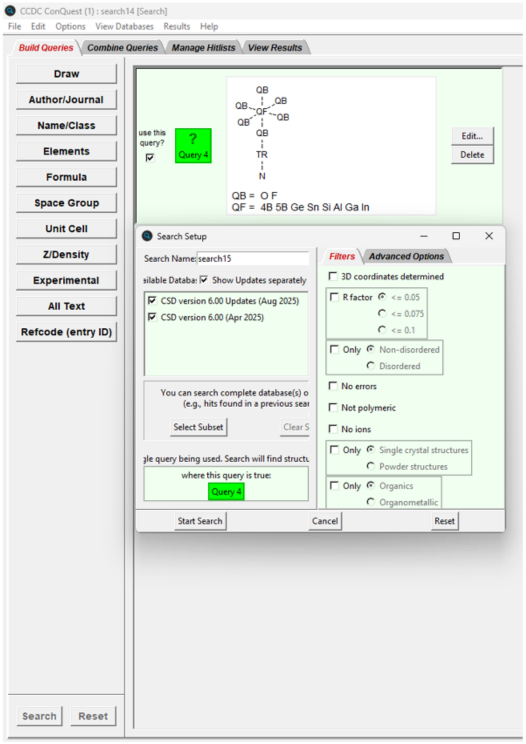

b)

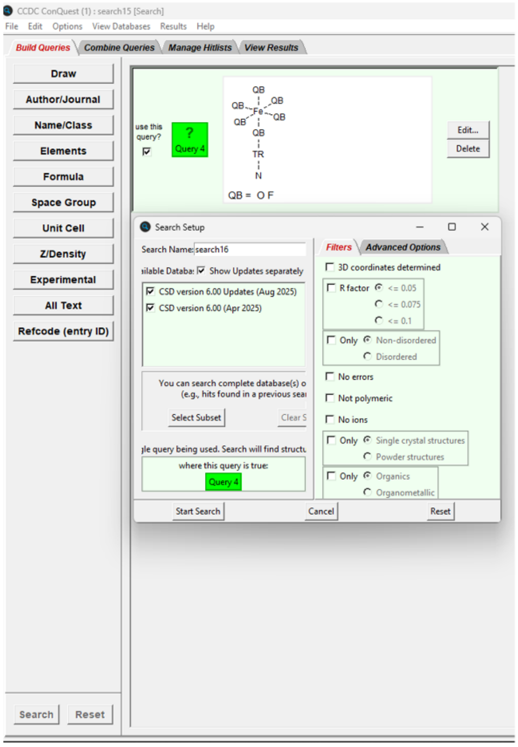

Figure S1: ConQuest queries used to search the CSD database; a) for hexafluoro anions (except Fe); b) fluorinated Fe anion.

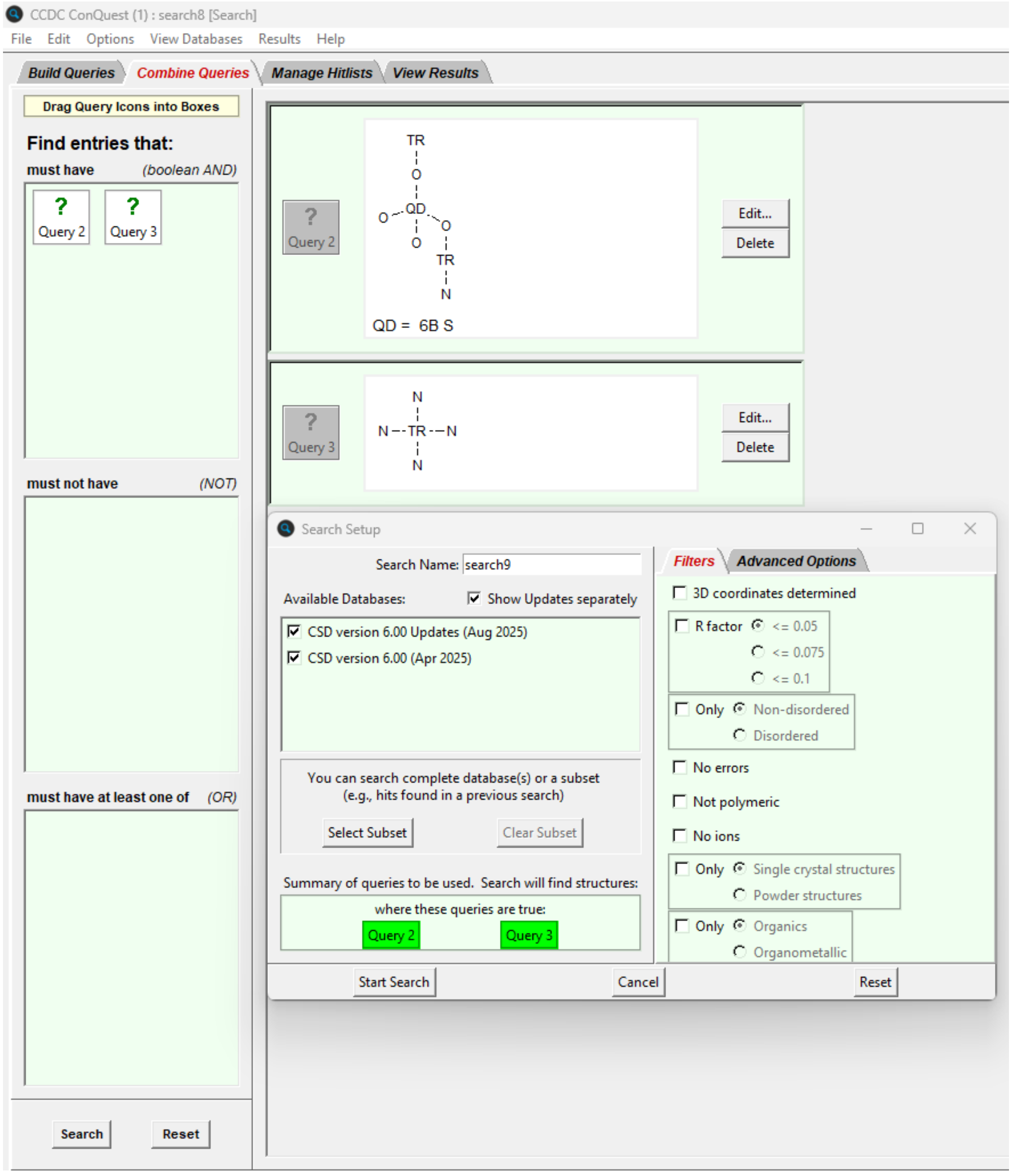

Figure S2: ConQuest queries used to search the CSD database for oxoanions.

Table S1: Complete list of CSD entries used in this study, grouped by the organic ligands (IUPAC name below the structure drawing). For each structure, the CSD Refcode, inorganic anion, and metal centre is provided in columns. The blue highlighted rows (numbers 90 to 95) denote the chelating ligands-derived HCNs, including chelating HUMs.

No.	Organic ligand	Inorganic ligand	Metal	Guest/solvent	Topicity of ligand	Dimensionality of framework	Refcode	Reference
1	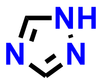 1,2,4-triazole	SiF_6^{2-}	Zn^{2+}	-	Tritopic	3D	**KEVFUO**	[5]
2	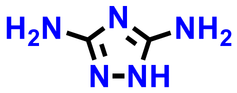 3,5-Diamino-1,2,4-triazole	SO_4^{2-}	Cd^{2+}	Water	Ditopic	3D	**NENLIC**	[6]
3	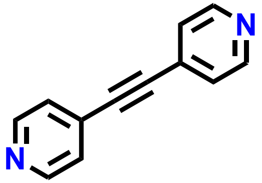 1,2-bis(pyridin-4-yl)ethyne	TiF_6^{2-}	Cu^{2+}	Methanol	Ditopic	3D	**INEGEP**	[7]
		$NbOF_5^{2-}$	Ni^{2+}	-	Ditopic	3D	**DOJJIY**	[8]
		$NbOF_5^{2-}$	Cu^{2+}	-	Ditopic	3D	**XIMMAK (01)**	[9]
		SiF_6^{2-}	Zn^{2+}	Chloroform	Ditopic	3D	**WONZOP**	[10]
		SiF_6^{2-}	Cu^{2+}	-	Ditopic	3D	**YEMTER**	[11]
		SiF_6^{2-}	Cu^{2+}	Methanol	Ditopic	3D	**YEMTIV**	[11]
		SiF_6^{2-}	Cu^{2+}	Deuterated acetylene (C_2D_2)	Ditopic	3D	**EMEHUB**	[12]

		NbF_6^-	Cu^{2+}	F^-, methanol	Ditopic	3D	**DENWAX**	[13]
		NbF_6^-	Cu^{2+}	F^-,	Ditopic	3D	**PEGKIY**	[13]
		GeF_6^{2-}	Cu^{2+}	-	Ditopic	3D	**PEGKUK**	[13]
		SiF_6^{2-}	Cu^{2+}	Sulphur dioxide	Ditopic	3D	**PESVIV**	[14]
		GeF_6^{2-}	Cu^{2+}	Methanol	Ditopic	3D	**TEYBEH**	[13]
		TiF_6^{2-}	Ni^{2+}	-	Ditopic	3D	**LOZHEQ**	[15]
		ZrF_6^{2-}	Cu^{2+}	-	Ditopic	3D	**OQOFUY**	[15]
		SnF_6^{2-}	Cu^{2+}	-	Ditopic	3D	**MOKRUD**	[16]
		SnF_6^{2-}	Cu^{2+}	-	Ditopic	3D	**MOKSAK**	[16]
		SnF_6^{2-}	Cu^{2+}	*n*-Hexane	Ditopic	3D	**MOKSEO**	[16]
		SnF_6^{2-}	Cu^{2+}		Ditopic	3D	**MOKSIS**	[16]
		$Cr_2O_7^{2-}$	Ni^{2+}	-	Ditopic	3D	**PUSJUK**	[17]
4	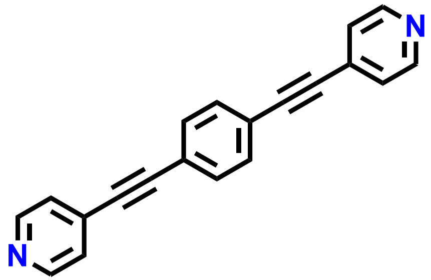 1,4-bis(pyridin-4-ylethynyl)benzene	SiF_6^{2-}	Zn^{2+}	Methanol	Ditopic	3D	**NATHIC**	[18]
		SiF_6^{2-}	Cu^{2+}	-	Ditopic	3D	**NATKAX**	[18]

5	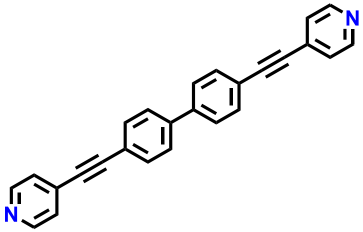 4,4'-bis(pyridin-4-ylethynyl)-1,1'-biphenyl	SiF_6^{2-}	Zn^{2+}	Water	Ditopic	3D	**NATHOI**	[18]
6	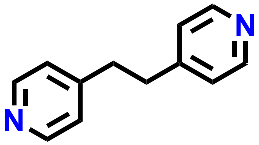 4,4'-(ethane-1,2-diyl)dipyridine	SiF_6^{2-}	Zn^{2+}	-	Ditopic	2D	**RAPMII**	[19]
		SiF_6^{2-}	Zn^{2+}	Methanol	Ditopic	2D	**RAPMUU**	[19]
		SiF_6^{2-}	Zn^{2+}	Ethyne	Ditopic	2D	**RAPPIL**	[19]
		SiF_6^{2-}	Zn^{2+}	-	Ditopic	3D	**RAPPOR**	[19]
		SiF_6^{2-}	Zn^{2+}	Ethyne	Ditopic	2D	**RAPPUX**	[19]
		$NbOF_5^{2-}$	Cu^{2}	-	Ditopic	2D	**KIPBUK (01-03)**	[20]
		$NbOF_5^{2-}$	Cu^{2}	Water	Ditopic	2D	**KIPJOM**	[20]
		$NbOF_5^{2-}$	Cu^{2}	-	Ditopic	3D	**KIPMAB**	[20]
		$NbOF_5^{2-}$	Cu^{2}	Methanol	Ditopic	2D	**KIPMEF**	[20]
		SO_4^{2-}	Co^{2+}	-	Ditopic	3D	**EJAXIW**	[21]
		SO_4^{2-}	Cu^{2+}	Water	Ditopic	3D	**XASFAY**	[22]
7		SiF_6^{2-}	Zn^{2+}	Methanol + Water	Ditopic	3D	**GELJEN**	[23]

	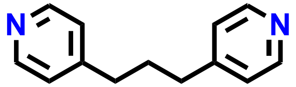 1,3-bis(pyridin-4-yl)propane	TiF_6^{2-}	Zn^{2+}	-	Ditopic	3D	**TEQGOP**	[24]
		SO_4^{2-}	Fe^{2+}	-	Ditopic	3D	**EJAWUH**	[21]
		MoO_4^{2-}	NI^{2+}	Water	Ditopic	3D	**FAKZUP**	[25]
		CrO_4^{2-}	Co^{2+}	Water	Ditopic	3D	**VOZDUM**	[26]
		$Cr_2O_7^{2-}$	Co^{2+}	-	Ditopic	3D	**VOZFAU**	[26]
		SO_4^{2-}	Cu^{2+}	Ethylene glycol	Ditopic	3D	**WISVID**	[27]
8	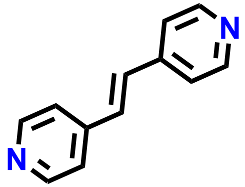 (*E*)-1,2-di(pyridin-4-yl)ethene	SiF_6^{2-}	Cu^{2+}	-	Ditopic	3D	**HAPKUG**	[28]
		ZrF_6^{2-}	Zn^{2+}	Methanol	Ditopic	3D	**WOHCII (01)**	[29]
		TiF_6^{2-}	Cu^{2+}	-	Ditopic	3D	**JEZLUZ**	[30]
		SO_4^{2-}	Cu^{2+}	Water	Ditopic	3D	**FAKQIR**	[31]
		WO_4^{2-}	Ni^{2+}	Acetonitrile	Ditopic	3D	**GIHPAQ**	[32]

		WO_4^{2-}	Co^{2+}	Water	Ditopic	3D	**GIHPEU**	[32]
		MoO_4^{2-}	Co^{2+}	Acetonitrile, Water	Ditopic	3D	**YEZKOF**	[33]
		MoO_4^{2-}	Ni^{2+}	Water	Ditopic	3D	**YEZKUL**	[33]
9	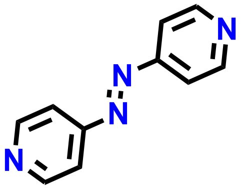 (*E*)-1,2-di(pyridin-4-yl)diazene	SiF_6^{2-}	Cu^{2+}	Methanol	Ditopic	3D	**XEBTAC**	[34]
		SiF_6^{2-}	Cu^{2+}	Deuterated acetylene (C_2D_2)	Ditopic	3D	**YEPTIZ**	[35]
		SiF_6^{2-}	Cu^{2+}	Deuterated acetylene (C_2D_2)	Ditopic	3D	**YEPTOF (01)**	[35]
		GeF_6^{2-}	Cu^{2+}	-	Ditopic	3D	**PEGKEU**	[36]
		TiF_6^{2-}	Cu^{2+}	-	Ditopic	3D	**HIQKUQ**	[37]
		SiF_6^{2-}	Cu^{2+}	Carbon dioxide	Ditopic	3D	**IPAKOC**	[38]
		SiF_6^{2-}	Cu^{2+}	-	Ditopic	3D	**WIBWEM**	[39]
		GeF_6^{2-}	Cu^{2+}	Acetylene	Ditopic	3D	**NAYHII (01)**	[40]

		$Cr_2O_7^{2-}$	Co^{2+}	-	Ditopic	3D	**SEPZOF**	[41]
		$Cr_2O_7^{2-}$	Cu^{2+}	-	Ditopic	3D	**SEPZUL**	[41]
		$Cr_2O_7^{2-}$	Ni^{2+}	-	Ditopic	3D	**SEQBAU**	[41]
		$Cr_2O_7^{2-}$	Zn^{2+}	-	Ditopic	3D	**SEQBEY**	[41]
10	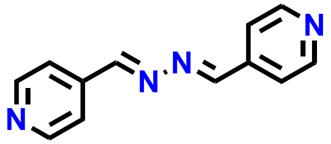	SiF_6^{2-}	Zn^{2+}	Dichloromethane	Ditopic	3D	**CEVGIW**	[42]
11	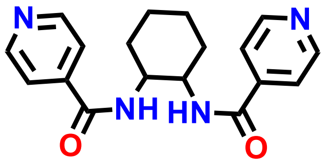 *N*,*N*'-(cyclohexane-1,2-diyl) diisonicotinamide	$Mo_2O_7^{2-}$	Ni^{2+}	-	Ditopic	3D	**TOHSOC (01)**	[43]
12	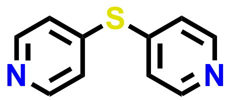 di(pyridin-4-yl)sulfane	SiF_6^{2-}	Zn^{2+}	Water	Ditopic	2D	**SAXPAL**	[44]
		SiF_6^{2-}	Zn^{2+}	Deuterated acetylene (C_2D_2)	Ditopic	2D	**SAXPEP**	[44]
		SiF_6^{2-}	Zn^{2+}	-	Ditopic	2D	**SAXPIT**	[44]
		SiF_6^{2-}	Cu^{2+}	Acetylene, water	Ditopic	2D	**MUBQAE (01)**	[45]
		GeF_6^{2-}	Cu^{2+}	-	Ditopic	2D	**NUQGEO (01)**	[46]

		GeF_6^{2-}	Zn^{2+}	Methanol	Ditopic	2D	**NUQGIS**	[46]
		GeF_6^{2-}	Zn^{2+}	-	Ditopic	2D	**NUQGUE**	[46]
		GeF_6^{2-}	Cu^{2+}	Acetylene	Ditopic	2D	**NUQHAL**	[46]
		GeF_6^{2-}	Cu^{2+}	Prop-1-yne	Ditopic	2D	**NUQHEP**	[46]
		$NbOF_5^{2-}$	Cu^{2}	-	Ditopic	2D	**QALNUQ**	[47]
		$NbOF_5^{2-}$	Cu^{2}	-	Ditopic	2D	**QALPAY**	[47]
		SiF_6^{2-}	Cu^{2+}	Water	Ditopic	2D	**RUJDIM**	[48]
		GeF_6^{2-}	Cu^{2+}	Water	Ditopic	2D	**FAWXOU**	[49]
		$NbOF_5^{2-}$	Cu^{2}	Water	Ditopic	2D	**FAWXUA**	[49]
		GeF_6^{2-}	Cu^{2+}	Water	Ditopic	2D	**CONRIK**	[50]
		GeF_6^{2-}	Cu^{2+}	Methanol	Ditopic	2D	**CONROQ**	[50]
		GeF_6^{2-}	Cu^{2+}	-	Ditopic	2D	**CONRUW**	[50]
		SO_4^{2-}	Cu^{2+}	Dimethylformamide	Ditopic	3D	**KETNON**	[51]
13	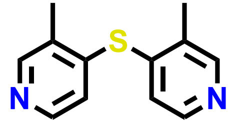 bis(3-methylpyridin-4-yl) sulfane	SO_4^{2-}	Cu^{2+}	Dichloromethane, water	Ditopic	2D	**ECOWOI**	[52]
14		$NbOF_5^{2-}$	Cu^{2}	-	Ditopic	2D	**QALNIE**	[53]

	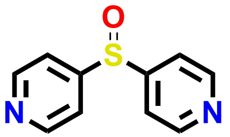<br>4,4'-sulfinyldipyridine	$NbOF_5^{2-}$	Cu^{2}	-	Ditopic	2D	**QALNOK**	[53]
15	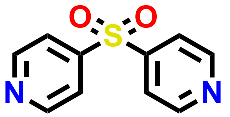<br>4,4'-sulfonyldipyridine<br>(overall 2D structures)	TiF_6^{2-}	Cu^{2+}	-	Ditopic	2D	**QALMUP**	[53]
		$NbOF_5^{2-}$	Cu^{2}	-	Ditopic	2D	**QALNAW**	[53]
		$NbOF_5^{2-}$	Cu^{2}	-	Ditopic	2D	**QALNEA**	[53]
		$NbOF_5^{2-}$	Cu^{2}	Acetylene	Ditopic	2D	**QALPEC**	[53]
		GeF_6^{2-}	Cu^{2+}	-	Ditopic	2D	**KITVES**	[54]
		GeF_6^{2-}	Cu^{2+}	-	Ditopic	2D	**KITVIW**	[54]
		GeF_6^{2-}	Cu^{2+}	Acetylene	Ditopic	2D	**KITVOC**	[54]
		SiF_6^{2-}	Cu^{2+}	-	Ditopic	2D	**TIPKIQ**	[55]
16	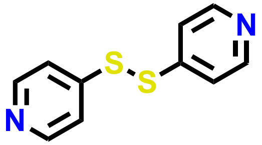<br>1,2-di(pyridin-4-yl)disulfane	SiF_6^{2-}	Zn^{2+}	Methanol	Ditopic	3D	**LEMMAS**	[56]
		SnF_6^{2-}	Cu^{2+}	-	Ditopic	2D	**COVLEI (01)**	[57]
		SnF_6^{2-}	Cu^{2+}	-	Ditopic	3D	**COVLIM (01)**	[57]
		WO_4^{2-}	Ni^{2+}	Acetylene	Ditopic	2D	**BIQPOK (01)**	[58]
		MoO_4^{2-}	Ni^{2+}	Acetylene	Ditopic	2D	**BIQPUQ (01)**	[58]
		CrO_4^{2-}	Ni^{2+}	Acetylene	Ditopic	2D	**BIQQAX (01)**	[58]

		SO_4^{2-}	Cu^{2+}	Methanol, water	Ditopic	2D	**EMADEB**	[59]
		CrO_4^{2-}	Co^{2+}	-	Ditopic	2D	**FIGVUQ**	[60]
		CrO_4^{2-}	Co^{2+}	-	Ditopic	2D	**FIGWAX**	[60]
		MoO_4^{2-}	Co^{2+}	-	Ditopic	2D	**FIGWEB**	[60]
		WO_4^{2-}	Co^{2+}	-	Ditopic	2D	**FIGWIF**	[60]
		CrO_4^{2-}	Co^{2+}	Acetylene	Ditopic	2D	**FIGWOL**	[60]
		MoO_4^{2-}	Co^{2+}	Acetylene	Ditopic	2D	**FIGWUR**	[60]
		WO_4^{2-}	Co^{2+}	-	Ditopic	2D	**FIGXAY**	[60]
		MoO_4^{2-}	Co^{2+}	-	Ditopic	2D	**FIGXEC**	[60]
		WO_4^{2-}	Co^{2+}	Acetylene	Ditopic	2D	**FIGXIG**	[60]
		CrO_4^{2-}	Ni^{2+}	-	Ditopic	2D	**YANZUM**	[61]
		MoO_4^{2-}	Ni^{2+}	-	Ditopic	2D	**YAPBAW**	[61]
		WO_4^{2-}	Ni^{2+}	-	Ditopic	2D	**YAPBEA**	[61]
		MoO_4^{2-}	Ni^{2+}	-	Ditopic	2D	**YAPBOK**	[61]
		CrO_4^{2-}	Ni^{2+}	-	Ditopic	2D	**YAPBUQ**	[61]
		WO_4^{2-}	Ni^{2+}	-	Ditopic	2D	**YAPCAX**	[61]
		CrO_4^{2-}	Ni^{2+}	Xenon	Ditopic	2D	**YAPCEB**	[61]
		MoO_4^{2-}	Ni^{2+}	Xenon	Ditopic	2D	**YAPCIF**	[61]

17	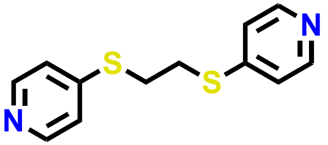 1,2-bis(pyridin-4-ylthio)ethane	SiF_6^{2-}	Zn^{2+}	Chloroform	Ditopic	2D	**HUKSEM**	[62]
18	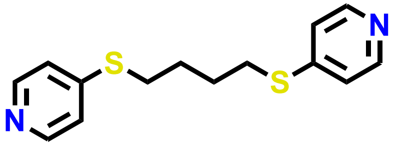 1,4-bis(pyridin-4-ylthio)butane	SiF_6^{2-}	Zn^{2+}	Chloroform, Ethanol	Ditopic	2D	**HUKSIQ**	[62]
19	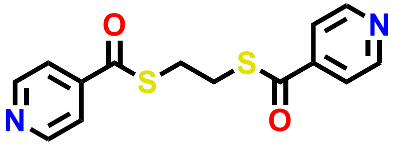 *S*,*S*'-(ethane-1,2-diyl) bis(pyridine-4-carbothioate)	SiF_6^{2-}	Zn^{2+}	Chloroform, Ethanol	Ditopic	2D	**HUKSOW**	[62]
20	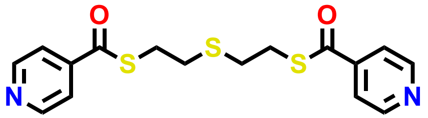 *S*,*S*'-(thiobis(ethane-2,1-diyl)) bis(pyridine-4-carbothioate)	SiF_6^{2-}	Zn^{2+}	Chloroform, Ethanol	Ditopic	2D	**HUKSUC**	[62]
21	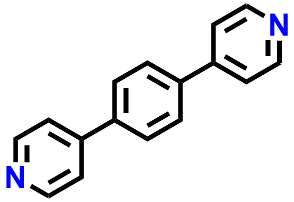 1,4-bis(pyridin-4-yl)benzene	SiF_6^{2-}	Zn^{2+}	Chloroform, water	Ditopic	3D	**WONZUV**	[63]
		TiF_6^{2-}	Cu^{2+}	-	Ditopic	3D	**RECZEH**	[64]
		$NbOF_5^{2-}$	Cu^{2+}	Methanol	Ditopic	3D	**FOKRAC**	[65]

		$Cr_2O_7^{2-}$	Ni^{2+}	-	Ditopic	3D	**XIKTAP**	[66]
22	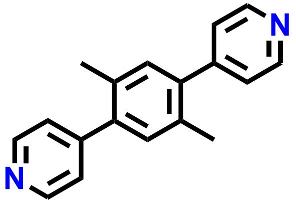<br>4,4'-(2,5-dimethyl-1,4-phenylene)dipyridine	$Cr_2O_7^{2-}$	Co^{2+}	Water	Ditopic	3D	**TAQGOK**	[67]
23	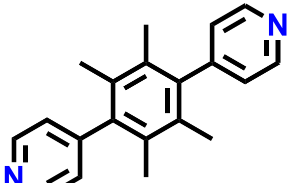<br>4,4'-(2,3,5,6-tetramethyl-1,4-phenylene)dipyridine	$Cr_2O_7^{2-}$	Co^{2+}	Acetonitrile	Ditopic	3D	**TAQGUQ**	[67]
24	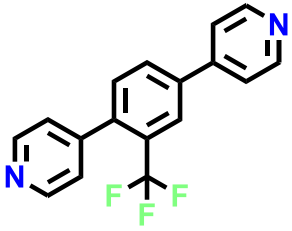<br>4,4'-(2-(trifluoromethyl)-1,4-phenylene)dipyridine	SiF_6^{2-}	Cu^{2+}	-	Ditopic	3D	**DICSOA**	[68]
25		TiF_6^{2-}	Cu^{2+}	Acetonitrile	Ditopic	3D	**DOCVAV**	[69]

	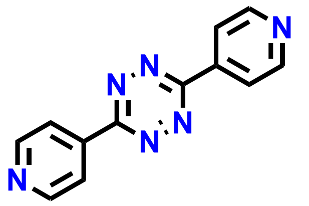 3,6-di(pyridin-4-yl)-1,2,4,5-tetrazine	SiF_6^{2-}	Cu^{2+}	Ethylene glycol	Ditopic	3D	**DOCVEZ**	[69]
		TiF_6^{2-}	Cu^{2+}	Carbon dioxide	Ditopic	3D	**DOCVID**	[69]
		SiF_6^{2-}	Cu^{2+}	Carbon dioxide	Ditopic	3D	**KOCDEO**	[70]
		SiF_6^{2-}	Cu^{2+}	-	Ditopic	3D	**KOCFAM**	[70]
26	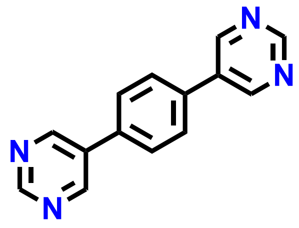 1,4-di(pyrimidin-5-yl)benzene	SiF_6^{2-}	Zn^{2+}	Dichloroethane	Ditopic	3D	**QANQAB**	[71]
27	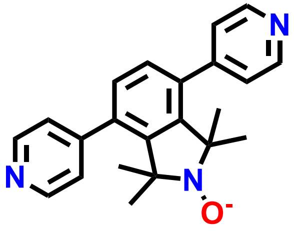 1,1,3,3-tetramethyl-4,7-di(pyridin-4-yl)isoindolin-2-olate	SiF_6^{2-}	Cu^{2+}	Water	Ditopic	3D	**VOLQAQ**	[72]

28	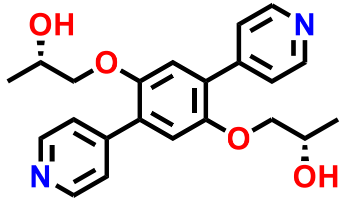 (2*S*,2'*S*)-1,1'-((2,5-di(pyridin-4-yl)-1,4-phenylene)bis(oxy))bis(propan-2-ol)	SiF_6^{2-}	Zn^{2+}	Chloroform	Ditopic	3D	**SERWAP**	[73]
29	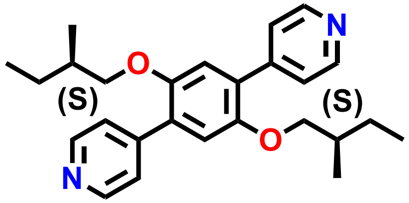 4,4'-(2,5-bis((*S*)-2-methylbutoxy)-1,4-phenylene)dipyridine	SiF_6^{2-}	Zn^{2+}	Chloroform	Ditopic	3D	**SERWET**	[73]
30	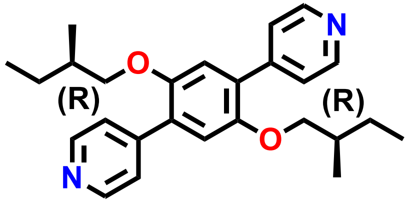 4,4'-(2,5-bis((*R*)-2-methylbutoxy)-1,4-phenylene)dipyridine	SiF_6^{2-}	Zn^{2+}	Chloroform	Ditopic	3D	**SERWIX**	[73]
31	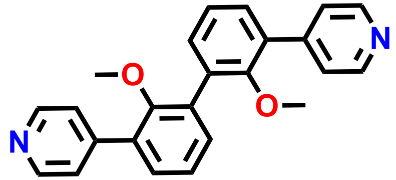 4,4'-(2,2'-dimethoxy-[1,1'-biphenyl]-3,3'-diyl)dipyridine	SiF_6^{2-}	Zn^{2+}	-	Ditopic	3D	**HANMAO**	[74]

32	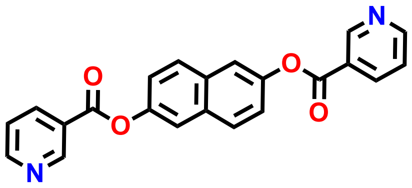 naphthalene-2,6-diyl dinicotinate	SiF_6^{2-}	Zn^{2+}	dichloromethane	Ditopic	3D	**QANTUY**	[75]
33	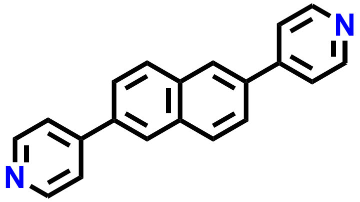 2,6-di(pyridin-4-yl)naphthalene	SiF_6^{2-}	Zn^{2+}	DMSO, Ethanol	Ditopic	3D	**LIFWOO**	[76]
34	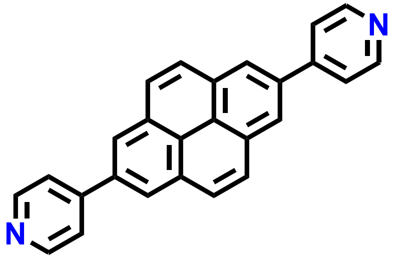 2,7-di(pyridin-4-yl)pyrene	SiF_6^{2-}	Zn^{2+}	Chloroform	Ditopic	3D	**CUHKAV**	[77]
35	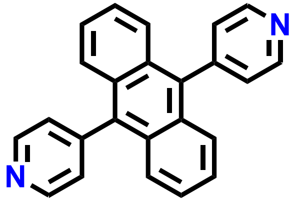 9,10-di(pyridin-4-yl)anthracene	SiF_6^{2-}	Cd^{2+}	-	Ditopic	2D	**DOHQAV**	[78]
		SiF_6^{2-}	Zn^{2+}	-	Ditopic	3D	**ZUFTUT**	[79]

36	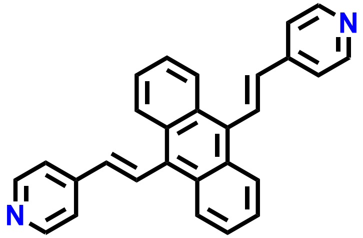 9,10-bis((*E*)-2-(pyridin-4-yl)vinyl)anthracene	SiF_6^{2-}	Zn^{2+}	Water	Ditopic	3D	**VAZJEO**	[80]
37	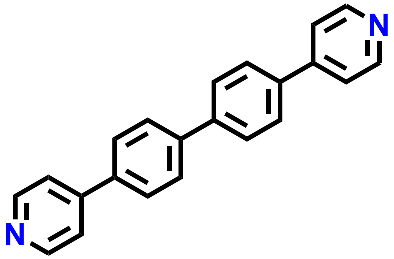 4,4'-di(pyridin-4-yl)-1,1'-biphenyl	SiF_6^{2-}	Zn^{2+}	tetrachloroethane	Ditopic	3D	**LIFWII**	[76]
38	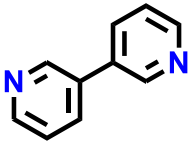 3,3'-bipyridine	SiF_6^{2-}	Zn^{2+}	Methanol	Ditopic	3D	**DOPCUK**	[81]
39	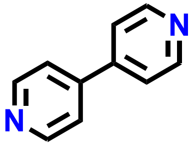 4,4'-Bipyridine	GeF_6^{2-}	Cu^{2+}	Water	Ditopic	3D	**AFEHUO**	[82]
		SiF_6^{2-}	Cu^{2+}	Water	Ditopic	3D	**AFEKAX**	[82]
		SiF_6^{2-}	Zn^{2+}	Water	Ditopic	3D	**WONZIJ**	[10]
		$NbOF_4^{-}$	Cu^{+}	Water	Ditopic	3D	**CUWRES**	[83]
		$NbOF_4^{-}$	Ag^{+}	Water	Ditopic	3D	**CUWRIW**	[83]

		SiF_6^{2-}	Cu^{2+}	-	Ditopic	3D	**HAPKOA**	[84]
		SnF_6^{2-}	Cu^{2+}	-	Ditopic	3D	**PETWES**	[85]
		TiF_6^{2-}	Cu^{2+}	-	Ditopic	3D	**PETWIW**	[85]
		SiF_6^{2-}	Cu^{2+}	4,4'-bipyridine, water	Ditopic	3D	**AYEQEC**	[86]
		SiF_6^{2-}	Cu^{2+}	Deuterated acetylene (C_2D_2)	Ditopic	3D	**EMEJAJ**	[12]
		SiF_6^{2-}	Cu^{2+}	Sulphur dioxide	Ditopic	3D	**PESVER**	[87]
		$NbOF_5^{2-}$	Ni^{2+}	Water	Ditopic	3D	**QAQWAK**	[88]
		$NbOF_5^{2-}$	Ni^{2+}	para-xylene	Ditopic	3D	**QAQWEO**	[88]
		$NbOF_5^{2-}$	Ni^{2+}	ortho-xylene	Ditopic	3D	**QAQWIS**	[88]
		SiF_6^{2-}	Cu^{2+}	melamine	Ditopic	3D	**CASYAA (01-06)**	[89]
		SiF_6^{2-}	Zn^{2+}	methanol	Ditopic	3D	**DOPCOE**	[81]
		SiF_6^{2-}	Zn^{2+}	dimethylformamide	Ditopic	3D	**ZESFUY**	[90]
		SiF_6^{2-}	Cu^{2+}	Water	Ditopic	3D	**GORWUF**	[91]
		$Cr_2O_7^{2-}$	Ni^{2+}	-	Ditopic	3D	**FOBHAG**	[92]
		$Cr_2O_7^{2-}$	Cu^{2+}	-	Ditopic	3D	**FOBHEK**	[92]

		$Cr_2O_7^{2-}$	Fe^{2+}	-	Ditopic	3D	**IRUVOG**	[93]
		SO_4^{2-}	Zn^{2+}	dimethylammonium	Ditopic	3D	**KUPJIR**	[94]
		$Cr_2O_7^{2-}$	Co^{2+}	-	Ditopic	3D	**NENCAK (01-02)**	[95]
		SO_4^{2-}	Co^{2+}	4,4'-bipyridine	Ditopic	3D	**REZVAU**	[96]
		SO_4^{2-}	Co^{2+}	-	Ditopic	3D	**REZVEY**	[96]
40	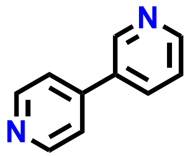 3,4'-bipyridine	MoO_4^{2-}	Ni^{2+}	Water	Ditopic	3D	**UGAQEY**	[97]
41	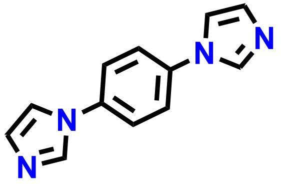 1,4-di(1*H*-imidazol-1-yl)benzene	SiF_6^{2-}	Cu^{2+}	Carbon dioxide, Ethylene	Ditopic	3D	**FAFPUB (01)**	[98]
		SiF_6^{2-}	Cu^{2+}	Methanol	Ditopic	3D	**QULPEV**	[99]
		SiF_6^{2-}	Cu^{2+}	Water	Ditopic	3D	**QULPIZ**	[99]
		SiF_6^{2-}	Cu^{2+}	-	Ditopic	3D	**QULPOF**	[99]
		SiF_6^{2-}	Cu^{2+}	-	Ditopic	3D	**QULPUL (01-03)**	[99]

			SiF_6^{2-}	Cu^{2+}	-	Ditopic	3D	**YAZBUA (01)**	[100]
			SiF_6^{2-}	Cu^{2+}	Acetylene	Ditopic	3D	**YAZCIP**	[98–100]
			SiF_6^{2-}	Cu^{2+}	Ethylene	Ditopic	3D	**YAZCOV**	[100]
			SiF_6^{2-}	Cu^{2+}	Carbon dioxide	Ditopic	3D	**YAZCUB (01)**	[100]
			TiF_6^{2-}	Cu^{2+}	Acetonitrile	Ditopic	3D	**ZEKDUS**	[101]
			SiF_6^{2-}	Cu^{2+}	Carbon monoxide	Ditopic	3D	**OGAXUT (01)**	[102]
			SiF_6^{2-}	Cd^{2+}	-	Ditopic	3D	**XIKKEL**	[103]
			SiF_6^{2-}	Cd^{2+}	-	Ditopic	3D	**XIKKIP**	[103]
			TiF_6^{2-}	Fe^{2+}	-	Ditopic	3D	**YISHUH (01)**	[104]
			ZrF_6^{2-}	Co^{2+}	-	Ditopic	3D	**YISJAP (01)**	[104]
			ZrF_6^{2-}	Fe^{2+}	-	Ditopic	3D	**YISJET (01)**	[104]
			TiF_6^{2-}	Co^{2+}	-	Ditopic	3D	**YISJIX (01)**	[104]
			SiF_6^{2-}	Cu^{2+}	-	Ditopic	3D	**ZIJRAP (01-23)**	[105]
			SiF_6^{2-}	Cu^{2+}	Propane	Ditopic	3D	**ZIJROD (01)**	[105]
			SiF_6^{2-}	Cu^{2+}	Prop-1-ene (C_3H_6)	Ditopic	3D	**ZIJSEU (01)**	[105]
			SiF_6^{2-}	Cu^{2+}	-	Ditopic	3D	**ZIJSIY (01)**	[105]
			SiF_6^{2-}	Cu^{2+}	DMSO, water	Ditopic	3D	**LUYXAI**	[106]
			SO_4^{2-}	Cu^{2+}	-	Ditopic	3D	**IZATOU**	[107]
			SO_4^{2-}	Co^{2+}	-	Ditopic	3D	**NEVBEX (01)**	[108]

		MoO_4^{2-}	Co^{2+}	-	Ditopic	3D	**QAGVON**	[109]
		SO_4^{2-}	Ni^{2+}	-	Ditopic	3D	**VATJAE**	[110]
		SO_4^{2-}	Mn^{2+}	-	Ditopic	3D	**YAMLOQ (01)**	[111]
		SO_4^{2-}	Zn^{2+}	-	Ditopic	3D	**YEGWEO**	[112]
42	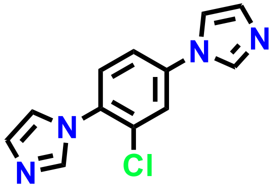 1,1'-(2-chloro-1,4-phenylene) bis(1*H*-imidazole)	SO_4^{2-}	Cu^{2+}	-	Ditopic	3D	**YUYLIR**	[113]
43	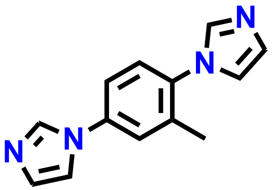 1,1'-(2-methyl-1,4-phenylene) bis(1*H*-imidazole)	SO_4^{2-}	Cu^{2+}	-	Ditopic	3D	**YUYLOX**	[113]

44	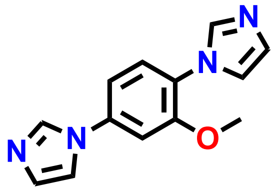<br>1,1'-(2-methoxy-1,4-phenylene) bis(1*H*-imidazole)	SO_4^{2-}	Cu^{2+}	-	Ditopic	3D	**YUYLUD**	[113]
45	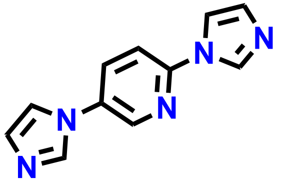<br>2,5-di(1*H*-imidazol-1-yl)pyridine	SiF_6^{2-}	Cu^{2+}	-	Ditopic	3D	**TOXDAP**	[114]
		SiF_6^{2-}	Cu^{2+}	-	Ditopic	3D	**TOXDET01**	[114]
		SiF_6^{2-}	Cu^{2+}	-	Ditopic	3D	**TOXDIX01**	[114]
46	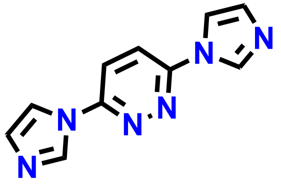<br>3,6-di(1*H*-imidazol-1-yl)pyridazine	SiF_6^{2-}	Cu^{2+}	-	Ditopic	3D	**IPOMOR (01)**	[115]
47	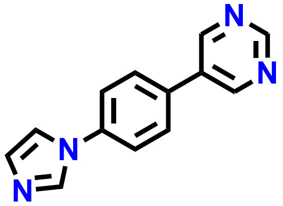<br>5-(4-(1*H*-imidazol-1-yl) phenyl)pyrimidine	SiF_6^{2-}	Cd^{2+}	-	Ditopic	3D	**LARPUS**	[116]
48		SiF_6^{2-}	Cu^{2+}	Cl^-,BF_4^-,SiF_6^{2-}	Tritopic	3D	**JISSEN (01)**	[117]

	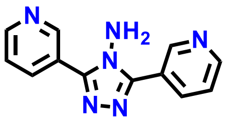<br>3,5-di(pyridin-3-yl)-4*H*-1,2,4-triazol-4-amine	ZrF_6^{2-}	Cu^{2+}	Cl^-,BF_4^-,ZrF_6^{2-}	Tritopic	3D	**JISSIR (01)**	[117]
		TiF_6^{2-}	Cu^{2+}	Cl^-,BF_4^-,TiF_6^{2-}	Tritopic	3D	**JISZAQ (01)**	[117]
49	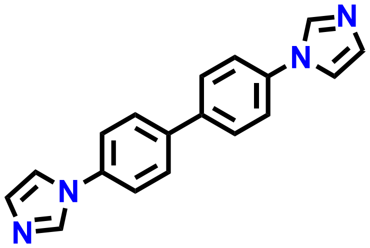<br>4,4'-di(1*H*-imidazol-1-yl)<br>-1,1'-biphenyl	MoO_4^{2-}	Co^{2+}	-	Ditopic	3D	**SESGAB**	[118]
		MoO_4^{2-}	Ni^{2+}	-	Ditopic	3D	**SESGEF**	[118]
50	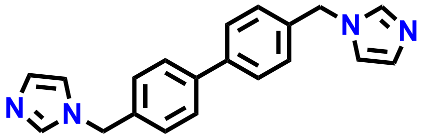<br>4,4'-bis((1*H*-imidazol-1-yl)<br>methyl)-1,1'-biphenyl	SO_4^{2-}	Co^{2+}	-	Ditopic	3D	**COKDUC**	[119]
		SO_4^{2-}	Cu^{2+}	Water	Ditopic	3D	**EWAXUV**	[120]
51	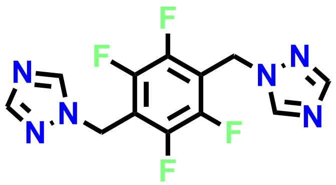<br>1,1'-((perfluoro-1,4-phenylene)<br>bis(methylene))<br>bis(1*H*-1,2,4-triazole)	SO_4^{2-}	Cu^{2+}	-	Ditopic	3D	**FEJNER**	[121]

52	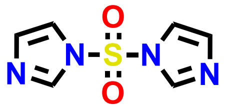 1,1'-sulfonylbis (1*H*-imidazole)	SO_4^{2-}	Ni^{2+}	-	Ditopic	3D	**VUKYEH**	[122]
53	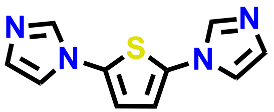 2,5-di(1*H*-imidazol-1-yl) thiophene	SO_4^{2-}	NI^{2+}	Water	Ditopic	3D	**FIZYAQ**	[123]
		SO_4^{2-}	Cu^{2+}	Water	Ditopic	3D	**FOBCEG**	[123]
		SO_4^{2-}	Co^{2+}	Water	Ditopic	3D	**FOBCIK**	[123]
54	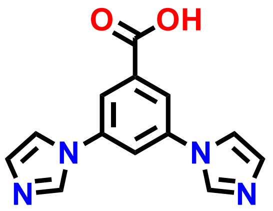 3,5-di(1*H*-imidazol-1-yl)benzoic acid	ZrF_6^{2-}	Cu^{2+}	-	Polytopic	3D	**SECWUW**	[124]
		TiF_6^{2-}	Cu^{2+}	-	Polytopic	3D	**SECXAD**	[124]
		SiF_6^{2-}	Cu^{2+}	-	Polytopic	3D	**SECXEH**	[124]
		TiF_6^{2-}	Cu^{2+}	Water	Ditopic	3D	**JIHGUG**	[125]
		TiF_6^{2-}	Cu^{2+}	Acetylene	Ditopic	3D	**JIHPOJ**	[125]
		TiF_6^{2-}	Cu^{2+}	Ethene (C_2H_4)	Ditopic	3D	**JIHPUP**	[125]
		TiF_6^{2-}	Cu^{2+}	-	Ditopic	3D	**JIHQAW**	[125]
		ZrF_6^{2-}	Cu^{2+}	-	Ditopic	3D	**JIHQEA**	[125]
		SiF_6^{2-}	Cu^{2+}	-	Ditopic	3D	**JIHQIE**	[125]
		ZrF_6^{2-}	Cu^{2+}	Ethane (C_2H_6)	Ditopic	3D	**ZOWCAT**	[126]
		ZrF_6^{2-}	Cu^{2+}	Ethane (C_2H_6)	Ditopic	3D	**ZOWCEX**	[126]

55	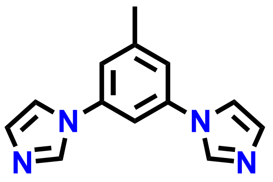 1,1'-(5-methyl-1,3-phenylene) bis(1*H*-imidazole)	ZrF_6^{2-}	Cu^{2+}	Ethane (C_2H_6)	Ditopic	3D	**ZOWCIB**	[126]
		ZrF_6^{2-}	Cu^{2+}	-	Ditopic	3D	**ZOWCOH**	[126]
		ZrF_6^{2-}	Cu^{2+}	Ethene (C_2H_4)	Ditopic	3D	**ZOWNAE**	[126]
56	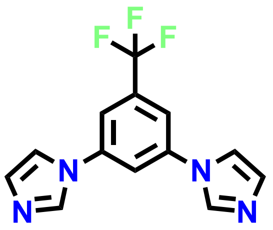 1,1'-(5-(trifluoromethyl)-1,3-phenylene) bis(1*H*-imidazole)	ZrF_6^{2-}	Cu^{2+}	Ethane (C_2H_6)	Ditopic	3D	**ZOWCUN**	[126]
		ZrF_6^{2-}	Cu^{2+}	-	Ditopic	3D	**ZOWDAU**	[126]
		ZrF_6^{2-}	Cu^{2+}	Ethene (C_2H_4)	Ditopic	3D	**ZOWDEY**	[126]
57	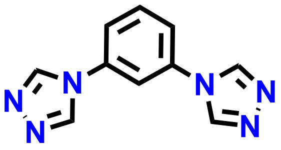 1,3-di(4*H*-1,2,4-triazol-4-yl)benzene	SO_4^{2-}	Cd^{2+}	1,2-dicarboxylic acid	Ditopic	3D	**WOXKAY**	[127]
58	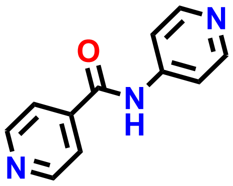 *N*-(pyridin-4-yl)isonicotinamide	SiF_6^{2-}	Cu^{2+}	Ethanol	Ditopic	3D	**GIKPIB**	[128]
59		SiF_6^{2-}	Cu^{2+}	-	Ditopic	2D	**BIFFAB**	[129]

	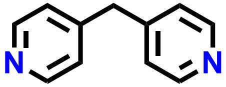 di(pyridin-4-yl)methane	SiF_6^{2-}	Cu^{2+}	Acetylene	Ditopic	2D	**BIFFEF**	[129]
		GeF_6^{2-}	Cu^{2+}	-	Ditopic	2D	**BIFFIJ (01)**	[129]
		SiF_6^{2-}	Cu^{2+}	Acetylene	Ditopic	2D	**BIFFOP**	[129]
		SiF_6^{2-}	Cu^{2+}	Carbon dioxide	Ditopic	2D	**BIFFUV**	[129]
		SiF_6^{2-}	Cu^{2+}	-	Ditopic	2D	**BIFGAC**	[129]
60	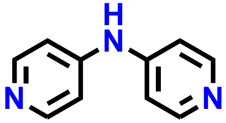 di(pyridin-4-yl)amine	SiF_6^{2-}	Cu^{2+}	Dimethylammonium	Ditopic	3D	**WODBAW**	[130]
		MoO_4^{2-}	Ni^{2+}	-	Ditopic	3D	**XAPXUH**	[131]
61	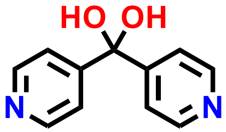 di(pyridin-4-yl)methanediol	$NbOF_5^{2-}$	Cu^{2+}	-	Ditopic	2D	**YUHGER**	[132]
62	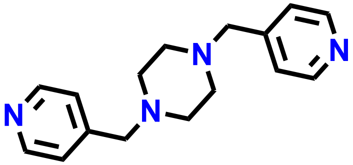 1,4-bis(pyridin-4-ylmethyl) piperazine	MoO_4^{2-}	Cu^{2+}	Water	Ditopic	3D	**IZAVUC**	[133]
63		$NbOF_5^{2-}$	Cu^{2+}	Pyrazine	Ditopic	3D	**ZOXZIV**	[133]
		SiF_6^{2-}	Zn^{2+}	-	Ditopic	3D	**FUDQIF**	[134]
		$NbOF_4^{-}$	Ag^{+}	-	Ditopic	3D	**CUWRAO**	[135]

		$NbOF_5^{2-}$	Cu^{2+}	Water	Ditopic	3D	**CEHRIS**	[136]
		SiF_6^{2-}	Zn^{2+}	-	Ditopic	3D	**KEDZAX**	[137]
		SiF_6^{2-}	Cu^{2+}	Water	Ditopic	3D	**WONKOB**	[138]
		SiF_6^{2-}	Ni^{2+}	Water	Ditopic	3D	**DUMFEY**	[139]
		$NbOF_5^{2-}$	Ni^{2+}	Water	Ditopic	3D	**ARAHIM (01-02)**	[140]
		$NbOF_5^{2-}$	Ni^{2+}	Carbon dioxide	Ditopic	3D	**OWIKAI (01)**	[141]
		SiF_6^{2-}	Fe^{2+}	Water	Ditopic	3D	**OCEGIP**	[142]
		SiF_6^{2-}	Ni^{2+}	Xenon	Ditopic	3D	**OCEGOV**	[142]
		SiF_6^{2-}	Ni^{2+}	-	Ditopic	3D	**OCEGUB (01)**	[142]
	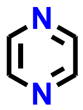	TiF_6^{2-}	Ni^{2+}	Carbon dioxide	Ditopic	3D	**RIJGIE**	[143]
	pyrazine	SiF_6^{2-}	Ni^{2+}	Propyne (C_3H_4)	Ditopic	3D	**PESVOB**	[144]
		$NbOF_5^{2-}$	Ni^{2+}	Dihydrogen sulfide	Ditopic	3D	**QOXKAS**	[145]
		$NbOF_5^{2-}$	Ni^{2+}	Sulfur dioxide	Ditopic	3D	**HIZMOV**	[146]
		GeF_6^{2-}	Fe^{2+}	-	Ditopic	3D	**HOXWOJ**	[147]
		GeF_6^{2-}	Co^{2+}	-	Ditopic	3D	**HOYXIF (01)**	[147]
		$NbOF_5^{2-}$	Ni^{2+}	-	Ditopic	3D	**REDQUQ**	[148]
		$NbOF_5^{2-}$	Ni^{2+}	Propylene (C_3H_6)	Ditopic	3D	**REDRAX**	[148]
		$NbOF_5^{2-}$	Ni^{2+}	Propane (C_3H_8)	Ditopic	3D	**REDREB**	[148]

		TiF_6^{2-}	Co^{2+}	-	Ditopic	3D	**SUHQUK (01)**	[149]
		GeF_6^{2-}	Ni^{2+}	-	Ditopic	3D	**ZUTVAO**	[150]
		SiF_6^{2-}	Ni^{2+}	Water	Ditopic	3D	**POCQUX**	[151]
		SiF_6^{2-}	Ni^{2+}	Water	Ditopic	3D	**POCRAE**	[151]
		SiF_6^{2-}	Ni^{2+}	Carbon dioxide	Ditopic	3D	**POCRIM**	[151]
		$TaOF_5^{2-}$	Ni^{2+}	-	Ditopic	3D	**VANJAZ**	[152]
		AlF_5^{2-}	Ni^{2+}	Water	Ditopic	3D	**DAXNIC**	[153]
		AlF_5^{2-}	Ni^{2+}	Water	Ditopic	3D	**DAXNOI**	[153]
		AlF_5^{2-}	Ni^{2+}	Carbon dioxide	Ditopic	3D	**DAXNUO**	[153]
		AlF_5^{2-}	Ni^{2+}	Hydrogen sulphide	Ditopic	3D	**QOXKEW**	[154]
		AlF_5^{2-}	Ni^{2+}	Carbon dioxide	Ditopic	3D	**QOXKIA**	[154]
		GaF_6^{2-}	Cu^{2+}	Water	Ditopic	3D	**UXOZIS02**	[155]
		FeF_5^{2-}	Ni^{2+}	Water	Ditopic	3D	**DAXPAW**	[153]
		VF_6^{2-}	Cu^{2+}	Water	Ditopic	3D	**UXOZEO02**	[155]
64	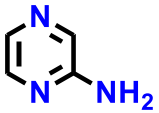 pyrazin-2-amine	TiF_6^{2-}	Ni^{2+}	-	Ditopic	3D	**UQAVAM**	[156]

65	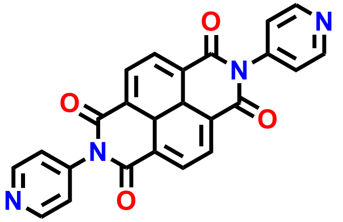 2,7-di(pyridin-4-yl)-3a1,5a1-dihydrobenzo[*lmn*][3,8]phenanthroline-1,3,6,8(2*H*,7*H*)-tetraone	SiF_6^{2-}	Zn^{2+}	-	Ditopic	3D	**FORKOO**	[157]
		SiF_6^{2-}	Zn^{2+}	Iodine	Ditopic	3D	**FORKUU**	[157]
66	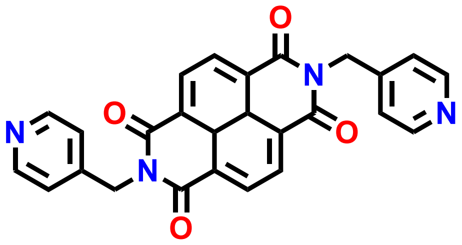 2,7-bis(pyridin-4-ylmethyl)-3a1,5a1-dihydrobenzo[*lmn*][3,8]phenanthroline-1,3,6,8(2*H*,7*H*)-tetraone	SiF_6^{2-}	Cd^{2+}	Methanol, chloroform	Ditopic	3D	**YOPJOF**	[158]
67	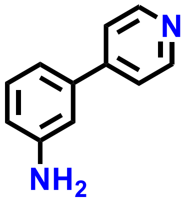 3-(pyridin-4-yl)aniline	SiF_6^{2-}	Cd^{2+}	Dichloromethane Water	Ditopic	3D	**POMCUR**	[159]
68		SiF_6^{2-}	Cu^{2+}	-	Tritopic	3D	**EMOWAH (01-03)**	[160]

	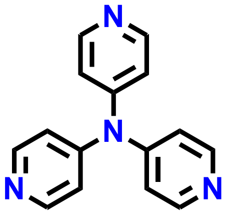 tri(pyridin-4-yl)amine	SiF_6^{2-}	Cu^{2+}	-	Tritopic	3D	**PESXOE (01)**	[161]
		$NbOF_5^{2-}$	Cu^{2+}	-	Tritopic	3D	**PETMEK**	[162]
		TiF_6^{2-}	Cu^{2+}	Propane	Tritopic	3D	**YUGCEM**	[163]
		SnF_6^{2-}	Cu^{2+}	Water	Tritopic		**KOPNOW**	[164]
		CrO_4^{2-}	Cu^{2+}	-	Tritopic	3D	**TORQEA**	[165]
		MoO_4^{2-}	Cu^{2+}	-	Tritopic	3D	**TORQIE**	[165]
		WO_4^{2-}	Cu^{2+}	-	Tritopic	3D	**TORQOK**	[165]
69	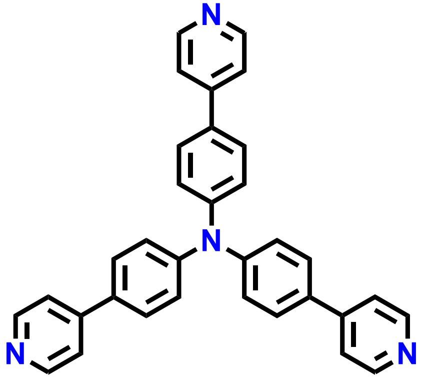 tris(4-(pyridin-4-yl)phenyl)amine	SO_4^{2-}	Cd^{2+}	-	Tritopic	3D	**CARGAG**	[166]
70		TiF_6^{2-}	Cu^{2+}	TiF_6^{2-}	Tritopic	3D	**WAHKUO**	[167]

	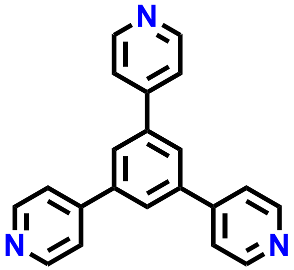 1,3,5-tri(pyridin-4-yl)benzene	SnF_6^{2-}	Cu^{2+}	SnF_6^{2-}	Tritopic	3D	**WAHNEB**	[167]
		ZrF_6^{2-}	Cu^{2+}	ZrF_6^{2-}	Tritopic	3D	**WAHNIF**	[167]
		SiF_6^{2-}	Cu^{2+}	SiF_6^{2-}	Tritopic	3D	**WAHNOL**	[167]
		GeF_6^{2-}	Cu^{2+}	GeF_6^{2-}	Tritopic	3D	**WAJDUJJ**	[167]
71	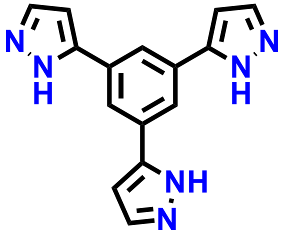 1,3,5-tri(1*H*-pyrazol-5-yl)benzene	SO_4^{2-}	Ni^{2+}	Methanol	Tritopic	3D	**ATIJEV**	[168]
72	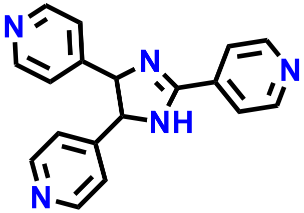 4,4',4''-(4,5-dihydro-1*H*-imidazole-2,4,5-triyl) tripyridine	SO_4^{2-}	Cu^{2+}	-	Tritopic	2D	**NELXAG**	[169]
		SO_4^{2-}	Cd^{2+}	Water	Tritopic	2D	**XILDOO**	[170]
		SO_4^{2-}	Zn^{2+}	Water	Tritopic	2D	**XILLOW**	[170]

73	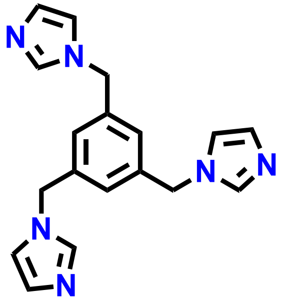 1,3,5-tris((1*H*-imidazol-1-yl) methyl)benzene	SO_4^{2-}	Mn^{2+}	-	Tritopic	3D	**AYAWEE**	[171]
		SO_4^{2-}	Cd^{2+}	-	Tritopic	3D	**WAGLEY**	[172]
74	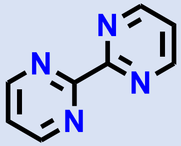 2,2'-Bipyrimidine	SO_4^{2-}	Cu^{2+}	-	Tetratopic Chelating	3D	**PAZXUJ (01)**	[173]
		SnF_5OH^{2-}	Cu^{2+}	Water	Tetratopic Chelating	3D	**ISERUX**	[174]
		$NbOF_5^{2-}$	Cu^{2+}	-	Tetratopic Chelating	3D	**ISIPAF**	
75	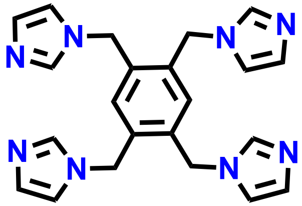 1,2,4,5-tetrakis((1*H*-imidazol-1-yl) methyl)benzene	SO_4^{2-}	Cd^{2+}	-	Ditopic and tetratopic	3D	**HORHUS**	[175]

76	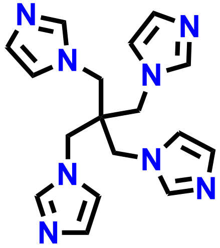 1,1'-(2,2-bis((1*H*-imidazol-1-yl) methyl)propane-1,3-diyl) bis(1*H*-imidazole)	SO_4^{2-}	Co^{2+}	Water	Tetratopic	3D	**CUYTOG**	[176]
		WO_4^{2-}	Co^{2+}	Water	Tetratopic	3D	**TESQEP**	[177]
77	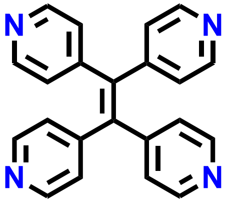 1,1,2,2-tetra(pyridin-4-yl)ethene	SiF_6^{2-}	Zn^{2+}	-	Tetratopic	3D	**RIRCUU**	[178]
		SiF_6^{2-}	Zn^{2+}	Acetylene	Tetratopic	3D	**RIRDAB**	[178]
		SiF_6^{2-}	Zn^{2+}	Ethene	Tetratopic	3D	**RIRDEF**	[178]
		SiF_6^{2-}	Cd^{2+}	-	Tetratopic	3D	**XUTDOJ**	[179]
78		SO_4^{2-}	Fe^{2+}	DMSO, water	Tetratopic	3D	**IGOZOX**	[180]

	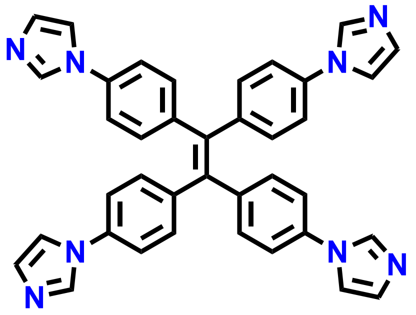 1,1,2,2-tetrakis(4-(1*H*-imidazol-1-yl) phenyl)ethene	SO_4^{2-}	Cd^{2+}	Water, OH^-	Tetratopic	3D	**SIYJIX**	[181]
		SO_4^{2-}	Cd^{2+}	Water	Tetratopic	3D	**SIYJOD**	[181]
79	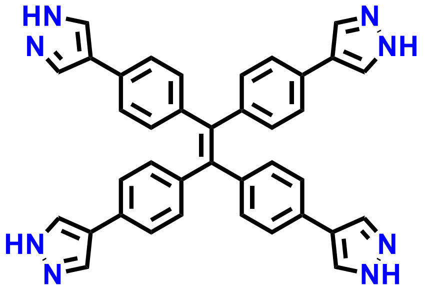 1,1,2,2-tetrakis(4-(1*H*-pyrazol-4-yl) phenyl)ethene	SO_4^{2-}	Co^{2+}	-	Tetratopic	3D	**LUWQON**	[182]
		SO_4^{2-}	Zn^{2+}	-	Tetratopic	3D	**LUWQUT**	[182]
80	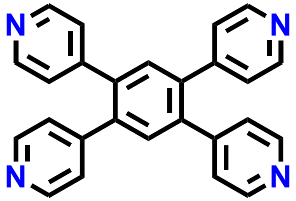 1,2,4,5-tetra(pyridin-4-yl)benzene	SiF_6^{2-}	Zn^{2+}	Methanol	Tetratopic	3D	**FAVJEV**	[183]
		SiF_6^{2-}	Zn^{2+}	-	Tetratopic	3D	**QANMAX**	[184]
		ZrF_6^{2-}	Zn^{2+}	-	Tetratopic	3D	**SEKVEN**	[185]
		SnF_6^{2-}	Zn^{2+}	-	Tetratopic	3D	**SEKVOX**	[185]
		GeF_6^{2-}	Zn^{2+}	-	Tetratopic	3D	**SEKVUD**	[185]
		TiF_6^{2-}	Zn^{2+}	-	Tetratopic	3D	**SEKWAK**	[185]
		SiF_6^{2-}	Cu^{2+}	-	Tetratopic	3D	**KOWHUD**	[186]

		TaF_7^{2-}	Zn	-	Tetratopic	3D	**SEKVIR**	[185]
		SO_4^{2-}	Zn^{2+}	Methanol	Tetratopic	3D	**FATSIG**	[183]
		MoO_4^{2-}	Co^{2+}	-	Tetratopic	3D	**SUKPUN**	[187]
81	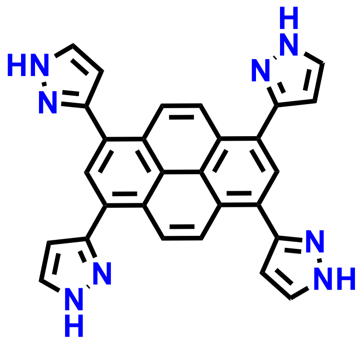 1,3,6,8-tetra(1*H*-pyrazol-3-yl) pyrene	SO_4^{2-}	NI^{2+}	-	Tetratopic	3D	**JUKLUA**	[188]
		SO_4^{2-}	Fe^{2+}	-	Tetratopic	3D	**UGIGEA**	[189]
82	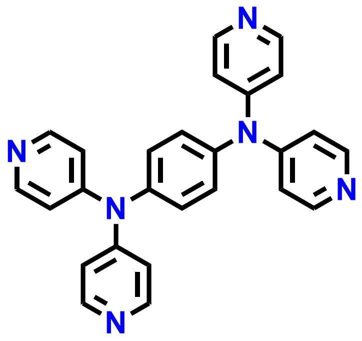 *N*¹,*N*¹,*N*⁴,*N*⁴-tetra(pyridin-4-yl) benzene-1,4-diamine	SiF_6^{2-}	Cu^{2+}	-	Tetratopic	3D	**YIMVID**	[190]
		AlF_6^{3-}	Cu^{2+}	AlF_6^{3-}, water	Tetratopic	3D	**YIMVOJ**	[190]

83	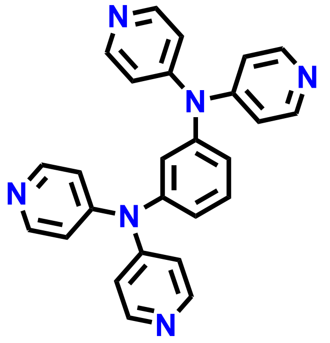 *N*[1],*N*[1],*N*[3],*N*[3]-tetra(pyridin-4-yl) benzene-1,3-diamine	SiF_6^{2-}	Cu^{2+}	Methanol, water	Tetratopic	3D	**BOSNUW**	[191]
84	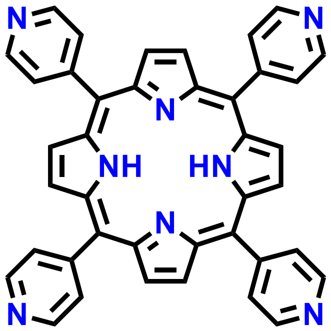 5,10,15,20-tetra(pyridin-4-yl)porphyrin	SiF_6^{2-}	Zn^{2+} (Porphyrin pocket with Fe)	-	Tetratopic	3D	**CECGOJ**	[192]
		SiF_6^{2-}	Zn^{2+} (Porphyrin pocket with Zn)	-	Tetratopic	3D	**CECGUP**	[192]
		SO_4^{2-}	Co^{2+} (porphyrin pocket	-	Tetratopic	3D	**LOZHIV**	[193]

			with Co)					
		SO_4^{2-}	Co^{2+} (porphyrin pocket with Cu)	-	Tetratopic	3D	**LOZHOB**	[193]
		SO_4^{2-}	Co^{2+} (porphyrin pocket with Zn)	-	Tetratopic	3D	**LOZKAQ**	[193]
85	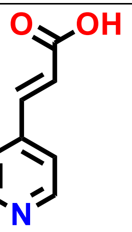<br>(*E*)-3-(pyridin-4-yl)acrylic acid	SiF_6^{2-}	Cu^{2+}	Water	Polytopic	3D	**ZAHLAY**	[194]

86	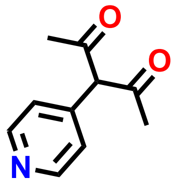 3-(4-pyridyl)pentane-2,4dione	SiF_6^{2-}	Cu^{2+}	-	Ditopic (overall, considering metallo-ligand as one)	3D	**HINZOX**	[195]
87	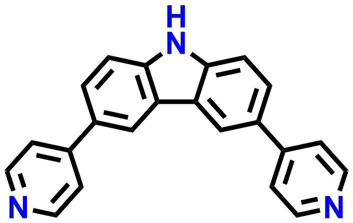 3,6-di(pyridin-4-yl)-9*H*-carbazole	SiF_6^{2-}	Zn^{2+}	-	Ditopic	3D	**REDFAK**	[196]
88	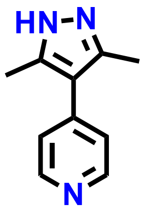 4-(3,5-dimethyl-1*H*-pyrazol-4-yl)pyridine	SiF_6^{2-}	Cu^{2+}	Methanol	Ditopic	3D	**EZOTAR**	[197]
		TiF_6^{2-}	Cu^{2+}	Methanol	Ditopic	3D	**EZOTEV**	[197]
		SiF_6^{2-}	Ni^{2+}	Methanol	Ditopic	3D	**EZOTIZ**	[197]
		TiF_6^{2-}	Ni^{2+}	Methanol	Ditopic	3D	**EZOTOF**	[197]
		ZrF_6^{2-}	Cu^{2+}	Ethylene glycol	Ditopic	3D	**DUKJUS**	[198]
		ZrF_6^{2-}	Cu^{2+}	Water	Ditopic	3|D	**DUKMIJ**	[198]
		$Cr_2O_7^{2-}$	Cu^{2+}	-	Ditopic	3D	**DEXFEU**	[199]

89	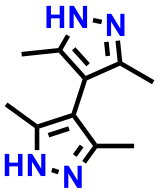 3,3',5,5'-tetramethyl-1*H*,1'*H*-4,4'-bipyrazole	SiF_6^{2-}	Co^{2+}	Chloroform, methanol	Ditopic	3D	**CIKGUZ**	[200]
		SiF_6^{2-}	Cd^{2+}	Chloroform	Ditopic	3D	**KIKDEP**	[201]
		SiF_6^{2-}	Cd^{2+}	Water	Ditopic	3D	**KIKDOZ**	[201]
		SiF_6^{2-}	Cd^{2+}	Dichloromethane	Ditopic	3D	**KIKDUF**	[201]
		SO_4^{2-}	Co^{2+}	ClO_4^-	Ditopic	3D	**MIGVED**	[202]
		SO_4^{2-}	Ni^{2+}	NO_3^-	Ditopic	3D	**MIGVIH**	[202]
		SO_4^{2-}	Co^{2+}	Chloroform	Ditopic	3D	**MIGVON**	[202]
		SO_4^{2-}	Ni^{2+}	Chloroform	Ditopic	3D	**MIGVUT**	[202]
90	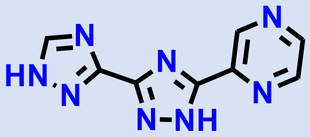 5-(pyrazin-2-yl)-1*H*,1'*H*-3,3'-bi(1,2,4-triazole)	SO_4^{2-}	Mn^{2+}	Water	Polytopic, **Chelating**	3D	**TEDMAT**	[203]
		SO_4^{2-}	Zn^{2+}	Water	Polytopic, **Chelating**	3D	**XORGOC**	[204]

91	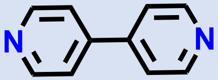 4,4'-bipyridine 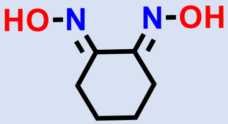 (1*E*,2*E*)-cyclohexane -1,2-dione dioxime	SO_4^{2-}	Zn^{2+}	Water	Ditopic **Chelating**	3D	**EXEHIZ**	[205]
92	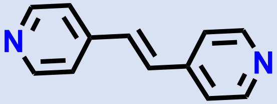 (*E*)-1,2-di(pyridin-4-yl)ethene 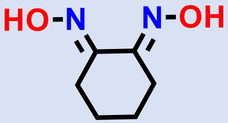 (1*E*,2*E*)-cyclohexane -1,2-dione dioxime	SO_4^{2-}	Zn^{2+}	Water	Ditopic **Chelating**	3D	**EXEHOF**	[205]
93	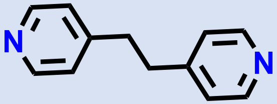 1,2-di(pyridin-4-yl)ethane 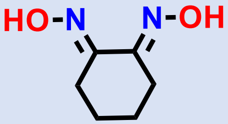 (1*E*,2*E*)-cyclohexane -1,2-dione dioxime	SO_4^{2-}	Zn^{2+}	Water	Ditopic **Chelating**	3D	**EXEHUL**	[205]

94	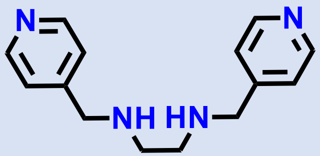<br>*N*1,*N*2-bis(pyridin-4-ylmethyl) ethane-1,2-diamine	MoO_4^{2-}	Zn^{2+}	Water	Tetratopic, **chelating**	3D	**ZOMNEW**	[206]
		SO_4^{2-}	Zn^{2+}	Water	Tetratopic, **chelating**	3D	**ZOMNOG**	[206]
		CrO_4^{2-}	Zn^{2+}	Water	Tetratopic, **chelating**	3D	**ZOMNUM**	[206]
		SiF_6^{2-}	Zn^{2+}	Water	Tetratopic, **chelating**	3D	**UGAZEL**	[207]
95	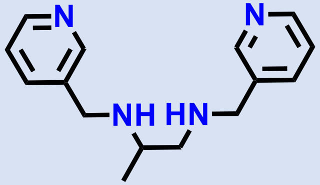<br>*N*1,*N*2-bis(pyridin-3-ylmethyl) propane-1,2-diamine	SO_4^{2-}	Cd^{2+}	-	Tetratopic, **chelating**	3D	**WOXWIS**	[208]

No. = number of CSD entries in sequence.

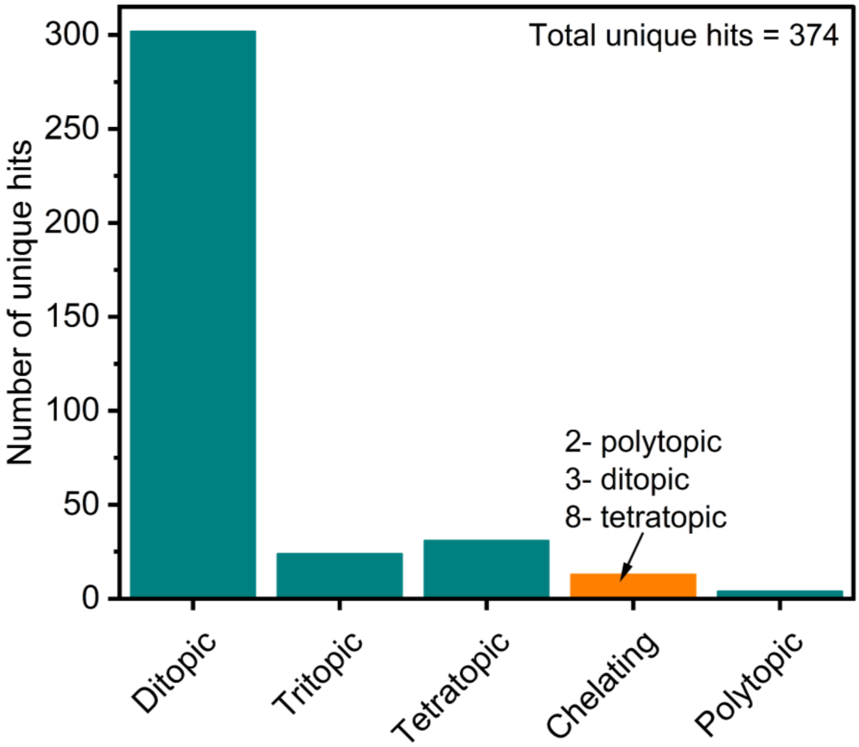

Figure S3: Distribution of ligand topicity among HCNs retrieved from the CSD (n = 374 unique Refcodes). Bars show counts for ditopic, tritopic, tetratopic, chelating, and polytopic ligands. Polytopic ligands refer here to linkers with complex or variable connectivity that cannot be unambiguously classified as di-, tri-, or tetratopic. Entries tagged as both *e.g.*, polytopic and chelating (n = 2) were counted in the chelating category only. Chelating HUMs (13 entries) account for 3.47% of all HCNs reported to date in the CSD.

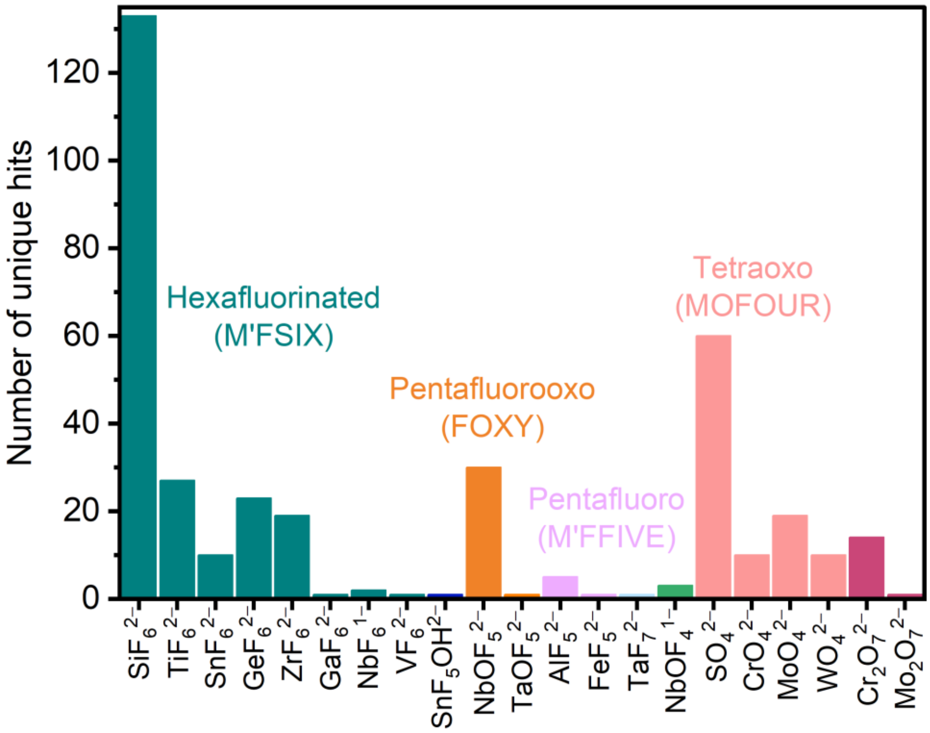

Figure S4: Distribution of inorganic ligands in the CSD-derived dataset (counts are unique hits). Individual anions are listed on the x-axis; coloured annotations group them into families: hexafluorinated ("MFSIX" ; *e.g.*, SiF_6^{2-}, TiF_6^{2-}, GeF_6^{2-}, ZrF_6^{2-}, SnF_6^{2-}), oxopentafluoro ("FOXY"; $NbOF_5^{2-}$, $TaOF_5^{2-}$), pentafluoro ("MFFIVE" ; AlF_5^{2-}), and tetraoxo ("MOFOUR"; SO_4^{2-}, CrO_4^{2-}, MoO_4^{2-}, WO_4^{2-}, etc.). As shown, SiF_6^{2-} dominates the corpus, with smaller but notable contributions from FOXY and MOFOUR families.

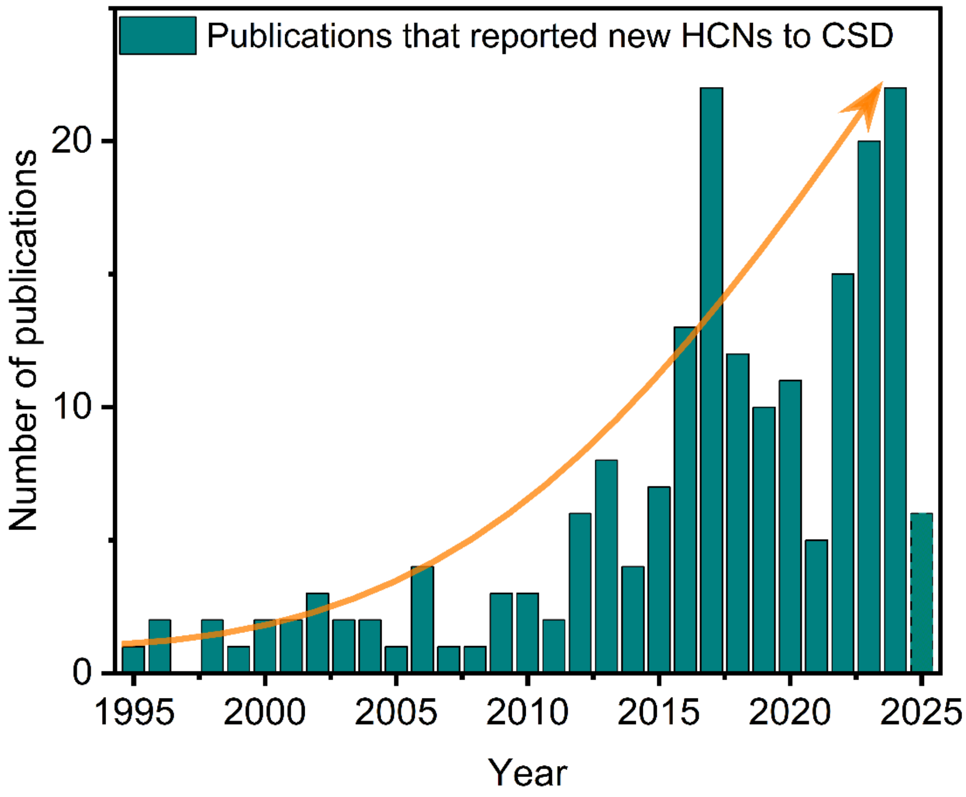

Figure S5: Yearly number of publications reporting new HCNs with crystallographic data deposited to the CSD (1995–2025), from entries listed in Table S1. Bars give counts per calendar year; the 2025 bar (hatched) is year-to-date (11/09/2025). The orange arrow is a guide-to-the-eye emphasising the accelerating growth in reports over the last decade.

Single Crystal Structural Information

Single crystal X-ray diffraction data for **TIFSIX-enmepy-Zn**, **SNIFSIX-enmepy-Zn**, and **NbOFFIVE-enmepy-Zn** were recorded on a Bruker Quest diffractometer equipped with a CMOS detector and IµS microfocus X-ray source (Mo Kα, λ = 0.71073 Å) under N_2 flow at 150 K. All SCXRD data were indexed, integrated and scaled in APEX.[209] Absorption correction was performed by multi-scan method using SADABS.[210] Space group determination was achieved using XPREP4 implemented in APEX4. Structures were solved using intrinsic phasing method (SHELXT) and refined on F^2 using nonlinear least-squares techniques with SHELXL programs[211] incorporated in X-seed graphical user interface. Anisotropic thermal parameters were applied to all non-hydrogen atoms. Hydrogen atoms were placed in calculated positions using riding models. Pore volumes and geometries (pore limiting diameters and maximum pore diameters) were calculated using the Pore Analyser (probe radius of 1.2 Å, default settings) feature in Mercury. Crystal structures and void volumes were visualised using Mercury.

Table S2: Crystallographic and refinement data of compounds **TIFSIX-enmepy-Zn**, **SNIFSIX-enmepy-Zn** and **NbOFFIVE-enmepy-Zn**.

	**TIFSIX-enmepy-Zn**	**SNIFSIX-enmepy-Zn**	**NbOFFIVE-enmepy-Zn**
CCDC number	2485704	2485705	2485706
Formula	$C_{14}H_{18}F_6N_4TiZn·4.5H_2O$	$C_{14}H_{18}F_6N_4NbZn·7.5H_2O$	$C_{14}H_{18}F_6N_4NbZn·4.5H_2O$
Formula weight	550.66	675.50	595.67
Temperature (K)	150	150	150
Crystal system	Monoclinic		
Space group	*C*2/*c*		
Z	4	4	4
a(Å)	13.7471(18)	13.8347(6)	13.7818(12)
b(Å)	12.5104(17)	12.4301(6)	12.6215(11)
c(Å)	15.476(2)	15.7486(7)	15.5317(13)
β(°)	90.282(3)	90.0120(10)	91.2870(10)
V (Å^3)	2661.6(6)	2708.2(2)	2701.0(4)
D_c (g/cm $^{-3}$)	1.374	1.657	1.465
μ(mm^{-1})	1.267	1.888	1.377
F(000)	1124	1356.0	1200
Crystal size (mm^3)	0.25 x 0.22 x 0.16	0.28 × 0.2 × 0.15	0.26 × 0.21 × 0.15
Reflections collected/unique	47120/2339 [R_{int}= 0.0507]	22659/2372 [R_{int}= 0.0424]	35808/2372 [R_{int}= 0.1085]
Reflections with [I>2σ(I)]	2310	2266	1950
Parameters	79	79	120
GOF on F^2	1.016	1.018	1.069
R_1, wR_2 [I>2σ(I)]	0.0476, 0.1347	0.0313, 0.0824	0.0391, 0.0870
R_1, wR_2 (all data)	0.0480, 0.1350	0.0335, 0.0839	0.0514, 0.0899

Table S3: List of bond lengths and bond angles in **TIFSIX-enmepy-Zn**, **SNIFSIX-enmepy-Zn** and **NbOFFIVE-enmepy-Zn**.

Compound	**TIFSIX-enmepy-Zn**	**SNIFSIX-enmepy-Zn**	**NbOFFIVE-enmepy-Zn**
Bond lengths			
Zn(1)-N(1)	2.126(4)	2.1210(19)	2.137(3)
Zn(1)-N(2)*	2.149(4)	2.136(4)	2.171(3)
Zn(1)-F(1)	2.139(3)	2.152(3)	2.1086(19)
Me(1)-(F1)	1.901(3)	1.990(3)	1.9251(19)
Me(1)-(F2)	1.863(4)	1.970(5)	1.924(2)
Me(1)-(F3)	1.872(4)	1.931(5)	1.922(2)
	[a] 1/2-X,1/2+Y,1/2-Z	[a] -1/2+X,1/2+Y,+Z	[a] -1/2+X,-1/2+Y,+Z
Bond Angles			
F(1)-Me(1)-F(1)*	180.0[b]	180.0[b]	180.0[b]
F(2)-Me(1)-F(2)*	180.0[b]	180.0[b]	180.0[b]
F(3)-Me(1)-F(3)*	180.0[b]	180.0[b]	180.0[b]
F(1)-Me(1)-F(2)	89.61(14)	89.6(2)	90.40(9)
F(1)*-Me(1)-F(2)	90.39(14)[c]	90.4(2)[b]	89.60(9)[b]
F(1)-Me(1)-F(3)	90.06(15)	89.5(2)	89.31(9)
F(1)-Me(1)-F(3)*	89.94(15)[c]	90.5(2)[b]	90.69(9)[b]
F(2)-Me(1)-F(3)	91.17(19)	89.63(18)	90.28(11)
F(2)-Me(1)-F(3)*	88.83(19)[c]	90.37(18)[b]	89.71(11)[b]
F(1)-Zn(1)-F(1)*	175.63(14)[b]	175.21(15)[c]	175.14(12)[c]
F(1)-Zn(1)-N(1)	90.15(17)	89.99(15)	90.58(9)
F(1)-Zn(1)-N(1)*	93.00(17)[b]	89.99(15)[c]	92.91(9)[c]
F(1)-Zn(1)-N(2)*	90.16(14)[b]	88.5(3)[d]; 87.9(3)[e]	86.28(9)[d]
F(1)-Zn(1)-N(3)*	-	-	90.05(9)[e]
N(1)-Zn(1)-N(1)*	87.8(2)[b]	88.68(16)[c]	88.33(15)[c]
N(1)-Zn(1)-N(2)*	94.56(15)[d]; 176.05(17)[e]	94.03(13)[e]; 176.67(18)[d]	94.87(11)[e]; 175.63(10)[d]
N(2)*-Zn(1)-N(2)**	83.3(2)[e, d]	83.3(2)[e, d]	82.10(15)[d,e]
	[b]1-x,+y,1/2-z; [c]1-x,1-y,1-z; [d]1/2-x,1/2+y,1/2-z; [e]1/2+x,1/2+y,+z	[b]1-x,1-y,1-z; [c]1-x,+y,3/2-z; [d]-1/2+x,1/2+y,+z; [e]3/2-x,1/2+y,3/2-z	[b]1-x,1-y,1-z; [c]1-x,+y,3/2-z; [d] -1/2+x,-1/2+y,+z; [e] 3/2-x,-1/2+y,3/2-z

Me = Ti, Sn, Nb

Table S4: Classic hydrogen bond distances (Å) and angles (°) in **TIFSIX-enmepy-Zn**, **NbOFFIVE-enmepy-Zn**, and **SNIFSIX-enmepy-Zn**.

**Compound**	**D-H...A**	**d(H...A)**	**d(D...A)**	**<(DHA)**	**Symmetry transformations for acceptor**
**TIFSIX-enmepy-Zn**	N(2)-H(2)...F(3)	2.049(4)	3.008(6)	160.0(3)	x-1/2, y-1/2, z
**NbOFFIVE-enmepy-Zn**	N(2)-H(2)...F(3)	2.087(2)	3.043(4)	159.17(17)	1/2+x, 1/2+y, z
**SNIFSIX-enmepy-Zn**	N(2)-H(2A)...F(3)	2.134(6)	3.105(12)	162.2(3)	1-x, 1-y, 1-z

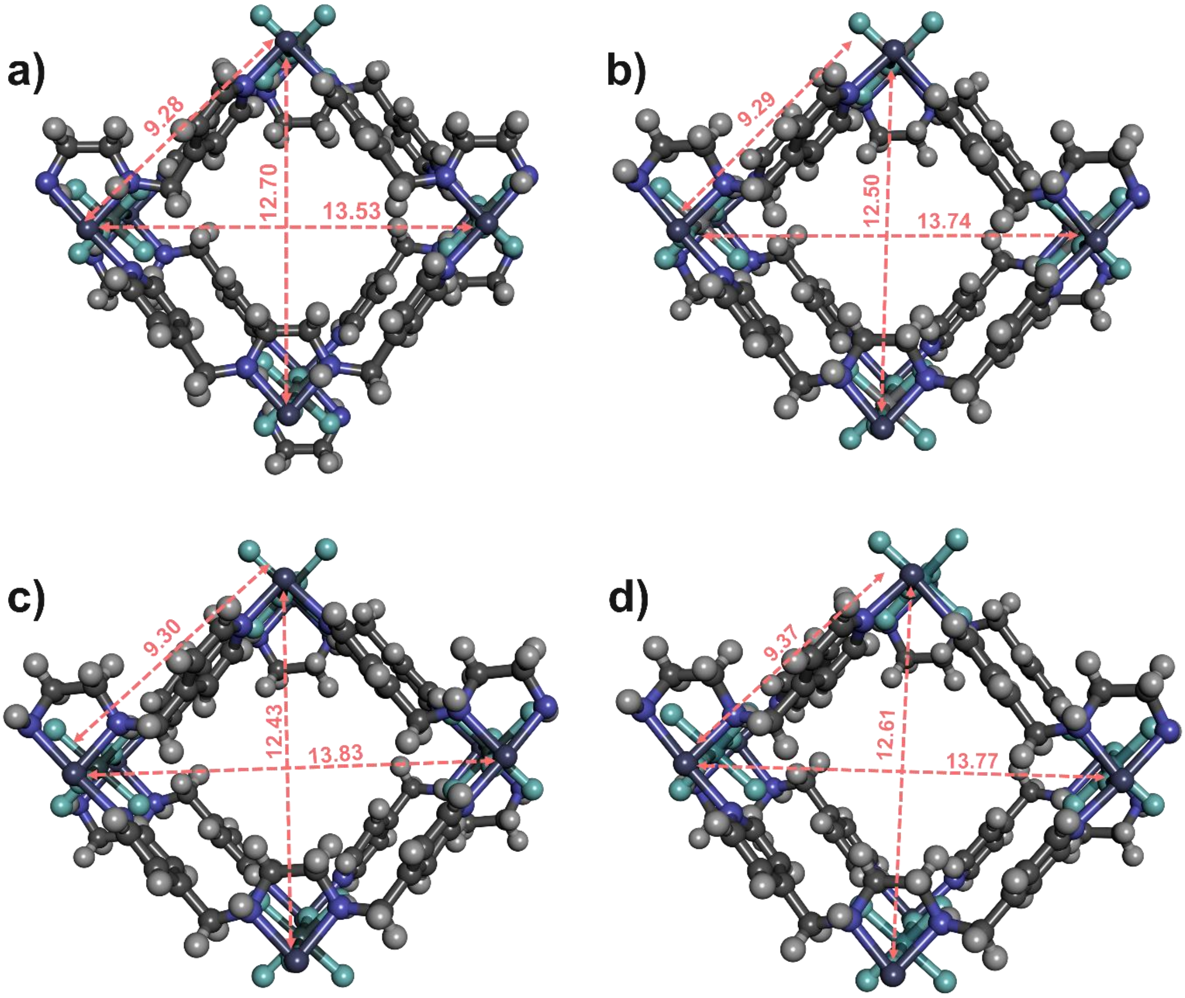

Figure S6: Top-view of the chelating HUM structures, showing distance (in Å) between the Zn(II) nodes from the adjacent **sql** layers: a) **SIFSIX-24-Zn**, b) **TIFSIX-enmepy-Zn**, c) **SNIFSIX-enmepy-Zn** and d) **NbOFFIVE-enmepy-Zn**. Colour code: Zn (dark blue), Ge (dark teal), F (green), N (blue), C (dark grey), H (light grey), Si (light cyan), Ti (aquamarine), Sn (turquoise) and Nb (teal).

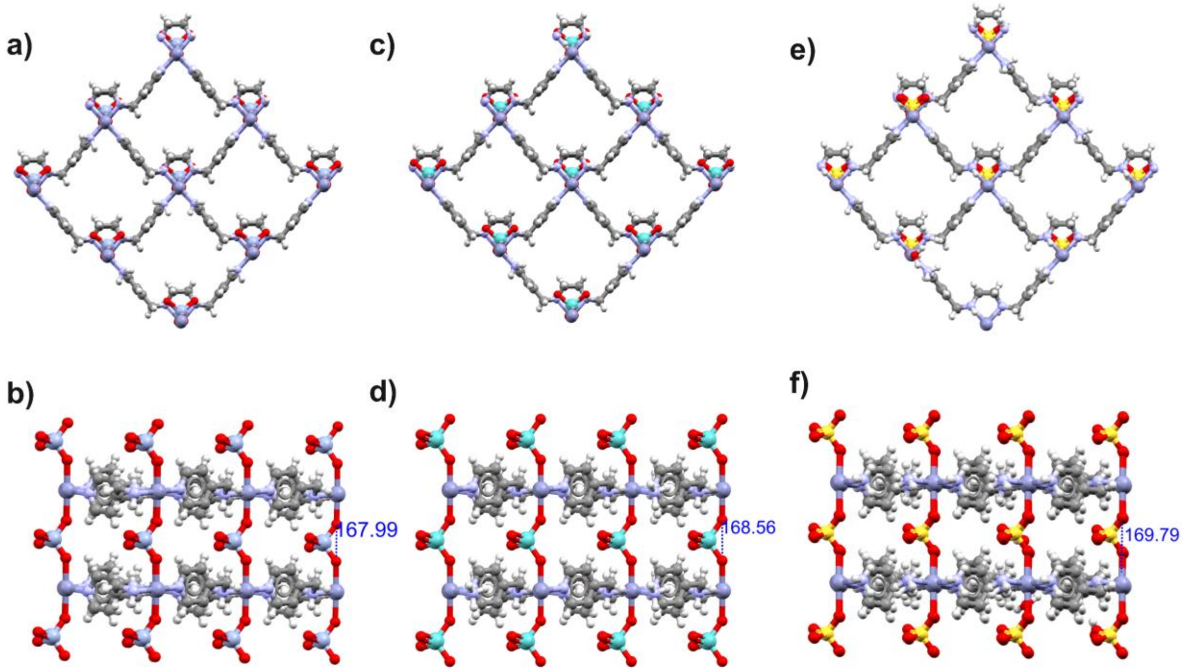

Figure S7: Single-crystal structures of previously reported chelating HUMs illustrating packing viewed along *c*-axis; a), c) and e) showing a non-alternate stacking of **sql** layers of $[Zn(enmepy)(MoO_4)]_n$ (**MOFOUR-enmepy-Zn**) CSD Refcode ZOMNEW, $[Zn(enmepy)(CrO_4)]_n$ (**CROFOUR-enmepy-Zn**) CSD Refcode ZOMNUM and $[Zn(enmepy)(SO_4)]_n$ (**SOFOUR-enmepy-Zn**) CSD Refcode ZOMNOG respectively ; b), d)) and f) shows packing along *a*-axis highlighting near linear pillaring (∠ Zn-O-Zn angle close to 180°) of the layers.

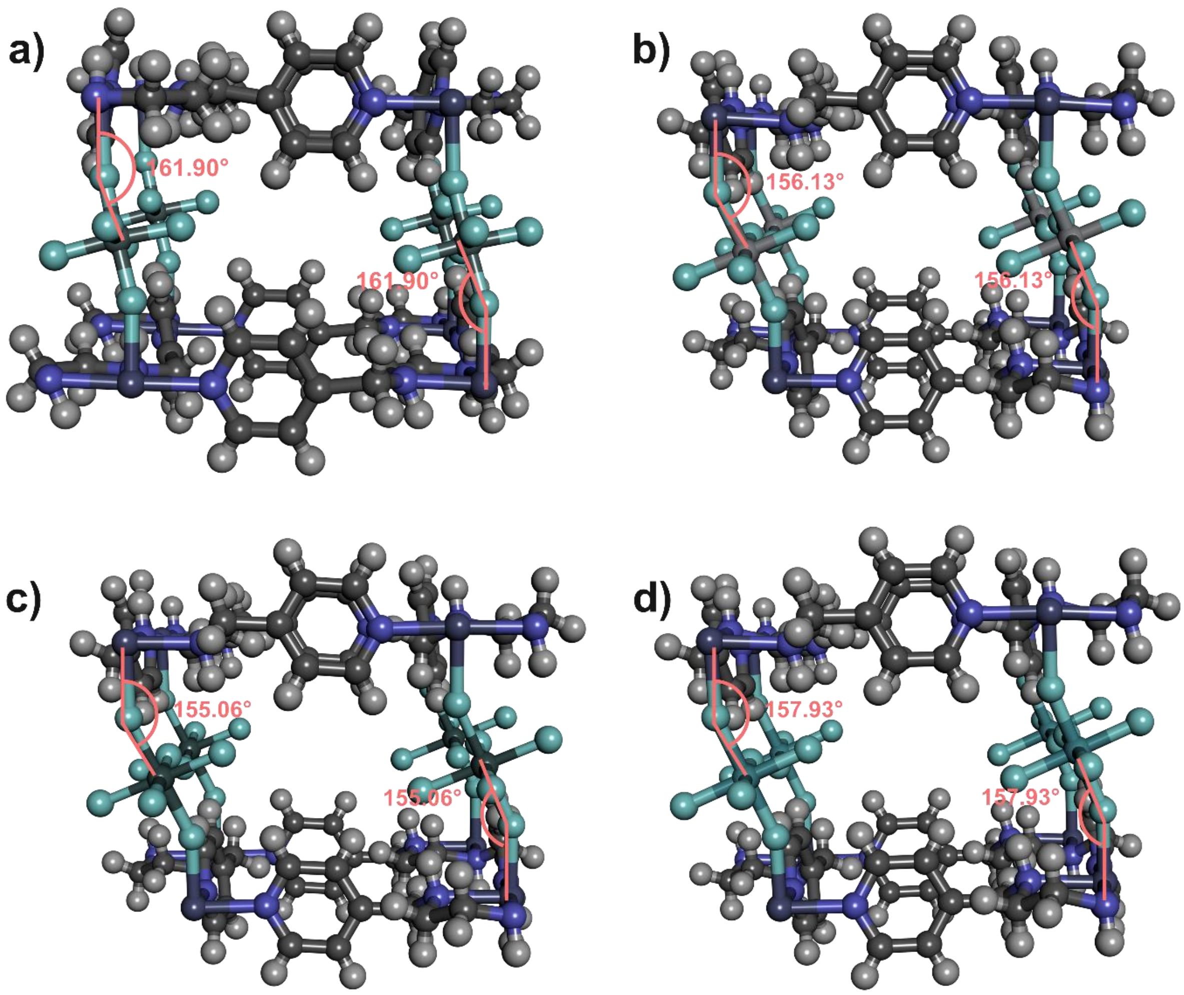

Figure S8: Side view of the chelating HUM structures, highlighting the pillaring angle (∠M-F-X) a) **SIFSIX-24-Zn**, b) **TIFSIX-enmepy-Zn**, c) **SNIFSIX-enmepy-Zn** and d) **NbOFFIVE-enmepy-Zn**. Colour code: Zn (dark blue), Ge (dark teal), F (green), N (blue), C (dark grey), H (light grey), Si (light cyan), Ti (aquamarine), Sn (turquoise) and Nb (teal).

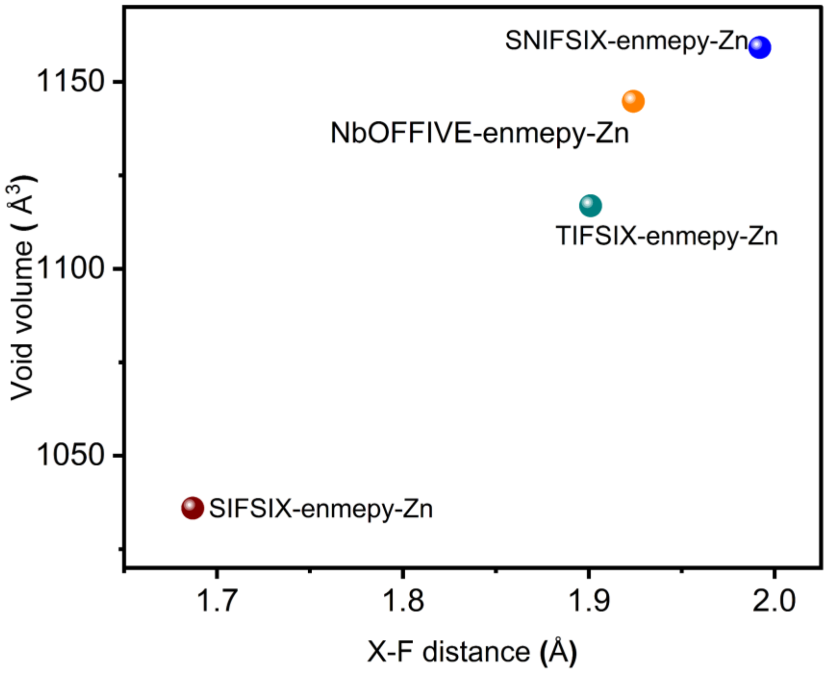

Figure S9: Variation of X–F bond distance with void volume, illustrating how substitution of a single atom alters the overall porosity (X = Si, Ti, Nb, Sn).

Scanning Electron Microscopy (SEM)

Scanning electron microscopy measurements were carried out for the activated samples **TIFSIX-enmepy-Zn** and **NbOFFIVE-enmepy-Zn** to ensure particle size uniformity. The images were captured on a Hitachi SU-70 instrument, using 3 kV acceleration voltage and a working distance of 15 mm. Before the measurement, the samples were dispersed on carbon tape attached to SEM stubs,and were gold-coated for 50 seconds to enhance surface conductivity.

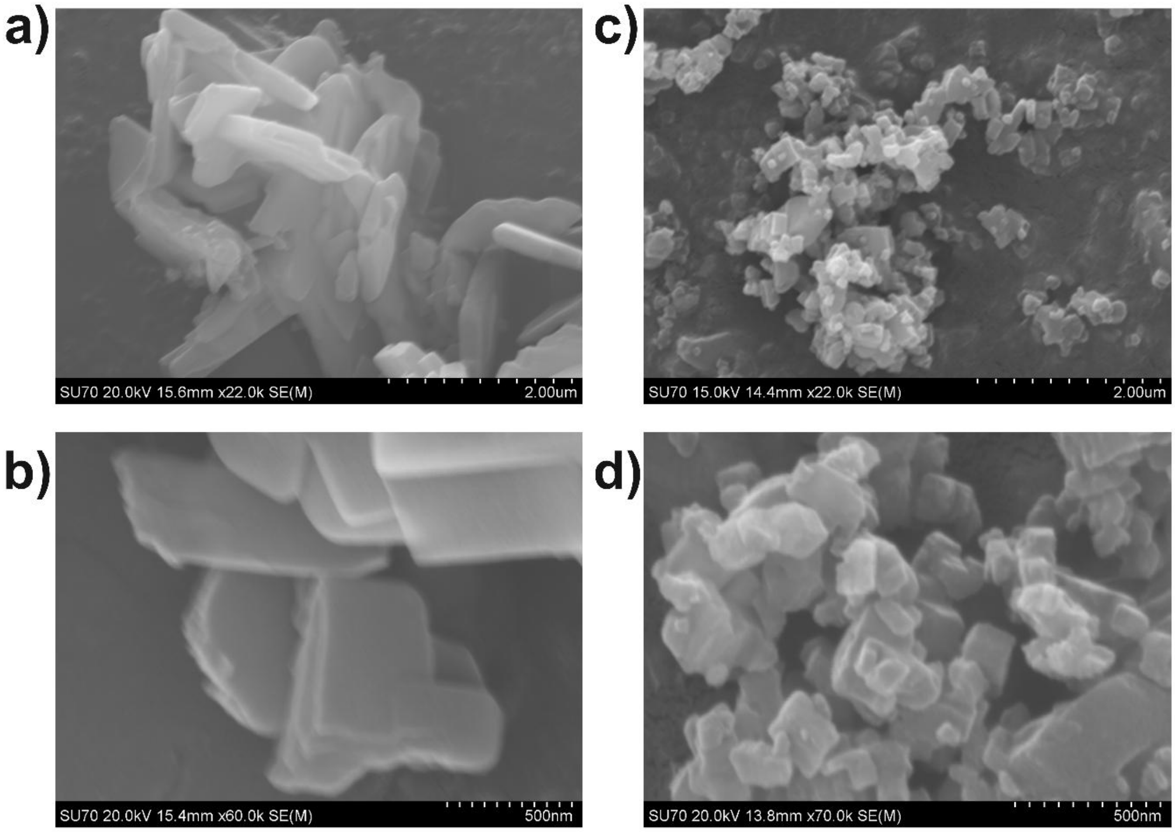

Figure S10: SEM images of chelating HUMs. (a, b) **TIFSIX-enmepy-Zn** showing block-like crystals with well-defined facets. (c, d) **NbOFFIVE-enmepy-Zn** displaying smaller, more aggregated crystallites. Low-magnification images (a, c) highlight overall morphology, while higher-magnification images (b, d) reveal surface features.

Particle size analysis

Particle size distributions were determined using a Morphologi G3 automated imaging system (Malvern Panalytical). Measurements were performed on dry, as-synthesised powder samples of **TIFSIX-enmepy-Zn** and **NbOFFIVE-enmepy-Zn**. Prior to analysis, powders were gently dispersed onto glass slides using a controlled dry powder disperser to minimise agglomeration. Images of individual particles were captured under bright-field illumination, and size distributions were generated based on equivalent circular diameter (ECD). Gaussian fitting of the distributions was carried out using the Origin software package. Reported size ranges may include both primary crystallites and aggregates. The same batches of samples were subsequently employed in gas sorption and other experiments (unless mentioned otherwise) including dynamic breakthrough experiments.

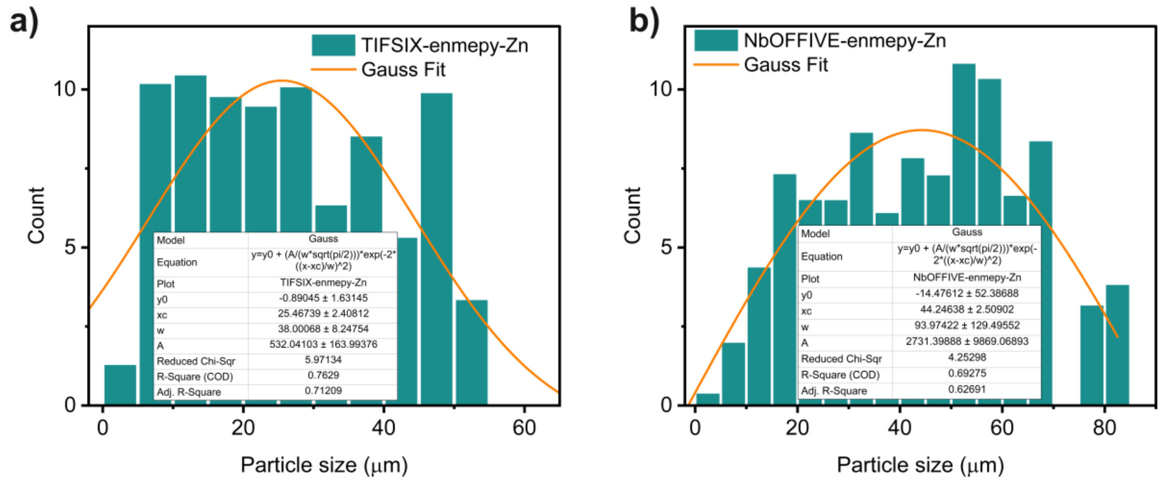

Figure S11: Particle size distributions of as-synthesised HUM powders measured using a Morphologi G3 instrument under dry conditions. (a) **TIFSIX-enmepy-Zn** and (b) **NbOFFIVE-enmepy-Zn**, with Gaussian fits applied to the distributions. The observed size ranges likely reflect not only primary crystallites but also aggregates of particles.

Powder X-ray diffraction (PXRD)

Diffractograms were recorded using a PANalytical Empyrean™ diffractometer equipped with a PIXcel-1D detector operating in scanning line detector mode with an active length of 3.3473 degrees utilising 255 channels. The diffractometer is outfitted with an Empyrean Cu LFF (long fine-focus) HR (9430 033 7310x) tube operated at 40 kV and 40 mA and CuK$_{\alpha}$ radiation (λ_{α} = 1.540598 Å) was used for diffraction experiments. Continuous scanning mode with the goniometer in the theta-theta orientation was used to collect the data. Incident beam optics included the Fixed Divergences slit with anti-scatter slit PreFIX module, with a 1/8° divergence slit and a 1/4° anti-scatter slit, as well as a 10 mm fixed incident beam mask and a Soller slit (0.04 rad). Divergent beam optics included a P7.5 anti-scatter slit, a Soller slit (0.04 rad), and a Ni-*β* filter. In a typical experiment, ≈20 mg of sample was dried, ground into a fine powder, and loaded on a zero background silicon disks. The data was collected from 4–40° (2*θ*) with a step-size of 0.0131303° and a scan time of 30.6 s per step (°/2*θ*). Crude data were analysed using the X'Pert HighScore Plus™ software V 4.1 (PANalytical, The Netherlands)

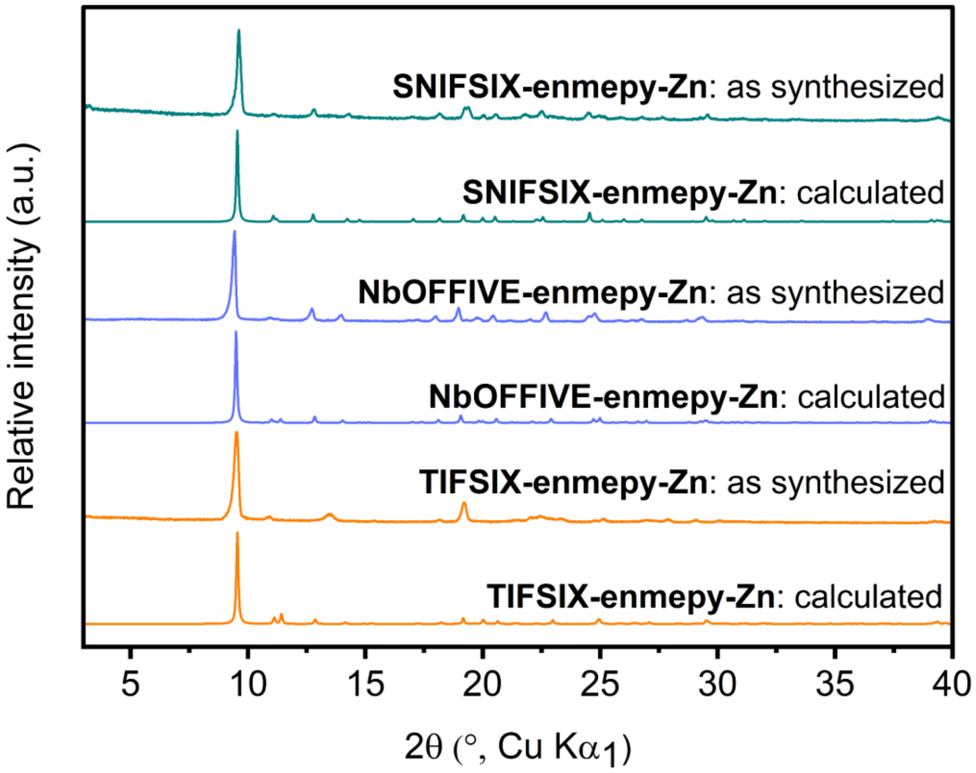

Figure S12: PXRD patterns recorded for the as synthesized bulk samples of **TIFSIX-enmepy-Zn**, **NbOFFIVE-enmepy-Zn** and **SNIFSIX-enmepy-Zn**, compared to the calculated PXRD patterns.

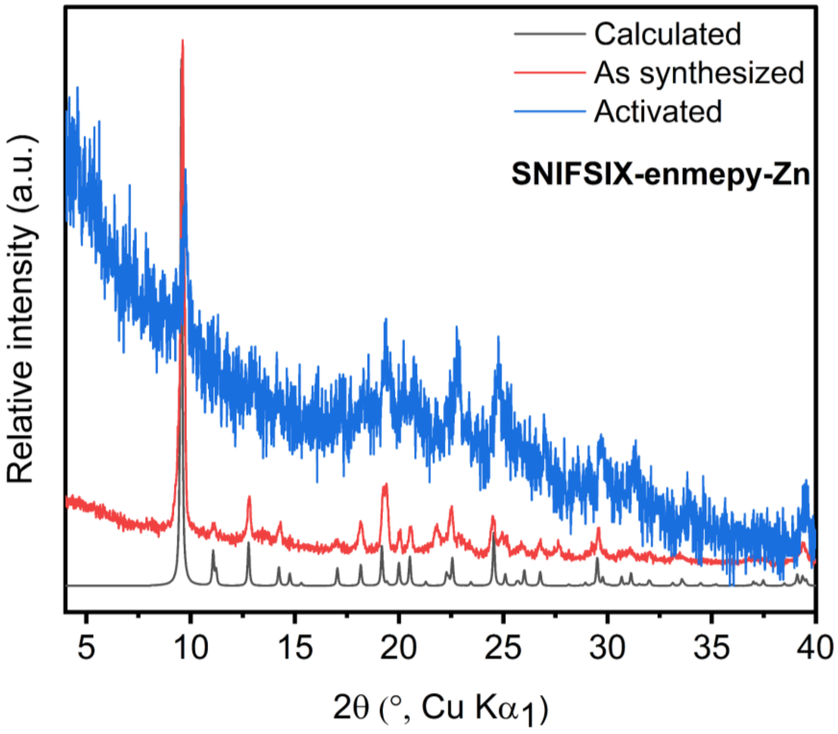

Figure S13: PXRD patterns for **SNIFSIX-enmepy-Zn**: calculated in black, as-synthesised bulk in red, and after activation in blue.

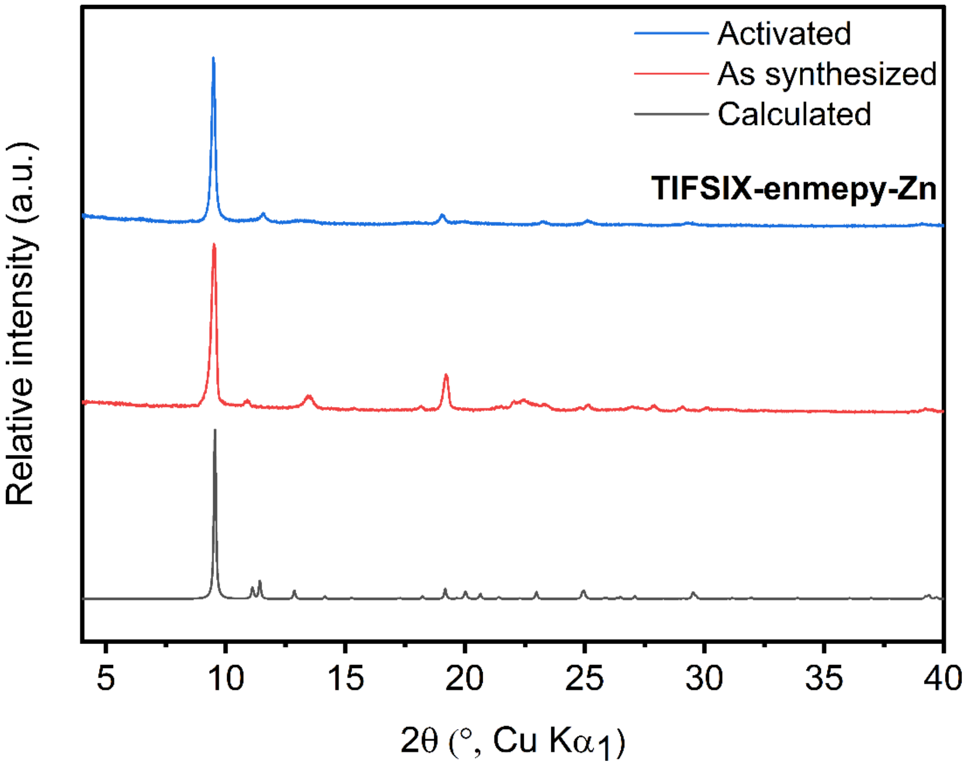

Figure S14: PXRD patterns for **TIFSIX-enmepy-Zn**: calculated in black, as-synthesised bulk in red, and after activation in blue.

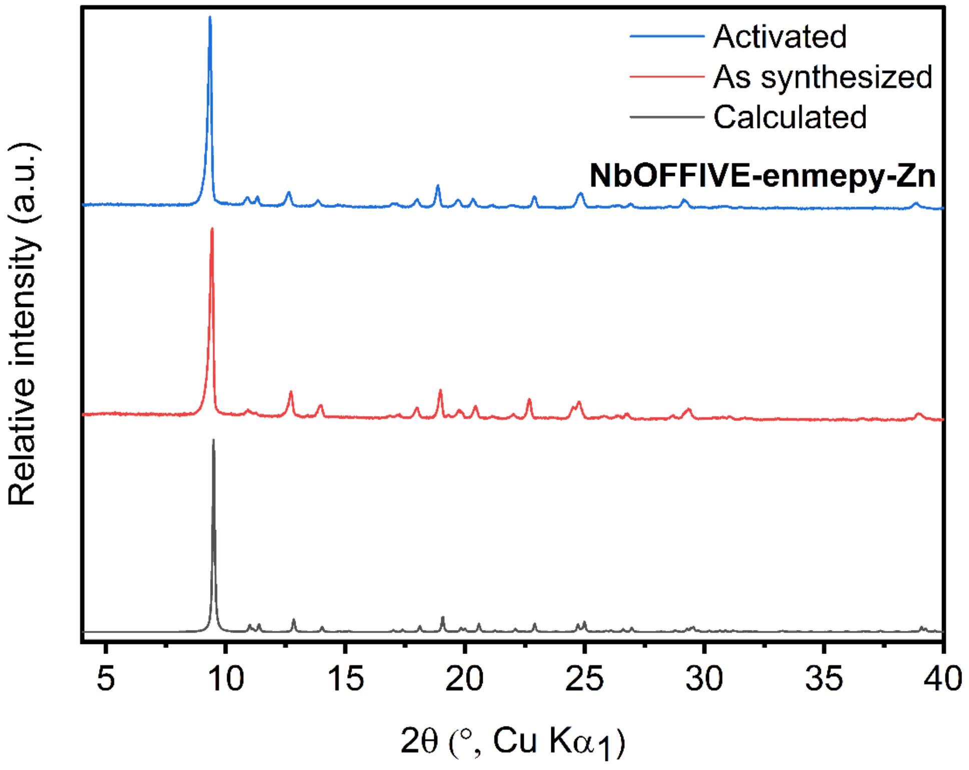

Figure S15: PXRD patterns for **NbOFFIVE-enmepy-Zn**: calculated in black, as-synthesised bulk in red, and after activation in blue.

Thermogravimetric analysis (TGA)

Thermograms were recorded under nitrogen using TGA instrument TA Q50 V20.13 Build 39. Platinum pans and a flow rate of 60 cm^3 min^{-1} for the nitrogen gas were used for the experiments. The data was recorded in the High-Resolution Dynamic mode with a sensitivity of 1.0, a resolution of 4.0, and a temperature ramp of 10 K min^{-1} up to 773 K. The data was evaluated using the T.A. Universal Analysis suite.

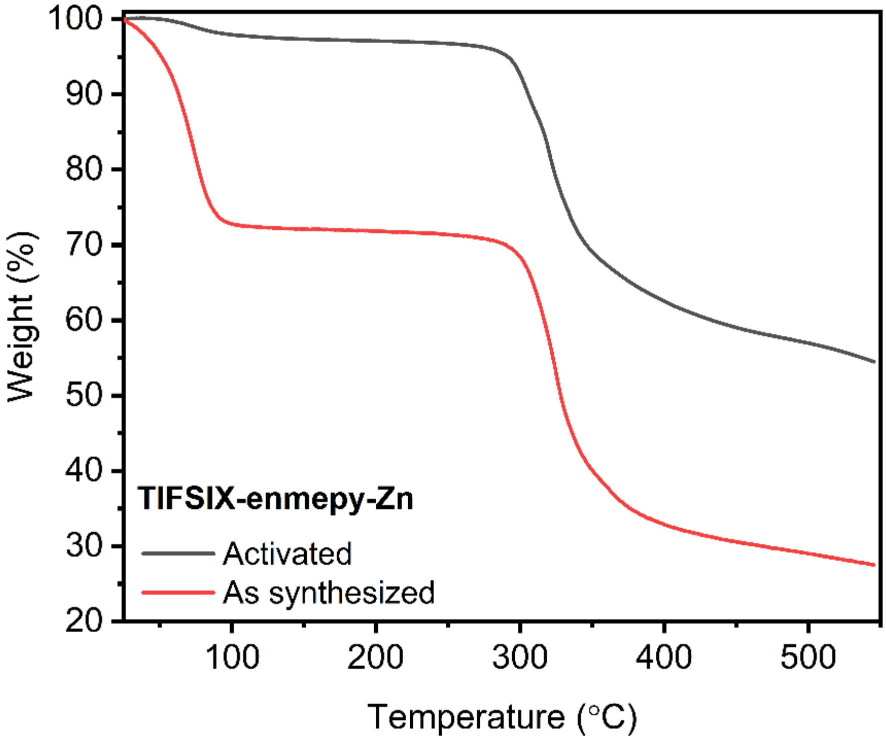

Figure S16: Thermogravimetric analysis traces for **TIFSIX-enmepy-Zn**: as-synthesised (in red) and activated phase (in black).

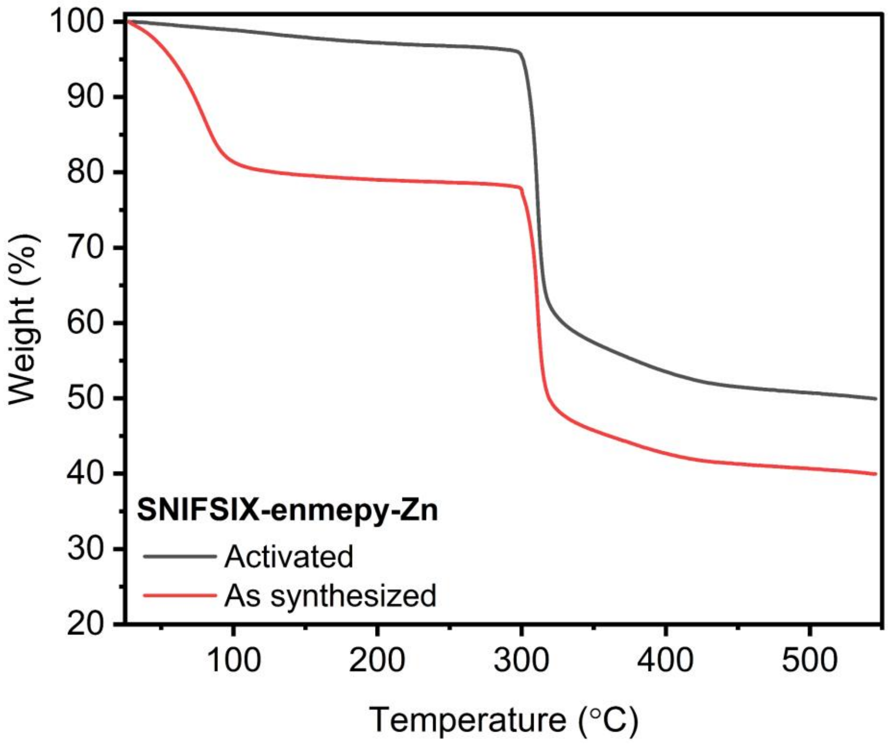

Figure S17: Thermogravimetric analysis traces for **SNIFSIX-enmepy-Zn**: as-synthesised (in red) and activated phase (in black).

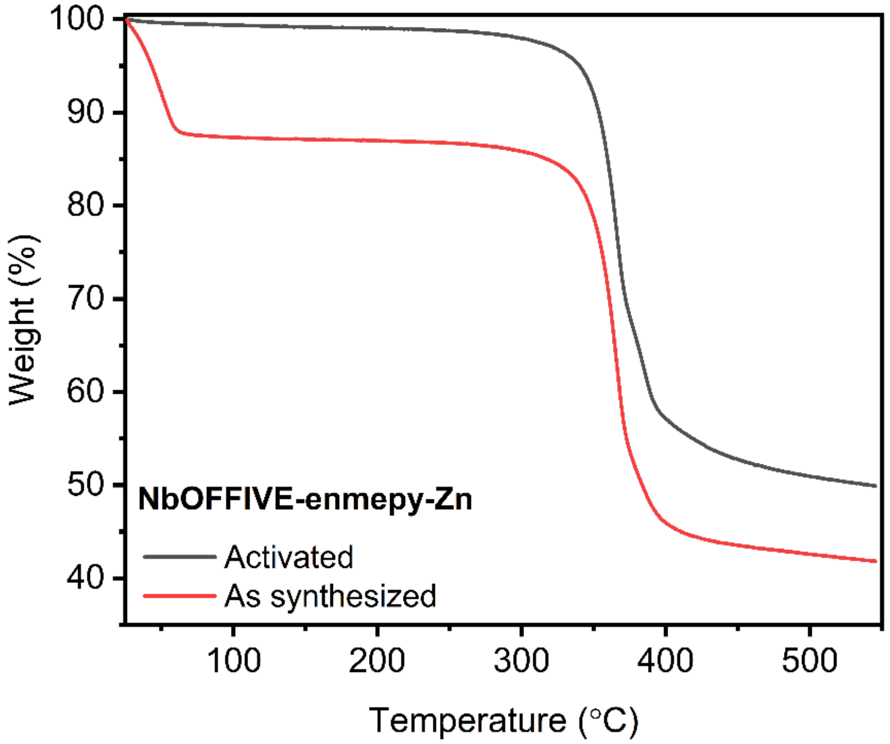

Figure S18: Thermogravimetric analysis traces for **NbOFFIVE-enmepy-Zn**: as-synthesised (in red) and activated phase (in black).

Variable-Temperature Powder X-ray Diffraction (VT PXRD)

Diffractograms at different temperatures were recorded using a Anton Paar XRDynamic 500: TTK 600 diffractometer equipped with a PIXcel3D detector operating in scanning line detector mode. Anton Paar TTK 450 stage coupled with the Anton Paar TCU 110 Temperature Control Unit was used to record the variable temperature diffractograms. Continuous scanning mode with the goniometer in the theta-theta orientation was used to collect the data. Incident beam optics included the Fixed Divergences slit, with a 1/4° divergence slit and a Soller slit (0.04 rad). Divergent beam optics included a P7.5 S7 anti-scatter slit, a Soller slit (0.04 rad), and a Ni-β filter. In a typical experiment, ≈50 mg of the sample was loaded on a zero-background disc made for Anton Paar TTK 600 chamber. The data was recorded between 4° and 40° (2θ) with a step-size of 0.0167113°, and a scan time of 200 seconds per step. Crude diffractograms were analysed using the X'Pert HighScore Plus9 ™ software V 4.1 (PANalytical, The Netherlands).

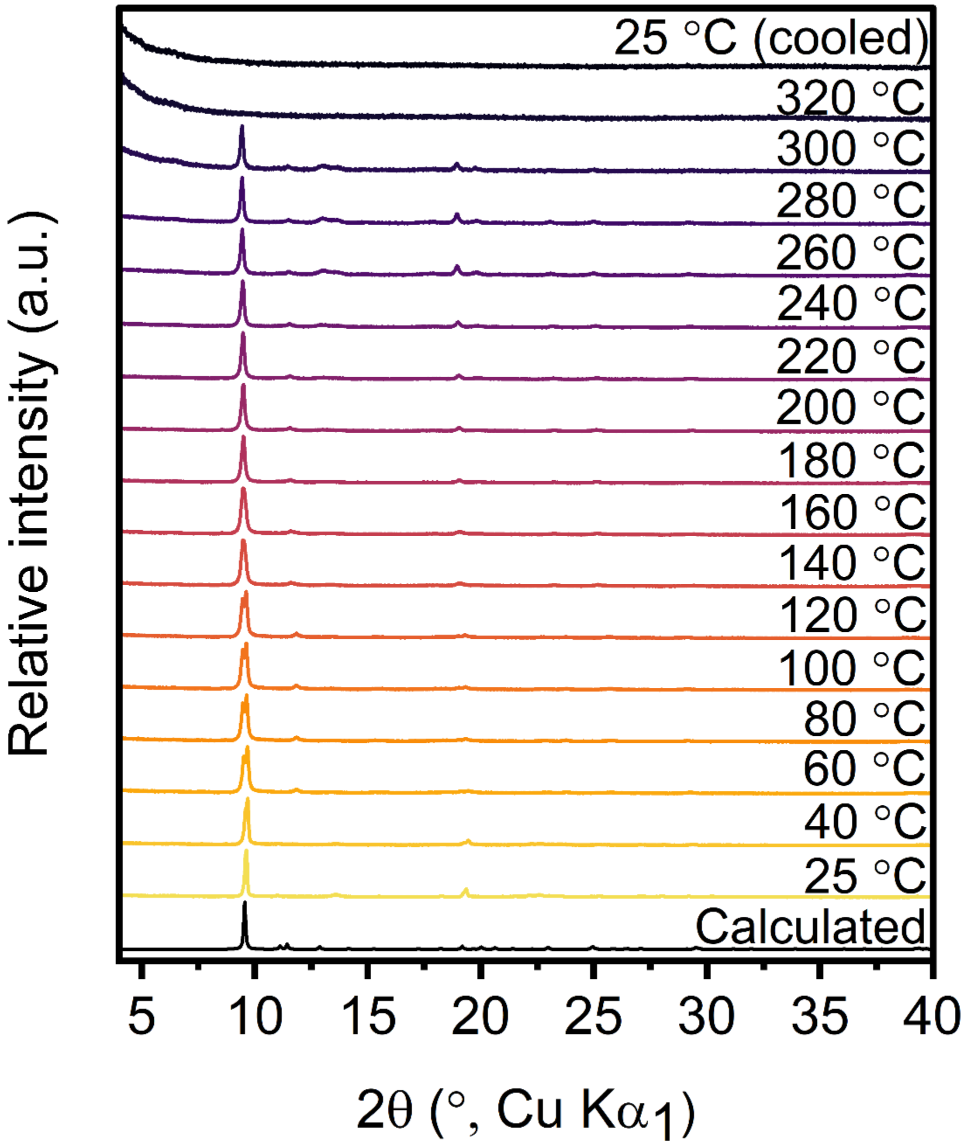

Figure S19: *In-situ* variable-temperature PXRD patterns of as synthesized **TIFSIX-enmepy-Zn** recorded under N_2 flow.

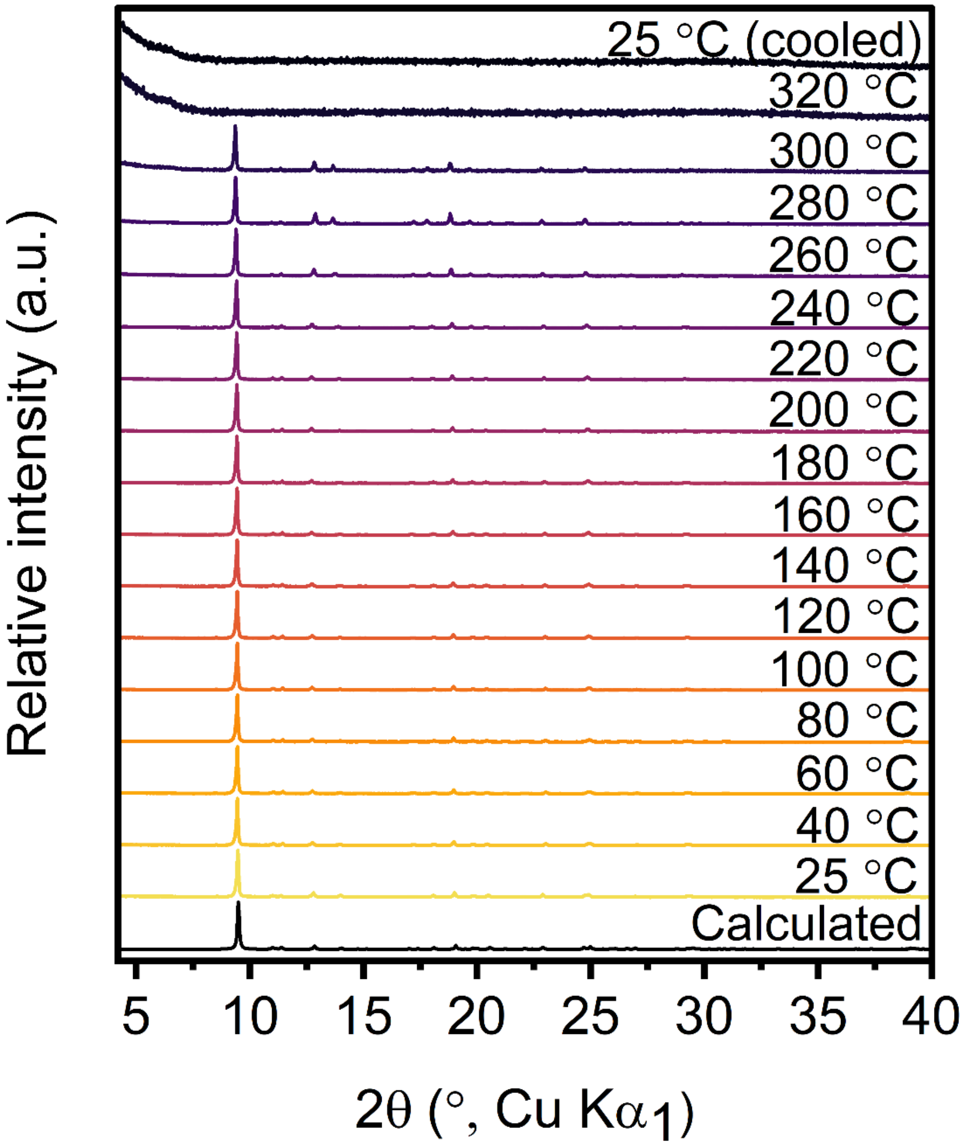

Figure S20: *In-situ* variable-temperature PXRD patterns of as synthesized **NbOFFIVE-enmepy-Zn,** recorded under N_2 flow.

Single-component gas sorption experiments

The methanol exchanged polymers were activated under high vacuum by a Micromeritics® Smart VacPrepTM at 80 °C for 12 h before gas sorption studies. The sorption isotherms for CO_2 at 195 K were measured using a Micromeritics® 3Flex adsorption analyser. The 195 K environment was maintained by a 2.75 L Dewar flask containing a bath of acetone and dry ice. Brunauer–Emmett–Teller (BET) surface areas were determined from the adsorption isotherms.[212–213] The pore size distribution curves were obtained from the adsorption branches using Horvath-Kawazoe (H-K) method.

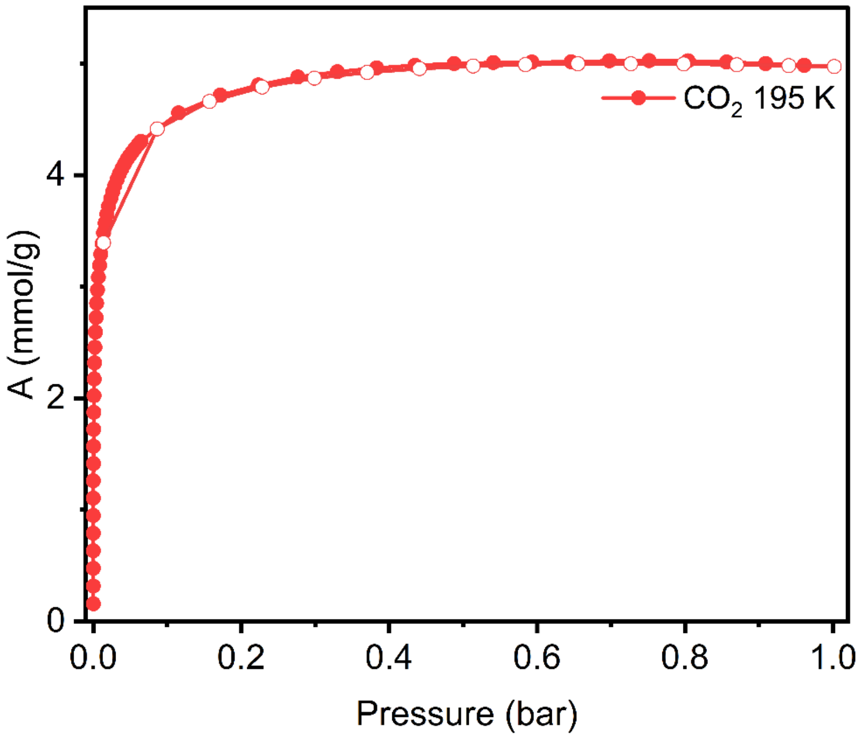

Figure S21: CO_2 isotherm for **TIFSIX-enmepy-Zn** at 195 K (filled symbols: adsorption; open symbols: desorption).

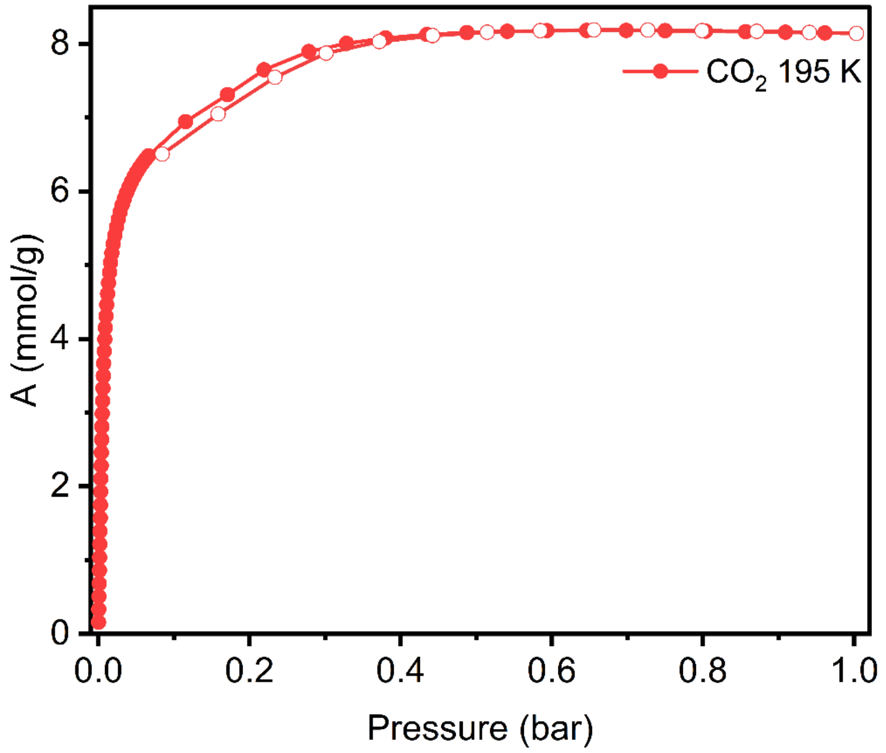

Figure S22: CO_2 isotherm for **NbOFFIVE-enmepy-Zn** at 195 K (filled symbols: adsorption; open symbols: desorption).

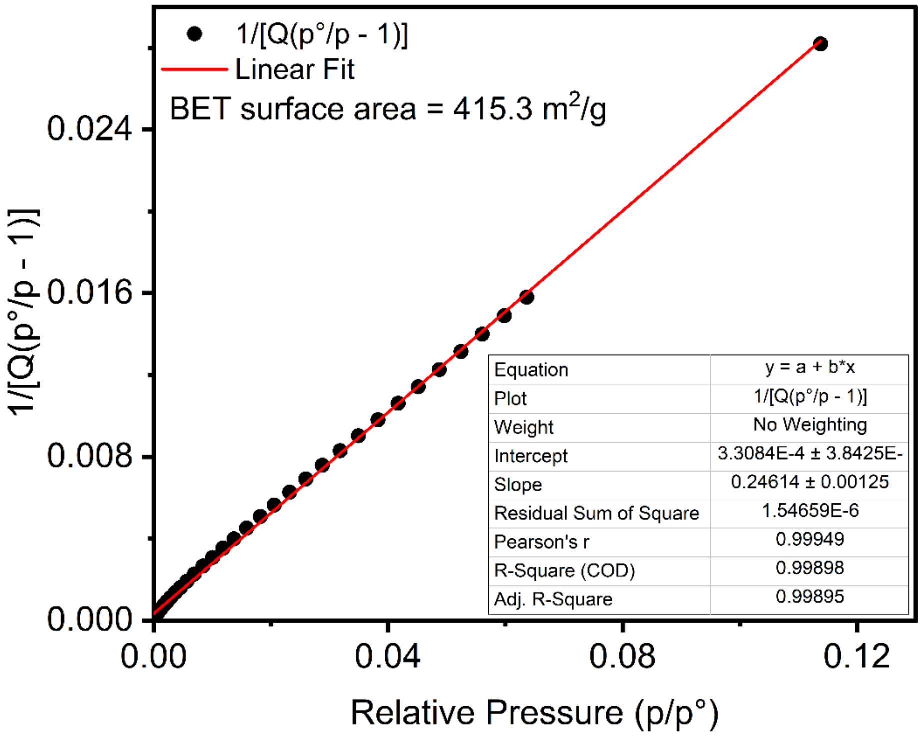

Figure S23: BET surface area of **TIFSIX-enmepy-Zn** determined from its CO_2 adsorption isotherm at 195 K.

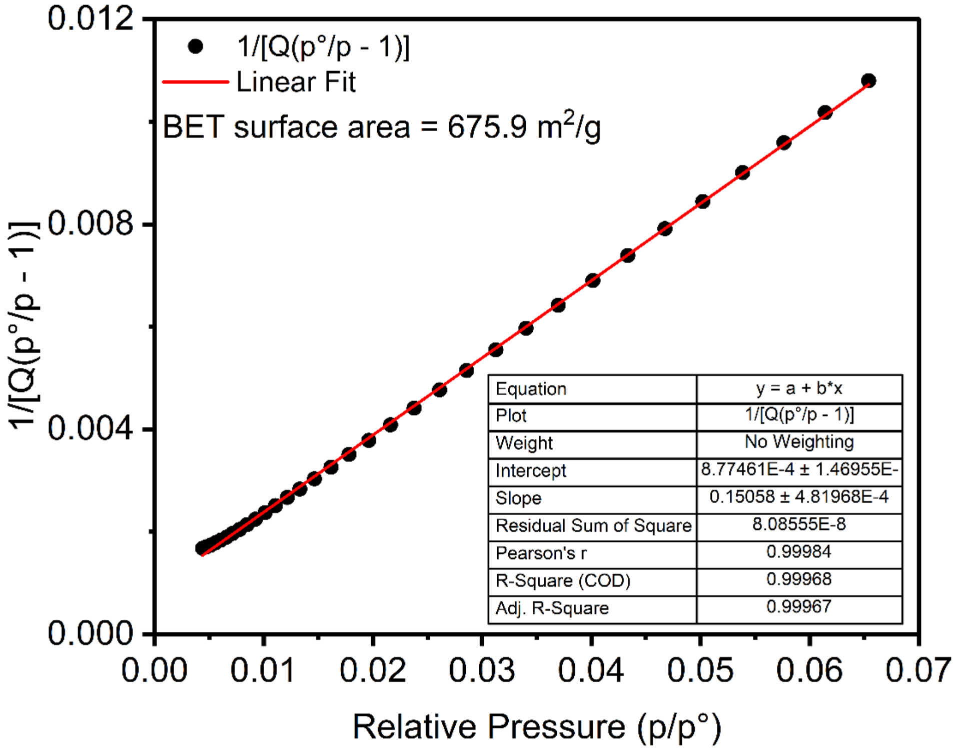

Figure S24: BET surface area of **NbOFFIVE-enmepy-Zn** determined from its CO_2 adsorption isotherm at 195 K.

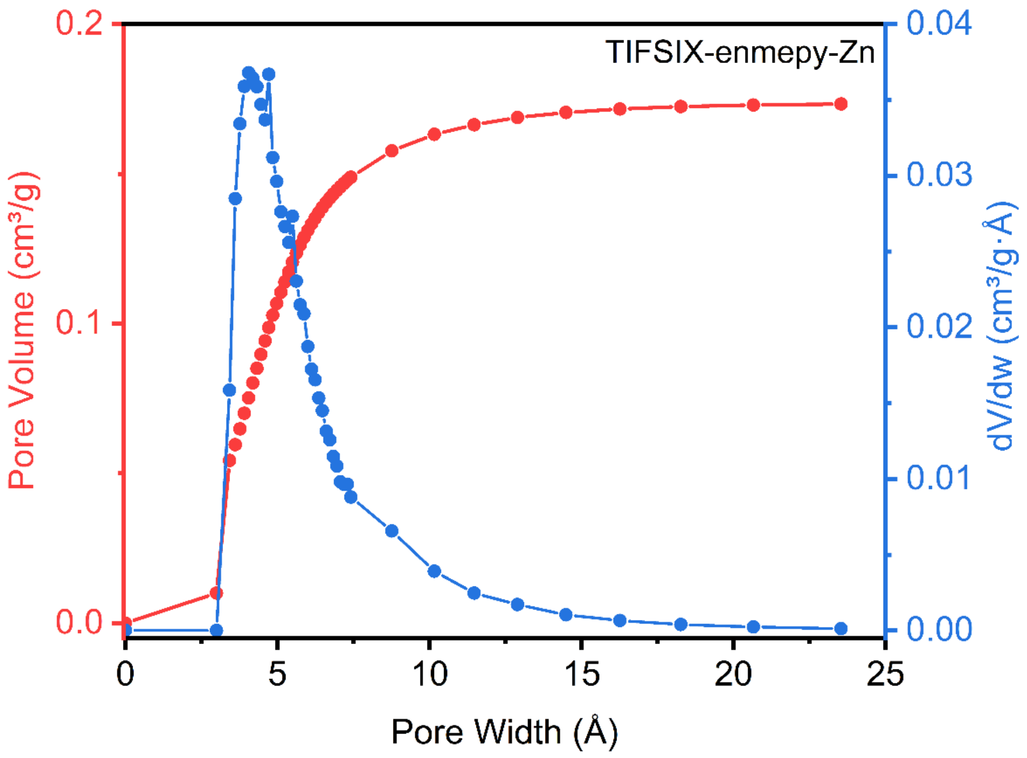

Figure S25: Pore size distribution profile for **TIFSIX-enmepy-Zn**, based on Horvath-Kawazoe model using slit pore geometry fit of its CO_2 adsorption isotherm at 195 K.

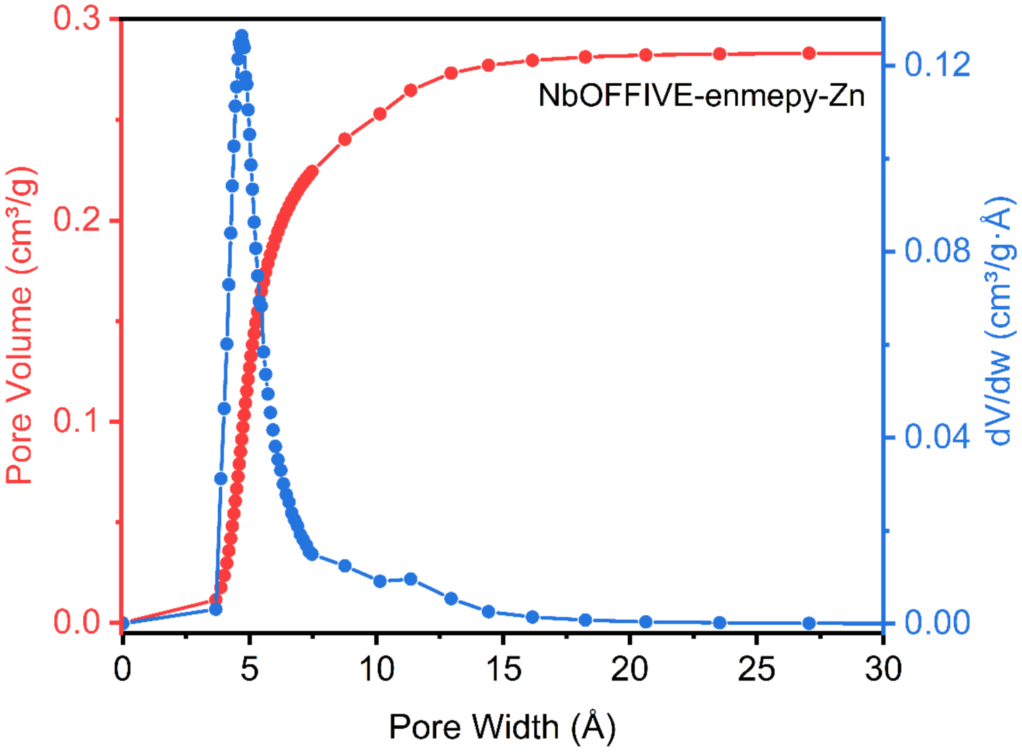

Figure S26: Pore size distribution profile for **NbOFFIVE-enmepy-Zn**, based on Horvath-Kawazoe model using slit pore geometry fit of its CO_2 adsorption isotherm at 195 K.

Adsorption selectivity calculations.

The selectivities for the adsorbate mixture composition of interest were calculated from the single component adsorption isotherms using Ideal Adsorbed Solution Theory (IAST), using IAST++ program.[214] First, the single-component isotherms for the gas sorbates at 298 K were fitted to the dual-site Langmuir (DSL) equation.

$$n(P) = \frac{q_1(k_1P)}{1+(k_1P)} + \frac{q_2(k_2P)}{1+(k_2P)}$$

Here, p is the pressure of the bulk gas at equilibrium with the adsorbed phase (Pa), q is the adsorbed amount per mass of adsorbent (molkg^{-1}), q_A an q_B are the saturation capacities of site A and B (mol kg-1), b_A and b_B are the affinity coefficients of site A and B (P_a^{-1}), v_A and v_B represent the deviations from an ideal homogeneous surface.
The selectivity for C_2H_2/CO_2 separation was calculated using the following equation:

$$S_{ads} = \frac{\frac{q_1}{q_2}}{\frac{p_1}{p_2}}$$

where q_1 and q_2 are the molar loadings in the adsorbed phase in equilibrium with the bulk gas phase with partial pressures p1 and p2.

Table S5: Isotherm fitting parameters and fit R^2 values for IAST calculations for **TIFSIX-enmepy-Zn**.

Adsorbate	Model	R^2	q_1(mmol g^{-1})	q_2(mmol g^{-1})	k_1(bar^{-1})	k_2(bar^{-1})
**C_2H_2**	Dual-site Langmuir	0.99992	1.45719	2.81374	64.23926	1.60793
**CO_2**	Dual-site Langmuir	1	2.2482	1.52723	1.14946	7.35800

Table S6: Isotherm fitting parameters and fit R^2 values for IAST calculations for **NbOFFIVE-emepy-Zn**.

Adsorbate	Model	R^2	q_1(mmol g^{-1})	q_2(mmol g^{-1})	k_1(bar^{-1})	k_2(bar^{-1})
**C_2H_2**	Dual-site Langmuir	0.99998	4.16611	287.19661	3.93762	0.00108
**CO_2**	Dual-site Langmuir	0.99999	3.40299	3.40299	0.34401	0.34401

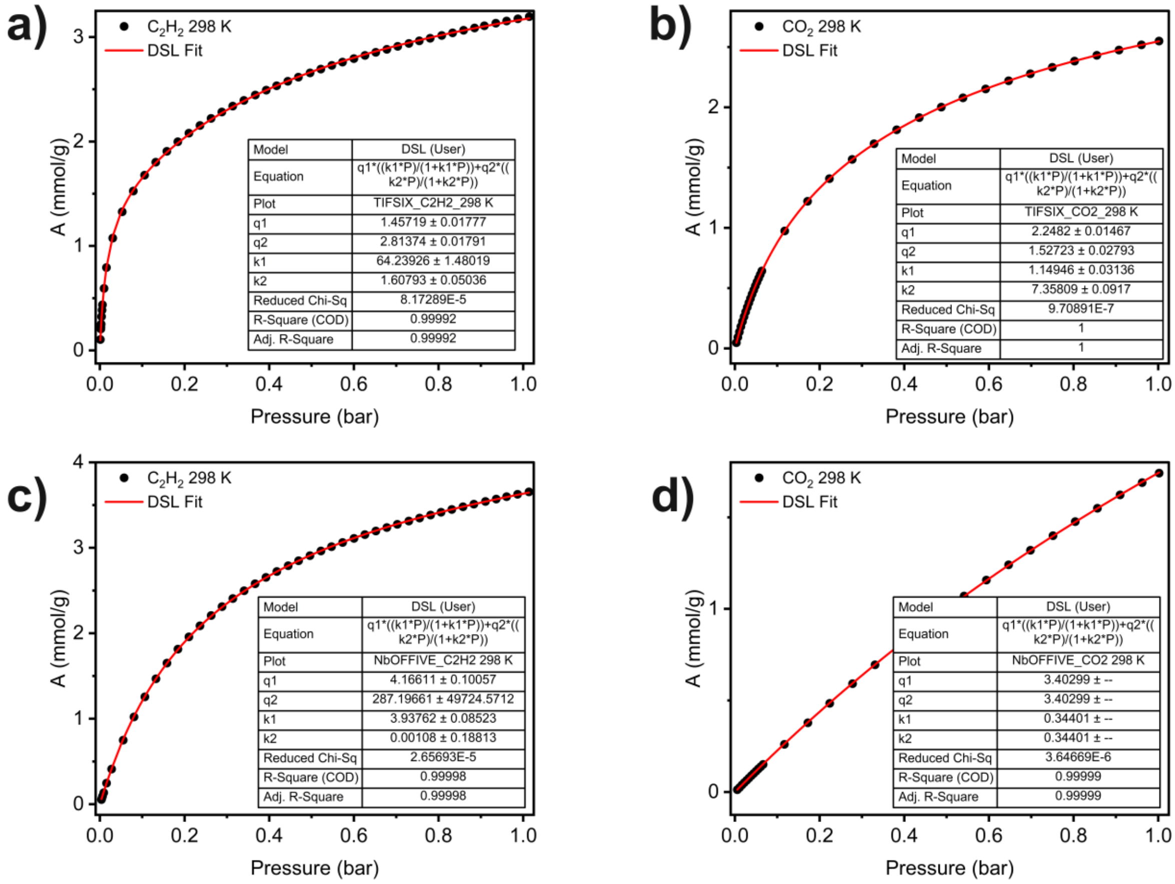

Figure S27: Dual-site Langmuir (DSL) fits of single-component adsorption isotherms at 298 K for (a) C_2H_2 on **TIFSIX-enmepy-Zn**, (b) CO_2 on **TIFSIX-enmepy-Zn**, (c) C_2H_2 on **NbOFFIVE-enmepy-Zn**, and (d) CO_2 on **NbOFFIVE-enmepy-Zn**. Experimental data are shown as black symbols, with DSL fits shown as red lines. The fitted parameters are listed in the insets. These models were used for IAST calculations of C_2H_2/CO_2 selectivity.

Adsorption energy calculations.

Isosteric heat of adsorption (Q_{st}) values were calculated from isotherms measured at 273 K, 283 K and 298 K for C_2H_2 and CO_2 on the activated samples. The sorption isotherms were measured using a Micromeritics® 3Flex adsorption analyser. 273 K, 283 K and 298 K environment was controlled by a Julabo temperature controller with an ethylene glycol/water = 1:1 (v/v) mixture. The isotherms were first fit to a viral equation:

$$lnP = lnN + \frac{1}{T}\sum_{i=0}^{mn} a_i N^i + \sum_{i=0}^{n} b_i N^i$$

Where N is the amount of gas adsorbed at the pressure P, a and b are virial coefficients, m and n are the number of coefficients require to adequately describe the isotherm. To calculate Q_{st}, the fitting parameters from the above equation were used for the following equation:

$$Q_{st} = -R\sum_{i=0}^{mn} a_i N^i$$

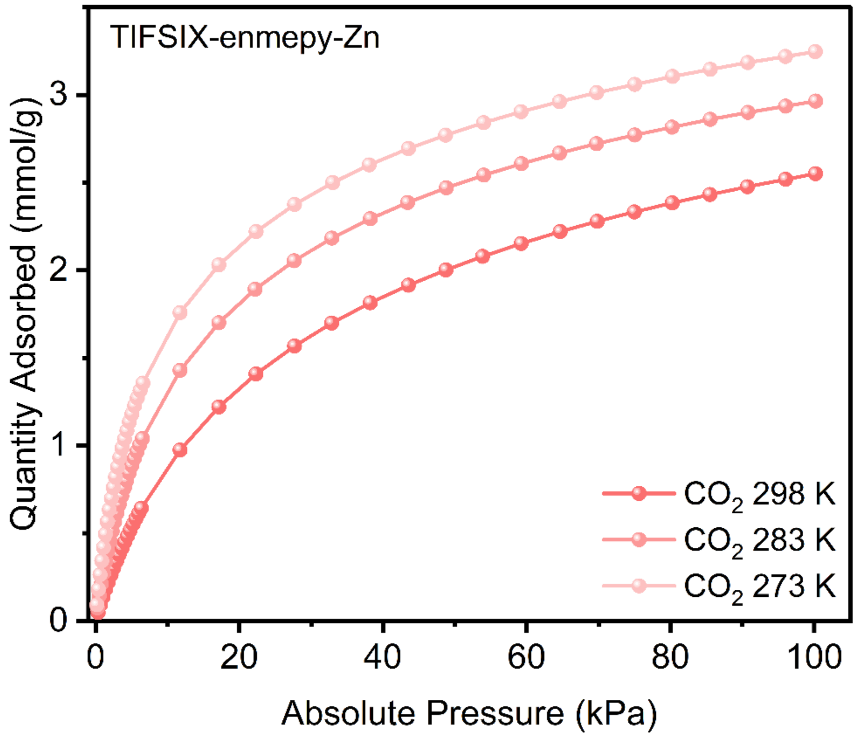

Figure S28: CO_2 adsorption isotherms recorded at different temperatures for **TIFSIX-enmepy-Zn**.

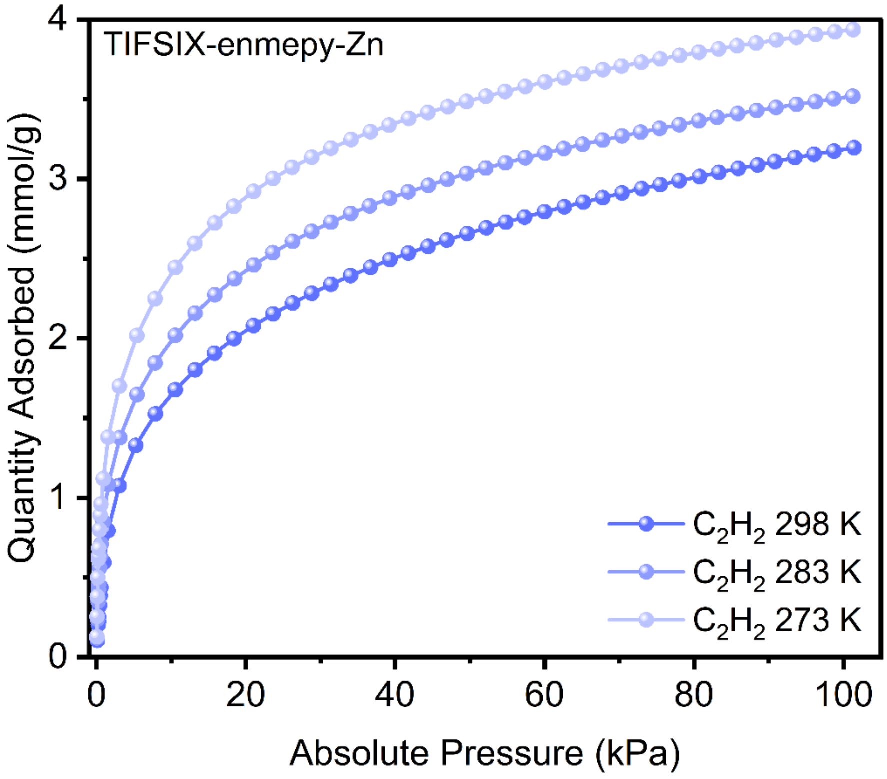

Figure S29: C_2H_2 adsorption isotherms recorded at different temperatures for **TIFSIX-enmepy-Zn**.

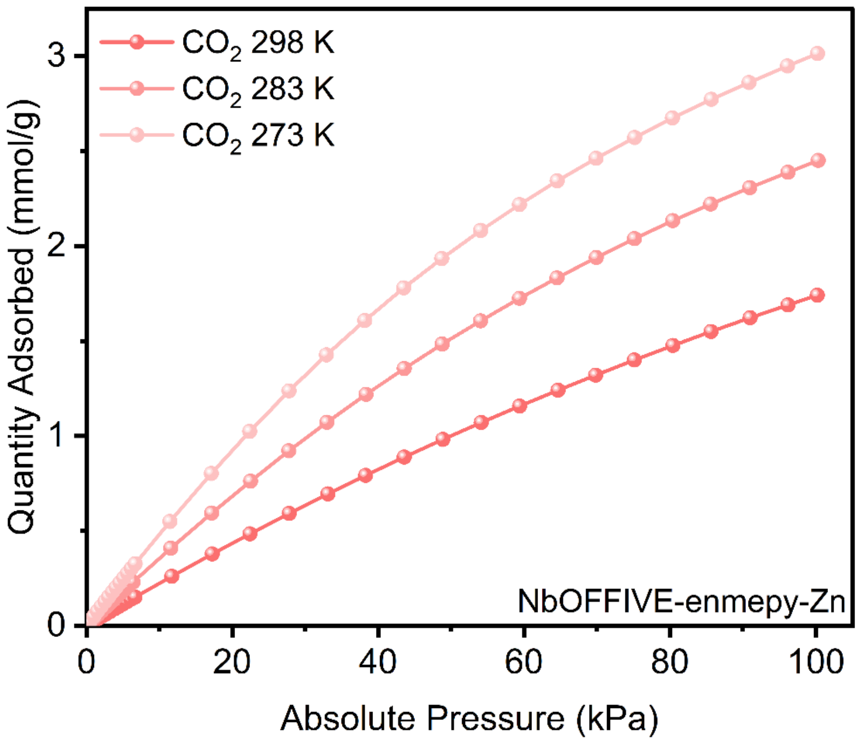

Figure S30: CO_2 adsorption isotherms recorded at different temperatures for **NbOFFIVE-enmepy-Zn**.

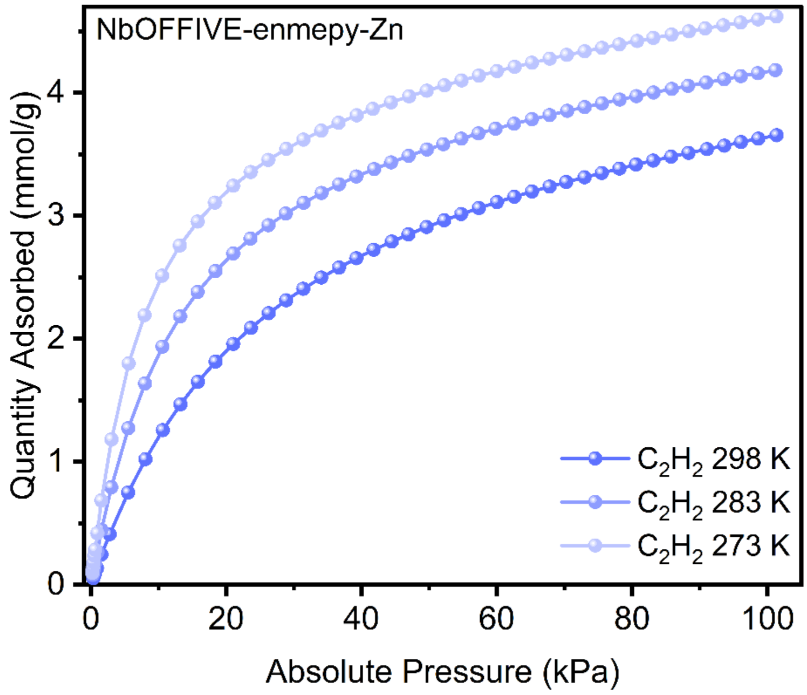

Figure S31: C_2H_2 adsorption isotherms recorded at different temperatures for **NbOFFIVE-enmepy-Zn**.

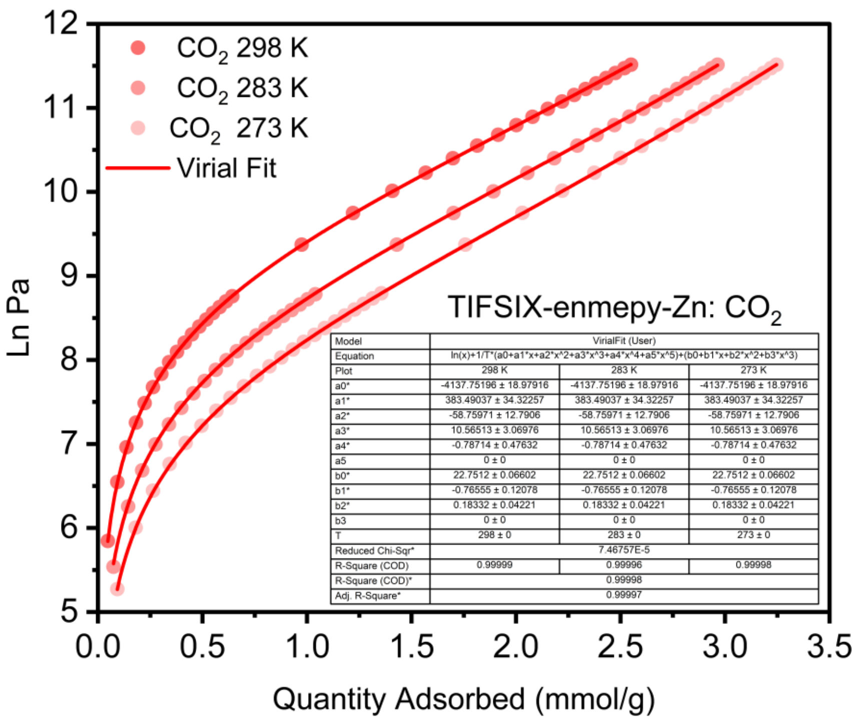

Model	VirialFit (User)		
Equation	ln(x)+1/T*(a0+a1*x+a2*x^2+a3*x^3+a4*x^4+a5*x^5)+(b0+b1*x+b2*x^2+b3*x^3)		
Plot	298 K	283 K	273 K
a0*	-4137.75196 ± 18.97916	-4137.75196 ± 18.97916	-4137.75196 ± 18.97916
a1*	383.49037 ± 34.32257	383.49037 ± 34.32257	383.49037 ± 34.32257
a2*	-58.75971 ± 12.7906	-58.75971 ± 12.7906	-58.75971 ± 12.7906
a3*	10.56513 ± 3.06976	10.56513 ± 3.06976	10.56513 ± 3.06976
a4*	-0.78714 ± 0.47632	-0.78714 ± 0.47632	-0.78714 ± 0.47632
a5	0 ± 0	0 ± 0	0 ± 0
b0*	22.7512 ± 0.06602	22.7512 ± 0.06602	22.7512 ± 0.06602
b1*	-0.76555 ± 0.12078	-0.76555 ± 0.12078	-0.76555 ± 0.12078
b2*	0.18332 ± 0.04221	0.18332 ± 0.04221	0.18332 ± 0.04221
b3	0 ± 0	0 ± 0	0 ± 0
T	298 ± 0	283 ± 0	273 ± 0
Reduced Chi-Sqr*	7.46757E-5		
R-Square (COD)	0.99999	0.99996	0.99998
R-Square (COD)*	0.99998		
Adj. R-Square*	0.99997		

Figure S32: Virial fits and parameters used for Q_{st} calculations for; **TIFSIX-enmepy-Zn** for CO_2.

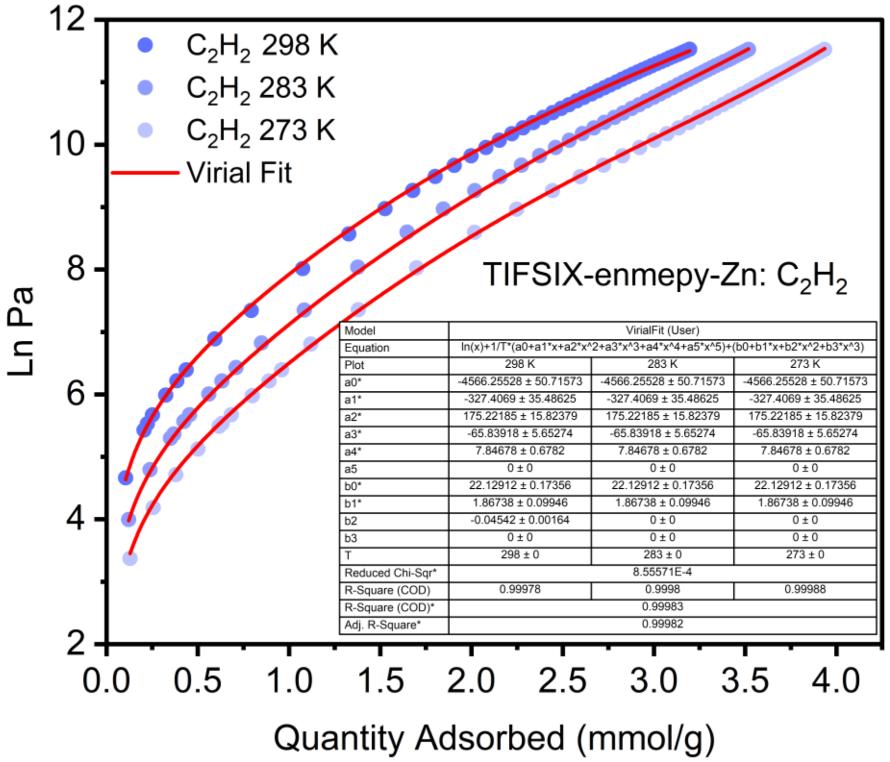

Model	VirialFit (User)		
Equation	ln(x)+1/T*(a0+a1*x+a2*x^2+a3*x^3+a4*x^4+a5*x^5)+(b0+b1*x+b2*x^2+b3*x^3)		
Plot	298 K	283 K	273 K
a0*	-4566.25528 ± 50.71573	-4566.25528 ± 50.71573	-4566.25528 ± 50.71573
a1*	-327.4069 ± 35.48625	-327.4069 ± 35.48625	-327.4069 ± 35.48625
a2*	175.22185 ± 15.82379	175.22185 ± 15.82379	175.22185 ± 15.82379
a3*	-65.83918 ± 5.65274	-65.83918 ± 5.65274	-65.83918 ± 5.65274
a4*	7.84678 ± 0.6782	7.84678 ± 0.6782	7.84678 ± 0.6782
a5	0 ± 0	0 ± 0	0 ± 0
b0*	22.12912 ± 0.17356	22.12912 ± 0.17356	22.12912 ± 0.17356
b1*	1.86738 ± 0.09946	1.86738 ± 0.09946	1.86738 ± 0.09946
b2	-0.04542 ± 0.00164	0 ± 0	0 ± 0
b3	0 ± 0	0 ± 0	0 ± 0
T	298 ± 0	283 ± 0	273 ± 0
Reduced Chi-Sqr*	8.55571E-4		
R-Square (COD)	0.99978	0.9998	0.99988
R-Square (COD)*	0.99983		
Adj. R-Square*	0.99982		

Figure S33: Virial fits and parameters used for Q_{st} calculations for; **TIFSIX-enmepy-Zn** for C_2H_2.

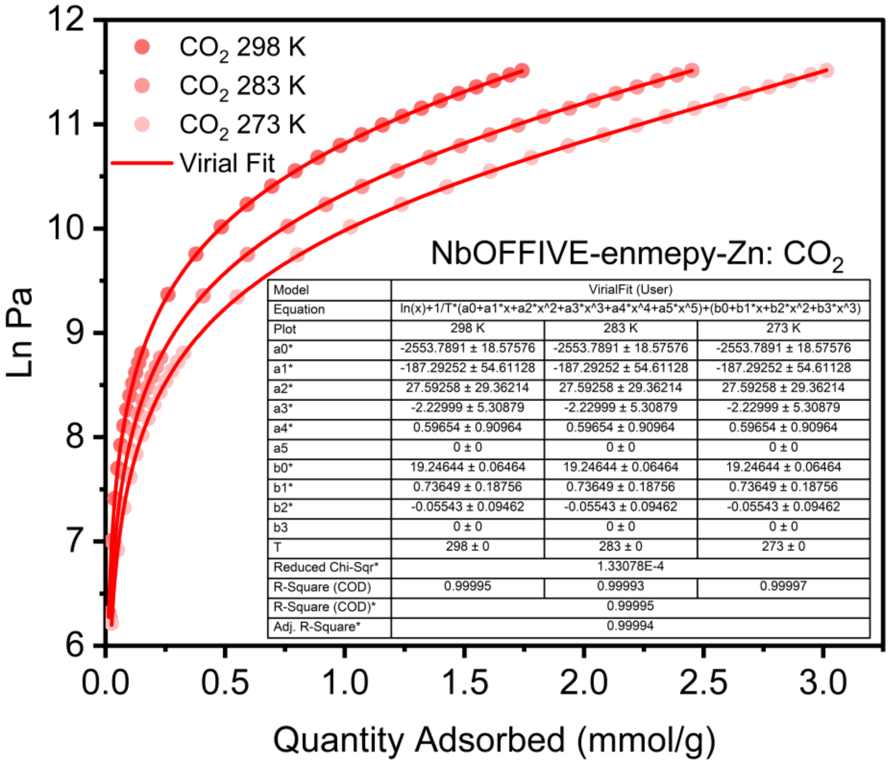

Model	VirialFit (User)		
Equation	ln(x)+1/T*(a0+a1*x+a2*x^2+a3*x^3+a4*x^4+a5*x^5)+(b0+b1*x+b2*x^2+b3*x^3)		
Plot	298 K	283 K	273 K
a0*	-2553.7891 ± 18.57576	-2553.7891 ± 18.57576	-2553.7891 ± 18.57576
a1*	-187.29252 ± 54.61128	-187.29252 ± 54.61128	-187.29252 ± 54.61128
a2*	27.59258 ± 29.36214	27.59258 ± 29.36214	27.59258 ± 29.36214
a3*	-2.22999 ± 5.30879	-2.22999 ± 5.30879	-2.22999 ± 5.30879
a4*	0.59654 ± 0.90964	0.59654 ± 0.90964	0.59654 ± 0.90964
a5	0 ± 0	0 ± 0	0 ± 0
b0*	19.24644 ± 0.06464	19.24644 ± 0.06464	19.24644 ± 0.06464
b1*	0.73649 ± 0.18756	0.73649 ± 0.18756	0.73649 ± 0.18756
b2*	-0.05543 ± 0.09462	-0.05543 ± 0.09462	-0.05543 ± 0.09462
b3	0 ± 0	0 ± 0	0 ± 0
T	298 ± 0	283 ± 0	273 ± 0
Reduced Chi-Sqr*	1.33078E-4		
R-Square (COD)	0.99995	0.99993	0.99997
R-Square (COD)*	0.99995		
Adj. R-Square*	0.99994		

Figure S34: Virial fits and parameters used for Q_{st} calculations for; **NbOFFIVE-enmepy-Zn** for CO_2.

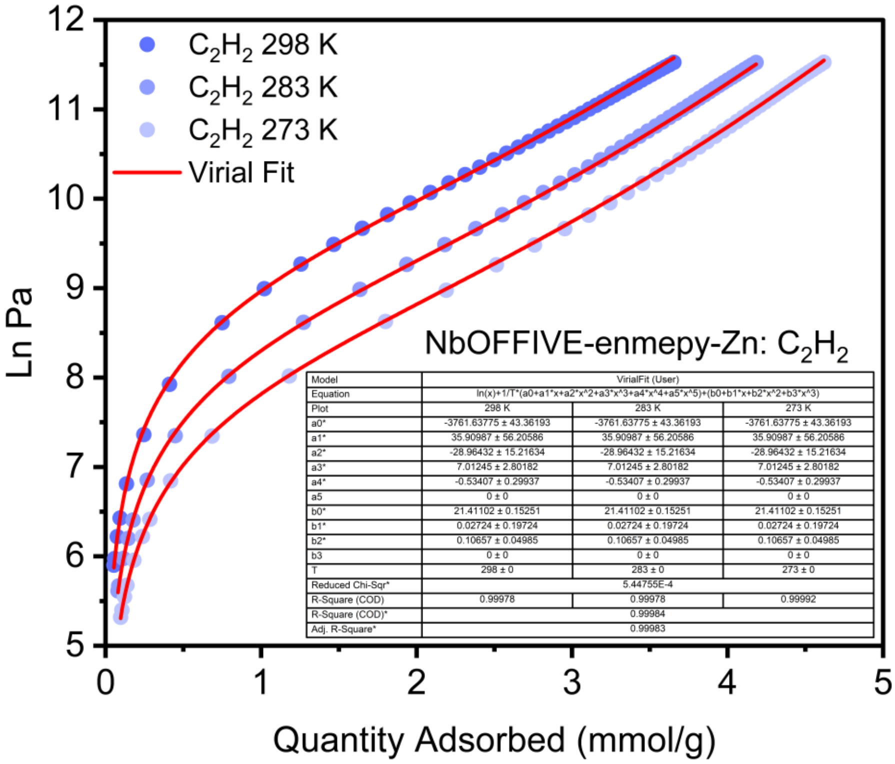

Model	VirialFit (User)		
Equation	ln(x)+1/T*(a0+a1*x+a2*x^2+a3*x^3+a4*x^4+a5*x^5)+(b0+b1*x+b2*x^2+b3*x^3)		
Plot	298 K	283 K	273 K
a0*	-3761.63775 ± 43.36193	-3761.63775 ± 43.36193	-3761.63775 ± 43.36193
a1*	35.90987 ± 56.20586	35.90987 ± 56.20586	35.90987 ± 56.20586
a2*	-28.96432 ± 15.21634	-28.96432 ± 15.21634	-28.96432 ± 15.21634
a3*	7.01245 ± 2.80182	7.01245 ± 2.80182	7.01245 ± 2.80182
a4*	-0.53407 ± 0.29937	-0.53407 ± 0.29937	-0.53407 ± 0.29937
a5	0 ± 0	0 ± 0	0 ± 0
b0*	21.41102 ± 0.15251	21.41102 ± 0.15251	21.41102 ± 0.15251
b1*	0.02724 ± 0.19724	0.02724 ± 0.19724	0.02724 ± 0.19724
b2*	0.10657 ± 0.04985	0.10657 ± 0.04985	0.10657 ± 0.04985
b3	0 ± 0	0 ± 0	0 ± 0
T	298 ± 0	283 ± 0	273 ± 0
Reduced Chi-Sqr*	5.44755E-4		
R-Square (COD)	0.99978	0.99978	0.99992
R-Square (COD)*	0.99984		
Adj. R-Square*	0.99983		

Figure S35: Virial fits and parameters used for Q_{st} calculations for; **NbOFFIVE-enmepy-Zn** for C_2H_2.

Gravimetric kinetic adsorption of gases

Gas adsorption kinetics were monitored under pure 9 cm^3 min^{-1} CO_2 and C_2H_2 flows using a TA Instruments Q50 system. Before the gravimetric kinetic adsorption cycles, the microcrystalline powders of **TIFSIX-enmepy-Zn** and **NbOFFIVE-enmepy-Zn** were activated under N_2 flow. For each cycle, the temperature was ramped up at 20 °C min^{-1} and the crystals were activated under 60 mL min^{-1} N_2 flow, then the temperature was ramped down to 30 °C (20 °C min^{-1}) and the samples were balanced under 60 mL min^{-1} N_2 flow for 30 minutes at 30 °C. After the activation and equilibrium, the sample was exposed to the 10 cm^3 min^{-1} flows of CO_2 and C_2H_2, each for **TIFSIX-enmepy-Zn** and for **NbOFFIVE-enmepy-Zn.** The kinetic adsorption profiles were evaluated using the T.A. Universal Analysis software. To convert the weight (%) values to $mmolg^{-1}$; following conversion was used:

$$n = \frac{10 \times (\Delta w)}{M}$$

Where

- n is the uptake in $mmolg^{-1}$.
- Δw is the mass gain in weight %, and
- M is the molar mass of the adsorbate in $gmol^{-1}$.

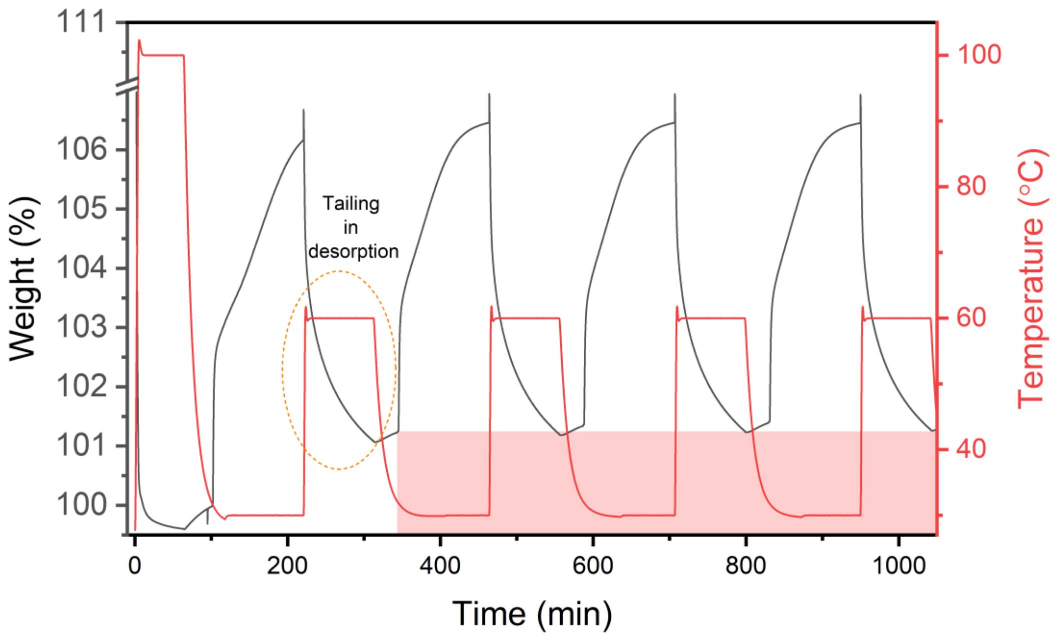

Figure S36: C_2H_2 sorption recycling test at 30 °C for **TIFSIX-enmepy-Zn**. Sorbent regeneration occurs at 60 °C under N_2 flow of 60 cm^3 min^{-1}. The rate of desorption is slow (orange circle) and does not reach full regeneration (red region).

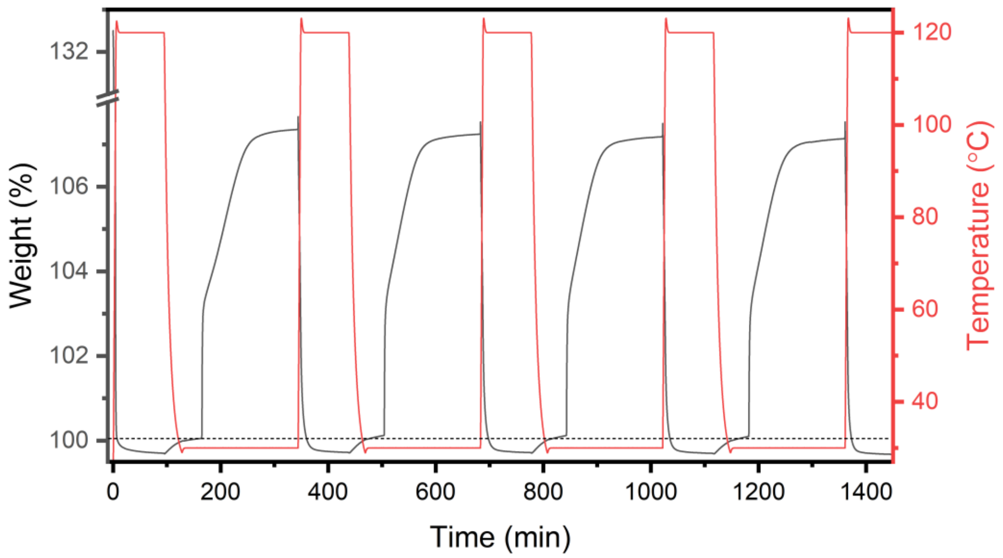

Figure S37: C_2H_2 sorption recycling test at 30 °C for **TIFSIX-enmepy-Zn**. Sorbent regeneration occurs at 120 °C under N_2 flow of 60 cm^3 min^{-1}. At higher temperature the tailing disappears, and the material gets fully regenerated (shown by dotted baseline).

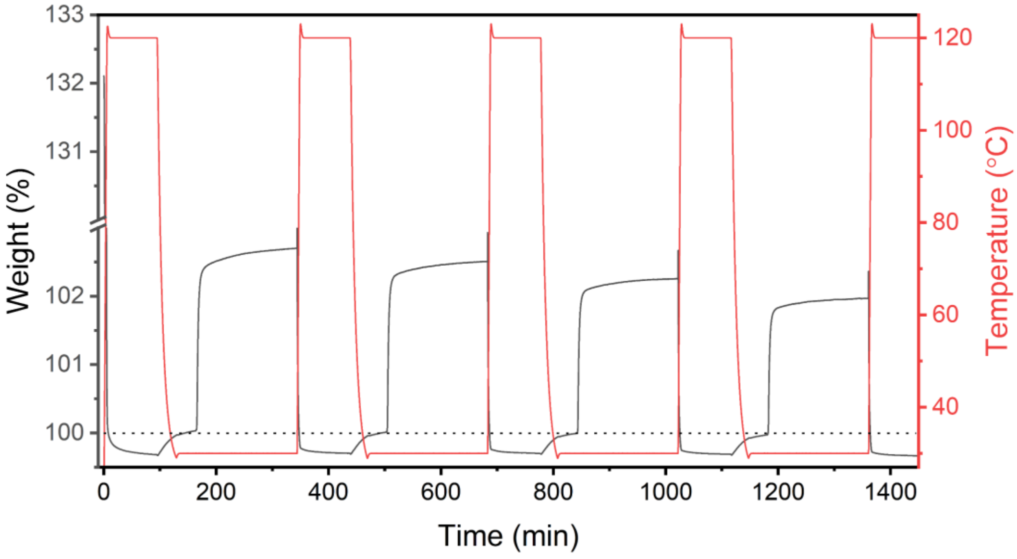

Figure S38: CO_2 sorption recycling test at 30 °C for **TIFSIX-enmepy-Zn**. Sorbent regeneration occurs at 120 °C under N_2 flow of 60 cm^3 min^{-1}. The uptake capacity is reduced with each cycle, meanwhile the baseline (dotted-line) remains intact suggesting full-desorption.

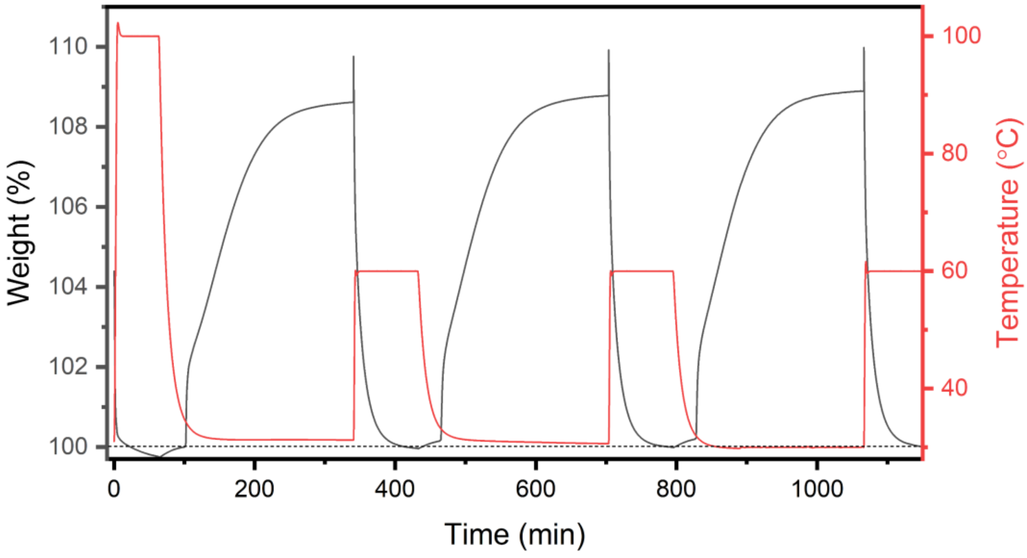

Figure S39: C_2H_2 sorption recycling test at 30 °C for **NbOFFIVE-enmepy-Zn**. Sorbent regeneration occurs at 60 °C under N_2 flow of 60 cm^3 min^{-1}.

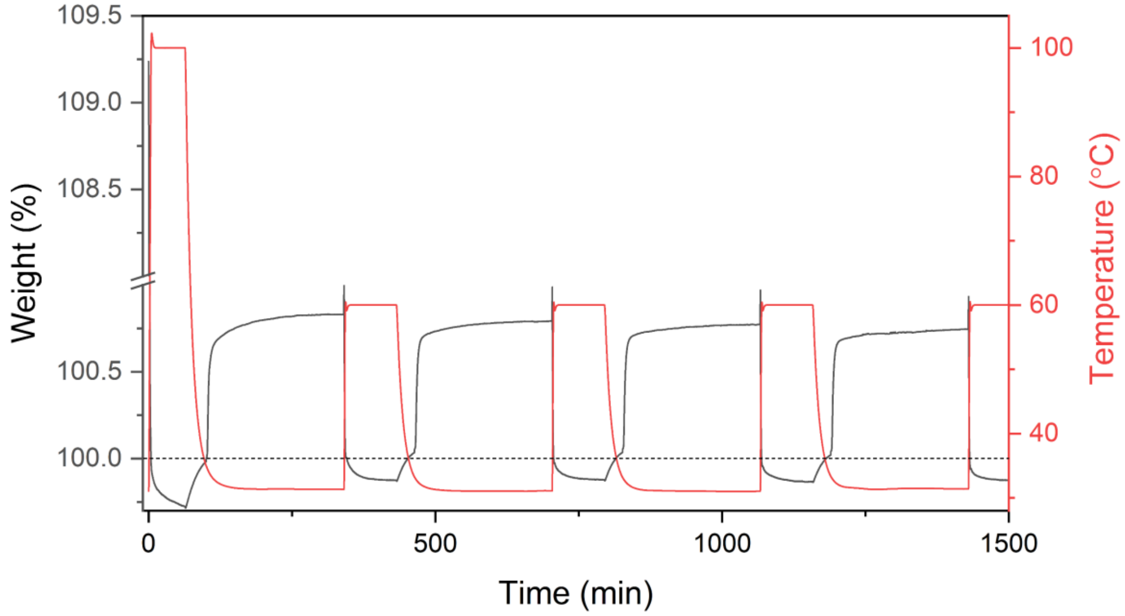

Figure S40: CO_2 sorption recycling test at 30 °C for **NbOFFIVE-enmepy-Zn**. Sorbent regeneration occurs at 60 °C under N_2 flow of 60 cm^3 min^{-1}.

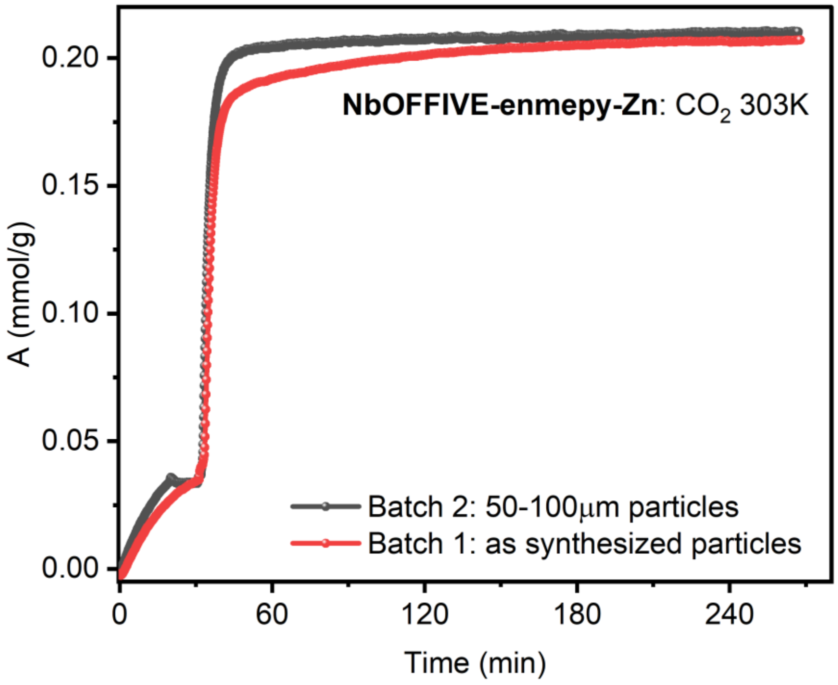

Figure S41: CO_2 adsorption profiles of **NbOFFIVE-enmepy–Zn** at **303 K** obtained from TGA for two batches of material: Batch 1 (as-synthesized particles) and Batch 2 (50–100 µm fraction). Both samples reach similar equilibrium uptakes (≈0.2 mmol g^{-1}). The region between first 0-30 minutes highlights the initial buoyancy artefact arising from the temperature change.

Water vapour sorption

Dynamic water vapour sorption isotherms were recorded using ca. ≈10 mg each of **TIFSIX-enmepy-Zn** and **NbOFFIVE-enmepy-Zn** using a DVS Adventure system from Surface Measurement Systems. Temperature was maintained at 300 K by enclosing the system in a temperature-controlled incubator. The mass of the sample was determined in parallel by comparison to an empty reference chamber and recorded by a high-resolution microbalance Ultrabalance Low Mass with a precision of 0.01 µg. Isotherms were measured from 0 to 90% R.H. with an equilibrium criterion dm/dt = 0.05 %/min. The minimum and maximum equilibration times for each step were 10 and 360 min. Before isotherms were recorded, samples were activated *in situ* by heating at 333 K for 90 minutes using the built-in sample pre-heater. Dry air was used as a carrier gas with a total flow rate of 400 mL min^{-1} (200 mL min^{-1} for each sample). Water vapour sorption kinetics was performed at 300 K using air as a carrier gas to gravimetrically measure the uptake (in weight %) and loss of vapour (desorption). Kinetics was measured using Humidity swing experiment performed between two points for 0-30 and 0-60 % RH on a ≈10 mg as synthesized sample (unless mentioned otherwise). For **TIFSIX-enmepy-Zn**; temperature swing experiments were also performed with desorption temperature 60 °C because of incomplete desorption at 27 °C

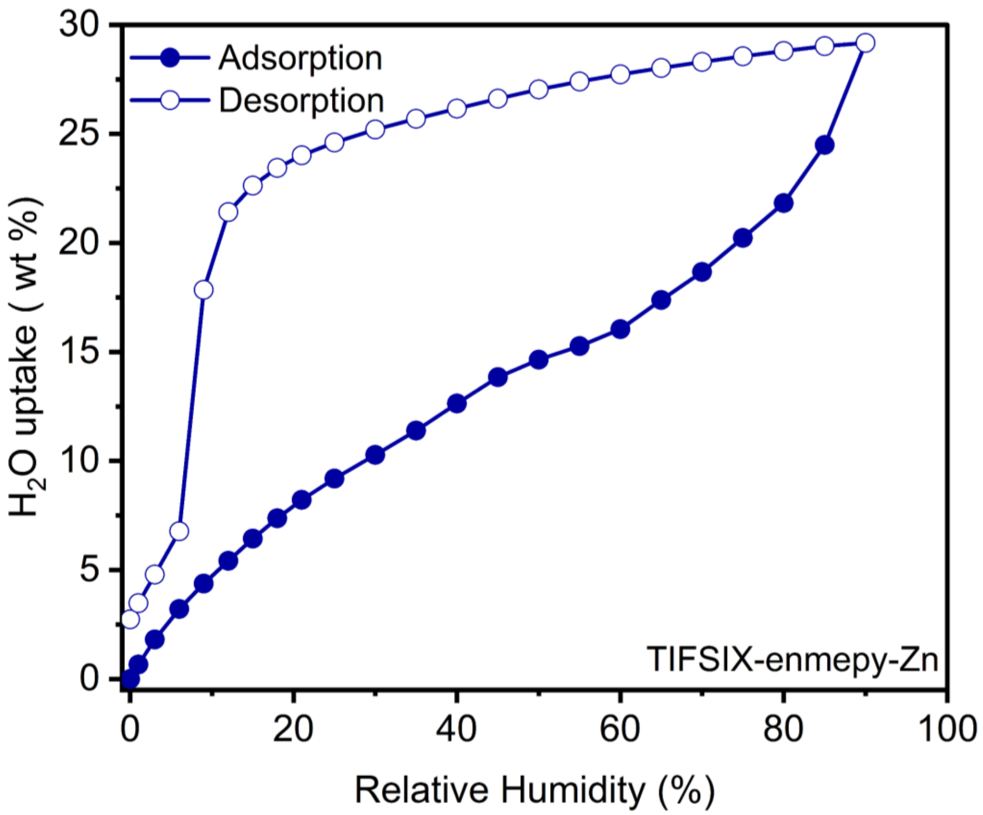

Figure S42: Water vapour sorption isotherms for **TIFSIX-enmepy-Zn** at 27 °C measured using a DVS Adventure with dry air as carrier gas (closed symbols: adsorption; open symbols: desorption; uptakes are in weight %).

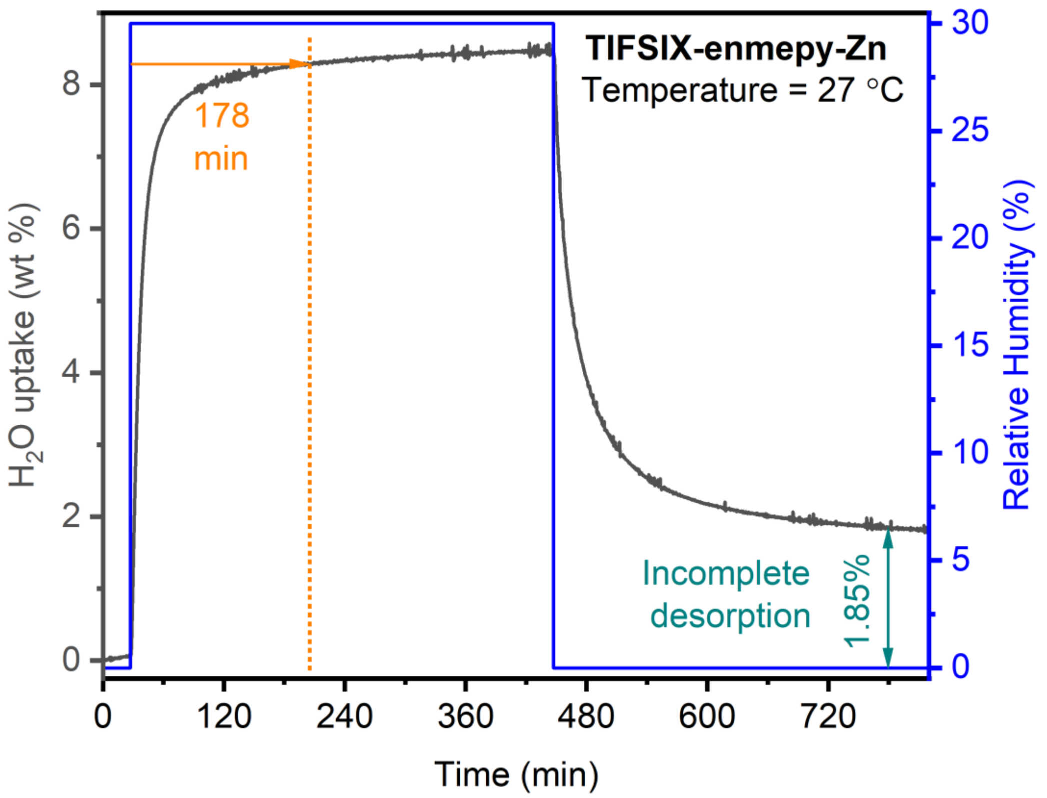

Figure S43: Adsorption and desorption kinetics for **TIFSIX-enmepy-Zn** when exposed to 30% RH, data recorded at 300 K.

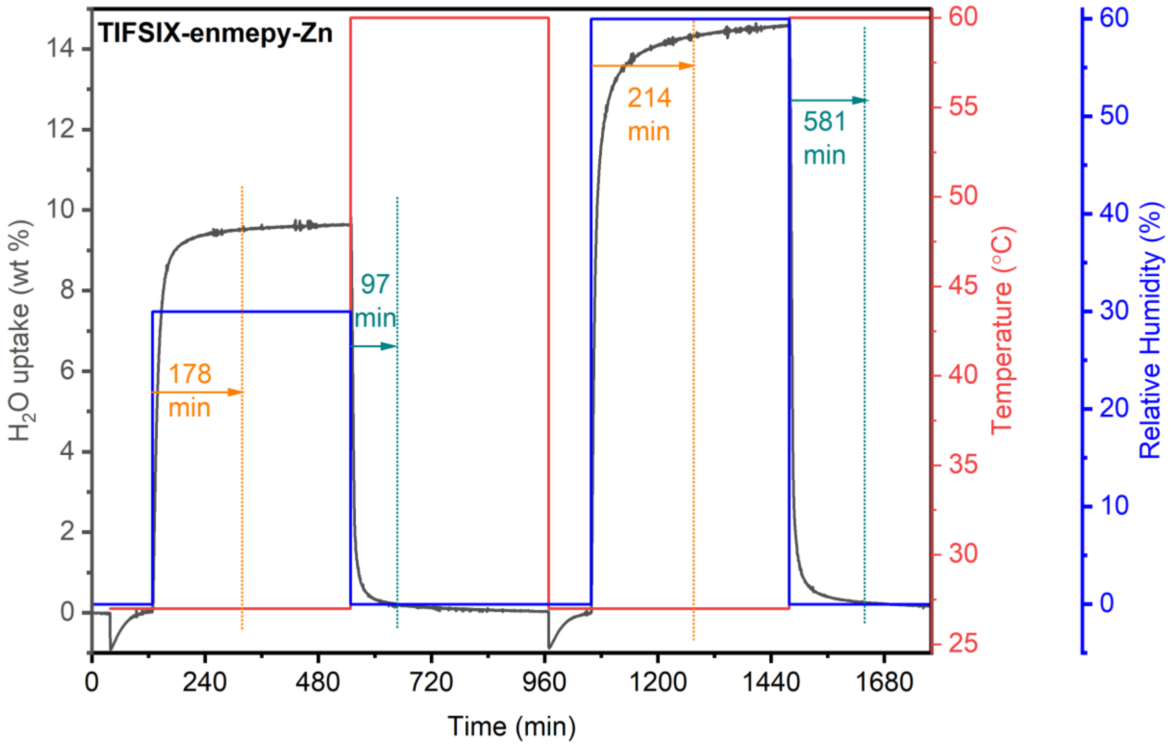

Figure S44: Adsorption and desorption kinetics for **TIFSIX-enmepy-Zn** when exposed to 30 % RH and 60%, recorded at 300 K, desorption at 333K.

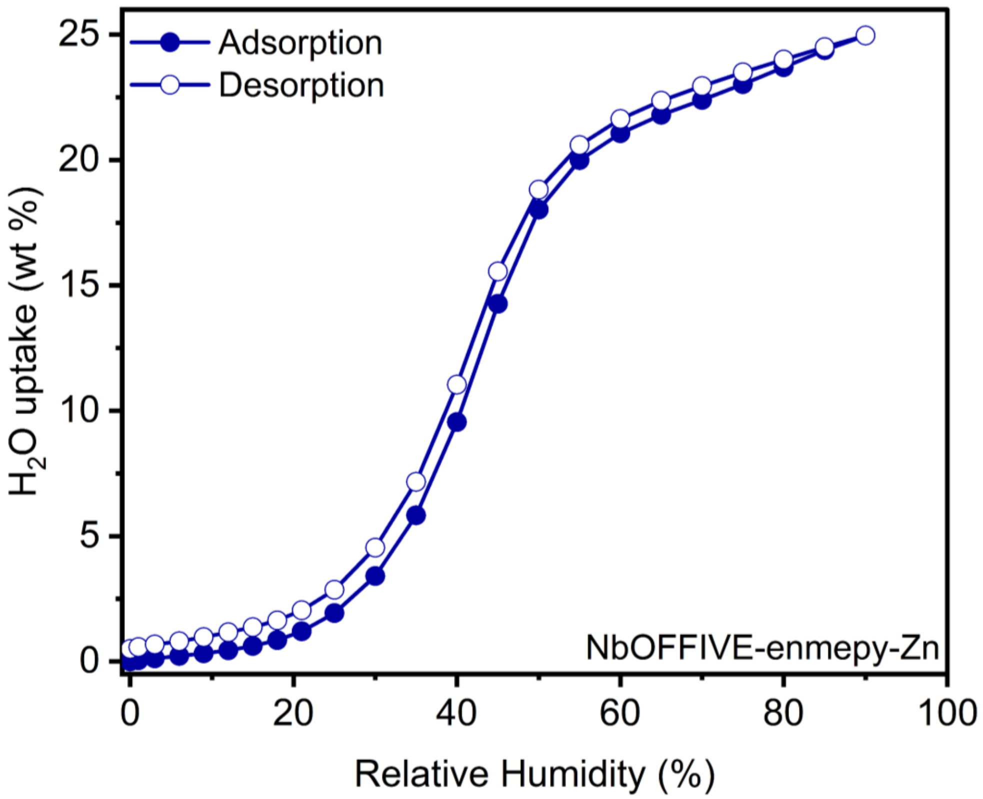

Figure S45: Water vapour sorption isotherms for **NbOFFIVE-enmepy-Zn** at 27 °C measured using the DVS Adventure with air as carrier gas (closed symbols: adsorption; open symbols: desorption; uptakes are in weight %).

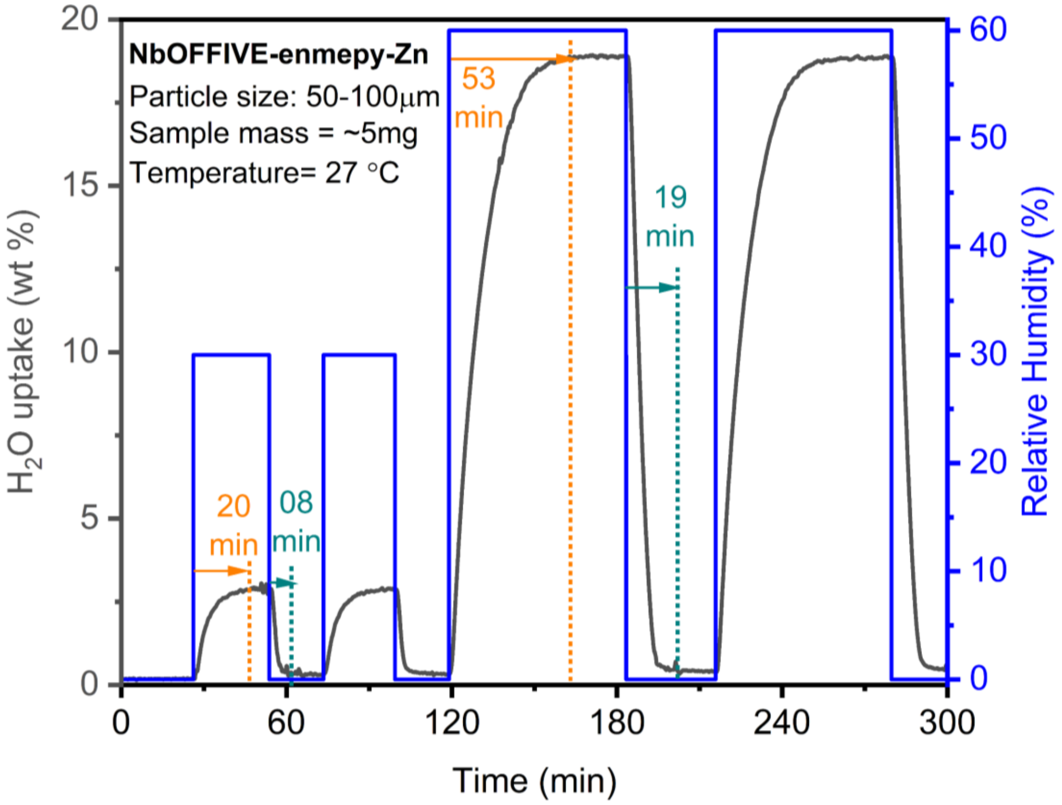

Figure S46: Adsorption and desorption kinetics for **NbOFFIVE-enmepy-Zn** when exposed to 30% RH and 60% RH with two cycles each, data recorded at 300 K.

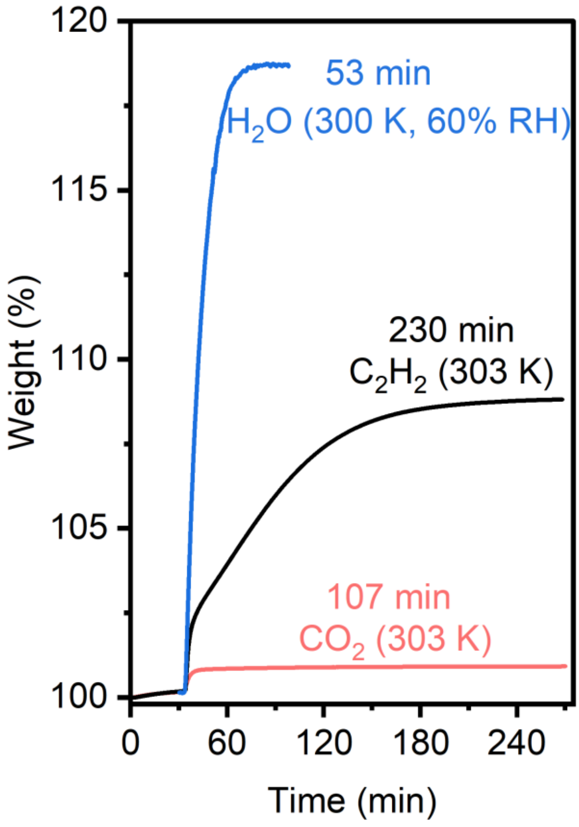

Figure S47: Adsorption kinetics of C_2H_2, CO_2 (measured by TGA), and H_2O (measured by DVS) on **NbOFFIVE-enmepy-Zn**, illustrating the relative rates of uptake. C_2H_2 displays sharp uptake at beginning and trails till saturation, CO_2 displays limited uptake, while H_2O shows fast and high adsorption. Note: Because different instruments and conditions were used, this data should be interpreted qualitatively rather than quantitatively.

Stability tests

Hydrolytic stability tests: The activated samples (≈50 mg) were kept in a vial and was kept (uncapped) in a humidity chamber 75% relative humidity (RH) at 35 °C. No activation or heat treatment was followed before PXRD.

Chemical stability tests: Approximately 10 mg of the activated sample was immersed in the respective solvent (from polar to non-polar) and stirred for 24 h at room temperature. The suspension was then allowed to settle, and the solid was recovered directly from the solvent without further activation or drying treatment prior to PXRD analysis.

Long-term capacity retention test: The sample was left in ambient for one year and then gas sorption was done in the same conditions as done earlier for the fresh sample.

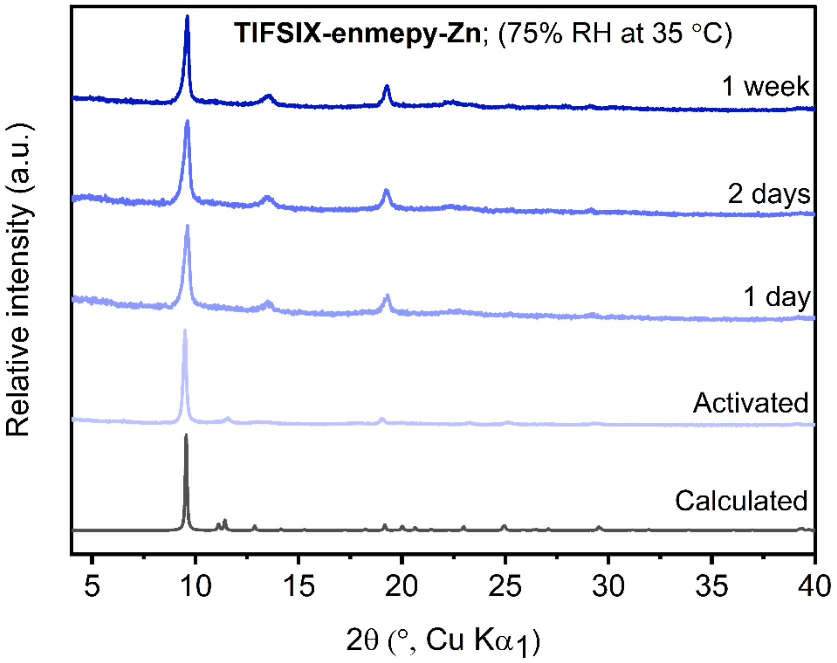

Figure S48: PXRD patterns of **TIFSIX-enmepy-Zn** upon exposing the samples at controlled humidity chamber under different conditions

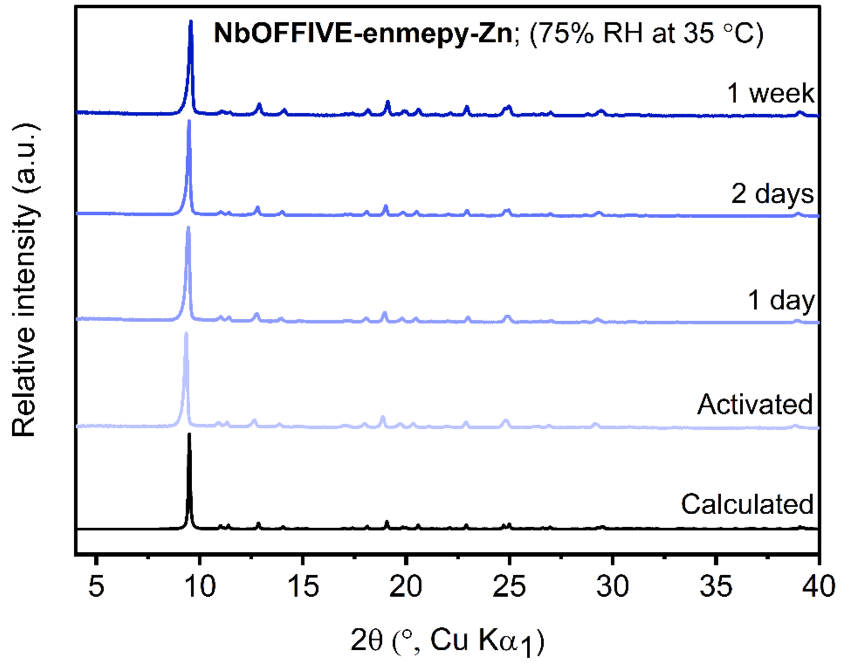

Figure S49: PXRD patterns of **NbOFFIVE-enmepy-Zn** upon exposing the samples at controlled humidity chamber under different conditions.

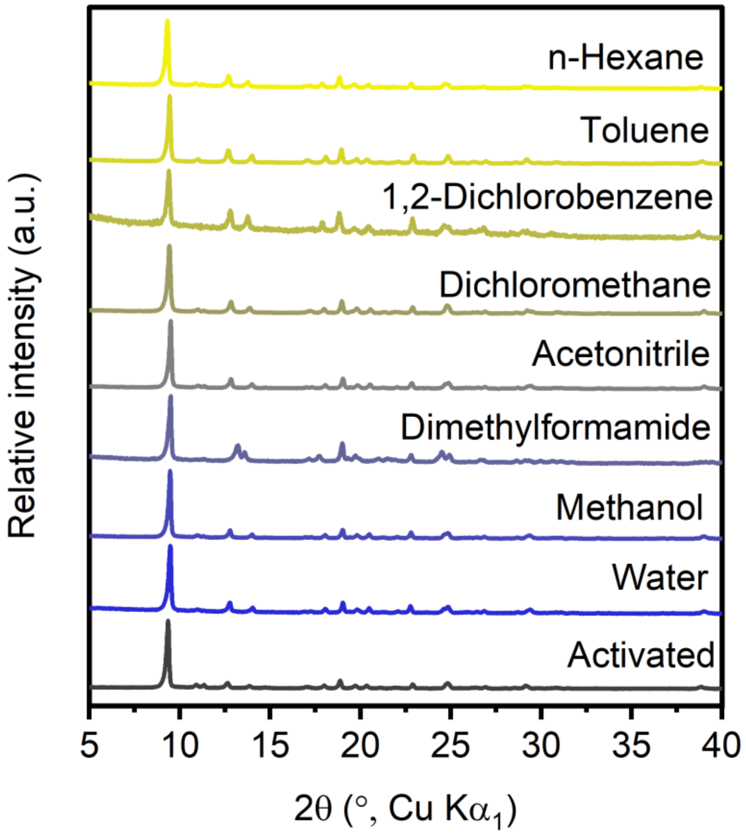

Figure S50: PXRD patterns of **NbOFFIVE-enmepy-Zn** after soaking for 24 h to different solvents, including water, methanol, dimethylformamide (DMF), acetonitrile, dichloromethane (DCM), 1,2-dichlorobenzene, toluene, and n-hexane, compared with the activated sample.

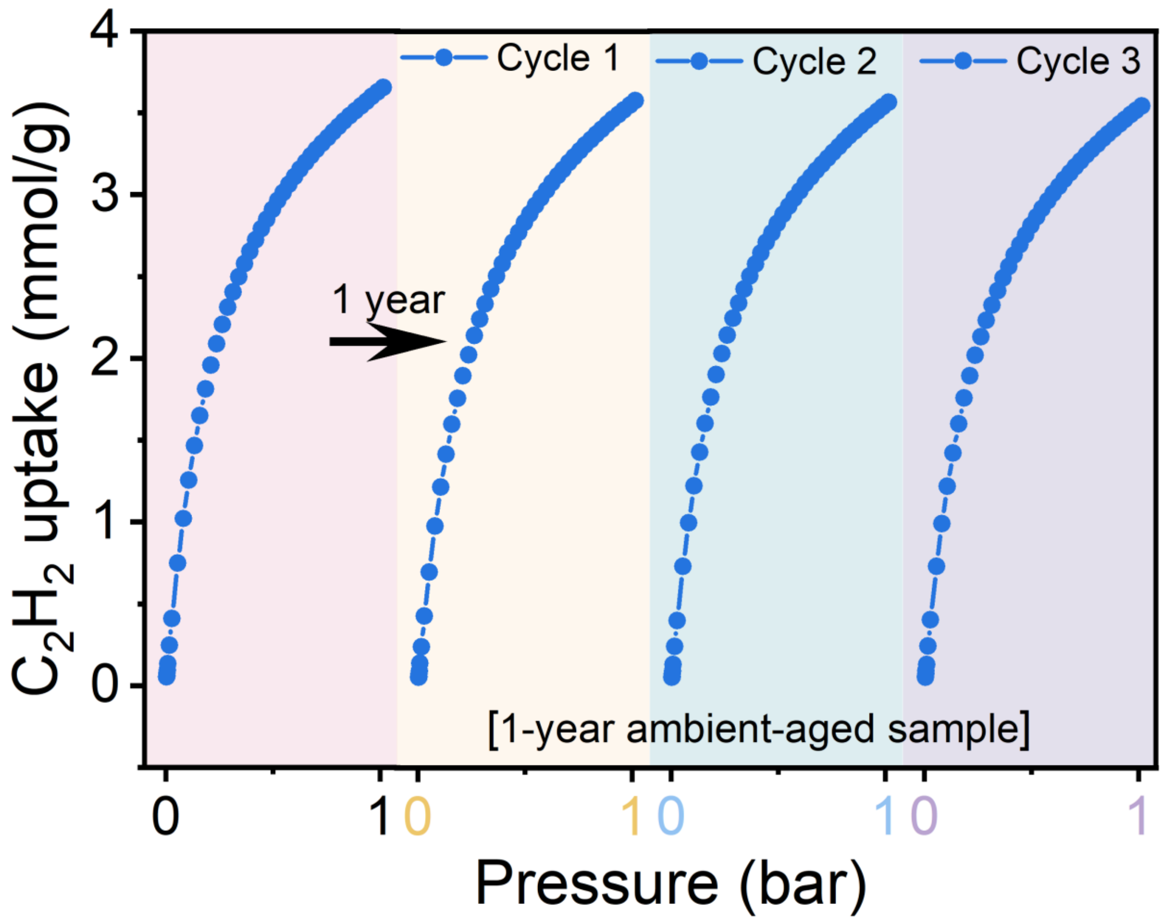

Figure S51: Multi-cycle C_2H_2 adsorption isotherms collected at 298 K for **NbOFFIVE-enmepy-Zn**, sample was aged under ambient laboratory conditions for one year. The overlapping adsorption profiles over three consecutive cycles indicate that the material retains its C_2H_2 uptake capacity and regenerability after long-term ambient storage, demonstrating excellent long-term stability.

Dynamic column breakthrough experiments

Based on single-component gas sorption data, we conducted dynamic column breakthrough (DCB) experiments on two samples of **NbOFFIVE-enmepy-Zn** and **TIFSIX-enmepy-Zn** packed in quartz tubing (0.8 mm diameter). A 1:1 C_2H_2/CO_2 mixture was passed through a column packed with the activated samples at a total flow rate of 1.28 cm^3/min, with the effluent composition monitored via a Hiden HPR-20 R&D specialist gas analysis system (mass spectrometry). Temperature-programmed desorption (TPD) experiments were performed following the dry and wet adsorption branches of the DCB experiments for **NbOFFIVE-enmepy-Zn**. For the TPD experiments, the inlet binary gas mixture was replaced with 20 cm^3/min of He, and a temperature ramp of 5 K min^{-1} was applied from 298 K to 333 K followed by holding the temperature constant at 333 K. Desorption was continued until no further adsorbate was detected.

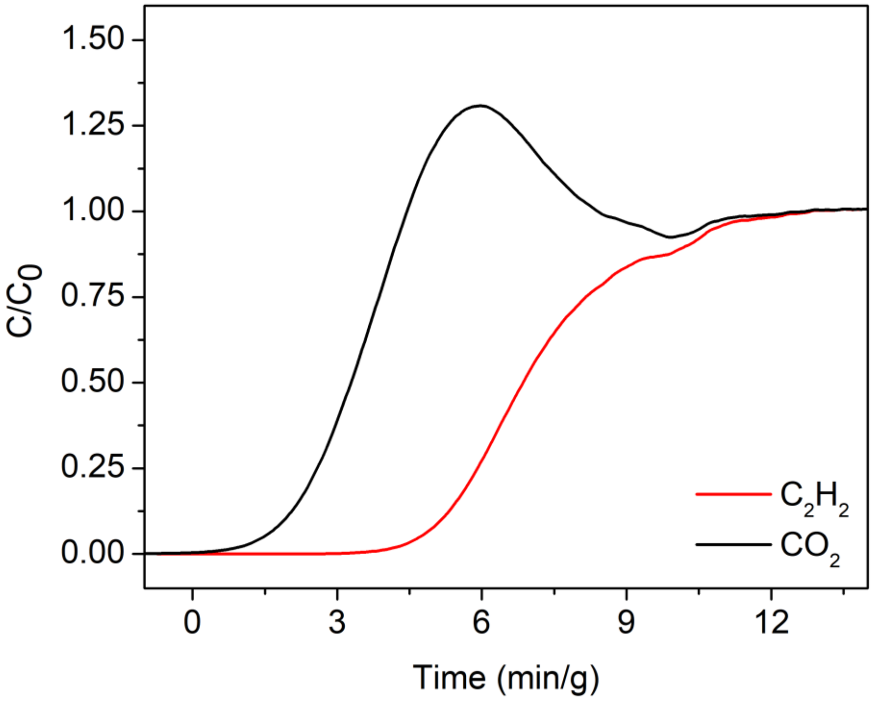

Figure S52: Dynamic column breakthrough traces showing the separation of C_2H_2 and CO_2 from an equimolar dry mixture by **TIFSIX-enmepy-Zn**.

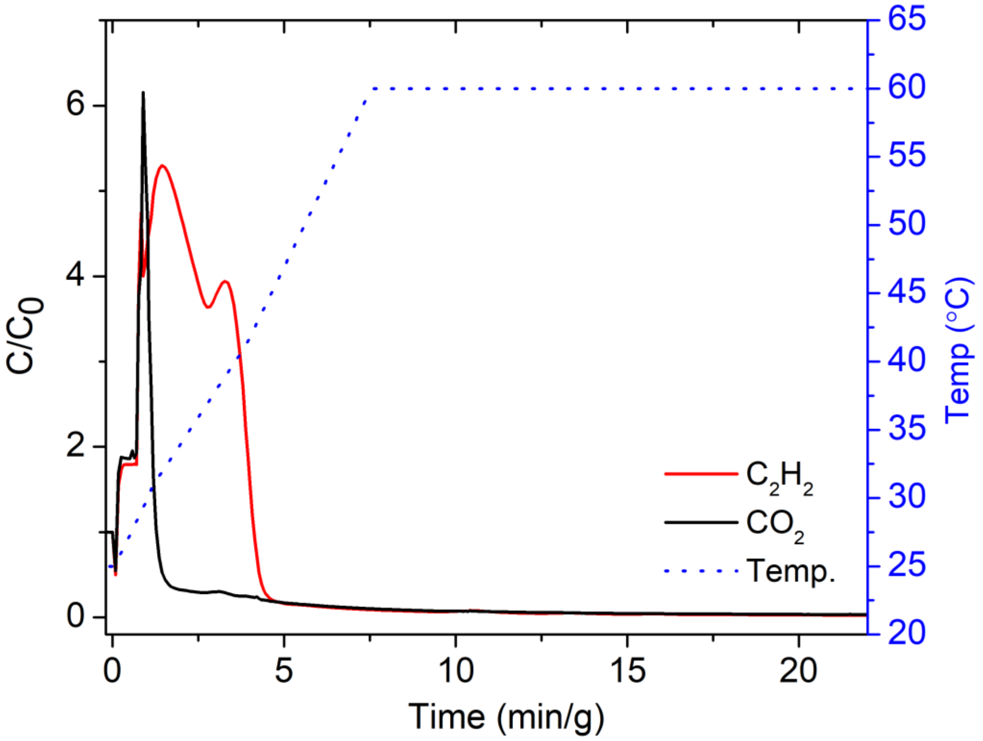

Figure S53: Temperature programmed desorption traces for C_2H_2 and CO_2 from **NbOFFIVE-enmepy-Zn** after equilibration in a wet equimolar mixture of C_2H_2 and CO_2.

Comparison of gas sorption and separation performance

Table S7: Comparison of sorption parameters for the leading C_2H_2 selective adsorbents that exhibit C_2H_2/CO_2 separation.

Serial Number	Adsorbent	Factor(s) driving C_2H_2/CO_2 selectivity	C_2H_2 uptake (mmol/g)	CO_2 uptake (mmol/g)	$Q_{st}(C_2H_2)$ (kJ/mol)	$Q_{st}(CO_2)$ (kJ/mol)	S_{AC} (1:1)	Regeneration temperature (°C)	Reference
**1**	**NbOFFIVE-enmepy-Zn**	Electrostatics	3.65	1.74	31.26	21.25	5.82	60	This work
**2**	**TIFSIX-enmepy-Zn**	Electrostatics	3.2	2.55	38.23	34.25	3.77	60	This work
**3**	**SOFOUR-TEPE-Zn**	Electrostatics	3.98	0.63	45.5	26.3	16833	70	[215]
**4**	**SIFSIX-dps-Cu**	Sieving	4.57	0.6	60.5	-	1787	25^{V}	[216]
**5**	**UTSA-300a**	Sieving	3.08	0.15	57.6	-	743	100	[217]
**6**	**Zn-SDBA-bpy**	Sieving	≈1.05	0.15	50.8	-	490.2	25	[218]
**7**	**GeFSIX-dps-Cu**	Sieving	4.04	≈0.49	56.3	-	172	70	[215]
**8**	**CPL-1-NH_2**	Sieving	1.84	0.21	50	32.4	119	100	[217]
**9**	**$CuClO_4$bipy**	Sieving	3.33	0.05	39.4	-	76	25	[219]
**10**	**Cu@UiO-66-$(COOH)_2$**	UMC	2.31	0.89	74.5	28.9	73	25^{V}	[220]
**11**	**ZNU-1**	Electrostatics	3.4	1.7	54	44	56.6	25^{V}	[221]
**12**	**ATC-Cu**	UMC	5.01	-	79.1	-	53.6	190	[222]
**13**	**Zn-bpy-DLmal**	Electrostatics	3.1	≈2.06	38.4	23.4	49	80	[223]
**14**	**ZJU-74a**	UMC	3.83	3.08	44.5	30	36.5	100	[224]
**15**	**Zn_2(bpy)(btec)**	Electrostatics	4.17	1.29	28.7	24.8	33.3	150	[225]
**16**	**sql-16-Cu-NO3-α**	Electrostatic	1.55	0.74	25.6	38.6	27.8	100	[226]
**17**	**NKMOF-1-Ni**	UMC	2.72	2.28	60.3	40.9	26	90	[227]
**18**	**FeNi-M'MOF**	UMC	4.29	2.72	27	24.5	24	25^{v}	[228]
**19**	**$Ni_3(HCOO)_6$**	Electrostatics	4.2	3.04	40.9	24.5	22	130	[229]
**20**	**ZJU-280**	Electrostatics	4.7	3.1	51	39	18.1	100	[230]

**21**	**TCuCl**	Electrostatics	3	2	41	30.1	16.9	60	[231]
**22**	**BSF-3**	Electrostatics	3.56	2.11	42.7	25.5	16.3	25	[232]
**23**	**DICRO-4-Ni-i**	Electrostatics	1.92	1.03	37.7	33.9	13.9	80	[233]
**24**	**JCM-1**	Electrostatics	3.35	1.7	36.9	33.4	13.7	100	[234]
**25**	**SNNU-65-Cu-Sc**	UMC	7.99	3.14	44.9	22.2	13.5	-	[235]
**26**	**pacs-CoMOF-2a**	UMC	5.4	2.81	34.2	24	13	-	[236]
**27**	**Li^+@NOTT-101-$(COOH)_2$**	UMC	9.14[a]	3.7	43.9[a]	29.2	13	80	[237]
**28**	**Ni_2(L-asp)$_2$(bpy)**	Electrostatics	2.68	1.4	16.6	24.2	12.6	-	[238]
**29**	**MIL-100(Fe)**	UMC	5.31[b]	2.5[b]	65	-	12.5[b]	150	[239]
**30**	**ZJU-40a**	UMC	9.64	3.34	34.5	-	11.5	-	[240]
**31**	**[$Co_2(HCOO)_2(CPT)_2$]**	Electrostatics	6.47	2.68	33	27	11		[241]
**32**	**V-bcp-tppy**	-	5.37	3.25	44.1	29.7	11		[242]
**33**	**CAU-10-NH_2**	Amine sites	4.3[d]	2.5[d]	31.3	24.5	10.8		[243]
**34**	**ZNU-9**	Electrostatics	7.94	4.32	33.1	26.6	10.3	50	[244]
**35**	**SIFSIX-21-Cu**	Electrostatics	3.9	1.5	36.3	24	10	60	[245]
**36**	**TIFSIX-2-Cu-i**	Electrostatics	4.1	4.3	46.3	34.2	10	70	[246]
**37**	**ZJUT-2a**	Electrostatics	3.39[a]	2.19[a]	41.5	31.5	10[a]	100	[247]
**38**	**MIL-160**	Electrostatics	9.5	4	31.8	26.9	10	80	[248]
**39**	**Zn-bpy-fum**	Electrostatics	1.94	≈0.62	24.8	23.8	10		[223]
**40**	**SNNU-37(Fe)**	UMC	4.9	2.1	34.4	33.4	9.9		[249]
**41**	**BSF-4**	Electrostatics	2.38	1.6	35	24.5	9.8		[250]
**42**	**NbOFFIVE-3-Cu**	Electrostatics	4	1.6	41.9	24.8	9.5	60	[245]
**43**	**TCuBr**	Electrostatics	2.8	2	36.8	26.8	9.5		[231]
**44**	**NbOFFIVE-dps-Cu**	Electrostatics	1.65	1.1	53.6	-	9	25	[216]
**45**	**UTSA-74**	UMC	4.78	3.17	31.7	25	9		[251]
**46**	**CPL-1**	Electrostatics	2.01	1.26	45.5	36.6	9		[252]
**47**	**ZJUT-6a**	UMC	4.93[a]	3.77[a]	35	30	8.9[a]	100	[253]

**48**	**TIFSIX-4-Cu**	Electrostatics	3.5	2	40.6	24	8.3	60	[245]
**49**	**SOFOUR-2-Zn**	Electrostatics	2.6	1.4	34.3	21.9	8.1	60	[254]
**50**	**iMOF-6C**	Electrostatics	1.2	0.68	38	-	8		[255]
**51**	**SIFSIX-21-Ni**	Electrostatics	4	1.3	37.9	19.8	7.8	60	[245]
**52**	**TIFSIX-4-Ni**	Electrostatics	3.8	2	41.4	27.4	7.6	60	[245]
**53**	**SIFSIX-24-Zn**	Electrostatics	3.7	1.8	31.8	17.1	7.4	60	[254]
**54**	**$[Zn_3(HCOO)_6]_n$**	Electrostatics	3.5	3.1	41.5	25.4	7.4		[256]
**55**	**SNNU-150-Al**	UMC	4.33	1.98	29	24	7.27		[257]
**56**	**ZNU-14**	Electrostatics	1.94	1.07	34.4	25.9	7.2		[258]
**57**	**ZJU-60a**	UMC	6.7	3.3	17.6	15.2	6.7[c]		[259]
**58**	**SNNU-65-Cu-Fe**	UMC	7.25	2.9	28.2	21.8	6.7		[249]
**59**	**SOFOUR-1-Zn**	Electrostatics	3.1	3.6	57	33	6.6	60	[260]
**60**	**NTU-55**	UMC	6.05	3.13	25	22	6.6[c]		[261]
**61**	**SIFSIX-22-Zn**	Electrostatics	5.7	4.2	36.5	25	6.5	60	[260]
**62**	**UTSA-83**	UMC	0.53	0.17	24.4	16.6	6.2		[262]
**63**	**DZU-1**	Electrostatics	4	3	3.17	30.6	6.1		[263]
**64**	**TIFSIX-2-Ni-i**	Electrostatics	4.21	4.54	40	34	6.1	35	[264]
**65**	**NbOFFIVE-3-Ni**	Electrostatics	3.8	1.9	36.7	25	6	60	[245]
**66**	**PCP-33**	UMC	5.44	2.62	27.5	26.2	6		[265]
**67**	**NTU-66-Cu**	UMC	4.98	2.19	32.3	21.7	6		[266]
**68**	**MUF-17**	Electrostatics	3.01[b]	2.51[b]	49.5	33.8	6		[267]
**69**	**iMOF-5C**	Electrostatics	1.5	0.71	35.5	-	6		[255]
**70**	**FJI-H8-Me**	UMC	10.2	4.73	33.7	21.77	≈6		[268]
**71**	**CPM-107op**	Electrostatics	4.35	1.7	32	24	5.7		[269]
**72**	**ZJNU-13**	Electrostatics	5.29	3.92	33.5	22.5	5.64		[270]
**73**	**SNFSIX-2-Zn**	Electrostatics	5	3.9	41.3	43.3	5.6	-	[271]
**74**	**Cu-ABTC**	Electrostatics	8.74	4.46	31.9	22.8	5.6		[272]

**75**	**IPM-101**	Electrostatics	2.55	3.04	43.7	30.7	5.4		[273]
**76**	**TCuI**	Electrostatics	2.2	1.6	38.4	30.7	5.3		[231]
**77**	**SIFSIX-Cu-TPA**	Electrostatics	8.26	≈4.5	39.1	25.7	5.3	100	[270]
**78**	**TIFSIX-6-Zn**	Electrostatics	5.7	4.3	44.8	30.4	5.3	-	[271]
**79**	**UTSA-98**	UMC	3.7	1.8	22.8	18.3	5.2		[274]
**80**	**UPC-110**	Electrostatics	3.28	1.08	24.6	16	5.1		[275]
**81**	**BSF-2**	Electrostatics	1.85	1.33	37.3	28.7	5.1		[231]
**82**	**JXNU-5a**	UMC	2.5	1.55	32.9	25.2	5		[276]
**83**	**GEFSIX-4-Zn**	Electrostatics	5.4	4.4	44.8	34	5	-	[271]
**84**	**Cu-TPTC**	Electrostatics	7.58	3.48	29.7	22	4.5		[272]
**85**	**ZRFSIX-3-Zn**	Electrostatics	4.6	3.6	42.7	42.6	4.4	-	[271]
**86**	**Co_4-1,4-ndc**	Electrostatics	5.63	3.39	29.9	≈24.6	4.3		[277]
**87**	**FJUT-1**	Electrostatics	5.94	2.65	43.75	37.39	4.06		[278]
**88**	**MFM-160a**	Electrostatics	5.71	3.06	37	30	4		[279]
**89**	**NOTT-101-$(COOH)_2$**	UMC	6.6[a]	3.43[a]	29.7	27.4	3.8		[237]
**90**	**MFOF-1**	Electrostatics	4.72	2.3	31.88	25.97	3.66		[278]
**91**	**V-bdc-tpt**	-	≈8.21	4.44	27	19.9	3.3		[242]
**92**	**Zn-MOF-74**	UMC	5.5	5.4	22.1	-	2		[251]
**93**	**JNU-1**	Induced fit	2.7	2.2	13	24	3		[280]
**94**	**UTSA-68**	UMC	3.13	1.77	25.8	26	3.4		[281]

a = data collected at 296 K; b = data collected at 293 K; ≈ = data extrapolated from reported plots.[282];

c = at 0.15 bar; d = volumetric uptake. UMC= unsaturated metal centres. V = under vacuum

In-situ Infrared (IR) spectroscopy

In situ IR measurements were performed on a Nicolet™ iS50 FTIR spectrometer using a liquid N_2-cooled mercury cadmium telluride (MCT-A) detector. The spectrometer is equipped with a vacuum cell that is placed in the main compartment with the sample at the focal point of the infrared beam. The samples (≈5 mg) were gently pressed onto KBr pellet and placed into a cell that is connected to a vacuum line for evacuation. The sample was activated by evacuation at 100 ºC and then cooled back to room temperature for gas adsorption measurement.

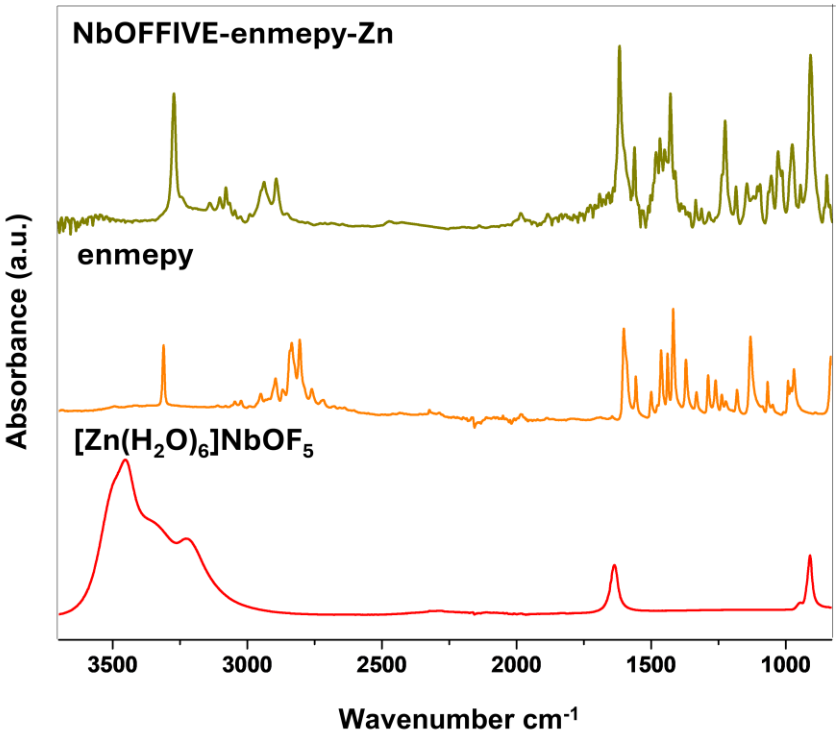

Figure S54: FTIR spectra of **NbOFFIVE-enmepy-Zn**, the enmepy ligand, and the inorganic precursor $[Zn(H_2O)_6]NbOF_5$.

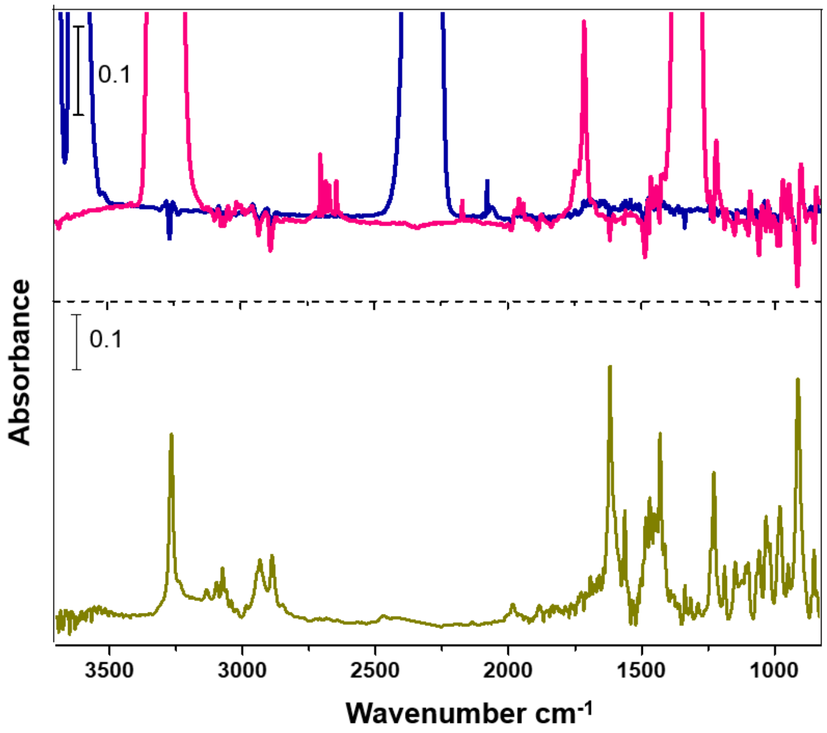

Figure S55: *In-situ* IR difference spectra by **NbOFFIVE-enmepy-Zn** referencing the spectra of C_2H_2- (pink line) and CO_2- (deep blue line) loaded **NbOFFIVE-enmepy-Zn** to that of activated (empty) structure (bottom brown line). The gas was loaded at ≈760 Torr and 24 ºC.

Molecular Modelling

Canonical Monte Carlo (CMC) simulations [283] were conducted to identify the primary binding site locations for C_2H_2, CO_2, and H_2O within the **NbOFFIVE-enmepy-Zn** and **TIFSIX-enmepy-Zn** frameworks. These simulations were performed using Materials Studio at 298 K on a 2x2x2 supercell of the framework. Atomic point charges for both the frameworks and the adsorbates were calculated using the charge equilibration (Q_{eq}) method[284] to ensure an accurate representation of electrostatic interactions. The specific point charges applied for C_2H_2, CO_2, and H_2O are provided in **Figure S56**.

In the CMC simulations, the framework structures were kept rigid. Each simulation maintained a constant loading of one sorbate molecule per supercell. Simulations were carried out in the canonical ensemble using the Metropolis sampling method to explore different molecular configurations. Four types of moves were employed to sample the configurational space: translation (shifting the centre of mass of a selected adsorbate), rotation (rotating the molecule), regrowth (removing and randomly reinserting the molecule with a new orientation), and conformer changes (sampling different internal conformations of the adsorbate). These moves were attempted with relative probabilities of 1 for translation, 1 for rotation, 0.1 for regrowth, and 1 for conformer changes.

A cutoff distance of 12 Å was used for non-bonded interactions, with potentials smoothly truncated using a cubic spline with a width of 1 Å. All CMC simulations were performed using the Universal Force Field (UFF)[284] from the Materials Studio forcefield library. Each simulation consisted of 4×10^6 loading steps, followed by 4×10^6 equilibration steps and 4×10^6 production steps to ensure robust ensemble averaging. Simulation results were visualised as adsorbate density fields, based on the mass-centre positions of all accepted Monte Carlo moves (see **Tables S9** and **S10**). Additionally, isosurfaces at a

constant density (isovalue = 0.0003) were generated and colour-coded by the corresponding potential energy values (see **Figures 6d-f** and **S57**). These colour-mapped isosurfaces clearly show that C_2H_2 in both **NbOFFIVE-enmepy-Zn** and **TIFSIX-enmepy-Zn** experiences the lowest potential energies, revealing the most favourable adsorption sites, consistent with experimental findings from Q_{st} and IAST analysis (**Figures 2d, 2g**).

Grand Canonical Monte Carlo (GCMC) simulations were performed to better understand the adsorption behaviour of C_2H_2 and CO_2 in a 2×2×2 supercell of the **NbOFFIVE-enmepy-Zn** framework. Details of the Metropolis sampling technique used can be found in the CMC section above. Adsorption isotherms were computed across a pressure range from 0 to 1 bar. Each GCMC simulation included 3 × 10^6 equilibration steps followed by 3 × 10^6 production steps to ensure statistically reliable ensemble averages. The same force field parameters and cutoff distances as in the CMC simulations were applied to maintain consistency. **Figure S58** shows the simulated adsorption isotherms of C_2H_2 and CO_2 in **NbOFFIVE-enmepy-Zn**. The results clearly indicate that C_2H_2 exhibits the highest adsorption capacity within the framework, in agreement with experimental data (**Figure 2b**).

Furthermore, we performed **hybrid Grand Canonical Monte Carlo/Molecular Dynamics (GCMC/MD)** simulations to investigate the adsorption behaviour of C_2H_2, CO_2, and H_2O within the 2×2×2 supercell **NbOFFIVE-enmepy-Zn**, incorporating dynamic effects beyond static adsorption. For C_2H_2 and CO_2, hybrid GCMC/MD simulations were carried out across a range of pressures from 0 to 1 bar at 298 K, specifically at 23 pressure points: 0.001, 0.03, 0.05, 0.10, 0.15, 0.20, 0.25, 0.30, 0.35, 0.40, 0.45, 0.50, 0.55, 0.60, 0.65, 0.70, 0.75, 0.80, 0.85, 0.90, 0.95, and 1 bar. For H_2O, simulations were conducted over the relative humidity (RH) range from 0% to 100%, also at 298 K. At each pressure point, simulations began with GCMC calculations consisting of 1×10^6 Monte Carlo (MC) steps for both equilibration and production. Details of the Metropolis sampling approach used here are described in the Computational Methodology section. From the GCMC output at each pressure, five low-energy configurations were randomly selected. The configuration with the lowest total energy was then chosen as the starting structure for a subsequent MD simulation. Each selected configuration was equilibrated via a 200 ps MD run under NVT (constant number of particles, volume, and temperature) conditions, with the framework treated as fully flexible. The temperature was maintained at 298 K using a Nosé–Hoover thermostat, and atomic trajectories were integrated using the velocity-Verlet algorithm with a 1 fs time step. From the last 50 ps of each MD trajectory, ten frames were sampled at 5 ps intervals. The frame with the lowest energy was selected and used as the input for the next GCMC calculation at the subsequent pressure level. This hybrid GCMC/MD approach offers a key advantage over traditional GCMC simulations: by incorporating atomic dynamics and framework flexibility, it captures both adsorption and diffusion processes to provide a more realistic picture of molecular behaviour within porous materials.

As part of the post-simulation analysis, we calculated the mean squared displacement (MSD) of the adsorbates at every studied pressure to assess their dynamic behaviour within the framework. The MSD was calculated using the following equation:

$$MSD\ (t) =< [r(t) - r(0)]^2 > \quad (1)$$

$r(t)$ represents the position of a particle at time t. The MSD plots as a function of both time and pressure presented in **Figure S59**. The graphs reveal that H_2O exhibits significantly higher mobility compared to both C_2H_2 and CO_2 across all simulated pressures. This enhanced mobility of water can be attributed primarily to its smaller molecular size and compact geometry, which allow it to diffuse more easily through the framework's pore network with reduced steric hindrance.

Additionally, the interaction energy maps (**Figures 6 and S57**) indicate that H_2O interacts weakly with the frameworks compared to C_2H_2 and CO_2, which experience stronger binding at specific adsorption sites. As a result, H_2O is less confined and can move more freely between different regions of the pore space. Although water's polar nature may lead to transient electrostatic interactions with the framework, these are generally short-lived and do not significantly impede its motion. This computational observation aligns well with the results of kinetic uptake experiments (**Figure S47**), which show that water displays the fastest adsorption–desorption kinetics among the three sorbates.

Computational studies were undertaken to rationalise the choice of the enmepy ligand, which introduces chemical heterogeneity through the presence of both secondary amine and pyridyl nitrogen donors, unlike ligands that feature only one type of N-donor. Local charges were calculated using the Q_{eq} method and compared against two known hybrid coordination networks, 4,4'-bipyridine (bpy) based **SIFSIX-1-Zn (CSD: ZESFUY)**, and pyrazine-derived **SIFSIX-3-Zn (CSD: FUDQIF)** (**Table S8**). The Zn centre in **NbOFFIVE-enmepy-Zn** carries a higher local charge (+0.348) than in **SIFSIX-3-Zn** (+0.327) and **SIFSIX-1-Zn** (+0.307), indicating greater net electron donation to the metal. This is rationalized within ligand field theory: the aliphatic amine nitrogens of enmepy are sp^3-hybridized, with lone pairs fully available for σ-donation and not delocalized into any aromatic system, making them inherently stronger donors than the sp^2-hybridized pyridyl or pyrazine nitrogens, as directly evidenced by their markedly more negative Q_{eq} charges (−0.408, −0.403 vs −0.265, −0.270). This increased polarization of the coordination environment may also contribute to enhanced framework stability.

Periodic Density Functional Theory calculations were also performed to quantify the binding energies of C_2H_2 and CO_2 in **NbOFFIVE-enmepy-Zn**. Calculations were carried out using the BEEF-vdW exchange-correlation functional[285] as implemented in the Vienna Ab Initio Simulation Package (VASP 6.4.2).[286–287] The projected augmented wave (PAW) formalism[288] was employed with standard PAW potentials. Atomic positions were relaxed within the experimental unit cells using the conjugate gradient algorithm, with force and electronic convergence criteria of 0.02 eV $Å^{-1}$ and 10^{-6} eV, respectively. A plane-wave energy cutoff of 550 eV, and Gaussian smearing of 0.02 eV. Furthermore, the optimization was performed employing a Γ-centred 3×3×3 k-point grid. The calculated binding energies for C_2H_2 and CO_2 in **NbOFFIVE-enmepy-Zn** are ≈-39.5 and ≈-35.9 kJ mol^{-1}, respectively. These values are consistent with the Monte Carlo results, confirming stronger binding affinity for C_2H_2 over CO_2 in **NbOFFIVE-enmepy-Zn**.

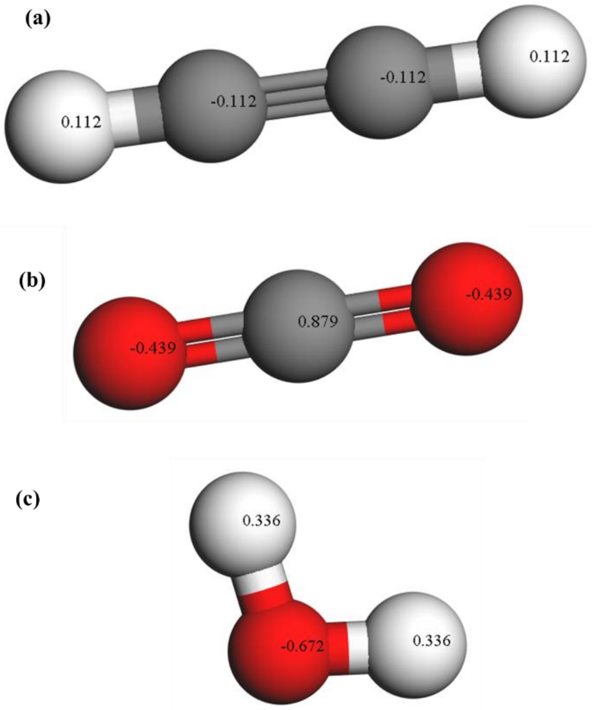

Figure S56: Atomic structures and point charges of (a) C_2H_2, (b) CO_2, and (c) H_2O.

Table S8. Local charges on Zn and coordinating N atoms in the DFT-optimized structures.

Material	Zn	N(amine)	N(pyridyl/pyrazine)
**NbOFFIVE-enmepy-Zn**	+0.348	−0.408, −0.403	−0.265, −0.265
**SIFSIX-1-Zn**	+0.307	-	−0.265, −0.265, −0.265, −0.265
**SIFSIX-3-Zn**	+0.327	-	−0.270, −0.270, −0.270, −0.270

Table S9: The adsorbate density fields obtained from CMC simulations for C_2H_2, CO_2, and H_2O at 298 K in **NbOFFIVE-enmepy-Zn**. They allow the identification of optimal binding sites. The colour map values indicate the density of the adsorbate in g/cm[3].

	**View I**	**View II**
C_2H_2	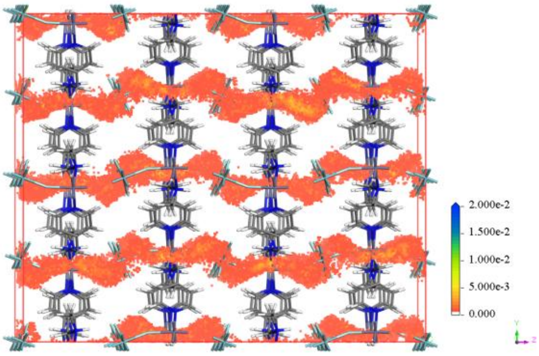	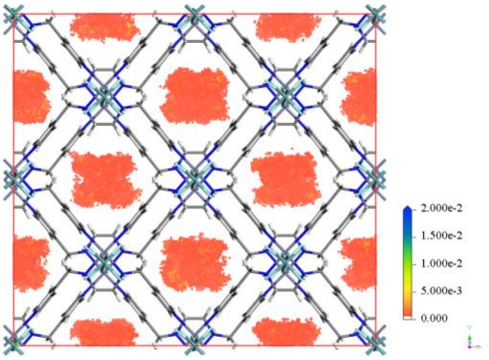
CO_2	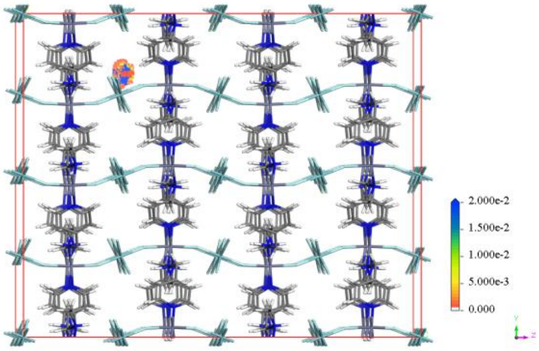	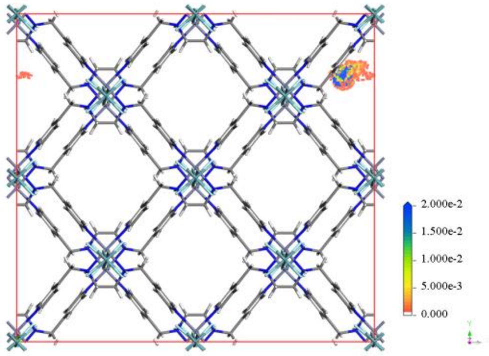
H_2O	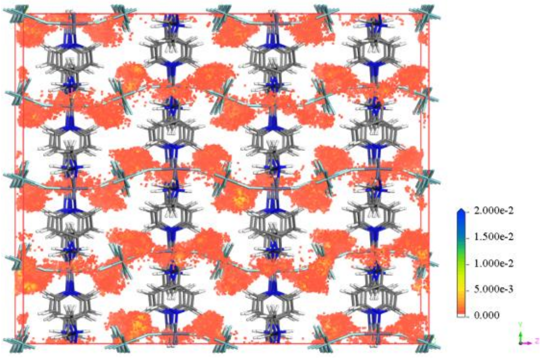	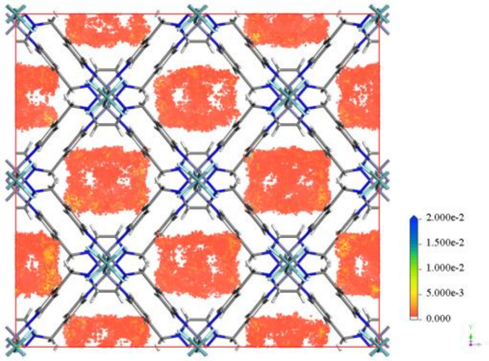

Table S10: The adsorbate density fields obtained from CMC simulations for C_2H_2, CO_2, and H_2O at 298 K in **TIFSIX-enmepy-Zn**. They allow the identification of optimal binding sites. The colour map values indicate the density of the adsorbate in g/cm^3.

	View I	View II
C_2H_2	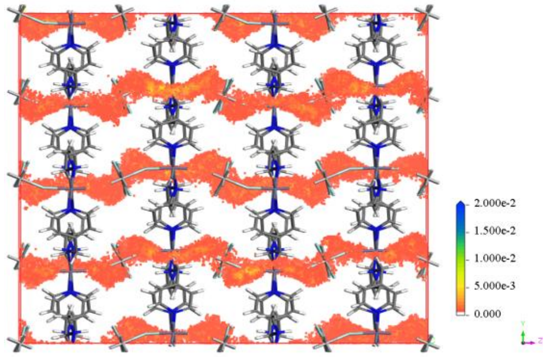	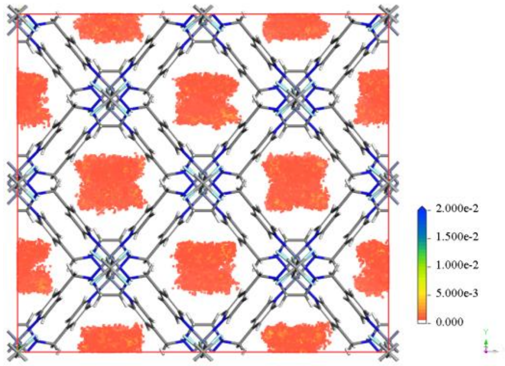
CO_2	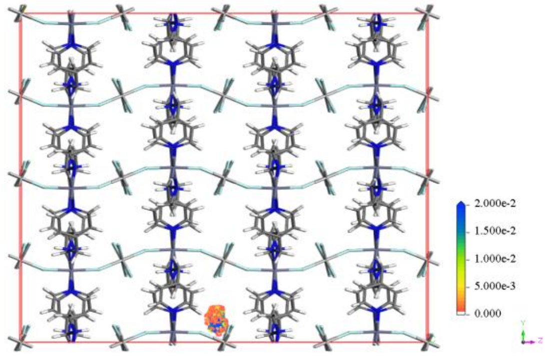	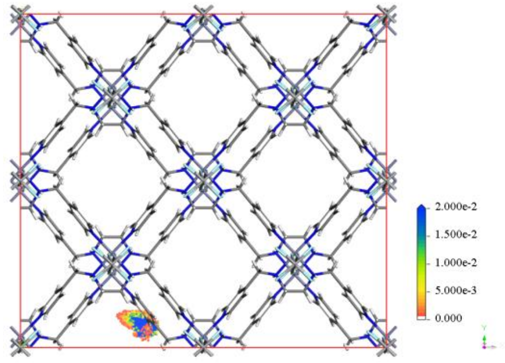
H_2O	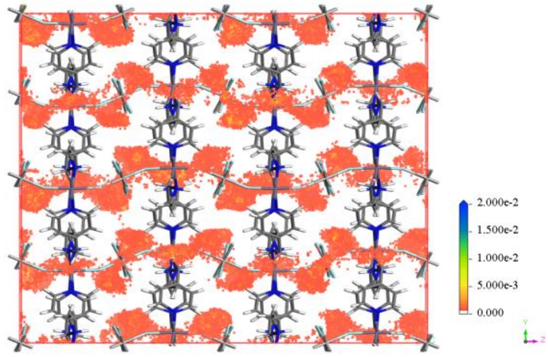	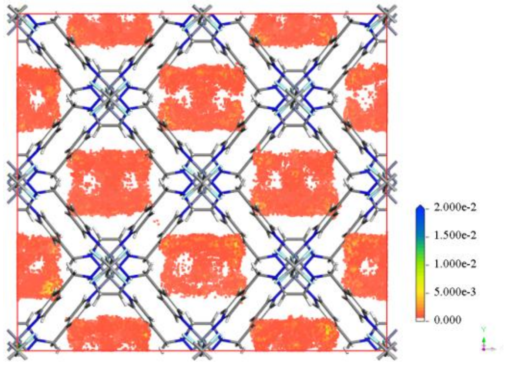

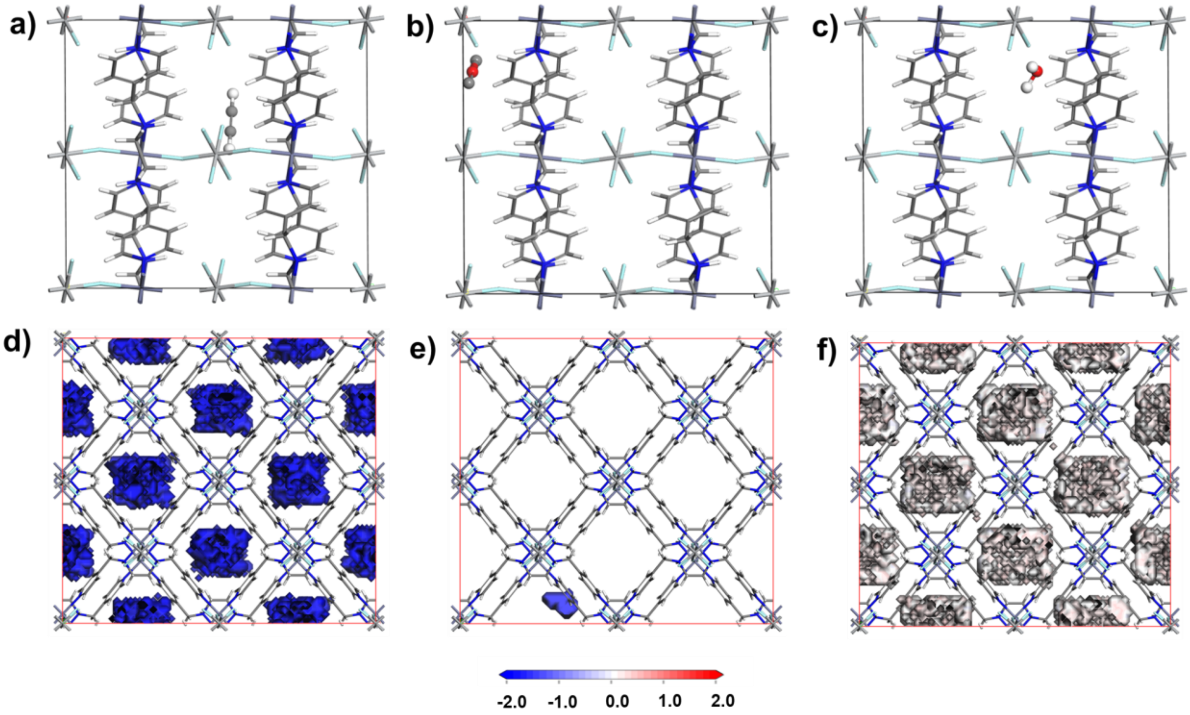

Figure S57: Sorbate binding sites: **(a)**–**(c)** show **TIFSIX-enmepy-Zn** loaded with C_2H_2, CO_2, and H_2O, respectively, using the lowest-energy loaded structures obtained from CMC calculations. **(d)**–**(f)** illustrate combined visualisations of C_2H_2, CO_2, and H_2O energy and density distributions. The isosurfaces represent constant density (isovalue = 0.0003) and are coloured by potential energy. The colour map values indicate the adsorbate potential energy in kcal/mol.

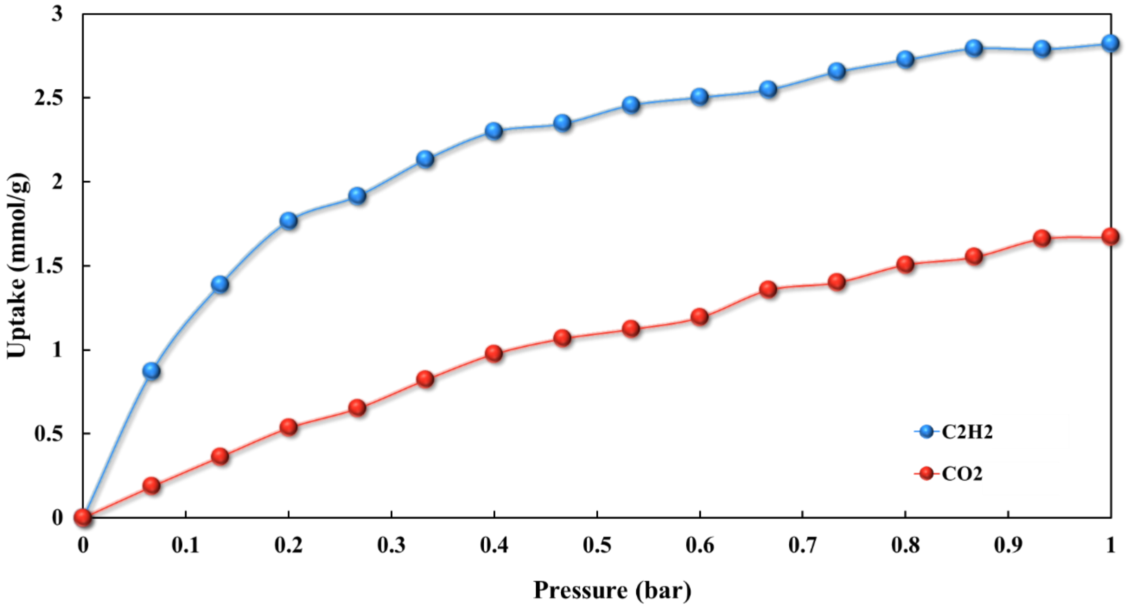

Figure S578: Adsorption isotherms obtained for GCMC calculations for **NbOFFIVE-enmepy-Zn** of C_2H_2 and CO_2 at 298 K.

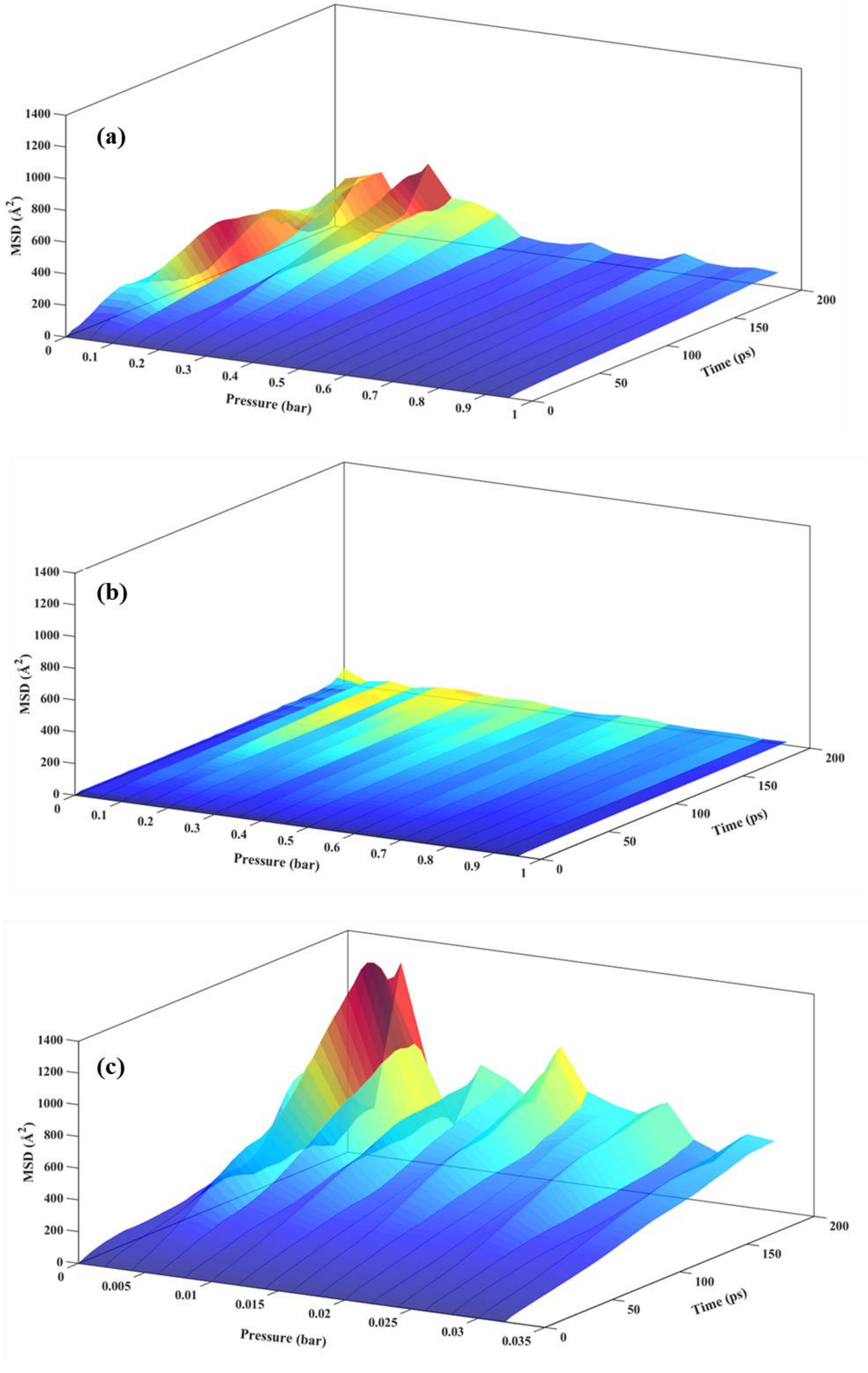

Figure S59: Three-dimensional mean square displacement graph of (a) C_2H_2, (b) CO_2, and (c) H_2O in **NbOFFIVE-enmepy-Zn** as a function of pressure and time at 298 K.

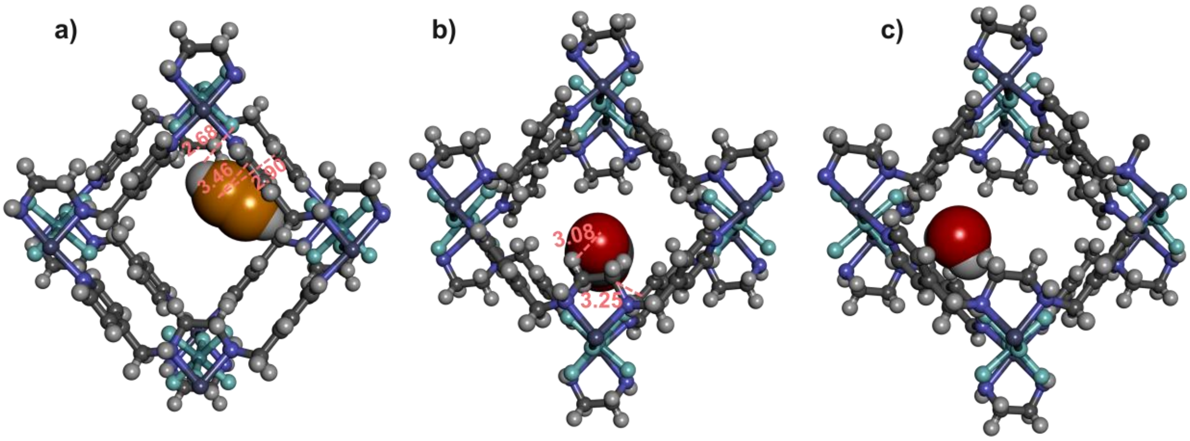

Figure S60: Optimised binding sites of (a) C_2H_2 and (b) CO_2 and c) H_2O inside **NbOFFIVE-enmepy-Zn**. The closest contacts between the framework atoms and adsorbates are indicated by distances (in Å). **NbOFFIVE-enmepy-Zn** is shown in ball-and-stick style, whereas the adsorbate molecules are shown in a space-filling style. Colour codes: N, blue; Zn, turquoise; Nb, dark teal; F, green; O, red; H, grey; C, dark grey, and C (C_2H_2), orange.

References

1. C. Legein, M. Body, J. Lhoste, W. Li, T. Charpentier, & D. Dambournet, Synthesis, crystal structure and 19F NMR parameters modelling of CaTiF6(H2O)2 yielding to a revision of the bond-valence parameters for the Ti4+/F− ion pair. *Journal of Solid State Chemistry*, **319** (2023) 123793. https://doi.org/10.1016/j.jssc.2022.123793.
2. R. Hoshino & S. Adachi, Optical spectroscopy of ZnSiF6·6H2O:Mn4+ red phosphor. *Journal of Applied Physics*, **114** (2013). https://doi.org/10.1063/1.4836896.
3. R. L. Davidovich, T. F. Levchishina, T. A. Kaidalova, & V. I. Sergienko, The synthesis and properties of oxofluoroniobates and fluorotantalates of bivalent metals. *Journal of the Less Common Metals*, **27** (1972) 35–43. https://doi.org/10.1016/0022-5088(72)90102-6.
4. B.-L. Fei, W.-Y. Sun, K.-B. Yu, & W.-X. Tang, Construction of co-ordination networks of 1,6-bis(4′-pyridyl)-2,5-diazahexane with silver(I) and copper(I). Structural diversity through change in metal ions and counter ions. *Journal of the Chemical Society, Dalton Transactions*, (2000) 805–811. https://doi.org/10.1039/a908910a.
5. D.-P. Li, J. Li, L. I. Xu, C. Ping-Hua, J. Xiao-Yan, & Y.-X. Li, Solvothermal Synthesis and Crystal Structure of a Zn(II) Microporous Metal-organic Framework. *Chinese J. Struct. Chem*, **31** (2012) 31–42. https://doi.org/10.14102/j.cnki.0254-5861.2012.11.021.
6. G.-F. Zhang, S.-M. Zhao, J.-B. She, & S. W. Ng, Poly[[bis(μ $_2$ -3,5-diamino-1,2,4-triazole)-κ ^{2} *N* ^{2} : *N* ^{4} ;κ ^{2} *N* ^{4} : *N* ^{2} -μ $_2$ -sulfato-κ ^{2} *O* : *O* ′-cadmium(II)] monohydrate]. *Acta Crystallographica Section E Structure Reports Online*, **62** (2006) m2148–m2150. https://doi.org/10.1107/S1600536806029096.
7. K.-J. Chen, H. S. Scott, D. G. Madden, T. Pham, A. Kumar, A. Bajpai, M. Lusi, K. A. Forrest, B. Space, J. J. Perry, & M. J. Zaworotko, Benchmark C2H2/CO2 and CO2/C2H2 Separation by Two Closely Related Hybrid Ultramicroporous Materials. *Chem*, **1** (2016) 753–765. https://doi.org/10.1016/j.chempr.2016.10.009.
8. L. Yang, A. Jin, L. Ge, X. Cui, & H. Xing, A novel interpenetrated anion-pillared porous material with high water tolerance afforded efficient C_2H_2/C_2H_4 separation. *Chemical Communications*, **55** (2019) 5001–5004. https://doi.org/10.1039/C9CC00976K.
9. L. Yang, X. Cui, Z. Zhang, Q. Yang, Z. Bao, Q. Ren, & H. Xing, An Asymmetric Anion-Pillared Metal–Organic Framework as a Multisite Adsorbent Enables Simultaneous Removal of Propyne and Propadiene from Propylene. *Angewandte Chemie International Edition*, **57** (2018) 13145–13149. https://doi.org/10.1002/anie.201807652.
10. M.-J. Lin, A. Jouaiti, N. Kyritsakas, & M. W. Hosseini, Molecular tectonics: modulation of size and shape of cuboid 3-D coordination networks. *CrystEngComm*, **11** (2009) 189–191. https://doi.org/10.1039/B815695F.
11. P. Nugent, Y. Belmabkhout, S. D. Burd, A. J. Cairns, R. Luebke, K. Forrest, T. Pham, S. Ma, B. Space, L. Wojtas, M. Eddaoudi, & M. J. Zaworotko, Porous materials with optimal adsorption thermodynamics and kinetics for CO2 separation. *Nature*, **495** (2013) 80–84. https://doi.org/10.1038/nature11893.

12. X. Cui, K. Chen, H. Xing, Q. Yang, R. Krishna, Z. Bao, H. Wu, W. Zhou, X. Dong, Y. Han, B. Li, Q. Ren, M. J. Zaworotko, & B. Chen, Pore chemistry and size control in hybrid porous materials for acetylene capture from ethylene. *Science*, **353** (2016) 141–144. https://doi.org/10.1126/science.aaf2458.

13. Z. Zhang, Q. Yang, X. Cui, L. Yang, Z. Bao, Q. Ren, & H. Xing, Sorting of C_4 Olefins with Interpenetrated Hybrid Ultramicroporous Materials by Combining Molecular Recognition and Size-Sieving. *Angewandte Chemie International Edition*, **56** (2017) 16282–16287. https://doi.org/10.1002/anie.201708769.

14. X. Cui, Q. Yang, L. Yang, R. Krishna, Z. Zhang, Z. Bao, H. Wu, Q. Ren, W. Zhou, B. Chen, & H. Xing, Ultrahigh and Selective SO_2 Uptake in Inorganic Anion-Pillared Hybrid Porous Materials. *Advanced Materials*, **29** (2017). https://doi.org/10.1002/adma.201606929.

15. M. Jiang, X. Cui, L. Yang, Q. Yang, Z. Zhang, Y. Yang, & H. Xing, A thermostable anion-pillared metal-organic framework for C2H2/C2H4 and C2H2/CO2 separations. *Chemical Engineering Journal*, **352** (2018) 803–810. https://doi.org/10.1016/j.cej.2018.07.104.

16. Q. Wang, L. Yang, T. Ke, J. Hu, X. Suo, X. Cui, & H. Xing, Selective sorting of hexane isomers by anion-functionalized metal-organic frameworks with optimal energy regulation. *Nature Communications*, **15** (2024) 2620. https://doi.org/10.1038/s41467-024-46738-2.

17. H. S. Scott, A. Bajpai, K.-J. Chen, T. Pham, B. Space, J. J. Perry, & M. J. Zaworotko, Novel mode of 2-fold interpenetration observed in a primitive cubic network of formula [Ni(1,2-bis(4-pyridyl)acetylene)$_2$(Cr$_2$O$_7$)]$_n$. *Chemical Communications*, **51** (2015) 14832–14835. https://doi.org/10.1039/C5CC05866J.

18. A. Bajpai, M. Lusi, & M. J. Zaworotko, The role of weak interactions in controlling the mode of interpenetration in hybrid ultramicroporous materials. *Chemical Communications*, **53** (2017) 3978–3981. https://doi.org/10.1039/C6CC10217D.

19. M. Shivanna, K. Otake, B. Song, L. M. van Wyk, Q. Yang, N. Kumar, W. K. Feldmann, T. Pham, S. Suepaul, B. Space, L. J. Barbour, S. Kitagawa, & M. J. Zaworotko, Benchmark Acetylene Binding Affinity and Separation through Induced Fit in a Flexible Hybrid Ultramicroporous Material. *Angewandte Chemie International Edition*, **60** (2021) 20383–20390. https://doi.org/10.1002/anie.202106263.

20. M.-Y. Gao, A. A. Bezrukov, B.-Q. Song, M. He, S. J. Nikkhah, S.-Q. Wang, N. Kumar, S. Darwish, D. Sensharma, C. Deng, J. Li, L. Liu, R. Krishna, M. Vandichel, S. Yang, & M. J. Zaworotko, Highly Productive C_3H_4/C_3H_6 Trace Separation by a Packing Polymorph of a Layered Hybrid Ultramicroporous Material. *Journal of the American Chemical Society*, **145** (2023) 11837–11845. https://doi.org/10.1021/jacs.3c03505.

21. L. Carlucci, G. Ciani, D. M. Proserpio, & S. Rizzato, New architectures from the self-assembly of MIISO4 salts with bis(4-pyridyl) ligands. The first case of polycatenation involving three distinct sets of 2D polymeric (4,4)-layers parallel to a common axis. *CrystEngComm*, **5** (2003) 190. https://doi.org/10.1039/b304201d.

22. L. Carlucci, G. Ciani, D. M. Proserpio, & S. Rizzato, New examples of self-catenation in two three-dimensional polymeric co-ordination networks†. *Journal of the Chemical Society, Dalton Transactions*, (2000) 3821–3828. https://doi.org/10.1039/b003092i.
23. M.-C. Suen, Z.-K. Chan, J.-D. Chen, J.-C. Wang, & C.-H. Hung, Syntheses and structures of three new coordination polymers generated from the flexible 1,3-bis(4-pyridyl)propane ligand and zinc salts. *Polyhedron*, **25** (2006) 2325–2332. https://doi.org/10.1016/j.poly.2006.01.031.
24. T. Kurihara, K. Ohara, K. Kadota, H. Izu, Y. Nishiyama, M. Mizuno, & S. Horike, Three-Dimensional Metal–Organic Network Glasses from Bridging MF_6^{2-} Anions and Their Dynamic Insights by Solid-State NMR. *Inorganic Chemistry*, **61** (2022) 16103–16109. https://doi.org/10.1021/acs.inorgchem.2c02580.
25. X. Li, L. Yang, T. Su, X. Wang, C. Sun, & Z. Su, Graphene-coated hybrid electrocatalysts derived from bimetallic metal–organic frameworks for efficient hydrogen generation. *Journal of Materials Chemistry A*, **5** (2017) 5000–5006. https://doi.org/10.1039/C6TA10405C.
26. J. Othong, J. Boonmak, & S. Youngme, Highly selective Cr2O72- removal in aqueous medium by using a flexible 2D metal-organic framework through single-crystal-to-single-crystal transformation. *Journal of Environmental Chemical Engineering*, **7** (2019) 102998. https://doi.org/10.1016/j.jece.2019.102998.
27. L. Carlucci, G. Ciani, M. Moret, D. M. Proserpio, & S. Rizzato, Polymeric layers catenated by ribbons of rings in a three-dimensional self-assembled architecture: A nanoporous network with spongelike behavior. *Angewandte Chemie - International Edition*, **39** (2000) 1506–1510. https://doi.org/10.1002/(SICI)1521-3773(20000417)39:8<1506::AID-ANIE1506>3.0.CO;2-U.
28. S. D. Burd, S. Ma, J. A. Perman, B. J. Sikora, R. Q. Snurr, P. K. Thallapally, J. Tian, L. Wojtas, & M. J. Zaworotko, Highly Selective Carbon Dioxide Uptake by [Cu(bpy- *n*)$_2$ (SiF$_6$)] (bpy-1 = 4,4′-Bipyridine; bpy-2 = 1,2-Bis(4-pyridyl)ethene). *Journal of the American Chemical Society*, **134** (2012) 3663–3666. https://doi.org/10.1021/ja211340t.
29. L. Yang, X. Cui, Y. Zhang, Q. Wang, Z. Zhang, X. Suo, & H. Xing, Anion Pillared Metal–Organic Framework Embedded with Molecular Rotors for Size-Selective Capture of CO_2 from CH_4 and N_2. *ACS Sustainable Chemistry & Engineering*, **7** (2019) 3138–3144. https://doi.org/10.1021/acssuschemeng.8b04916.
30. N. Xu, T. Yan, J. Li, L. Wang, D. Liu, & Y. Zhang, A new TIFSIX anion pillared metal organic framework with abundant electronegative sites for efficient C_2H_2/CO_2 separation. *Inorganic Chemistry Frontiers*, **10** (2023) 522–528. https://doi.org/10.1039/D2QI01989B.
31. D. Hagrman, R. P. Hammond, R. Haushalter, & J. Zubieta, Organic/Inorganic Composite Materials: Hydrothermal Syntheses and Structures of the One-, Two-, and Three-Dimensional Copper(II) Sulfate−Organodiamine Phases [Cu(H_2O)$_3$ (4,4‘-bipyridine)(SO_4)]·2H_2O, [Cu(bpe)$_2$][Cu(bpe)(H_2O)$_2$ (SO_4)$_2$]·2H_2O, and [Cu(bpe)(H_2O)(SO_4)] (bpe = *trans*- 1,2-Bis(4-pyridyl)ethylene). *Chemistry of Materials*, **10** (1998) 2091–2100. https://doi.org/10.1021/cm9707566.

32. M. H. Mohamed, S. K. Elsaidi, T. Pham, K. A. Forrest, B. Tudor, L. Wojtas, B. Space, & M. J. Zaworotko, Pillar substitution modulates CO2 affinity in “mmo” topology networks. *Chemical Communications*, **49** (2013) 9809. https://doi.org/10.1039/c3cc44745f.
33. M. H. Mohamed, S. K. Elsaidi, L. Wojtas, T. Pham, K. A. Forrest, B. Tudor, B. Space, & M. J. Zaworotko, Highly Selective CO_2 Uptake in Uninodal 6-Connected “mmo” Nets Based upon $MO_4{}^{2-}$ (M = Cr, Mo) Pillars. *Journal of the American Chemical Society*, **134** (2012) 19556–19559. https://doi.org/10.1021/ja309452y.
34. D. O’Nolan, A. Kumar, & M. J. Zaworotko, Water Vapor Sorption in Hybrid Pillared Square Grid Materials. *Journal of the American Chemical Society*, **139** (2017) 8508–8513. https://doi.org/10.1021/jacs.7b01682.
35. B. Li, X. Cui, D. O’Nolan, H. Wen, M. Jiang, R. Krishna, H. Wu, R. Lin, Y. Chen, D. Yuan, H. Xing, W. Zhou, Q. Ren, G. Qian, M. J. Zaworotko, & B. Chen, An Ideal Molecular Sieve for Acetylene Removal from Ethylene with Record Selectivity and Productivity. *Advanced Materials*, **29** (2017). https://doi.org/10.1002/adma.201704210.
36. Z. Zhang, Q. Yang, X. Cui, L. Yang, Z. Bao, Q. Ren, & H. Xing, Sorting of C_4 Olefins with Interpenetrated Hybrid Ultramicroporous Materials by Combining Molecular Recognition and Size-Sieving. *Angewandte Chemie International Edition*, **56** (2017) 16282–16287. https://doi.org/10.1002/anie.201708769.
37. L. Yang, X. Cui, Y. Zhang, Q. Yang, & H. Xing, A highly sensitive flexible metal–organic framework sets a new benchmark for separating propyne from propylene. *Journal of Materials Chemistry A*, **6** (2018) 24452–24458. https://doi.org/10.1039/C8TA08198K.
38. M. Jiang, B. Li, X. Cui, Q. Yang, Z. Bao, Y. Yang, H. Wu, W. Zhou, B. Chen, & H. Xing, Controlling Pore Shape and Size of Interpenetrated Anion-Pillared Ultramicroporous Materials Enables Molecular Sieving of CO_2 Combined with Ultrahigh Uptake Capacity. *ACS Applied Materials & Interfaces*, **10** (2018) 16628–16635. https://doi.org/10.1021/acsami.8b03358.
39. D. Sensharma, S. Vaesen, C. Healy, J. Hartmann, A. C. Kathalikkattil, P. Wix, F. Steuber, N. Zhu, & W. Schmitt, CO_2 Adsorption in SIFSIX-14-Cu-i: High Performance, Inflected Isotherms, and Water-Triggered Release via Reversible Structural Transformation. *European Journal of Inorganic Chemistry*, **2018** (2018) 1993–1997. https://doi.org/10.1002/ejic.201800217.
40. Q. Wang, J. Hu, L. Yang, Z. Zhang, T. Ke, X. Cui, & H. Xing, One-step removal of alkynes and propadiene from cracking gases using a multi-functional molecular separator. *Nature Communications*, **13** (2022) 2955. https://doi.org/10.1038/s41467-022-30408-2.
41. H. S. Scott, N. Ogiwara, K.-J. Chen, D. G. Madden, T. Pham, K. Forrest, B. Space, S. Horike, J. J. Perry IV, S. Kitagawa, & M. J. Zaworotko, Crystal engineering of a family of hybrid ultramicroporous materials based upon interpenetration and dichromate linkers. *Chemical Science*, **7** (2016) 5470–5476. https://doi.org/10.1039/C6SC01385F.
42. B. Manna, S. Sharma, & S. Ghosh, Synthesis and Crystal Structure of a Zn(II)-Based MOF Bearing Neutral N-Donor Linker and SiF62− Anion. *Crystals*, **8** (2018) 37. https://doi.org/10.3390/cryst8010037.

43. Jian Luan, CCDC 2327168: Experimental Crystal Structure Determination. *CSD Communication*, (2024).
44. R.-B. Lin, L. Li, H. Wu, H. Arman, B. Li, R.-G. Lin, W. Zhou, & B. Chen, Optimized Separation of Acetylene from Carbon Dioxide and Ethylene in a Microporous Material. *Journal of the American Chemical Society*, **139** (2017) 8022–8028. https://doi.org/10.1021/jacs.7b03850.
45. J. Wang, Y. Zhang, P. Zhang, J. Hu, R.-B. Lin, Q. Deng, Z. Zeng, H. Xing, S. Deng, & B. Chen, Optimizing Pore Space for Flexible-Robust Metal–Organic Framework to Boost Trace Acetylene Removal. *Journal of the American Chemical Society*, **142** (2020) 9744–9751. https://doi.org/10.1021/jacs.0c02594.
46. T. Ke, Q. Wang, J. Shen, J. Zhou, Z. Bao, Q. Yang, & Q. Ren, Molecular Sieving of C_2-C_3 Alkene from Alkyne with Tuned Threshold Pressure in Robust Layered Metal–Organic Frameworks. *Angewandte Chemie International Edition*, **59** (2020) 12725–12730. https://doi.org/10.1002/anie.202003421.
47. J. Shen, X. He, T. Ke, R. Krishna, J. M. van Baten, R. Chen, Z. Bao, H. Xing, M. Dincă, Z. Zhang, Q. Yang, & Q. Ren, Simultaneous interlayer and intralayer space control in two-dimensional metal−organic frameworks for acetylene/ethylene separation. *Nature Communications*, **11** (2020) 6259. https://doi.org/10.1038/s41467-020-20101-7.
48. J. Wang, Y. Zhang, P. Zhang, J. Hu, R.-B. Lin, Q. Deng, Z. Zeng, H. Xing, S. Deng, & B. Chen, Optimizing Pore Space for Flexible-Robust Metal–Organic Framework to Boost Trace Acetylene Removal. *Journal of the American Chemical Society*, **142** (2020) 9744–9751. https://doi.org/10.1021/jacs.0c02594.
49. J. Wang, Y. Zhang, Y. Su, X. Liu, P. Zhang, R.-B. Lin, S. Chen, Q. Deng, Z. Zeng, S. Deng, & B. Chen, Fine pore engineering in a series of isoreticular metal-organic frameworks for efficient C2H2/CO2 separation. *Nature Communications*, **13** (2022) 200. https://doi.org/10.1038/s41467-021-27929-7.
50. J. Zhu, T. Ke, L. Yang, Z. Bao, Z. Zhang, B. Su, Q. Ren, & Q. Yang, Optimizing Trace Acetylene Removal from Acetylene/Ethylene Mixture in a Flexible Metal–Organic Framework by Crystal Downsizing. *ACS Applied Materials & Interfaces*, **16** (2024) 22455–22464. https://doi.org/10.1021/acsami.4c03517.
51. Y. Niu, Z. Li, Y. Song, M. Tang, B. Wu, & X. Xin, Three-dimensional molecular network, [{Cu(dps)2(SO4)}·3H2O·DMF]n, and its different third-order NLO performance (dps=4,4′-dipyridyl sulfide). *Journal of Solid State Chemistry*, **179** (2006) 4003–4010. https://doi.org/10.1016/j.jssc.2006.09.009.
52. X.-C. Su, S. Zhu, H.-K. Lin, X.-B. Leng, & Y.-T. Chen, Engineering of non-interpenetrating 3-D and 2-D networks and guest adsorption based on 4,4'-di(3-methyl)pyridyl sulfide copper(ii) complexes. *Journal of the Chemical Society, Dalton Transactions*, (2001) 3163–3168. https://doi.org/10.1039/b104515f.
53. J. Shen, X. He, T. Ke, R. Krishna, J. M. van Baten, R. Chen, Z. Bao, H. Xing, M. Dincă, Z. Zhang, Q. Yang, & Q. Ren, Simultaneous interlayer and intralayer space control in two-dimensional

metal−organic frameworks for acetylene/ethylene separation. *Nature Communications*, **11** (2020) 6259. https://doi.org/10.1038/s41467-020-20101-7.

54. T. Ke, Q. Wang, X. Zhu, J. Hu, Z. Bao, Z. Zhang, Q. Ren, & Q. Yang, High-capacity dynamic exclusion for highly efficient dilute C2H2 separation from CO2 and multicomponent mixture in robust ultramicroporous MOF by topology regulation. *Chemical Engineering Journal*, **472** (2023) 144852. https://doi.org/10.1016/j.cej.2023.144852.

55. Y. Song, T. Ke, J. Shen, J. Li, X. Zhu, L. Yang, Z. Zhang, Z. Bao, Q. Ren, & Q. Yang, Shaped layered two-dimensional fluorinated metal-organic frameworks for highly efficient acetylene/ethylene separation. *Separation and Purification Technology*, **323** (2023) 124377. https://doi.org/10.1016/j.seppur.2023.124377.

56. M.-C. Suen & J.-C. Wang, Syntheses and structural characterization of inifinite coordination polymers from bipyridyl ligands and zinc salts. *Structural Chemistry*, **17** (2006) 315–322. https://doi.org/10.1007/s11224-006-9049-6.

57. H. Xiong, Y. Peng, X. Liu, P. Wang, P. Zhang, L. Yang, J. Liu, H. Shuai, L. Wang, Z. Deng, S. Chen, J. Chen, Z. Zhou, S. Deng, & J. Wang, Topology Reconfiguration of Anion‐Pillared Metal–Organic Framework from Flexibility to Rigidity for Enhanced Acetylene Separation. *Advanced Materials*, **36** (2024). https://doi.org/10.1002/adma.202401693.

58. F. Zheng, R. Chen, Z. Ding, Y. Liu, Z. Zhang, Q. Yang, Y. Yang, Q. Ren, & Z. Bao, Interlayer Symmetry Control in Flexible-Robust Layered Metal–Organic Frameworks for Highly Efficient C $_2$ H $_2$ /CO $_2$ Separation. *Journal of the American Chemical Society*, **145** (2023) 19903–19911. https://doi.org/10.1021/jacs.3c06138.

59. J. Luo, M. Hong, R. Wang, D. Yuan, R. Cao, L. Han, Y. Xu, & Z. Lin, Self-Assembly of Three Cd ^{II} - and Cu ^{II} -Containing Coordination Polymers from 4,4′-Dipyridyl Disulfide. *European Journal of Inorganic Chemistry*, **2003** (2003) 3623–3632. https://doi.org/10.1002/ejic.200300153.

60. F. Zheng, R. Chen, Y. Liu, Q. Yang, Z. Zhang, Y. Yang, Q. Ren, & Z. Bao, Strengthening Intraframework Interaction within Flexible MOFs Demonstrates Simultaneous Sieving Acetylene from Ethylene and Carbon Dioxide. *Advanced Science*, **10** (2023). https://doi.org/10.1002/advs.202207127.

61. F. Zheng, L. Guo, R. Chen, L. Chen, Z. Zhang, Q. Yang, Y. Yang, B. Su, Q. Ren, & Z. Bao, Shell‐like Xenon Nano‐Traps within Angular Anion‐Pillared Layered Porous Materials for Boosting Xe/Kr Separation. *Angewandte Chemie International Edition*, **61** (2022). https://doi.org/10.1002/anie.202116686.

62. M.-J. Lin, A. Jouaiti, D. Pocic, N. Kyritsakas, J.-M. Planeix, & M. W. Hosseini, Molecular tectonics: tubular crystals with controllable channel size and orientation. *Chem. Commun.*, **46** (2010) 112–114. https://doi.org/10.1039/B915665H.

63. M.-J. Lin, A. Jouaiti, N. Kyritsakas, & M. W. Hosseini, Molecular tectonics: modulation of size and shape of cuboid 3-D coordination networks. *CrystEngComm*, **11** (2009) 189–191. https://doi.org/10.1039/B815695F.

64. A. Bajpai, D. O'Nolan, D. G. Madden, K.-J. Chen, T. Pham, A. Kumar, M. Lusi, J. J. Perry, B. Space, & M. J. Zaworotko, The effect of centred versus offset interpenetration on C $_2$ H $_2$ sorption

in hybrid ultramicroporous materials. *Chem. Commun.*, **53** (2017) 11592–11595. https://doi.org/10.1039/C7CC05882A.

65. Y. Han, Y. Jiang, J. Hu, L. Wang, & Y. Zhang, Efficient C2H2/CO2 and C2H2/C2H4 separations in a novel fluorinated metal–organic framework. *Separation and Purification Technology*, **332** (2024) 125777. https://doi.org/10.1016/j.seppur.2023.125777.

66. H. S. Scott, M. Shivanna, A. Bajpai, D. G. Madden, K.-J. Chen, T. Pham, K. A. Forrest, A. Hogan, B. Space, J. J. Perry IV, & M. J. Zaworotko, Highly Selective Separation of C_2H_2 from CO_2 by a New Dichromate-Based Hybrid Ultramicroporous Material. *ACS Applied Materials & Interfaces*, **9** (2017) 33395–33400. https://doi.org/10.1021/acsami.6b15250.

67. H. S. Scott, M. Shivanna, A. Bajpai, K.-J. Chen, D. G. Madden, J. J. Perry IV, & M. J. Zaworotko, Enhanced Stability toward Humidity in a Family of Hybrid Ultramicroporous Materials Incorporating $Cr_2O_7^{2-}$ Pillars. *Crystal Growth & Design*, **17** (2017) 1933–1937. https://doi.org/10.1021/acs.cgd.6b01881.

68. O. Alduhaish, R.-B. Lin, H. Wang, B. Li, H. D. Arman, T.-L. Hu, & B. Chen, Metal–Organic Framework with Trifluoromethyl Groups for Selective C_2H_2 and CO_2 Adsorption. *Crystal Growth & Design*, **18** (2018) 4522–4527. https://doi.org/10.1021/acs.cgd.8b00506.

69. W. Liang, P. M. Bhatt, A. Shkurenko, K. Adil, G. Mouchaham, H. Aggarwal, A. Mallick, A. Jamal, Y. Belmabkhout, & M. Eddaoudi, A Tailor-Made Interpenetrated MOF with Exceptional Carbon-Capture Performance from Flue Gas. *Chem*, **5** (2019) 950–963. https://doi.org/10.1016/j.chempr.2019.02.007.

70. H.-M. Wen, C. Liao, L. Li, A. Alsalme, Z. Alothman, R. Krishna, H. Wu, W. Zhou, J. Hu, & B. Chen, A metal–organic framework with suitable pore size and dual functionalities for highly efficient post-combustion CO_2 capture. *Journal of Materials Chemistry A*, **7** (2019) 3128–3134. https://doi.org/10.1039/C8TA11596F.

71. M.-H. You, M.-H. Li, & M.-J. Lin, Two Face Diagonally Linked Cuboid Coordination Networks with Enhanced Thermal Stability. *Crystal Growth & Design*, **22** (2022) 1384–1389. https://doi.org/10.1021/acs.cgd.1c01334.

72. L. Li, R. Matsuda, I. Tanaka, H. Sato, P. Kanoo, H. J. Jeon, M. L. Foo, A. Wakamiya, Y. Murata, & S. Kitagawa, A Crystalline Porous Coordination Polymer Decorated with Nitroxyl Radicals Catalyzes Aerobic Oxidation of Alcohols. *Journal of the American Chemical Society*, **136** (2014) 7543–7546. https://doi.org/10.1021/ja5019095.

73. P. Larpent, A. Jouaiti, N. Kyritsakas, & M. W. Hosseini, Molecular tectonics: homochiral 3D cuboid coordination networks based on enantiomerically pure organic tectons and ZnSiF6. *Chemical Communications*, **49** (2013) 4468. https://doi.org/10.1039/c3cc41140k.

74. E. M. Ketatni, N. Kyritsakas, P. Mobian, & A. Jouaiti, Synthesis of coordination polymers based on a 2,2′-dimethoxy-1,1′-biphenyl scaffold and Hg(II), Co(II), or Zn(II). *Journal of Molecular Structure*, **1248** (2022) 131466. https://doi.org/10.1016/j.molstruc.2021.131466.

75. M.-H. You, M.-H. Li, & M.-J. Lin, Two Face Diagonally Linked Cuboid Coordination Networks with Enhanced Thermal Stability. *Crystal Growth & Design*, **22** (2022) 1384–1389. https://doi.org/10.1021/acs.cgd.1c01334.

76. M.-J. Lin, A. Jouaiti, N. Kyritsakas, & M. W. Hosseini, Molecular tectonics: control of interpenetration in cuboid 3-D coordination networks. *CrystEngComm*, **13** (2011) 776–778. https://doi.org/10.1039/C0CE00777C.
77. G. Gupta, A. Paul, A. Gupta, J. Lee, & C. Y. Lee, Removal of organic dyes from aqueous solution using a novel pyrene appended Zn(<scp>ii</scp>)-based metal–organic framework and its photocatalytic properties. *Dalton Transactions*, **53** (2024) 15732–15741. https://doi.org/10.1039/D4DT01869A.
78. S. I. Vasylevskyi, D. M. Bassani, & K. M. Fromm, Anion-Induced Structural Diversity of Zn and Cd Coordination Polymers Based on Bis-9,10-(pyridine-4-yl)-anthracene, Their Luminescent Properties, and Highly Efficient Sensing of Nitro Derivatives and Herbicides. *Inorganic Chemistry*, **58** (2019) 5646–5653. https://doi.org/10.1021/acs.inorgchem.8b03628.
79. A. C. K. M. Agonist Kastrati, CCDC 1991434: Experimental Crystal Structure Determination. *CSD Communication*, (2024).
80. H. S. Quah, V. Nalla, K. Zheng, C. A. Lee, X. Liu, & J. J. Vittal, Tuning Two-Photon Absorption Cross Section in Metal Organic Frameworks. *Chemistry of Materials*, **29** (2017) 7424–7430. https://doi.org/10.1021/acs.chemmater.7b02417.
81. Q. Yu, H. H. Y. Sung, F. Gao, I. D. Williams, J. W. Y. Lam, J. Sun, & B. Z. Tang, Ligand *Meta* -Anchoring Strategy in Metal–Organic Frameworks for Remarkable Promotion of Quantum Yields. *Angewandte Chemie International Edition*, **63** (2024). https://doi.org/10.1002/anie.202401261.
82. S. Noro, R. Kitaura, M. Kondo, S. Kitagawa, T. Ishii, H. Matsuzaka, & M. Yamashita, Framework Engineering by Anions and Porous Functionalities of Cu(II)/4,4'-bpy Coordination Polymers. *Journal of the American Chemical Society*, **124** (2002) 2568–2583. https://doi.org/10.1021/ja0113192.
83. H. Lin & P. A. Maggard, Microporosity, Optical Bandgap Sizes, and Photocatalytic Activity of M(I)-Nb(V) (M = Cu, Ag) Oxyfluoride Hybrids. *Crystal Growth & Design*, **10** (2010) 1323–1331. https://doi.org/10.1021/cg9013625.
84. S. D. Burd, S. Ma, J. A. Perman, B. J. Sikora, R. Q. Snurr, P. K. Thallapally, J. Tian, L. Wojtas, & M. J. Zaworotko, Highly Selective Carbon Dioxide Uptake by $[Cu(bpy\text{-}n)_2(SiF_6)]$ (bpy-1 = 4,4′-Bipyridine; bpy-2 = 1,2-Bis(4-pyridyl)ethene). *Journal of the American Chemical Society*, **134** (2012) 3663–3666. https://doi.org/10.1021/ja211340t.
85. P. Nugent, V. Rhodus, T. Pham, B. Tudor, K. Forrest, L. Wojtas, B. Space, & M. Zaworotko, Enhancement of CO2 selectivity in a pillared pcu MOM platform through pillar substitution. *Chemical Communications*, **49** (2013) 1606. https://doi.org/10.1039/c3cc37695h.
86. E. Aubert, A. Doudouh, P. Peluso, & V. Mamane, Channels with ordered water and bipyridine molecules in the porous coordination polymer $\{[Cu(SiF_6)(C_{10}H_8N_2)_2]\cdot 2C_{10}N_2H_8\cdot 5H_2O\}_n$. *Acta Crystallographica Section E Crystallographic Communications*, **72** (2016) 1654–1658. https://doi.org/10.1107/S2056989016016686.
87. X. Cui, Q. Yang, L. Yang, R. Krishna, Z. Zhang, Z. Bao, H. Wu, Q. Ren, W. Zhou, B. Chen, & H. Xing, Ultrahigh and Selective SO_2 Uptake in Inorganic Anion-Pillared Hybrid Porous Materials. *Advanced Materials*, **29** (2017). https://doi.org/10.1002/adma.201606929.

88. X. Cui, Z. Niu, C. Shan, L. Yang, J. Hu, Q. Wang, P. C. Lan, Y. Li, L. Wojtas, S. Ma, & H. Xing, Efficient separation of xylene isomers by a guest-responsive metal–organic framework with rotational anionic sites. *Nature Communications*, **11** (2020) 5456. https://doi.org/10.1038/s41467-020-19209-7.
89. Y. Shen, K. Sugimoto, S. Yamashita, T. Yoshida, Y. Nakazawa, B. K. Breedlove, H. Zhang, & M. Yamashita, Melamine-induced synthesis of a structurally perfect kagomé antiferromagnet. *Chemical Communications*, **58** (2022) 3763–3766. https://doi.org/10.1039/D2CC00416J.
90. S. Subramanian & M. J. Zaworotko, Porous Solids by Design: [Zn(4,4′-bpy)$_2$(SiF$_6$)]$_n$ · *x* DMF, a Single Framework Octahedral Coordination Polymer with Large Square Channels. *Angewandte Chemie International Edition in English*, **34** (1995) 2127–2129. https://doi.org/10.1002/anie.199521271.
91. S. I. Noro, S. Kitagawa, M. Kondo, & K. Seki, A new, methane adsorbent, porous coordination polymer [{CuSiF6(4,4'- bipyridine)2}(n)]. *Angewandte Chemie - International Edition*, **39** (2000) 2082–2084. https://doi.org/10.1002/1521-3773(20000616)39:12<2081::aid-anie2081>3.0.co;2-a.
92. A. L. Kopf, P. A. Maggard, C. L. Stern, & K. R. Poeppelmeier, Poly[nickel(II)-di-μ-4,4′-bipyridyl-κ^4 *N*: *N* ′-μ-dichromato-κ^2 *O*: *O* ′] and poly[copper(II)-di-μ-4,4'-bipyridyl-κ^4 *N*: *N* ′-μ-dichromato-κ^2 *O*: *O* ′]. *Acta Crystallographica Section C Crystal Structure Communications*, **61** (2005) m165–m168. https://doi.org/10.1107/S0108270105001605.
93. X. Chen, B. Zhao, P. Cheng, B. Ding, D. Liao, S. Yan, & Z. Jiang, Multi-Dimensional Systems Built from Dichromate Anions − Syntheses, Crystal Structures, and Magnetic Properties. *European Journal of Inorganic Chemistry*, **2004** (2004) 562–569. https://doi.org/10.1002/ejic.200300410.
94. W.-P. Zhong, Q.-W. Chen, X.-X. Cao, S.-L. Liu, Q. Chen, & N. Lin, A zinc(II) sulfate-based porous framework with new topology and its usage as catalyst in Friedel–Crafts alkylation reaction. *Inorganic Chemistry Communications*, **119** (2020) 108037. https://doi.org/10.1016/j.inoche.2020.108037.
95. Y. Hayashi, T. Tagami, H. Mano, & A. Uehara, Three-Dimensional Polymers with Metal–Oxygen and Metal–Ligand Chains: [Mn(II)(4,4′-bipy)2(Cr2O7)(H2O)2] and [Co(II)(4,4′-bipy)2(Cr2O7)]. *Chemistry Letters*, **30** (2001) 562–563. https://doi.org/10.1246/cl.2001.562.
96. D. Bradshaw, J. E. Warren, & M. J. Rosseinsky, Reversible Concerted Ligand Substitution at Alternating Metal Sites in an Extended Solid. *Science*, **315** (2007) 977–980. https://doi.org/10.1126/science.1135445.
97. R. L. LaDuca, M. Desiak, R. S. Rarig, & J. Zubieta, A bimetallic oxide hybrid material constructed from a coordination complex polymer and molybdenum oxide subunits, [Ni(3,4′-bipyridine)2MoO4]·3H2O. *Inorganica Chimica Acta*, **332** (2002) 79–86. https://doi.org/10.1016/S0020-1693(02)00705-3.
98. Q. Dong, Y. Huang, K. Hyeon-Deuk, I. Chang, J. Wan, C. Chen, J. Duan, W. Jin, & S. Kitagawa, Shape- and Size-Dependent Kinetic Ethylene Sieving from a Ternary Mixture by a Trap-and-

Flow Channel Crystal. *Advanced Functional Materials*, **32** (2022). https://doi.org/10.1002/adfm.202203745.

99. B.-Q. Song, Q.-Y. Yang, S.-Q. Wang, M. Vandichel, A. Kumar, C. Crowley, N. Kumar, C.-H. Deng, V. GasconPerez, M. Lusi, H. Wu, W. Zhou, & M. J. Zaworotko, Reversible Switching between Nonporous and Porous Phases of a New SIFSIX Coordination Network Induced by a Flexible Linker Ligand. *Journal of the American Chemical Society*, **142** (2020) 6896–6901. https://doi.org/10.1021/jacs.0c01314.

100. Q. Dong, Y. Huang, K. Hyeon-Deuk, I. Chang, J. Wan, C. Chen, J. Duan, W. Jin, & S. Kitagawa, Shape- and Size-Dependent Kinetic Ethylene Sieving from a Ternary Mixture by a Trap-and-Flow Channel Crystal. *Advanced Functional Materials*, **32** (2022). https://doi.org/10.1002/adfm.202203745.

101. N. Xu, J. Hu, L. Wang, D. Luo, W. Sun, Y. Hu, D. Wang, X. Cui, H. Xing, & Y. Zhang, A TIFSIX pillared MOF with unprecedented zsd topology for efficient separation of acetylene from quaternary mixtures. *Chemical Engineering Journal*, **450** (2022) 138034. https://doi.org/10.1016/j.cej.2022.138034.

102. Q. Dong, J. Wan, H. Chen, Y. Huang, & J. Duan, Highly Efficient CO_2 Capture from Wet–Hot Flue Gas by a Robust Trap-and-Flow Crystal. *ACS Applied Materials & Interfaces*, **15** (2023) 39606–39613. https://doi.org/10.1021/acsami.3c09456.

103. X. Wu, D. Li, L. Xu, Y.-F. Jiang, Y. Zhao, & J. Zhao, Construction of Cd(<scp>ii</scp>)-based metal–organic frameworks incorporating SiF_6^{2-} as fluorescence sensors for arginine. *CrystEngComm*, **25** (2023) 3222–3228. https://doi.org/10.1039/D3CE00302G.

104. S. Liu, Y. Huang, J. Wan, J.-J. Zheng, R. Krishna, Y. Li, K. Ge, J. Tang, & J. Duan, Fine-regulation of gradient gate-opening in nanoporous crystals for sieving separation of ternary C3 hydrocarbons. *Chemical Science*, **15** (2024) 6583–6588. https://doi.org/10.1039/D3SC05489F.

105. Y. Huang, J. Wan, T. Pan, K. Ge, Y. Guo, J. Duan, J. Bai, W. Jin, & S. Kitagawa, Delicate Softness in a Temperature-Responsive Porous Crystal for Accelerated Sieving of Propylene/Propane. *Journal of the American Chemical Society*, **145** (2023) 24425–24432. https://doi.org/10.1021/jacs.3c10277.

106. M. Guelfi, M. Taddei, & G. Bresciani, Ice Ih Shaped Water Cluster in the Cage of an Ultramicroporous Metal-Organic Framework. *Journal of Chemical Crystallography*, **55** (2025) 198–205. https://doi.org/10.1007/s10870-025-01048-3.

107. X. Yang & Q. Zhang, Syntheses, Crystal Structures, and Photocatalytic Activities of Three Copper Coordination Polymers Based on Bis(1H-imidazol-4-yl)benzene/3-(2-Pyridyl)pyrazole. *Zeitschrift für anorganische und allgemeine Chemie*, **642** (2016) 1158–1165. https://doi.org/10.1002/zaac.201600235.

108. K. Zou, J. Zhao, C. Liu, Z. Wang, & Z. Li, One-Pot Synthesis of Two Magnetic Coordination Polymers with Different Structures Due to the Connectivity of Sulfate Ions. *European Journal of Inorganic Chemistry*, **2013** (2013) 293–298. https://doi.org/10.1002/ejic.201200706.

109. C.-Y. Zhou, Y.-J. Du, F. Su, B.-X. Zhang, & S.-D. Li, Crystal structure of poly[bis(μ_2-1,4-di(1*H*-imidazol-1-yl)benzene-κ^2 *N* : *N′*)-(μ_2-tetraoxidomolybdato(VI)-κ^2 *O* : *O′*)cobalt(II)], $C_{24}H_{20}N$

$_8$ O $_4$ MoCo. *Zeitschrift für Kristallographie - New Crystal Structures*, **235** (2020) 1555–1557. https://doi.org/10.1515/ncrs-2020-0388.

110. L. J. S. Z. H. K. H. G. J. Wang Airong, Hydrothermal Synthesis, Crystal Structure and Surface Photo-electric Properties of a New Nickel（Ⅱ） 3-D Metalorganic Framework Constructed from the Rigid 1,4-Bis（imidazol-1-yl） benzene Ligand. *Science and Engineering B (Chemistry, Chemical Metallurgy, Environment and Mining)*, **36** (2017) 647–653.

111. G. Ye, K.-Y. Zou, Y. Yang, J.-J. Wang, X.-F. Gou, & Z.-X. Li, Anion effect on the topological frameworks of a series of manganese coordination polymers based on 1,4-bis(imidazol-1-yl)-benzene: Syntheses, crystal structures and magnetic properties. *Journal of Solid State Chemistry*, **225** (2015) 31–40. https://doi.org/10.1016/j.jssc.2014.11.015.

112. C.-Z. Li, X.-R. Huang, & Y.-H. Chen, Auxiliary ligand-directed structural variation of the Zn(**II**)–1,4-bis(imidazol-1-yl)benzene net: synthesis, characterization, and luminescence. *Journal of Coordination Chemistry*, **65** (2012) 3699–3707. https://doi.org/10.1080/00958972.2012.721880.

113. G. Bresciani, M. Guelfi, M. Dosa, V. Guiotto, V. Crocellà, M. Lessi, & M. Taddei, Synthesis and structural characterisation of Cu ^{II} -based MOFs constructed by combining functionalised 1,4-bis(1 *H* -imidazol-1-Yl)benzene ligands with copper sulfate. *CrystEngComm*, **27** (2025) 4071–4080. https://doi.org/10.1039/D5CE00247H.

114. B. Song, M. Shivanna, M. Gao, S. Wang, C. Deng, Q. Yang, S. J. Nikkhah, M. Vandichel, S. Kitagawa, & M. J. Zaworotko, Shape-Memory Effect Enabled by Ligand Substitution and CO $_2$ Affinity in a Flexible SIFSIX Coordination Network. *Angewandte Chemie International Edition*, **62** (2023). https://doi.org/10.1002/anie.202309985.

115. X.-F. Wang, C.-C. Du, S.-B. Zhou, & D.-Z. Wang, Six complexes based on bis(imidazole/benzimidazole-1-yl)pyridazine ligands: Syntheses, structures and properties. *Journal of Molecular Structure*, **1128** (2017) 103–110. https://doi.org/10.1016/j.molstruc.2016.08.051.

116. N. Li, Y. Hou, & G.-G. Hou, Synthesis, structure, and luminescence of Cd-MOF based on 5-(4-(1-Imidazolyl)phenyl)pyrimidine and cadmium silicofluoride. *Inorganic and Nano-Metal Chemistry*, **47** (2017) 1141–1144. https://doi.org/10.1080/24701556.2017.1284095.

117. J. Tang, Y. Shen, X. He, M. Chen, H. Zhao, Y. Wang, J. Jiang, P. Liu, R. Dang, M. Zhang, G. Qin, J. Bai, & J. Duan, Tuning Multiple Counter-Anions in Porous Coordination Polymers with **lcy** Topology for Acetylene/Ethylene Separation. *Inorganic Chemistry*, **63** (2024) 3667–3674. https://doi.org/10.1021/acs.inorgchem.3c03182.

118. X. Li, X. Wang, J. Zhou, L. Han, C. Sun, Q. Wang, & Z. Su, Ternary hybrids as efficient bifunctional electrocatalysts derived from bimetallic metal–organic-frameworks for overall water splitting. *Journal of Materials Chemistry A*, **6** (2018) 5789–5796. https://doi.org/10.1039/C7TA10558D.

119. L. Carlucci, G. Ciani, S. Maggini, & D. M. Proserpio, Metal–organic coordination frameworks assembled with the long flexible ligand 4,4′-bis(imidazol-1-ylmethyl)biphenyl. *CrystEngComm*, **10** (2008) 1191. https://doi.org/10.1039/b803815e.

120. L. Carlucci, G. Ciani, D. M. Proserpio, & L. Spadacini, Supramolecular isomers in the same crystal: a new case involving two different types of layers polycatenated in the 3D architecture of [Cu(bix) $_2$ (SO_4)]·7.5H_2O [bix = 1,4-bis(imidazol-1-ylmethyl)benzene]. *CrystEngComm*, **6** (2004) 96–101. https://doi.org/10.1039/B402935F.
121. X.-Y. Wang, H. Lu, K.-L. Huang, C.-P. Zhang, F. Tian, M.-Y. He, S.-C. Chen, & Q. Chen, Synthesis, characterization, ion-exchange, and catalytic properties of three isostructural copper(II) coordination polymers with a flexible bis(triazole) ligand. *Journal of Solid State Chemistry*, **312** (2022) 123201. https://doi.org/10.1016/j.jssc.2022.123201.
122. K. Liu, B. Ma, X. Guo, D. Ma, L. Meng, G. Zeng, F. Yang, G. Li, Z. Shi, & S. Feng, Syntheses, structures, luminescence and magnetic properties of eleven coordination polymers constructed by a N,N′-sulfuryldiimidazole ligand. *CrystEngComm*, **17** (2015) 5054–5065. https://doi.org/10.1039/C5CE00807G.
123. N. Singh & G. Anantharaman, Coordination polymers built with transition metal sulphates and angular 2,5-bis(imidazol-1-yl)thiophene (thim $_2$): synthesis, structure and photoluminescent properties. *CrystEngComm*, **16** (2014) 6203–6212. https://doi.org/10.1039/C4CE00691G.
124. H. Wang, Y. Duan, Y. Wang, Y. Huang, K. Ge, S. Wang, B. Zheng, Z. Wang, J. Bai, & J. Duan, Anion Regulates **scu** Topological Porous Coordination Polymers into the Acetylene Trap. *ACS Applied Materials & Interfaces*, **14** (2022) 13550–13559. https://doi.org/10.1021/acsami.2c01940.
125. Y. Duan, Y. Huang, C. Wang, Q. Wang, K. Ge, Z. Lu, H. Wang, J. Duan, J. Bai, & W. Jin, Formation and fine-tuning of metal–organic frameworks with carboxylic pincers for the recognition of a C_2H_2 tetramer and highly selective separation of C_2H_2/C_2H_4. *Chemical Science*, **14** (2023) 4605–4611. https://doi.org/10.1039/D3SC00877K.
126. Y. Li, Y. Wu, J. Zhao, J. Duan, & W. Jin, Systemic regulation of binding sites in porous coordination polymers for ethylene purification from ternary C2 hydrocarbons. *Chemical Science*, **15** (2024) 9318–9324. https://doi.org/10.1039/D4SC02659D.
127. S. Miao, Z. Li, C. Xu, D. Deng, & B. Ji, A Three-Dimensional Cadmium(II) Coordination Network Based on 1,3-Di-(1,2,4-triazole-4-yl)benzene: Synthesis, Structure, and Luminescence Properties. *Crystals*, **9** (2019) 592. https://doi.org/10.3390/cryst9110592.
128. S. Xiong, Y. He, R. Krishna, B. Chen, & Z. Wang, Metal–Organic Framework with Functional Amide Groups for Highly Selective Gas Separation. *Crystal Growth & Design*, **13** (2013) 2670–2674. https://doi.org/10.1021/cg4004438.
129. X. Zhu, T. Ke, J. Zhou, Y. Song, Q. Xu, Z. Zhang, Z. Bao, Y. Yang, Q. Ren, & Q. Yang, Vertex Strategy in Layered 2D MOFs: Simultaneous Improvement of Thermodynamics and Kinetics for Record C_2H_2/CO_2 Separation Performance. *Journal of the American Chemical Society*, **145** (2023) 9254–9263. https://doi.org/10.1021/jacs.3c01784.
130. J. You, H. Wang, T. Xiao, X. Wu, L. Zhang, & C.-Z. Lu, Introducing high concentration of hexafluorosilicate anions into an ultra-microporous MOF for highly efficient C2H2/CO2 and C2H2/C2H4 separation. *Chemical Engineering Journal*, **477** (2023) 147001. https://doi.org/10.1016/j.cej.2023.147001.

131. M. C. Laskoski, R. L. LaDuca Jr., R. S. Rarig Jr., & J. Zubieta, Oxoanion influences on the self-assembly of cationic co-ordination complex polymers of the nickel(II)–di-4-pyridylamine family. *Journal of the Chemical Society, Dalton Transactions*, (1999) 3467–3472. https://doi.org/10.1039/a903880i.

132. Lingyao Wang, CCDC 2411802: Experimental Crystal Structure Determination. *CSD Communication*, (2024).

133. P. Halasyamani, K. R. Heier, M. J. Willis, C. L. Stern, & K. R. Poeppelmeier, Syntheses and Structures of Two New Cu/Nb/pyrazine Complexes: Three dimensional CuNb(pyz) $_2$ OF $_5$ · (pyz)(H $_2$ O) and two dimensional [Cu(pyz) $_{2.5}$] $^+$ [NbF $_6$] $^-$ · (pyz). *Zeitschrift für anorganische und allgemeine Chemie*, **622** (1996) 479–485. https://doi.org/10.1002/zaac.19966220316.

134. K. Uemura, A. Maeda, T. K. Maji, P. Kanoo, & H. Kita, Syntheses, Crystal Structures and Adsorption Properties of Ultramicroporous Coordination Polymers Constructed from Hexafluorosilicate Ions and Pyrazine. *European Journal of Inorganic Chemistry*, **2009** (2009) 2329–2337. https://doi.org/10.1002/ejic.200900144.

135. H. Lin & P. A. Maggard, Microporosity, Optical Bandgap Sizes, and Photocatalytic Activity of M(I)-Nb(V) (M = Cu, Ag) Oxyfluoride Hybrids. *Crystal Growth & Design*, **10** (2010) 1323–1331. https://doi.org/10.1021/cg9013625.

136. R. Gautier, M. D. Donakowski, & K. R. Poeppelmeier, Orientational order of [VOF5]2− and [NbOF5]2− polar units in chains. *Journal of Solid State Chemistry*, **195** (2012) 132–139. https://doi.org/10.1016/j.jssc.2012.01.033.

137. K. A. Forrest, T. Pham, A. Hogan, K. McLaughlin, B. Tudor, P. Nugent, S. D. Burd, A. Mullen, C. R. Cioce, L. Wojtas, M. J. Zaworotko, & B. Space, Computational Studies of CO $_2$ Sorption and Separation in an Ultramicroporous Metal–Organic Material. *The Journal of Physical Chemistry C*, **117** (2013) 17687–17698. https://doi.org/10.1021/jp405781c.

138. O. Shekhah, Y. Belmabkhout, Z. Chen, V. Guillerm, A. Cairns, K. Adil, & M. Eddaoudi, Made-to-order metal-organic frameworks for trace carbon dioxide removal and air capture. *Nature Communications*, **5** (2014) 4228. https://doi.org/10.1038/ncomms5228.

139. O. Shekhah, Y. Belmabkhout, K. Adil, P. M. Bhatt, A. J. Cairns, & M. Eddaoudi, A facile solvent-free synthesis route for the assembly of a highly CO $_2$ selective and H $_2$ S tolerant NiSIFSIX metal–organic framework. *Chemical Communications*, **51** (2015) 13595–13598. https://doi.org/10.1039/C5CC04487A.

140. A. Cadiau, K. Adil, P. M. Bhatt, Y. Belmabkhout, & M. Eddaoudi, A metal-organic framework–based splitter for separating propylene from propane. *Science*, **353** (2016) 137–140. https://doi.org/10.1126/science.aaf6323.

141. P. M. Bhatt, Y. Belmabkhout, A. Cadiau, K. Adil, O. Shekhah, A. Shkurenko, L. J. Barbour, & M. Eddaoudi, A Fine-Tuned Fluorinated MOF Addresses the Needs for Trace CO $_2$ Removal and Air Capture Using Physisorption. *Journal of the American Chemical Society*, **138** (2016) 9301–9307. https://doi.org/10.1021/jacs.6b05345.

142. S. K. Elsaidi, M. H. Mohamed, C. M. Simon, E. Braun, T. Pham, K. A. Forrest, W. Xu, D. Banerjee, B. Space, M. J. Zaworotko, & P. K. Thallapally, Effect of ring rotation upon gas adsorption in

SIFSIX-3-M (M = Fe, Ni) pillared square grid networks. *Chemical Science*, **8** (2017) 2373–2380. https://doi.org/10.1039/C6SC05012C.

143. A. Kumar, C. Hua, D. G. Madden, D. O'Nolan, K.-J. Chen, L.-A. J. Keane, J. J. Perry, & M. J. Zaworotko, Hybrid ultramicroporous materials (HUMs) with enhanced stability and trace carbon capture performance. *Chemical Communications*, **53** (2017) 5946–5949. https://doi.org/10.1039/C7CC02289A.

144. L. Yang, X. Cui, Q. Yang, S. Qian, H. Wu, Z. Bao, Z. Zhang, Q. Ren, W. Zhou, B. Chen, & H. Xing, A Single-Molecule Propyne Trap: Highly Efficient Removal of Propyne from Propylene with Anion-Pillared Ultramicroporous Materials. *Advanced Materials*, **30** (2018). https://doi.org/10.1002/adma.201705374.

145. Y. Belmabkhout, P. M. Bhatt, K. Adil, R. S. Pillai, A. Cadiau, A. Shkurenko, G. Maurin, G. Liu, W. J. Koros, & M. Eddaoudi, Natural gas upgrading using a fluorinated MOF with tuned H2S and CO2 adsorption selectivity. *Nature Energy*, **3** (2018) 1059–1066. https://doi.org/10.1038/s41560-018-0267-0.

146. M. R. Tchalala, P. M. Bhatt, K. N. Chappanda, S. R. Tavares, K. Adil, Y. Belmabkhout, A. Shkurenko, A. Cadiau, N. Heymans, G. De Weireld, G. Maurin, K. N. Salama, & M. Eddaoudi, Fluorinated MOF platform for selective removal and sensing of SO2 from flue gas and air. *Nature Communications*, **10** (2019) 1328. https://doi.org/10.1038/s41467-019-09157-2.

147. Z. Zhang, Q. Ding, S. B. Peh, D. Zhao, J. Cui, X. Cui, & H. Xing, Mechano-assisted synthesis of an ultramicroporous metal–organic framework for trace CO_2 capture. *Chemical Communications*, **56** (2020) 7726–7729. https://doi.org/10.1039/D0CC03196H.

148. D. Antypov, A. Shkurenko, P. M. Bhatt, Y. Belmabkhout, K. Adil, A. Cadiau, M. Suyetin, M. Eddaoudi, M. J. Rosseinsky, & M. S. Dyer, Differential guest location by host dynamics enhances propylene/propane separation in a metal-organic framework. *Nature Communications*, **11** (2020) 6099. https://doi.org/10.1038/s41467-020-19207-9.

149. Z. Zhang, Q. Ding, J. Cui, X. Cui, & H. Xing, Fine-Tuning Pore Dimension in Hybrid Ultramicroporous Materials Boosting Simultaneous Trapping of Trace Alkynes from Alkenes. *Small*, **16** (2020). https://doi.org/10.1002/smll.202005360.

150. Z. Zhang, Q. Ding, S. B. Peh, D. Zhao, J. Cui, X. Cui, & H. Xing, Mechano-assisted synthesis of an ultramicroporous metal–organic framework for trace CO_2 capture. *Chemical Communications*, **56** (2020) 7726–7729. https://doi.org/10.1039/D0CC03196H.

151. M. L. Barsoum, J. Hofmann, H. Xie, Z. Chen, S. M. Vornholt, R. dos Reis, N. Burns, S. Kycia, K. W. Chapman, V. P. Dravid, & O. K. Farha, Probing Structural Transformations and Degradation Mechanisms by Direct Observation in SIFSIX-3-Ni for Direct Air Capture. *Journal of the American Chemical Society*, **146** (2024) 6557–6565. https://doi.org/10.1021/jacs.3c11503.

152. B. Gao, Z. Zhang, J. Hu, J. Cui, L. Chen, X. Cui, & H. Xing, Efficient separation of C4 olefins using tantalum pentafluor oxide anion-pillared hybrid microporous material. *Chinese Journal of Chemical Engineering*, **42** (2022) 49–54. https://doi.org/10.1016/j.cjche.2021.09.001.

153. A. Cadiau, Y. Belmabkhout, K. Adil, P. M. Bhatt, R. S. Pillai, A. Shkurenko, C. Martineau-Corcos, G. Maurin, & M. Eddaoudi, Hydrolytically stable fluorinated metal-organic frameworks for energy-efficient dehydration. *Science*, **356** (2017) 731–735. https://doi.org/10.1126/science.aam8310.

154. Y. Belmabkhout, P. M. Bhatt, K. Adil, R. S. Pillai, A. Cadiau, A. Shkurenko, G. Maurin, G. Liu, W. J. Koros, & M. Eddaoudi, Natural gas upgrading using a fluorinated MOF with tuned H2S and CO2 adsorption selectivity. *Nature Energy*, **3** (2018) 1059–1066. https://doi.org/10.1038/s41560-018-0267-0.

155. J. L. Manson, J. A. Schlueter, K. E. Garrett, P. A. Goddard, T. Lancaster, J. S. Möller, S. J. Blundell, A. J. Steele, I. Franke, F. L. Pratt, J. Singleton, J. Bendix, S. H. Lapidus, M. Uhlarz, O. Ayala-Valenzuela, R. D. McDonald, M. Gurak, & C. Baines, Bimetallic MOFs $(H_3O)_x[Cu(MF_6)(pyrazine)_2]\cdot(4-x)H_2O$ ($M = V^{4+}$, $x = 0$; $M = Ga^{3+}$, $x = 1$): co-existence of ordered and disordered quantum spins in the V^{4+} system. *Chemical Communications*, **52** (2016) 12653–12656. https://doi.org/10.1039/C6CC05873F.

156. S. Mukherjee, N. Kumar, A. A. Bezrukov, K. Tan, T. Pham, K. A. Forrest, K. A. Oyekan, O. T. Qazvini, D. G. Madden, B. Space, & M. J. Zaworotko, Amino-Functionalised Hybrid Ultramicroporous Materials that Enable Single-Step Ethylene Purification from a Ternary Mixture. *Angewandte Chemie International Edition*, **60** (2021) 10902–10909. https://doi.org/10.1002/anie.202100240.

157. J.-J. Liu, Y.-J. Hong, Y.-F. Guan, M.-J. Lin, C.-C. Huang, & W.-X. Dai, Lone pair–π interaction-induced generation of non-interpenetrated and photochromic cuboid 3-D naphthalene diimide coordination networks. *Dalton Transactions*, **44** (2015) 653–658. https://doi.org/10.1039/C4DT03124E.

158. C. Li, Z. Wei, M. Pan, H. Deng, J. Jiang, & C. Su, Structural tuning of coordination polymers by 4-connecting metal node and secondary building process. *Chinese Chemical Letters*, **30** (2019) 1297–1301. https://doi.org/10.1016/j.cclet.2019.02.001.

159. D.-Q. Qi, G.-G. Hou, X.-J. Wang, Y.-P. Zhang, & J.-Z. You, A 6^3-Nets Framework Constructed by Cadmium(II) Hexafluorosilicate and 3-(Pyridin-4-yl)benzenamine: Synthesis, Structure, and Luminescence Property. *Synthesis and Reactivity in Inorganic, Metal-Organic, and Nano-Metal Chemistry*, **45** (2015) 407–410. https://doi.org/10.1080/15533174.2013.841206.

160. H. Li, C. Liu, C. Chen, Z. Di, D. Yuan, J. Pang, W. Wei, M. Wu, & M. Hong, An Unprecedented Pillar-Cage Fluorinated Hybrid Porous Framework with Highly Efficient Acetylene Storage and Separation. *Angewandte Chemie International Edition*, **60** (2021) 7547–7552. https://doi.org/10.1002/anie.202013988.

161. Y. Li, H. Li, S. Zou, Y. Liu, H. Li, Z. Ji, Z. Di, C. Chen, & M. Wu, Anion-Induced Structural Transformation of a Cage-Based Metal–Organic Framework. *Crystal Growth & Design*, **23** (2023) 2264–2271. https://doi.org/10.1021/acs.cgd.2c01315.

162. Y. Jiang, L. Wang, T. Yan, J. Hu, W. Sun, R. Krishna, D. Wang, Z. Gu, D. Liu, X. Cui, H. Xing, & Y. Zhang, Insights into the thermodynamic–kinetic synergistic separation of propyne/propylene in anion pillared cage MOFs with entropy–enthalpy balanced adsorption sites. *Chemical Science*, **14** (2023) 298–309. https://doi.org/10.1039/D2SC05742E.

163. Y. Chen, Y. Jiang, J. Li, X. Hong, H. Ni, L. Wang, N. Ma, M. Tong, R. Krishna, & Y. Zhang, Optimizing the cask effect in multicomponent natural gas purification to provide high methane productivity. *AIChE Journal*, **70** (2024). https://doi.org/10.1002/aic.18320.

164. W. Li, C. Cheng, G. Gao, H. Xu, W. Huang, Z. Qu, & N. Yan, Trace SO_2 capture within the engineered pore space using a highly stable SnF_6^{2-} -pillared MOF. *Materials Horizons*, **11** (2024) 1889–1898. https://doi.org/10.1039/D3MH02222F.

165. R. Chen, F. Zheng, J. Li, Y. Liu, F. Zhou, H. Sun, Q. Yang, Z. Zhang, Q. Ren, & Z. Bao, Aperture Fine-Tuning in Cage-Like Metal–Organic Frameworks via Molecular Valve Strategy for Efficient Hexane Isomer Separation. *Small Structures*, **5** (2024). https://doi.org/10.1002/sstr.202300302.

166. P. Zhou, A Porous Cadmium(II) Framework: Synthesis, Crystal Structure, Gas Adsorption, and Fluorescence Sensing Properties. *Zeitschrift für anorganische und allgemeine Chemie*, **643** (2017) 653–656. https://doi.org/10.1002/zaac.201600448.

167. M. Lusi, P. B. A. Fechine, K.-J. Chen, J. J. Perry, & M. J. Zaworotko, A rare cationic building block that generates a new type of polyhedral network with “cross-linked” **pto** topology. *Chemical Communications*, **52** (2016) 4160–4162. https://doi.org/10.1039/C5CC10203K.

168. S. Zhang, S. Zhang, N. Yin, Z. Huang, W. Xu, K. Yue, X. Li, & D. Li, Exploring Reversible Thermochromic Behavior in a Rare Ni(II)-MOF System. *ACS Applied Materials & Interfaces*, **13** (2021) 6430–6441. https://doi.org/10.1021/acsami.0c21116.

169. J.-J. Wang, Y.-Z. Yu, E.-N. Wang, F. Jin, M.-Y. Zhang, Q.-Q. Zhao, & H.-N. Huang, Inorganic-acid-induced structures of cobalt(II) and copper(II) complexes based on 2,4,5-tri(4-pyridyl)-imidazole ligand. *Inorganic and Nano-Metal Chemistry*, **47** (2017) 1590–1598. https://doi.org/10.1080/24701556.2017.1357614.

170. A. Dey, A. Garai, V. Gude, & K. Biradha, Thermochromic, Solvatochromic, and Piezochromic Cd(II) and Zn(II) Coordination Polymers: Detection of Small Molecules by Luminescence Switching from Blue to Green. *Crystal Growth & Design*, **18** (2018) 6070–6077. https://doi.org/10.1021/acs.cgd.8b00924.

171. X.-H. Lu & K.-L. Zhong, A new three-dimensional manganese(II) coordination polymer based on the 1,3,5-tris[(1 *H* -imidazol-1-yl)methyl]benzene ligand. *Acta Crystallographica Section C Structural Chemistry*, **72** (2016) 895–900. https://doi.org/10.1107/S2053229616015965.

172. L. L. Kai-Long Zhong, CCDC 1430131: Experimental Crystal Structure Determination. *Wuji Huaxue Xuebao*, **32** (2016) 275.

173. G. De Munno, T. Poerio, M. Julve, F. Lloret, J. Faus, & A. Caneschi, Syntheses, crystal structures and magnetic properties of one-, two- and three-dimensional 2,2′-bipyrimidine-containing copper(II) complexes. *Journal of the Chemical Society, Dalton Transactions*, (1998) 1679–1686. https://doi.org/10.1039/a708477c.

174. Y. H. Andaloussi, A. Subanbekova, K. Koupepidou, S. Javan Nikkhah, A. A. Bezrukov, A. Raza, H. Sakamoto, S. Kitagawa, M. Vandichel, S. Mukherjee, & M. J. Zaworotko, The Prototypal Diamondoid Topology Hybrid Ultramicroporous Materials and Their Water Vapor Sorption Properties. *Journal of the American Chemical Society*, (2025). https://doi.org/10.1021/jacs.5c14477.

175. G.-C. Xu, Q. Hua, T. Okamura, Z.-S. Bai, Y.-J. Ding, Y.-Q. Huang, G.-X. Liu, W.-Y. Sun, & N. Ueyama, Cadmium(<scp>ii</scp>) coordination polymers with flexible tetradentate ligand 1,2,4,5-tetrakis(imidazol-1-ylmethyl)benzene: anion effect and reversible anion exchange property. *CrystEngComm*, **11** (2009) 261–270. https://doi.org/10.1039/B813220H.

176. H.-Y. Bai, J.-F. Ma, J. Yang, L.-P. Zhang, J.-C. Ma, & Y.-Y. Liu, Eight Two-Dimensional and Three-Dimensional Metal−Organic Frameworks Based on a Flexible Tetrakis(imidazole) Ligand: Synthesis, Topological Structures, and Photoluminescent Properties. *Crystal Growth & Design*, **10** (2010) 1946–1959. https://doi.org/10.1021/cg100032n.

177. Yi.-Y. Liu & et al., Crystal structure of tetrakis-(imidazol-1-ylmethyl)methane cobalt(**II**) tungstate-monohydrate, [CoWO4(C17H20N8)](H2O), C17H22CoN8O5W. *Zeitschrift für Kristallographie - New Crystal Structures*, **227** (2012) 459–460. https://doi.org/10.1524/ncrs.2012.0208.

178. X.-W. Gu, E. Wu, J.-X. Wang, H.-M. Wen, B. Chen, B. Li, & G. Qian, Programmed fluorine binding engineering in anion-pillared metal-organic framework for record trace acetylene capture from ethylene. *Science Advances*, **9** (2023). https://doi.org/10.1126/sciadv.adh0135.

179. J. Yang, L. Lin, S. Zou, Z. Ji, C. Chen, & M. Wu, Robust Anion-Pillared Ultramicroporous Material for C_2H_2/C_2H_4 Separation with High C_2H_2 Uptake and Selectivity. *Inorganic Chemistry*, **64** (2025) 6786–6792. https://doi.org/10.1021/acs.inorgchem.5c00749.

180. R.-D. Wang, W.-Q. Zhang, H.-B. Lv, Y.-T. Chen, L. Wang, S.-H. Zhou, L. Du, & Q.-H. Zhao, Sulfate-functionalized Fe-based MOF for removal of Pb(Ⅱ) and NO3- in industrial wastewater. *Journal of Environmental Chemical Engineering*, **12** (2024) 112167. https://doi.org/10.1016/j.jece.2024.112167.

181. Y.-Y. Guo, R.-D. Wang, W.-M. Wei, F. Fang, X.-H. Zhao, S.-S. Zhang, T.-Z. Shen, J. Zhang, Q.-H. Zhao, & J. Wang, Structure and properties of metal–organic frameworks modulated by sulfate ions. *Dalton Transactions*, **52** (2023) 15940–15949. https://doi.org/10.1039/D3DT01995K.

182. S.-H. Zhou, R.-D. Wang, Y. Yang, A. Xiao, X. Pan, & F. Jiang, Synergistic adsorption-catalysis system based on the advanced oxidation technology of single-atom MOFs: Selective degradation and contribution to different ionization potential pollutants. *Chemical Engineering Journal*, **513** (2025) 163031. https://doi.org/10.1016/j.cej.2025.163031.

183. D. Sensharma, D. J. O'Hearn, A. Koochaki, A. A. Bezrukov, N. Kumar, B. H. Wilson, M. Vandichel, & M. J. Zaworotko, The First Sulfate-Pillared Hybrid Ultramicroporous Material, SOFOUR-1-Zn, and Its Acetylene Capture Properties. *Angewandte Chemie International Edition*, **61** (2022). https://doi.org/10.1002/anie.202116145.

184. S. Zou, Z. Di, Y. Liu, Z. Ji, H. Li, C. Chen, & M. Wu, An anion-functionalized zinc-organic framework for efficient C2H2/C2H4 separation. *Inorganic Chemistry Communications*, **137** (2022) 109198. https://doi.org/10.1016/j.inoche.2022.109198.

185. D. Sensharma, B. H. Wilson, N. Kumar, D. J. O'Hearn, & M. J. Zaworotko, Pillar Modularity in **fsc** Topology Hybrid Ultramicroporous Materials Based upon Tetra(4-pyridyl)benzene. *Crystal Growth & Design*, **22** (2022) 5472–5480. https://doi.org/10.1021/acs.cgd.2c00561.

186. L. Wang, Y. Zhang, P. Zhang, X. Liu, H. Xiong, R. Krishna, J. Liu, H. Shuai, P. Wang, Z. Zhou, J. Chen, S. Chen, S. Deng, & J. Wang, Electro-field alignment in a novel metal–organic

framework for benchmark separation of ethylene from a ternary gas mixture. *AIChE Journal*, **70** (2024). https://doi.org/10.1002/aic.18396.

187. Y. Su, R. Chen, P. Zhang, X. He, X. Liu, Y. Liu, H. Xiong, Z. Zhao, J. Luo, J. Chen, S. Chen, Z. Zeng, Z. Bao, S. Deng, & J. Wang, Dual pore-size sieving in a novel oxygenate-pillared microporous adsorbent for <scp>C6</scp> alkane isomers separation. *AIChE Journal*, **69** (2023). https://doi.org/10.1002/aic.17937.

188. S.-H. Zhou, R.-D. Wang, Y. Yang, H. Li, L. Luo, & F. Jiang, Multifunctional Ni-MOF (3D) with N-rich structures for self-generated reactive oxygen species and enhanced electron transfer for synergistic degradation of organic pollutants and removal of Hg(II). *Journal of Cleaner Production*, **476** (2024) 143807. https://doi.org/10.1016/j.jclepro.2024.143807.

189. S.-H. Zhou, Y. Yang, R.-D. Wang, Y. Cui, S. Ji, L. Du, & F. Jiang, Iron-based nitrogen-rich metal-organic framework structure for activation of hydrogen peroxide and peroxymonosulfate for ultra-efficient tetracycline degradation. *Journal of Colloid and Interface Science*, **680** (2025) 307–325. https://doi.org/10.1016/j.jcis.2024.11.004.

190. Y. Zhang, W. Sun, B. Luan, J. Li, D. Luo, Y. Jiang, L. Wang, & B. Chen, Topological Design of Unprecedented Metal-Organic Frameworks Featuring Multiple Anion Functionalities and Hierarchical Porosity for Benchmark Acetylene Separation. *Angewandte Chemie International Edition*, **62** (2023). https://doi.org/10.1002/anie.202309925.

191. Y. Zhang, Y. Han, B. Luan, L. Wang, W. Yang, Y. Jiang, T. Ben, Y. He, & B. Chen, Metal–Organic Framework with Space-Partition Pores by Fluorinated Anions for Benchmark C_2H_2/CO_2 Separation. *Journal of the American Chemical Society*, **146** (2024) 17220–17229. https://doi.org/10.1021/jacs.4c03442.

192. Q. Lin, C. Mao, A. Kong, X. Bu, X. Zhao, & P. Feng, Porphyrinic coordination lattices with fluoropillars. *J. Mater. Chem. A*, **5** (2017) 21189–21195. https://doi.org/10.1039/C7TA06658A.

193. Z. Liang, G. Zhou, H. Tan, Y. Mou, J. Zhang, H. Guo, S. Yang, H. Lei, H. Zheng, W. Zhang, H. Lin, & R. Cao, Constructing $Co_4(SO_4)_4$ Clusters within Metal–Organic Frameworks for Efficient Oxygen Electrocatalysis. *Advanced Materials*, **36** (2024). https://doi.org/10.1002/adma.202408094.

194. S. K. Elsaidi, M. H. Mohamed, T. Pham, T. Hussein, L. Wojtas, M. J. Zaworotko, & B. Space, Crystal Engineering of a 4,6-c fsc Platform That Can Serve as a Carbon Dioxide Single-Molecule Trap. *Crystal Growth & Design*, **16** (2016) 1071–1080. https://doi.org/10.1021/acs.cgd.5b01632.

195. A. M. Tous-Granados & A. J. Hernandez-Maldonado, A SIFSIX-MOF constructed from a metalloligand yields enhanced stability for selective CO_2 adsorption. *Chemical Communications*, **59** (2023) 10020–10023. https://doi.org/10.1039/D3CC02683C.

196. X.-Y. Tian, H.-L. Zhou, X. Fang, Z.-W. Mo, Y.-T. Xu, D.-D. Zhou, & J.-P. Zhang, Diverse coordination polymers from a new bent dipyridyl-type ligand 3,6-di(pyridin-4-yl)-9H-carbazole. *CrystEngComm*, **19** (2017) 6164–6169. https://doi.org/10.1039/C7CE01483J.

197. N. Kumar, S. Mukherjee, N. C. Harvey-Reid, A. A. Bezrukov, K. Tan, V. Martins, M. Vandichel, T. Pham, L. M. van Wyk, K. Oyekan, A. Kumar, K. A. Forrest, K. M. Patil, L. J. Barbour, B. Space, Y. Huang, P. E. Kruger, & M. J. Zaworotko, Breaking the trade-off between selectivity and

adsorption capacity for gas separation. *Chem*, **7** (2021) 3085–3098. https://doi.org/10.1016/j.chempr.2021.07.007.

198. N. C. Harvey-Reid, H. S. Scott, K. M. Patil, N. Kumar, C. Healy, M. J. Zaworotko, S. Mukherjee, & P. E. Kruger, Fine-tuning of gas uptake and selectivity in a hexafluorozirconate pillared coordination network that features two porous phases. *CrystEngComm*, **27** (2025) 1736–1741. https://doi.org/10.1039/D4CE01250J.
199. H. S. Scott, S. Mukherjee, D. R. Turner, M. I. J. Polson, M. J. Zaworotko, & P. E. Kruger, Crystal engineering of dichromate pillared hybrid ultramicroporous materials incorporating pyrazole-based ligands. *CrystEngComm*, **20** (2018) 1193–1197. https://doi.org/10.1039/C8CE00149A.
200. V. V. Ponomareva, K. V. Domasevich, V. V. Komarchuk, J. Sieler, H. Krautscheid, & V. V. Skopenko, Formation of five-connected three-dimensional coordination polymers through the bridging function of the anions. *Russian Journal of Inorganic Chemistry*, **51** (2006) 1355–1362. https://doi.org/10.1134/S003602360609004X.
201. V. V. Ponomarova, V. V. Komarchuk, I. Boldog, H. Krautscheid, & K. V. Domasevitch, Modular construction of 3D coordination frameworks incorporating SiF62− links: Accessing the significance of [M(pyrazole)4{SiF6}] synthon. *CrystEngComm*, **15** (2013) 8280. https://doi.org/10.1039/c3ce41238e.
202. V. V. Ponomarova, V. V. Komarchuk, I. Boldog, A. N. Chernega, J. Sieler, & K. V. Domasevitch, Mixed-anion complexes with a bipyrazolyl ligand. A new entry to a realm of three-dimensional five-connected coordination topologiesElectronic supplementary information (ESI) available: crystal structure determination and refinement details for 1â€“4. See http://www.rsc.org/suppdata/cc/b1/b110599j/. *Chemical Communications*, (2002) 436–437. https://doi.org/10.1039/b110599j.
203. W.-J. Shi, L.-Y. Du, H.-Y. Yang, K. Zhang, L. Hou, & Y.-Y. Wang, Ligand Configuration-Induced Manganese(II) Coordination Polymers: Syntheses, Crystal Structures, Sorption, and Magnetic Properties. *Inorganic Chemistry*, **56** (2017) 10090–10098. https://doi.org/10.1021/acs.inorgchem.7b01657.
204. L.-Y. Du, H. Wang, G. Liu, D. Xie, F.-S. Guo, L. Hou, & Y.-Y. Wang, Structural diversity of five new bitriazole-based complexes: luminescence, sorption, and magnetic properties. *Dalton Transactions*, **44** (2015) 1110–1119. https://doi.org/10.1039/C4DT03129F.
205. L. Croitor, E. B. Coropceanu, A. V. Siminel, V. Ch. Kravtsov, & M. S. Fonari, Polymeric Zn(II) and Cd(II) Sulfates with Bipyridine and Dioxime Ligands: Supramolecular Isomerism, Chirality, and Luminescence. *Crystal Growth & Design*, **11** (2011) 3536–3544. https://doi.org/10.1021/cg200465f.
206. Y. Wen, T. Sheng, Z. Sun, Z. Xue, Y. Wang, Y. Wang, S. Hu, X. Ma, & X. Wu, A combination of the “pillaring” strategy and chiral induction: an approach to prepare homochiral three-dimensional coordination polymers from achiral precursors. *Chemical Communications*, **50** (2014) 8320. https://doi.org/10.1039/c4cc03478c.
207. D. J. O’Hearn, D. Sensharma, A. Raza, A. A. Bezrukov, M. Vandichel, S. Mukherjee, & M. J. Zaworotko, Crystal engineering of a new platform of hybrid ultramicroporous materials and their

C_2H_2/CO_2 separation properties. *Chemical Science*, **15** (2024) 17937–17943. https://doi.org/10.1039/D4SC03029J.

208. L.-N. Zhu, Z.-P. Deng, L.-H. Huo, & S. Gao, A series of helical coordination polymers based on two racemic bis(pyridylmethylene) propane-1,2-diamine ligands: relationship of conformations, structures and properties. *CrystEngComm*, **21** (2019) 7249–7259. https://doi.org/10.1039/C9CE01210A.
209. Bruker AXS Inc., 2021, APEX4 v2021.4-0. (n.d.).
210. L. Krause, R. Herbst-Irmer, G. M. Sheldrick, & D. Stalke, Comparison of silver and molybdenum microfocus X-ray sources for single-crystal structure determination. *Journal of Applied Crystallography*, **48** (2015) 3–10. https://doi.org/10.1107/S1600576714022985.
211. G. M. Sheldrick, Crystal structure refinement with *SHELXL*. *Acta Crystallographica Section C Structural Chemistry*, **71** (2015) 3–8. https://doi.org/10.1107/S2053229614024218.
212. K. S. Walton & R. Q. Snurr, Applicability of the BET Method for Determining Surface Areas of Microporous Metal−Organic Frameworks. *Journal of the American Chemical Society*, **129** (2007) 8552–8556. https://doi.org/10.1021/ja071174k.
213. A. J. Howarth, A. W. Peters, N. A. Vermeulen, T. C. Wang, J. T. Hupp, & O. K. Farha, Best Practices for the Synthesis, Activation, and Characterization of Metal–Organic Frameworks. *Chemistry of Materials*, **29** (2017) 26–39. https://doi.org/10.1021/acs.chemmater.6b02626.
214. Sangwon Lee, IAST++ software for Ideal Adsorbed Solution Theory Calculations. (2017). https://sangwon91.github.io/IASTpp// (accessed September 9, 2025).
215. X. Liu, P. Zhang, H. Xiong, Y. Zhang, K. Wu, J. Liu, R. Krishna, J. Chen, S. Chen, Z. Zeng, S. Deng, & J. Wang, Engineering Pore Environments of Sulfate-Pillared Metal-Organic Framework for Efficient C_2H_2/CO_2 Separation with Record Selectivity. *Advanced Materials*, **35** (2023). https://doi.org/10.1002/adma.202210415.
216. J. Wang, Y. Zhang, Y. Su, X. Liu, P. Zhang, R.-B. Lin, S. Chen, Q. Deng, Z. Zeng, S. Deng, & B. Chen, Fine pore engineering in a series of isoreticular metal-organic frameworks for efficient C2H2/CO2 separation. *Nature Communications*, **13** (2022) 200. https://doi.org/10.1038/s41467-021-27929-7.
217. R.-B. Lin, L. Li, H. Wu, H. Arman, B. Li, R.-G. Lin, W. Zhou, & B. Chen, Optimized Separation of Acetylene from Carbon Dioxide and Ethylene in a Microporous Material. *Journal of the American Chemical Society*, **139** (2017) 8022–8028. https://doi.org/10.1021/jacs.7b03850.
218. S. Shang, P. Wang, H. Xiong, X. Liu, J. Liu, H. Shuai, L. Wang, Z. Zhu, Z. Zhao, Y. Peng, J. Chen, S. Chen, Z. Zhou, & J. Wang, Efficient molecular sieving separation of C2H2/CO2 by a flexible interpenetrated metal-organic framework. *Separation and Purification Technology*, **354** (2025) 129468. https://doi.org/10.1016/j.seppur.2024.129468.
219. D. Zhou, B. Meng, Z. Qiu, X. Liu, J. Cui, P. Zhang, R. You, T. Wu, X. Suo, X. Lu, Y. Zhou, J. Wang, X. Cui, L. Yang, & H. Xing, Linear Anion Chain-Assembled Nonporous Swelling Molecular Sieve for Benchmark C2–C4 Hydrocarbon Separations. *Journal of the American Chemical Society*, **147** (2025) 21725–21734. https://doi.org/10.1021/jacs.5c03723.

220. L. Zhang, K. Jiang, L. Yang, L. Li, E. Hu, L. Yang, K. Shao, H. Xing, Y. Cui, Y. Yang, B. Li, B. Chen, & G. Qian, Benchmark C_2H_2/CO_2 Separation in an Ultra-Microporous Metal–Organic Framework via Copper(I)-Alkynyl Chemistry. *Angewandte Chemie International Edition*, **60** (2021) 15995–16002. https://doi.org/10.1002/anie.202102810.

221. L. Wang, W. Sun, Y. Zhang, N. Xu, R. Krishna, J. Hu, Y. Jiang, Y. He, & H. Xing, Interpenetration Symmetry Control Within Ultramicroporous Robust Boron Cluster Hybrid MOFs for Benchmark Purification of Acetylene from Carbon Dioxide. *Angewandte Chemie International Edition*, **60** (2021) 22865–22870. https://doi.org/10.1002/anie.202107963.

222. Z. Niu, X. Cui, T. Pham, G. Verma, P. C. Lan, C. Shan, H. Xing, K. A. Forrest, S. Suepaul, B. Space, A. Nafady, A. M. Al-Enizi, & S. Ma, A MOF-based Ultra-Strong Acetylene Nano-trap for Highly Efficient C_2H_2/CO_2 Separation. *Angewandte Chemie International Edition*, **60** (2021) 5283–5288. https://doi.org/10.1002/anie.202016225.

223. S. Shang, Z. Zhou, H. Wang, Y. Wang, X. Liu, Z. Zhu, Y. Zeng, C. Liu, H. Xiong, H. Liu, F. Zhao, J. Chen, S. Chen, Z. Zhou, & J. Wang, A Rigid, Stable, and Scalable Aliphatic MOF Adsorbent for Efficient C_2H_2/CO_2 Separation with Record Acetylene Packing Density. *Angewandte Chemie International Edition*, **64** (2025). https://doi.org/10.1002/anie.202503317.

224. J. Pei, K. Shao, J. Wang, H. Wen, Y. Yang, Y. Cui, R. Krishna, B. Li, & G. Qian, A Chemically Stable Hofmann-Type Metal−Organic Framework with Sandwich-Like Binding Sites for Benchmark Acetylene Capture. *Advanced Materials*, **32** (2020). https://doi.org/10.1002/adma.201908275.

225. Y. Chen, Y. Du, Y. Wang, R. Krishna, L. Li, J. Yang, J. Li, & B. Mu, A stable metal–organic framework with well-matched pore cavity for efficient acetylene separation. *AIChE Journal*, **67** (2021). https://doi.org/10.1002/aic.17152.

226. N. Kumar, S. Mukherjee, A. A. Bezrukov, M. Vandichel, M. Shivanna, D. Sensharma, A. Bajpai, V. Gascón, K. Otake, S. Kitagawa, & M. J. Zaworotko, A square lattice topology coordination network that exhibits highly selective C_2H_2/CO_2 separation performance. *SmartMat*, **1** (2020). https://doi.org/10.1002/smm2.1008.

227. Y. Peng, T. Pham, P. Li, T. Wang, Y. Chen, K. Chen, K. A. Forrest, B. Space, P. Cheng, M. J. Zaworotko, & Z. Zhang, Robust Ultramicroporous Metal–Organic Frameworks with Benchmark Affinity for Acetylene. *Angewandte Chemie International Edition*, **57** (2018) 10971–10975. https://doi.org/10.1002/anie.201806732.

228. J. Gao, X. Qian, R. Lin, R. Krishna, H. Wu, W. Zhou, & B. Chen, Mixed Metal–Organic Framework with Multiple Binding Sites for Efficient C 2 H 2 /CO 2 Separation . *Angewandte Chemie*, **132** (2020) 4426–4430. https://doi.org/10.1002/ange.202000323.

229. L. Zhang, K. Jiang, J. Zhang, J. Pei, K. Shao, Y. Cui, Y. Yang, B. Li, B. Chen, & G. Qian, Low-Cost and High-Performance Microporous Metal–Organic Framework for Separation of Acetylene from Carbon Dioxide. *ACS Sustainable Chemistry & Engineering*, **7** (2019) 1667–1672. https://doi.org/10.1021/acssuschemeng.8b05431.

230. Q.-L. Qian, X.-W. Gu, J. Pei, H.-M. Wen, H. Wu, W. Zhou, B. Li, & G. Qian, A novel anion-pillared metal–organic framework for highly efficient separation of acetylene from ethylene and carbon

dioxide. *Journal of Materials Chemistry A*, **9** (2021) 9248–9255. https://doi.org/10.1039/D0TA11340A.

231. S. Mukherjee, Y. He, D. Franz, S. Wang, W. Xian, A. A. Bezrukov, B. Space, Z. Xu, J. He, & M. J. Zaworotko, Halogen–C_2H_2 Binding in Ultramicroporous Metal–Organic Frameworks (MOFs) for Benchmark C_2H_2/CO_2 Separation Selectivity. *Chemistry – A European Journal*, **26** (2020) 4923–4929. https://doi.org/10.1002/chem.202000008.

232. Y. Zhang, J. Hu, R. Krishna, L. Wang, L. Yang, X. Cui, S. Duttwyler, & H. Xing, Rational Design of Microporous MOFs with Anionic Boron Cluster Functionality and Cooperative Dihydrogen Binding Sites for Highly Selective Capture of Acetylene. *Angewandte Chemie International Edition*, **59** (2020) 17664–17669. https://doi.org/10.1002/anie.202007681.

233. H. S. Scott, M. Shivanna, A. Bajpai, D. G. Madden, K.-J. Chen, T. Pham, K. A. Forrest, A. Hogan, B. Space, J. J. Perry IV, & M. J. Zaworotko, Highly Selective Separation of C_2H_2 from CO_2 by a New Dichromate-Based Hybrid Ultramicroporous Material. *ACS Applied Materials & Interfaces*, **9** (2017) 33395–33400. https://doi.org/10.1021/acsami.6b15250.

234. J. Lee, C. Y. Chuah, J. Kim, Y. Kim, N. Ko, Y. Seo, K. Kim, T. H. Bae, & E. Lee, Separation of Acetylene from Carbon Dioxide and Ethylene by a Water-Stable Microporous Metal–Organic Framework with Aligned Imidazolium Groups inside the Channels. *Angewandte Chemie International Edition*, **57** (2018) 7869–7873. https://doi.org/10.1002/anie.201804442.

235. J.-W. Zhang, M.-C. Hu, S.-N. Li, Y.-C. Jiang, P. Qu, & Q.-G. Zhai, Assembly of $[Cu_2(COO)_4]$ and $[M_3(\mu_3\text{-}O)(COO)_6]$ (M = Sc, Fe, Ga, and In) building blocks into porous frameworks towards ultra-high C_2H_2/CO_2 and C_2H_2/CH_4 separation performance. *Chemical Communications*, **54** (2018) 2012–2015. https://doi.org/10.1039/C7CC09484A.

236. D.-M. Chen, C.-X. Sun, N.-N. Zhang, H.-H. Si, C.-S. Liu, & M. Du, Tunable Robust pacs-MOFs: a Platform for Systematic Enhancement of the C_2H_2 Uptake and C_2H_2/C_2H_4 Separation Performance. *Inorganic Chemistry*, **57** (2018) 2883–2889. https://doi.org/10.1021/acs.inorgchem.7b03278.

237. Y.-Z. Hao, K. Shao, X. Zhang, Y.-H. Yu, D. Liu, H.-M. Wen, Y. Cui, B. Li, B. Chen, & G. Qian, Pore Space Partition Enabled by Lithium(I) Chelation of a Metal–Organic Framework for Benchmark C_2H_2/CO_2 Separation. *Journal of the American Chemical Society*, **147** (2025) 11257–11266. https://doi.org/10.1021/jacs.4c18209.

238. W. Gong, H. Cui, Y. Xie, Y. Li, X. Tang, Y. Liu, Y. Cui, & B. Chen, Efficient C_2H_2/CO_2 Separation in Ultramicroporous Metal–Organic Frameworks with Record C_2H_2 Storage Density. *Journal of the American Chemical Society*, **143** (2021) 14869–14876. https://doi.org/10.1021/jacs.1c07191.

239. J. W. Yoon, J. S. Lee, S. Lee, K. H. Cho, Y. K. Hwang, M. Daturi, C. Jun, R. Krishna, & J. Chang, Adsorptive Separation of Acetylene from Light Hydrocarbons by Mesoporous Iron Trimesate MIL-100(Fe). *Chemistry – A European Journal*, **21** (2015) 18431–18438. https://doi.org/10.1002/chem.201502893.

240. H.-M. Wen, H. Wang, B. Li, Y. Cui, H. Wang, G. Qian, & B. Chen, A Microporous Metal–Organic Framework with Lewis Basic Nitrogen Sites for High C_2H_2 Storage and Significantly Enhanced

C_2H_2/CO_2 Separation at Ambient Conditions. *Inorganic Chemistry*, **55** (2016) 7214–7218. https://doi.org/10.1021/acs.inorgchem.6b00748.

241. D.-M. Chen, X.-H. Liu, J.-Y. Tian, J.-H. Zhang, C.-S. Liu, & M. Du, Microporous Cobalt(II)–Organic Framework with Open O-Donor Sites for Effective C_2H_2 Storage and C_2H_2/CO_2 Separation at Room Temperature. *Inorganic Chemistry*, **56** (2017) 14767–14770. https://doi.org/10.1021/acs.inorgchem.7b02764.
242. W. Wang, Y. Chen, P. Feng, & X. Bu, Tailorable Multi-Modular Pore-Space-Partitioned Vanadium Metal-Organic Frameworks for Gas Separation. *Advanced Materials*, **36** (2024). https://doi.org/10.1002/adma.202403834.
243. X. Zhang, R.-B. Lin, H. Wu, Y. Huang, Y. Ye, J. Duan, W. Zhou, J.-R. Li, & B. Chen, Maximizing acetylene packing density for highly efficient C2H2/CO2 separation through immobilization of amine sites within a prototype MOF. *Chemical Engineering Journal*, **431** (2022) 134184. https://doi.org/10.1016/j.cej.2021.134184.
244. Y. Zhang, W. Sun, B. Luan, J. Li, D. Luo, Y. Jiang, L. Wang, & B. Chen, Topological Design of Unprecedented Metal-Organic Frameworks Featuring Multiple Anion Functionalities and Hierarchical Porosity for Benchmark Acetylene Separation. *Angewandte Chemie International Edition*, **62** (2023). https://doi.org/10.1002/anie.202309925.
245. N. Kumar, S. Mukherjee, N. C. Harvey-Reid, A. A. Bezrukov, K. Tan, V. Martins, M. Vandichel, T. Pham, L. M. van Wyk, K. Oyekan, A. Kumar, K. A. Forrest, K. M. Patil, L. J. Barbour, B. Space, Y. Huang, P. E. Kruger, & M. J. Zaworotko, Breaking the trade-off between selectivity and adsorption capacity for gas separation. *Chem*, **7** (2021) 3085–3098. https://doi.org/10.1016/j.chempr.2021.07.007.
246. K.-J. Chen, H. S. Scott, D. G. Madden, T. Pham, A. Kumar, A. Bajpai, M. Lusi, K. A. Forrest, B. Space, J. J. Perry, & M. J. Zaworotko, Benchmark C2H2/CO2 and CO2/C2H2 Separation by Two Closely Related Hybrid Ultramicroporous Materials. *Chem*, **1** (2016) 753–765. https://doi.org/10.1016/j.chempr.2016.10.009.
247. H.-M. Wen, C. Liao, L. Li, L. Yang, J. Wang, L. Huang, B. Li, B. Chen, & J. Hu, Reversing C_2H_2–CO_2 adsorption selectivity in an ultramicroporous metal–organic framework platform. *Chemical Communications*, **55** (2019) 11354–11357. https://doi.org/10.1039/C9CC05997K.
248. H. Yang, Y. Chen, C. Dang, A. N. Hong, P. Feng, & X. Bu, Optimization of Pore-Space-Partitioned Metal–Organic Frameworks Using the Bioisosteric Concept. *Journal of the American Chemical Society*, **144** (2022) 20221–20226. https://doi.org/10.1021/jacs.2c09349.
249. S.-C. Fan, Y.-T. Li, Y. Wang, J.-W. Wang, Y.-Y. Xue, H.-P. Li, S.-N. Li, & Q.-G. Zhai, Amide-Functionalized Metal–Organic Frameworks Coupled with Open Fe/Sc Sites for Efficient Acetylene Purification. *Inorganic Chemistry*, **60** (2021) 18473–18482. https://doi.org/10.1021/acs.inorgchem.1c03044.
250. Y. Zhang, L. Wang, J. Hu, S. Duttwyler, X. Cui, & H. Xing, Solvent-dependent supramolecular self-assembly of boron cage pillared metal–organic frameworks for selective gas separation. *CrystEngComm*, **22** (2020) 2649–2655. https://doi.org/10.1039/D0CE00142B.

251. F. Luo, C. Yan, L. Dang, R. Krishna, W. Zhou, H. Wu, X. Dong, Y. Han, T.-L. Hu, M. O'Keeffe, L. Wang, M. Luo, R.-B. Lin, & B. Chen, UTSA-74: A MOF-74 Isomer with Two Accessible Binding Sites per Metal Center for Highly Selective Gas Separation. *Journal of the American Chemical Society*, **138** (2016) 5678–5684. https://doi.org/10.1021/jacs.6b02030.
252. L. Yang, L. Yan, Y. Wang, Z. Liu, J. He, Q. Fu, D. Liu, X. Gu, P. Dai, L. Li, & X. Zhao, Adsorption Site Selective Occupation Strategy within a Metal–Organic Framework for Highly Efficient Sieving Acetylene from Carbon Dioxide. *Angewandte Chemie International Edition*, **60** (2021) 4570–4574. https://doi.org/10.1002/anie.202013965.
253. M. Liu, X.-W. Gu, C. Lin, B. Dong, Z. He, B. Xie, X. Zhang, B. Li, H.-M. Wen, & J. Hu, Highly efficient separation of C_2H_2 from CO_2 and C_2H_4 enabled by an anion-pillared metalloporphyrin MOF with sandwich-like binding sites. *Journal of Materials Chemistry A*, **13** (2025) 1034–1041. https://doi.org/10.1039/D4TA07584F.
254. D. J. O'Hearn, D. Sensharma, A. Raza, A. A. Bezrukov, M. Vandichel, S. Mukherjee, & M. J. Zaworotko, Crystal engineering of a new platform of hybrid ultramicroporous materials and their C_2H_2/CO_2 separation properties. *Chemical Science*, **15** (2024) 17937–17943. https://doi.org/10.1039/D4SC03029J.
255. S. Dutta, S. Mukherjee, O. T. Qazvini, A. K. Gupta, S. Sharma, D. Mahato, R. Babarao, & S. K. Ghosh, Three-in-One C_2H_2-Selectivity-Guided Adsorptive Separation across an Isoreticular Family of Cationic Square-Lattice MOFs. *Angewandte Chemie International Edition*, **61** (2022). https://doi.org/10.1002/anie.202114132.
256. J.-H. Li, Y. Xie, M.-Y. Zhou, R.-B. Lin, & X.-M. Chen, Microporous Zinc Formate for Efficient Separation of Acetylene over Carbon Dioxide. *Chemical Research in Chinese Universities*, **38** (2022) 87–91. https://doi.org/10.1007/s40242-021-1380-3.
257. H.-J. Lv, Y.-P. Li, Y.-Y. Xue, Y.-C. Jiang, S.-N. Li, M.-C. Hu, & Q.-G. Zhai, Systematic Regulation of C_2H_2/CO_2 Separation by 3p-Block Open Metal Sites in a Robust Metal–Organic Framework Platform. *Inorganic Chemistry*, **59** (2020) 4825–4834. https://doi.org/10.1021/acs.inorgchem.0c00115.
258. C. Liu, Y. He, S. Wu, M. Shi, J. Hu, W. Zhu, Z. Gu, Y. Zhang, & L. Wang, C_2H_2/CO_2 Separation by a Carborane Hybrid 2D Metal–Organic Framework. *Inorganic Chemistry*, (2025). https://doi.org/10.1021/acs.inorgchem.5c01518.
259. X. Duan, Q. Zhang, J. Cai, Y. Yang, Y. Cui, Y. He, C. Wu, R. Krishna, B. Chen, & G. Qian, A new metal–organic framework with potential for adsorptive separation of methane from carbon dioxide, acetylene, ethylene, and ethane established by simulated breakthrough experiments. *Journal of Materials Chemistry A*, **2** (2014) 2628. https://doi.org/10.1039/c3ta14454b.
260. D. Sensharma, D. J. O'Hearn, A. Koochaki, A. A. Bezrukov, N. Kumar, B. H. Wilson, M. Vandichel, & M. J. Zaworotko, The First Sulfate-Pillared Hybrid Ultramicroporous Material, SOFOUR-1-Zn, and Its Acetylene Capture Properties. *Angewandte Chemie International Edition*, **61** (2022). https://doi.org/10.1002/anie.202116145.

261. Q. Dong, Y. Guo, H. Cao, S. Wang, R. Matsuda, & J. Duan, Accelerated C_2H_2/CO_2 Separation by a Se-Functionalized Porous Coordination Polymer with Low Binding Energy. *ACS Applied Materials & Interfaces*, **12** (2020) 3764–3772. https://doi.org/10.1021/acsami.9b20623.
262. H. Cui, S. Chen, H. Arman, Y. Ye, A. Alsalme, R.-B. Lin, & B. Chen, A microporous metal-organic framework of sql topology for C2H2/CO2 separation. *Inorganica Chimica Acta*, **495** (2019) 118938. https://doi.org/10.1016/j.ica.2019.05.037.
263. B.-Y. Zhu, T. Zhang, C.-H. Li, J.-W. Cao, Z.-Q. Zhang, W. Qi, G.-Y. Wang, Z.-H. Rong, Y. Wang, & K.-J. Chen, A (3,8)-Connected Metal–Organic Framework with Bending Dicarboxylate Linkers for C_2H_2/CO_2 Separation. *Inorganic Chemistry*, **61** (2022) 4555–4560. https://doi.org/10.1021/acs.inorgchem.2c00004.
264. M. Jiang, X. Cui, L. Yang, Q. Yang, Z. Zhang, Y. Yang, & H. Xing, A thermostable anion-pillared metal-organic framework for C2H2/C2H4 and C2H2/CO2 separations. *Chemical Engineering Journal*, **352** (2018) 803–810. https://doi.org/10.1016/j.cej.2018.07.104.
265. J. Duan, W. Jin, & R. Krishna, Natural Gas Purification Using a Porous Coordination Polymer with Water and Chemical Stability. *Inorganic Chemistry*, **54** (2015) 4279–4284. https://doi.org/10.1021/ic5030058.
266. S. Chen, N. Behera, C. Yang, Q. Dong, B. Zheng, Y. Li, Q. Tang, Z. Wang, Y. Wang, & J. Duan, A chemically stable nanoporous coordination polymer with fixed and free Cu2+ ions for boosted C2H2/CO2 separation. *Nano Research*, **14** (2021) 546–553. https://doi.org/10.1007/s12274-020-2935-1.
267. O. T. Qazvini, R. Babarao, & S. G. Telfer, Multipurpose Metal–Organic Framework for the Adsorption of Acetylene: Ethylene Purification and Carbon Dioxide Removal. *Chemistry of Materials*, **31** (2019) 4919–4926. https://doi.org/10.1021/acs.chemmater.9b01691.
268. Z. Di, C. Liu, J. Pang, C. Chen, F. Hu, D. Yuan, M. Wu, & M. Hong, Cage-Like Porous Materials with Simultaneous High C_2H_2 Storage and Excellent C_2H_2/CO_2 Separation Performance. *Angewandte Chemie International Edition*, **60** (2021) 10828–10832. https://doi.org/10.1002/anie.202101907.
269. H. Yang, T. X. Trieu, X. Zhao, Y. Wang, Y. Wang, P. Feng, & X. Bu, Lock-and-Key and Shape-Memory Effects in an Unconventional Synthetic Path to Magnesium Metal–Organic Frameworks. *Angewandte Chemie International Edition*, **58** (2019) 11757–11762. https://doi.org/10.1002/anie.201905876.
270. H. Li, C. Liu, C. Chen, Z. Di, D. Yuan, J. Pang, W. Wei, M. Wu, & M. Hong, An Unprecedented Pillar-Cage Fluorinated Hybrid Porous Framework with Highly Efficient Acetylene Storage and Separation. *Angewandte Chemie International Edition*, **60** (2021) 7547–7552. https://doi.org/10.1002/anie.202013988.
271. D. Sensharma, B. H. Wilson, N. Kumar, D. J. O'Hearn, & M. J. Zaworotko, Pillar Modularity in **fsc** Topology Hybrid Ultramicroporous Materials Based upon Tetra(4-pyridyl)benzene. *Crystal Growth & Design*, **22** (2022) 5472–5480. https://doi.org/10.1021/acs.cgd.2c00561.

272. G. Du, Y. Wang, T. Liu, Z. Yue, Y. Ma, & D. Xue, Isoreticular Contraction in Dicopper Paddle-Wheel-Based Metal-Organic Frameworks to Enhance C_2H_2/CO_2 Separation. *Chemistry – A European Journal*, **31** (2025). https://doi.org/10.1002/chem.202403478.

273. S. Sharma, S. Mukherjee, A. V. Desai, M. Vandichel, G. K. Dam, A. Jadhav, G. Kociok-Köhn, M. J. Zaworotko, & S. K. Ghosh, Efficient Capture of Trace Acetylene by an Ultramicroporous Metal–Organic Framework with Purine Binding Sites. *Chemistry of Materials*, **33** (2021) 5800–5808. https://doi.org/10.1021/acs.chemmater.1c01723.

274. X. Wang, B. Wang, X. Zhang, Y. Xie, H. Arman, & B. Chen, A Copper-Based Metal–Organic Framework for C_2H_2/CO_2 Separation. *Inorganic Chemistry*, **60** (2021) 18816–18821. https://doi.org/10.1021/acs.inorgchem.1c02552.

275. W. Fan, X. Wang, X. Liu, B. Xu, X. Zhang, W. Wang, X. Wang, Y. Wang, F. Dai, D. Yuan, & D. Sun, Regulating C_2H_2 and CO_2 Storage and Separation through Pore Environment Modification in a Microporous Ni-MOF. *ACS Sustainable Chemistry & Engineering*, **7** (2019) 2134–2140. https://doi.org/10.1021/acssuschemeng.8b04783.

276. R. Liu, Q.-Y. Liu, R. Krishna, W. Wang, C.-T. He, & Y.-L. Wang, Water-Stable Europium 1,3,6,8-Tetrakis(4-carboxylphenyl)pyrene Framework for Efficient C_2H_2/CO_2 Separation. *Inorganic Chemistry*, **58** (2019) 5089–5095. https://doi.org/10.1021/acs.inorgchem.9b00169.

277. L. Zhou, C. Jiang, A. Maibam, R. Babarao, D. Sun, D. Acharya, & P. Cui, Tailoring Pore Environments in Metal–Organic Frameworks for Efficient C_2H_2/CO_2 and C_2H_2/C_2H_4 Separations. *Small*, **21** (2025). https://doi.org/10.1002/smll.202412205.

278. L. Zhang, T. Xiao, X. Zeng, J. You, Z. He, C.-X. Chen, Q. Wang, A. Nafady, A. M. Al-Enizi, & S. Ma, Isoreticular Contraction of Cage-like Metal–Organic Frameworks with Optimized Pore Space for Enhanced C_2H_2/CO_2 and C_2H_2/C_2H_4 Separations. *Journal of the American Chemical Society*, **146** (2024) 7341–7351. https://doi.org/10.1021/jacs.3c12032.

279. W. J. F. Trenholme, D. I. Kolokolov, M. Bound, S. P. Argent, J. A. Gould, J. Li, S. A. Barnett, A. J. Blake, A. G. Stepanov, E. Besley, T. L. Easun, S. Yang, & M. Schröder, Selective Gas Uptake and Rotational Dynamics in a (3,24)-Connected Metal–Organic Framework Material. *Journal of the American Chemical Society*, **143** (2021) 3348–3358. https://doi.org/10.1021/jacs.0c11202.

280. H. Zeng, M. Xie, Y. Huang, Y. Zhao, X. Xie, J. Bai, M. Wan, R. Krishna, W. Lu, & D. Li, Induced Fit of C_2H_2 in a Flexible MOF Through Cooperative Action of Open Metal Sites. *Angewandte Chemie International Edition*, **58** (2019) 8515–8519. https://doi.org/10.1002/anie.201904160.

281. G. Chang, B. Li, H. Wang, T. Hu, Z. Bao, & B. Chen, Control of interpenetration in a microporous metal–organic framework for significantly enhanced C_2H_2/CO_2 separation at room temperature. *Chemical Communications*, **52** (2016) 3494–3496. https://doi.org/10.1039/C5CC10598F.

282. A. Rohatgi, WebPlotDigitizer (Version 5.2). (2025). https://doi.org/https://automeris.io/.

283. D. Frenkel, B. Smit, & M. A. Ratner, *Understanding Molecular Simulation: From Algorithms to Applications*. *Physics Today*, **50** (1997) 66–66. https://doi.org/10.1063/1.881812.

284. BIOVIA, Dassault Systèmes, Material Studio, Dassault Systèmes, . (2023).

285. J. Wellendorff, K. T. Lundgaard, A. Møgelhøj, V. Petzold, D. D. Landis, J. K. Nørskov, T. Bligaard, & K. W. Jacobsen, Density functionals for surface science: Exchange-correlation model

development with Bayesian error estimation. *Physical Review B*, **85** (2012) 235149. https://doi.org/10.1103/PhysRevB.85.235149.

286. G. Kresse & J. Furthmüller, Efficiency of ab-initio total energy calculations for metals and semiconductors using a plane-wave basis set. *Computational Materials Science*, **6** (1996) 15–50. https://doi.org/10.1016/0927-0256(96)00008-0.
287. G. Kresse & D. Joubert, From ultrasoft pseudopotentials to the projector augmented-wave method. *Physical Review B*, **59** (1999) 1758–1775. https://doi.org/10.1103/PhysRevB.59.1758.
288. P. E. Blöchl, Projector augmented-wave method. *Physical Review B*, **50** (1994) 17953–17979. https://doi.org/10.1103/PhysRevB.50.17953.
